# The strain-dependent cytostatic activity of *Lactococcus lactis* on CRC cell lines is mediated through the release of arginine deiminase

**DOI:** 10.1186/s12934-024-02345-w

**Published:** 2024-03-14

**Authors:** Rafał Jastrząb, Rafał Tomecki, Aneta Jurkiewicz, Damian Graczyk, Agnieszka K. Szczepankowska, Jennifer Mytych, Damian Wolman, Pawel Siedlecki

**Affiliations:** 1 Institute of Biochemistry and Biophysics, Polish Academy of Sciences, Adolfa Pawińskiego 5A, Warsaw, 02-106 Poland; 2 Olimp Laboratories, Pustynia 84F, Debica, 39-200 Poland; 3 Faculty of Biology, University of Warsaw, Miecznikowa 1, Warsaw, 02-089 Poland

**Keywords:** Probiotics, Postbiotics, Cell-free supernatant, Anti-cancer, Cytostatic, Arginine deiminase, *Lactococcus lactis*, Lactic acid bacteria, Protein release, Therapeutic vector

## Abstract

**Background:**

Colorectal cancer (CRC) is one of the most commonly diagnosed cancers, posing a serious public health challenge that necessitates the development of new therapeutics, therapies, and prevention methods. Among the various therapeutic approaches, interventions involving lactic acid bacteria (LAB) as probiotics and postbiotics have emerged as promising candidates for treating and preventing CRC. While human-isolated LAB strains are considered highly favorable, those sourced from environmental reservoirs such as dairy and fermented foods are also being recognized as potential sources for future therapeutics.

**Results:**

In this study, we present a novel and therapeutically promising strain, *Lactococcus lactis* ssp. *lactis* Lc4, isolated from dairy sources. Lc4 demonstrated the ability to release the cytostatic agent - arginine deiminase (ADI) - into the post-cultivation supernatant when cultured under conditions mimicking the human gut environment. Released arginine deiminase was able to significantly reduce the growth of HT-29 and HCT116 cells due to the depletion of arginine, which led to decreased levels of c-Myc, reduced phosphorylation of p70-S6 kinase, and cell cycle arrest. The ADI release and cytostatic properties were strain-dependent, as was evident from comparison to other *L. lactis* ssp. *lactis* strains.

**Conclusion:**

For the first time, we unveil the anti-proliferative properties of the *L. lactis* cell-free supernatant (CFS), which are independent of bacteriocins or other small molecules. We demonstrate that ADI, derived from a dairy-Generally Recognized As Safe (GRAS) strain of *L. lactis*, exhibits anti-proliferative activity on cell lines with different levels of argininosuccinate synthetase 1 (*ASS1*) expression. A unique feature of the Lc4 strain is also its capability to release ADI into the extracellular space. Taken together, we showcase *L. lactis* ADI and the Lc4 strain as promising, potential therapeutic agents with broad applicability.

**Supplementary Information:**

The online version contains supplementary material available at 10.1186/s12934-024-02345-w.

## Background

Colorectal cancer (CRC) is a life-threatening disease with one of the highest global prevalence and mortality. Based on the World Health Organization (WHO) statistics, 2 million new cases of CRC and 1 million deaths were recorded in 2022, which places CRC in 3rd position as the most lethal cancer [1]. Two types of CRC can be distinguished based on etiology. The sporadic CRCs, with no family history or genetic predispositions, account for approximately 65% of total CRCs diagnosed worldwide [2], whereas inherited types of CRCs constitute the remaining 35% [3, 4]. CRC development may take decades to transform into the final form of cancer. Many factors could modify the pace of this process, including diet, alcohol, and tobacco consumption, as well as contaminants present in water and food [5, 6]. Recently, the gut microbiota (GM) has also been correlated with CRC progression and found to be involved in modulating inflammatory pathways or causing initial inflammation, as well as modifying host metabolism and production of toxic metabolites [7–9].

GM takes part in multiple processes occurring in the gastrointestinal tract, i.e. food digestion, hormone and immune regulation, and toxin neutralization [8, 10] During CRC progression, specific molecular interactions are established between the healthy gut microbiome, tumor microbiome (TM) - found close to the tumor, and the host immune system. TM negatively affects the healthy gut microbiome, leading to disrupted host immune responses, resulting in limited efficacy of therapy response [11, 12]. Specific microbes, small molecules (e.g. short-chain fatty acids), dietary interventions, and their combinations are promising factors for slowing down disease progression and lowering CRC incidence [13].

In recent years, interventions with lactic acid bacteria (LAB), especially in the form of probiotics, and - most recently - postbiotics, have gained considerable attention [14–16]. Furthermore, LAB sourced from food, such as dairy or fermented products, have emerged as promising alternatives to gut-isolated bacteria, offering potential benefits as probiotics or postbiotics [17–20]. Probiotics are live microorganisms that are safe and exert beneficial effects on human health when consumed or applied to the body [21]. Different probiotics were shown to affect CRC via varying mechanisms, e.g. by decreasing the expression of the leptin receptor, one of the risk factors for CRC development [22], acting as enzymatic detoxification agents, shaping the overall microbial community [23–25], and producing modulating metabolites affecting cancer cells or immune response [26, 27]. Mechanisms of the positive influence of probiotics were recently reviewed by [28]. On the other hand, probiotics have known limitations. Among the most prominent are: the possible transfer of antibiotic resistance genes [29–31], reduced shelf life [32], and risk of bacteremia development, especially when administered to immunocompromised patients [33, 34]. To mitigate the above concerns, the concept of postbiotics has been explored. Postbiotics may contain intact inanimate microbial cells and/or microbial cell fragments/structures (cell walls, membranes, exopolysaccharides, pilins, etc.), with or without metabolites and end products (i.e. proteins, exopolysaccharides, organic acids, other small metabolites), that confer benefits to the host ( [35–39]. Postbiotics hold promise for future development as cytostatic or cytotoxic agents or modulatory factors targeting both the disrupted microbiome and host immune cells against CRC [28, 35–38, 40–42].

*Lactococcus lactis*, the well-known Gram-positive lactic acid bacteria used in the food industry, have recently gained attention as a prospective therapeutic. *L. lactis* strains have been shown to be a promising host for heterologous protein expression [43–45] and for the production of native bacteriocins (e.g. nisin and lactococcin) or other antimicrobial peptides (AMPs) [46, 47] with cytostatic activity. Additionally, several reports suggested that not only bacteriocins secreted to *L. lactis* cell-free supernatant (CFS) can exhibit cytostatic activity [48, 49]. Hosseini and Kim [50, 51] reported that cytoplasmic extracts, cell walls, and whole-cell lysates may also display such properties. To date, only a handful of clearly defined active molecules from LAB with confirmed therapeutic effects have been identified. For example, the p40 protein isolated from the CFS of a LAB strain *Lacticaseibacillus rhamnosus* was found to exert anti-inflammatory effects in vitro [52, 53]. A catalase derived from the *Lactococcus lactis* htrA-NZ9000 strain improved the inflammatory status and reduced colonic damage in BALB/c mice with 1,2-dimethylhydrazine (DMH)-induced CRC [54].

The main objective of this study was to identify the factor(s) responsible for the anti-proliferative (cytostatic) effect observed for a dairy-origin LAB strain, *Lactococcus lactis* spp. *lactis* Lc4. Such an effect was noted when applying the Lc4-derived CFS on two CRC cell lines - HCT116 and HT-29. We aimed to identify the main native molecule(s) responsible for this effect and to elucidate its mechanism of action.

## Materials and methods

### Bacterial strains, media, and growth conditions

In the study, we tested three *Lactococcus lactis* spp. *lactis* strains (Lc2, Lc3, Lc4), obtained from the Institute of Biochemistry and Biophysics Polish Academy of Sciences (IBB PAS) collection of LAB. To imitate temperature and oxygen availability in the human gut, bacteria were cultured in M17 broth (Millipore, cat. no. 56,156) + 0.5% glucose (GM17) anaerobically without agitation at 37 °C for 24 h unless otherwise stated.

Lc4 strain ability to survive in the gastrointestinal tract (GI) has been checked anaerobically without agitation at 37 °C for 24 h, using a growth medium (Feed; ProDigest cat. no. PD-NM001B), with or without pancreatic juice (PJ; 6 g/L of bile acids (Difco^™^ Oxgall cat. no. 212,820), 12,5 g/L of NaHCO_3_, and 0,9 g/L of pancreatin (Sigma Aldrich, cat.no. P1625), designed for the artificial SHIME® (Simulator of a Human Intestinal Microbial Ecosystem, ProDigest) digestive system [55–57]. The growth medium was neutralized with NaHCO_3_ if necessary. To verify the enzymatic activity of the ADI pathway in GI, Feed, and Feed + PJ were enriched by the addition of 10 mM L-arginine.

### Supernatants, fractions preparation, and proteolysis

Bacterial cell cultures were conducted for 24 h at the conditions described above. Then, cell-free supernatants (CFSs) were collected via centrifugation at 4 724 RCF for 10 min at 4℃. The pH of the CFSs was adjusted to 6.4 by the addition of 1 M NaOH to rule out acid influence in subsequent tests and filtered through 0.22 μm syringe filters (VWR, cat. no. I514-0073_P) to obtain a sterile solution free of bacterial cells.

To characterize the size of active bacterial products, bacterial CFSs were subjected to ultrafiltration through centrifugal concentrators with polyethersulfone (PES) membrane (Sartorius Vivaspin®, cat. no. VS15T91, VS15T01, VS15T31 or Vivacell® cat. no. VC1032) with defined molecular weight cut-offs (MWCO) − 3 kDa, 10 kDa, 50 kDa. Retained and filtered fractions were adjusted to the initial volume by adding the cell culture medium (Dulbecco’s Modified Eagle Medium; DMEM) directly before experiments.

The concentration of proteins in tested supernatants was measured using the Bradford method [58]. To confirm the possible influence of proteins on observed effects, the supernatants were treated with a mix of trypsin (Roth, cat. no. 2193.2) and chymotrypsin (Roth, cat. no. 0238.3) (1 mg/ml each) for 20 h at 37℃. After incubation, proteases were inactivated by exposure to high temperature (99 °C) for 2 min. Samples were centrifuged at 18 188 RCF for 10 min and the supernatants were used in subsequent experiments.

### Purification of proteins from high molecular weight CFS fraction (F > 50 kDa)

Three types of liquid chromatography columns were used to purify and identify proteins responsible for the observed activity - anion exchange column and two size exclusion columns. All active fractions were obtained from concentrated > 50 kDa fractions (F > 50 kDa), prepared as described above.

#### Anion exchange chromatography

F > 50 kDa was subjected to anion exchange chromatography on a Resource Q column (1 ml; GE Healthcare, Piscataway, NJ, USA) that had been pre-equilibrated with 10 mM-HEPES buffer (pH 6.5; adjusted with NaOH) containing 0.1 M NaCl. The loading volume was 1 ml. After loading, the column was washed with 30 ml of the same buffer and then eluted with a linear gradient (0.1-1.0 M) of NaCl in 42 ml of the 10 mM HEPES-NaOH (pH 6.5) buffer.

#### Size exclusion chromatography

Two types of columns were used: Superdex 75 10/300 GL and Superdex 200 10/300 GL (GE Healthcare, Piscataway, NJ, USA). In both cases, columns were washed with a 10 mM HEPES buffer (pH 6.5) containing 0.1 M NaCl. 1 ml of F > 50 kDa was loaded into the column and then eluted with the same buffer.

To analyze protein content, all obtained fractions were resolved in SDS-PAGE gels, which were then stained with Coomassie brilliant blue R250 (Sigma Aldrich, cat. no. C.I. 42,660) or PageBlue Protein Staining Solution (Thermo Fisher Scientific, cat. no. 24,620). Selected fractions were subsequently used for cell proliferation assays. Fractions that exhibited anti-proliferative (cytostatic) activity were subjected to mass spectrometry (MS) analysis and directly compared in SDS-PAGE gel. Selected bands (around 70 kDa, 45 kDa, 35 kDa), common for all lanes, were excised and subjected to the identification of protein content by mass spectrometry.

### Proteomic analysis

Cytostatic active fractions and bands excised from SDS-PAGE were subjected to mass spectrometry analysis using the LC-MS/MS system in the Laboratory of Mass Spectrometry (IBB PAS, Warsaw). Briefly, active fractions were reduced by incubation at 57˚C for 30 min with 10 mM 1,4-dithiothreitol (DTT) and 10 mM ammonium bicarbonate buffer, alkylated with 50 mM of indole-3-acetic acid (IAA) at room temperature for 45 min without light access, and then digested overnight at 37 °C by addition of 10 ng/ml trypsin (cat no. V5111, Promega, Madison, WI, USA). Finally, the digestion was terminated by the addition of a 5% trifluoroacetic (TFA) acid solution. The digest was centrifuged at 4 °C and 14 000 RCF for 30 min to pellet solids.

In the case of excised bands, they were additionally destained by a mix of 50% acetonitrile and 50 mM NH_4_CO_3_, dried by 100% solution of acetonitrile, then reduced and alkylated. Before digestion, bands were washed (100 mM NH_4_CO_3_) and dried again. Peptide extraction was performed by treatment with 0.1% TFA and 2% acetonitrile. The particle-free supernatant was analyzed using a nanoAcquity UPLC system (Waters) coupled to an Orbitrap QExative mass spectrometer (Thermo Fisher Scientific, Waltham, MA, USA). Acquired spectra were used for searching against a protein sequence database (UniProt) using the Mascot search engine (matrixscience.com), followed by validation and formatting of results.

The obtained results were mapped to the UniProtKB database using Uniprot web services. Results containing Gene ontology (GO) terms were curated and subjected to comparative proteomic using Python scripts. GO terms have been analyzed and visualized using ShinyGO 0.77 [59] or GraphPad Prism.

### Cell lines and culture conditions

Cells were cultured in a humidified incubator with 5% CO_2_ at 37 °C. The HT-29 (ATCC® HTB-38™) and HCT116 (ATCC® CCL-247™) colorectal cancer cell lines were grown in DMEM medium (VWR, cat. no. C392-0417) supplemented with 10% (v/v) fetal bovine serum (EurX, cat. no. E5050), 2 mM L-glutamine, penicillin (100 U/ml), streptomycin (100 U/ml) (Biowest, cat. no. L0014) unless otherwise stated.

### MTS assay

Cell metabolic activity was evaluated using the MTS (CellTiter 96® AQueous One Solution Cell Proliferation) assay. This assay is based on the conversion of MTS (3-(4,5-dimethylthiazol-2-yl)-5-(3-carboxymethoxyphenyl)-2-(4-sulfophenyl)-2 H-tetrazolium inner salt) to a soluble purple formazan by NADPH or NADH produced by dehydrogenase enzymes in metabolically active cells. HT-29 and HCT116 cells were seeded at a density of 5 × 10³ cells/well in a 96-well plate 24 h before the experiment, then treated with selected CFS at selected concentrations (10%, 15%, 20%, 25%, 33% (v/v)) and incubated at 37 °C in 5% CO_2_ for 72 h. Before the addition of the MTS reagent, the incubation mixture was replaced with 100 µl of fresh DMEM medium. The MTS assay was performed according to the manufacturer’s instructions (Promega, Madison, WI, USA, cat. no. G3581). Briefly, 20 µl of MTS solution was added to each 96-well microplate. The microplate was incubated at 37 °C in 5% CO_2_ for 4 h until the purple formazan appeared. Next, the readout was made at 490 nm using a microplate reader (Thermo Fisher Scientific, CA, USA, Varioscan Lux).

### Cell proliferation assay

Cells were plated at a density of 5 × 10³ cells/well in a 96-well plate 24 h before the experiment. Tested CFSs or fractions from molecular filtration diluted in DMEM were added at the final concentration of 33% (v/v) in 200 µl DMEM medium. Fractions obtained from protein purification methods were added into the wells at the final concentration of 5% (v/v). The confluence of cells as a proxy of proliferation was monitored for 72 h at 2 h intervals using the IncuCyte S3 live-cell imaging system (Essen BioScience) and analyzed using IncuCyte 2019B Rev2 software. To verify the anti-proliferative (cytostatic) effect, the growth rate constants were estimated from the equation: µ = ( (log10 N - log10 N0) 2.303) / (t - t0) where µ is a growth rate, N is confluence at t = 68 h, N0 confluence at t0 = 6 h [60].

### Cell cycle analysis

The HT-29 cells were seeded in 6-well cell culture plates 24 h prior to the experiment at a density of 25 × 10^4^ cells/well. Tested IEX fractions were prepared as stock solutions (10% v/v) in DMEM before the experiment and diluted 1:1 with DMEM or DMEM supplemented with 10 mM of L-arginine. Cells were treated with prepared solutions and after 24 h medium was removed, cells were collected by trypsinization, washed with phosphate-buffered saline (PBS), and dehydrated with cold 70% ethanol for 1 h. Subsequently, the DNA content of the nuclei was determined by staining nuclear DNA with 50 µg/mL propidium iodide, followed by flow cytometry (Attune™ NxT Flow Cytometer, Thermo Fisher Scientific). The proportion of nuclei in each phase of the cell cycle was determined using Attune™ NxT Software.

### Glucose and lactate determination

Measurements of glucose changes over time were performed in the same experimental design as described above (see Cell proliferation assay) using a blood glucometer (Contour® plus ONE ref. 84,656,348, Ascensia Diabetes Care AG) and dedicated strips (Contour® plus ref. 84,581,896, Ascensia Diabetes Care AG). One drop of cell culture supernatant of F-IEX-treated HT-29 and HCT116 was transferred on top of a strip and readout was noted. Measurements were made at three time points (0 h, 48 h, and 72 h). All measurements were performed in triplicate.

To determine lactate levels, HT-29 and HCT116 cells were seeded on a 96-well plate and treated with F > 50 kDa and F-IEX fractions as previously described (see Cell proliferation assay). Lactate concentration was measured after 72 h of treatment using a portable lactate sensor (Lactate Scout+, SensLab GmbH) with dedicated strips (Lactate Scout Sensors ref. 7023-3405-0846, SensLab GmbH). All measurements were performed in triplicate.

### Western blot analysis

The HT-29 and HCT116 cells were seeded in 6 cm cell culture dishes at a density of 6 × 10^5^ cells/dish. The following day, the cells were treated with tested fractions and incubated for 24 h. Then, the supernatant was removed, cells were washed twice with ice-cold PBS and harvested by scraping directly into lysis buffer (100 mM NaCl, 50 mM HEPES-NaOH pH 7.9, 1 mM EDTA, 5% glycerol, 0.05% NP-40, 0.1% SDS, proteases and phosphatases inhibitors − 10 mM sodium fluoride (Sigma Aldrich, cat. no. 201,154), 0.2 mM sodium orthovanadate (Sigma Aldrich, cat. no. S6508), 10 mM β-glycerophosphate (Sigma Aldrich, cat. no. G5422), 1:100 cOmplete protease inhibitor cocktail (Roche, cat. no. 11,836,145,001), rapidly frozen in liquid nitrogen, and stored at -20℃. To extract proteins, lysates were thawed on ice and sonicated using the Bioruptor XL (Diagenode, cat. no. UCD-500) and spun for 10 min at 20 000 RCF at 4 °C. Protein content was verified by the Pierce method (Pierce BCA Protein Assay Kit, cat. no. 23,227). An equal amount of protein was precipitated by the addition of 1:1 (v/v) of 10% trichloroacetic acid (TCA) and incubated for 20 min on ice. Samples were spun for 10 min at 20 000 RCF at 4 °C, and the pellet was washed with cold acetone, dried, and resuspended in Laemmli buffer (31.5 mM Tris-HCl pH 8.8, buffer 10% glycerol, 1% SDS, 0.005% Bromophenol Blue, 355 mM 2-mercaptoethanol), followed by incubation for 15 min at 55 °C with shaking. After denaturation for 5 min at 95 °C, proteins were resolved in SDS-PAGE gels, transferred via wet transfer onto a nitrocellulose membrane (GE Healthcare, cat. no. GE10600001), and incubated overnight with primary antibodies in dilution 1:1000 at 4 °C. Antibodies used in this study: anti-phospho-p70-S6 Kinase (Thr389) (Cell Signaling, cat. no. 9234s), anti-c-Myc (Santa Cruz Biotechnology, cat. no. sc-764), anti-pan-actin (Sigma-Aldrich, cat. no. MAB1501). Then, membranes were washed 4 times for 7 min in 1 x Tris-buffered saline with 0.1% Tween-20 (TBST), and incubated for 1 h with a secondary antibody (Sigma-Aldrich, cat. no. 401,215, 401,393). After washing with TBST, membranes were incubated with ECL-reagent (BioRad, cat. no. 1,705,061) according to the manufacturer’s protocol. Membranes were developed using standard X-ray films (AGFA, cat. no. EWPJH) or the chemiluminescence imaging system (Alliance Q9 Advanced, Uvitec, Cambridge).

### Genome sequencing and annotation

The arginine deiminase gene (*arcA*) sequence (Supplementary Table 3) was obtained from whole genome sequencing (DNA Sequencing and Synthesis Facility of IBB PAS) and annotation of the Lc4 strain, using the previously described method [48]. Briefly, DNA was isolated using the Genomic Maxi AX kit (A&A Biotechnology, cat. no. 995 − 10) and sequenced in a hybrid mode using the following sequencers:

i) Illumina MiSeq (Illumina, San Diego, United States) - the library was constructed using the NEB Ultra II FS kit (New England Biolabs, cat. no. e7805), and then sequenced using the MiSeq v3 600-cycle chemistry kit (Illumina, cat. no. MS-102-3003) cartridge in the mode of paired 2 × 300 bp reads.
ii) Oxford Nanopore GridION (Oxford Nanopore Technologies, Oxford, United Kingdom) - the library was constructed with the 1D Ligation Kit (LSK109) and barcoded with ONT Native barcodes. Samples were sequenced using Flow Cell R9.4.1 and the GridION instrument. The genome assembly has been performed using the Unicycler v.0.4.8 program [61]. The remaining ambiguities in the genome assemblies were verified by PCR and Sanger sequencing. Sequence correction was performed using the SeqMan program from the DNAStar package [62]. The genome annotation was performed using the PATRIC v2 database [63]. The nucleotide and protein sequence of ArcA/ADI were presented in Supplementary Table 3. The genomic data are available for request.

### Plasmids, heterologous protein production, and purification

The arginine deiminase gene (*arcA*) was PCR amplified from the custom DNA constructs (GeneArt, Thermo Scientific; Supplementary Table 3) using primers listed in Supplementary Table 3. The purified PCR product was ligated into a blunt TOPO vector (Invitrogen, cat. no. 450,245) and used for the transformation of the chemically competent *E. coli* MH1 strain. Based on Sanger sequencing with M13fwd and M13 rev primers (Supplementary Table 3), the selected recombinant plasmid was isolated using a Plasmid Mini kit (A&A Biotechnology, cat. no. 020–250). An insert was excised with *Nco*I and *Xho*I restriction enzymes (Thermo Fisher Scientific, cat. no. FD0574, FD0694), purified following gel electrophoresis using Gel-Out kit (A&A Biotechnology, cat. no. 023–250), ligated into the pET28 C-6xHis-Tag (pET) vector (Sigma Aldrich, cat. no. 69,864), digested with the same restriction endonucleases and additionally treated with FastAP Thermosensitive Alkaline Phosphatase (Thermo Fisher Scientific, cat. no. EF0652), and cloned using *E. coli* MH1 strain. Sanger sequencing with T7 and T7ter primers (Supplementary Table 3) was used to validate the insert sequence in selected recombinant plasmids.

Site-directed mutagenesis with the primer pair ARCA_C400A_F and ARCA_C400A_R (Supplementary Table 3) was employed to generate a vector for the expression of ADI with catalytic C400A mutation.

Expression vectors with inserts corresponding to ADI WT or ADI C400A were used for the transformation of chemically competent *E. coli* BL21-CodonPlus (DE3)-RIL (Agilent, cat. no. 230,245). Transformants were then grown overnight at 37 °C in standard Luria-Broth (LB) medium with 50 µg/ml kanamycin and 34 µg/ml chloramphenicol. This starter culture was subsequently used to inoculate 1 l of Auto Induction Medium (AIM) Super Broth Base including Trace elements (Formedium) with 2% glycerol and the above-mentioned antibiotics. Bacteria were then grown for 72 h at 18 °C with shaking (150 rpm) and collected by centrifugation at 5 938 RCF in a Sorvall H6000A/HBB6 swinging-bucket rotor for 15 min at 4 °C.

The bacterial pellet was resuspended in 100 ml of lysis buffer (50 mM Tris-HCl pH 7.4, 50 mM NaCl, 10 mM imidazole, 10 mM 2-mercaptoethanol, 1 mM phenylmethylsulfonyl fluoride [PMSF]), incubated with lysozyme (50 µg/ml; Roth) for 30 min in a cold room, and then broken in an EmulsiFlex-C3 High Pressure homogenizer at 1500 Bar. The homogenate was centrifuged in a Sorvall WX Ultra Series ultracentrifuge (F37L rotor) at 137 380 RCF for 45 min at 4 °C.

The lysate was used for protein purification using the ÄKTA Xpress system (GE Healthcare), employing nickel affinity chromatography on an ÄKTA-compatible 5 ml column that was filled with Ni-NTA Superflow resin (Qiagen). The column was equilibrated with 25 ml of low-salt (LS) buffer (50 mM Tris-HCl pH 7.4, 50 mM NaCl, 10 mM imidazole) before lysate loading. After protein binding, the column was sequentially washed with 40 ml of LS buffer, 25 ml of high-salt (HS) buffer (50 mM Tris-HCl pH 7.4, 1 M NaCl, 10 mM imidazole), and finally with 20 ml of LS buffer. Resin-bound proteins were recovered by elution with 30 ml of buffer E (50 mM Tris-HCl pH 7.4, 50 mM NaCl, 300 mM imidazole). Purification on Ni-NTA resulted in one peak after elution.

During the next step of purification, pooled elution fractions (less than 1/10th of available material) were subjected to gel filtration on size exclusion Superdex 75 10/300 GL column (GE Healthcare) using 1.2 column volumes of gel-filtration (GF) buffer (10 mM HEPES pH 6.5, 0.1 M NaCl) resulted in asymmetric peak (Supplementary Fig. 5a). SDS-PAGE analysis of fractions covered by this peak (Supplementary Fig. 5a) showed that ADI was contaminated with some chaperones and other proteins of similar size. Fractions marked between red lines on the chromatogram were pooled (red rectangle on SDS-PAGE) and used for another step of purification using an ion exchange column - Resource Q (buffers and conditions were the same as in the case of purification of F > 50 kDa). IEX purification resulted in an asymmetric peak (Supplementary Fig. 5b), whose left part contains a chaperone and a mix of ADI and chaperone, which were present as two bands in SDS-PAGE analysis (Fig. 5a; Supplementary Fig. 5b). The right side of the peak contained lower amounts of ADI protein, which additionally were contaminated by other proteins (Supplementary Fig. 5b, SDS-PAGE gel). Based on that and on the cell proliferation assays of all fractions (Supplementary Fig. 5c), fractions with ADI and chaperone were chosen for further experiments.

### ADI enzymatic activity assay

Enzymatic activity assays were performed as previously described [64], with slight modifications. Briefly, 1 ml of reaction volume contained 10 mM of L-arginine, tested fractions (F > 50 kDa, F-IEX), or heterologously expressed ADI protein in 10 mM phosphate buffered saline (PBS) with pH adjusted to 6.4. Mixtures were incubated for 1 h at 37℃. The reaction was terminated by the addition of 0.5 ml of 20% TCA and incubated for 10 min on ice to allow proteins to precipitate. Samples were spun at 14 000 RCF for 10 min at 4℃. 500 µl of supernatant was mixed with 150 µl of diacetyl monoxime (5% w/v) and 750 µl of 1:3 mixture (v/v) of H_2_SO_4_ and H_3_PO_4_ and incubated for 30 min in the dark at 99℃. The amount of L-citrulline formed during the incubation was determined by measuring optical density (OD) at 460 nm and referred to the standard curve. All assays included controls without the enzyme and the substrate. Enzymatic activity of F > 50 kDa was calculated in relation to immediately TCA-precipitated F > 50 kDa incubation mixture, to determine residual L-citrulline content. One U of ADI activity was defined as the amount of enzyme activity that converts 1 µmol of arginine into 1 µmol of citrulline in 1 min at 37 °C. Specific enzymatic activity (U) was presented per milligram of protein. Protein concentration was determined using the Bradford method [58] in the case of F > 50 kDa and the Pierce method in the case of purified protein [65].

### Bacterial viability and cell damage assay

Viability assays were performed using the BD™ Cell Viability Kit (BD Biosciences, cat. no. 349,483). The assay relies on the dye thiazole orange (TO), a permanent dye, to stain all cells, as well as propidium iodide (PI) which leaks into cells with compromised membranes and stains injured and dead cells. The assay was performed according to the manufacturer’s recommendations, with slight modifications. Briefly, 1 ml of bacterial cultures were centrifuged at 4 724 RCF for 5 min at 4℃, washed twice with 0.9% NaCl, and resuspended in maximum recovery media (Sigma Aldrich, cat. no. 07233), then diluted 1:1 in the same medium. 500 µl of cell suspension was mixed with 1 µl of propidium iodide and 2.5 µl of thiazole orange, vortexed, and incubated for 5 min at room temperature. Samples were acquired on a BD FACS™ flow cytometer equipped with a 488-nm excitation laser and analyzed by BD CellQuest™ software. The side scatter (SSC) threshold was used to discriminate microbial cells from impurities. Cells were gated using SSC and separate fluorescence channel (FL) 2, as previously described [66–69]. The discrimination between live, dead, and injured populations was performed on the FL1 vs. FL3 plot. Gates were adjusted on live cells from the logarithmic growth phase (30℃, GM17 medium), injured by 5 min treatment with 0.05% Triton X-100 (Sigma Aldrich, cat. no. 93,443), and dead cells, which were killed with 70% isopropanol. Based on the presented data, gates were adjusted for further experiments. The total number of events collected for each sample was 10 000.

### SEM & TEM analyses

For both microscopy analyses, the *L. lactis* Lc4 strain was cultivated in the same conditions as described before (GM17 medium, 24 h, anaerobic static conditions, 37℃).

#### Scanning electron microscopy (SEM)

Bacterial cells were fixed with 2.5% glutaraldehyde (reducing fixative) buffered with 0.1 M cacodylate buffer pH 7.2 at room temperature for 24 h. Then, the cells were washed with cacodylate buffer (3 × 10 min), post-fixed with 1% aqueous solution of osmium tetroxide (oxidative fixative) for 2 h, and washed 3 × 10 min in distilled water. Next, samples were dehydrated with a graded ethanol series: 30%, 50%, 70%, 80%, 90%, 96%, and 100%, and a drop of the suspension was applied to a coverslip and evaporated at room temperature. Samples were glued to the aluminum table covered with carbon foil and sprayed with a thin layer of pure gold using the POLARON SC7620 ion sputtering machine from Microtech. Observations were performed using a scanning electron microscope Merlin FE-SEM/EDS by Zeiss/Bruker at the Faculty of Measurement Laboratory, Faculty of Chemistry, University of Warsaw.

#### Transmission electron microscopy (TEM)

The material was fixed for 24 h in 2.5% glutaraldehyde in 0.1 M cacodylate buffer pH 7.2. It was then postfixed in 1% OsO_4_ (osmium tetroxide) in bidistilled water for 1 h. Then, the samples were dehydrated through an increasing gradient of ethanol concentrations (50% − 10 min, 70% − 24 h, 90% − 10 min, 96% − 10 min, anhydrous EtOH − 10 min, and acetone − 10 min). Then, the material was saturated with epoxy resin, starting with mixtures with acetone of increasing concentration (1:3–30 min, 1:1–2 h, 3:1–3 h) up to pure epoxy and the material was left in it for 12 h. After supersaturation, it was polymerized in blocks for 24 h at 60ºC. The polymerized samples were then cut into ultrathin Sect. (70 nm) using an RMC MTX ultramicrotome, applied to copper grids, contrasted with a saturated solution of uranyl acetate and Reynolds reagent [70], and analyzed in a transmission electron microscope. The photos were taken in the Laboratory of Electron Microscopy at the Nencki Institute of Experimental Biology of the Polish Academy of Sciences in Warsaw using a JEM 1400 transmission electron microscope (JEOL Co., Japan), equipped with an X-ray microanalyzer (EDS INCA Energy TEM, Oxford Instruments, Great Britain), a tomography system and a MORADA G2 CCD camera (EMSIS GmbH, Germany), purchased from the EU structural funds as part of the CZT BIM project - Equipment of the Laboratory of Biological and Medical Imaging.

### LC-MS/MS analysis of amino acids (AA)

LC-MS/MS analysis of L-ornithine and L-citrulline was conducted based on methods published by [71–73], with a few modifications in the Department of Analytical Methods Development of Olimp Laboratories. Briefly, all analyses were performed on ultra-high performance liquid chromatography apparatus Shimadzu Nexera X2 coupled with LCMS-8040 mass spectrometer (Shimadzu Corporation, Kyoto, Japan). Mobile phases consisted of Solution A (acetonitrile/tetrahydrofuran/25 mM ammonium formate/formic acid, - v:v:v:v 90:750:160:3) and Solution B (acetonitrile/100 mM ammonium formate, v:v − 200:800). Separation has been performed on Intrada Amino Acid column (100 × 3 mm; 3 μm; Imtakt, Portland, OR, USA) with optimized gradient for individual amino acids (AAs) (0–9 min, 0% Solution B, 9–16 min, 17% Solution B, 16–19 min, 100% Solution B, 19–23 min, 0% Solution B) with a total run time of 23 min. Retention times: citrulline: 10.18 min.; Ornithine: 17.25 min. Samples were ionized by electrospraying (ESI) and the ion source was operated in positive mode and desired conditions (auxiliary gas flow: 15.0 l/min; nebulizing gas flow: 3.0 l/min, temperature of desolvation line: 300ºC). Optimal multiple-reaction monitoring (MRM) transitions were collected in the table below.

**Table:** 

Amino acid	MRM [m/z]	CE (V)
L-citrulline	176.20 > 159.05	− 17.0
L-ornithine	133.10 > 70.20	− 23.0

Standards of L-citrulline (Sigma-Aldrich, cat. no. C7629), and L-ornithine HCL (Sigma-Aldrich, cat. no. 75,469) were weighed accurately into volumetric flasks using an analytical microbalance (Sartorius, Germany), dissolved in 0.1 M HCl to produce individual AA stock solutions, and then serially diluted in deionized water to prepare solutions for the calibration curve (25 ng/ml-1 µg/ml).

CFSs of tested strains were 100-fold diluted in 0.1 M HCl, sonicated, then diluted 1000 times by deionized water to fit into the calibration curve, and sterile-filtered through a syringe filter (0.22 μm). 5 µl was injected for LC-MS/MS analysis.

Data were acquired and analyzed using LabSolutions software.

### Statistical analysis

Statistical analysis was done using a one-sided t-test unless otherwise stated.

## Results

### Cell-free supernatant and > 50 kDa supernatant fraction present cytostatic activity

**Fig. 1:**
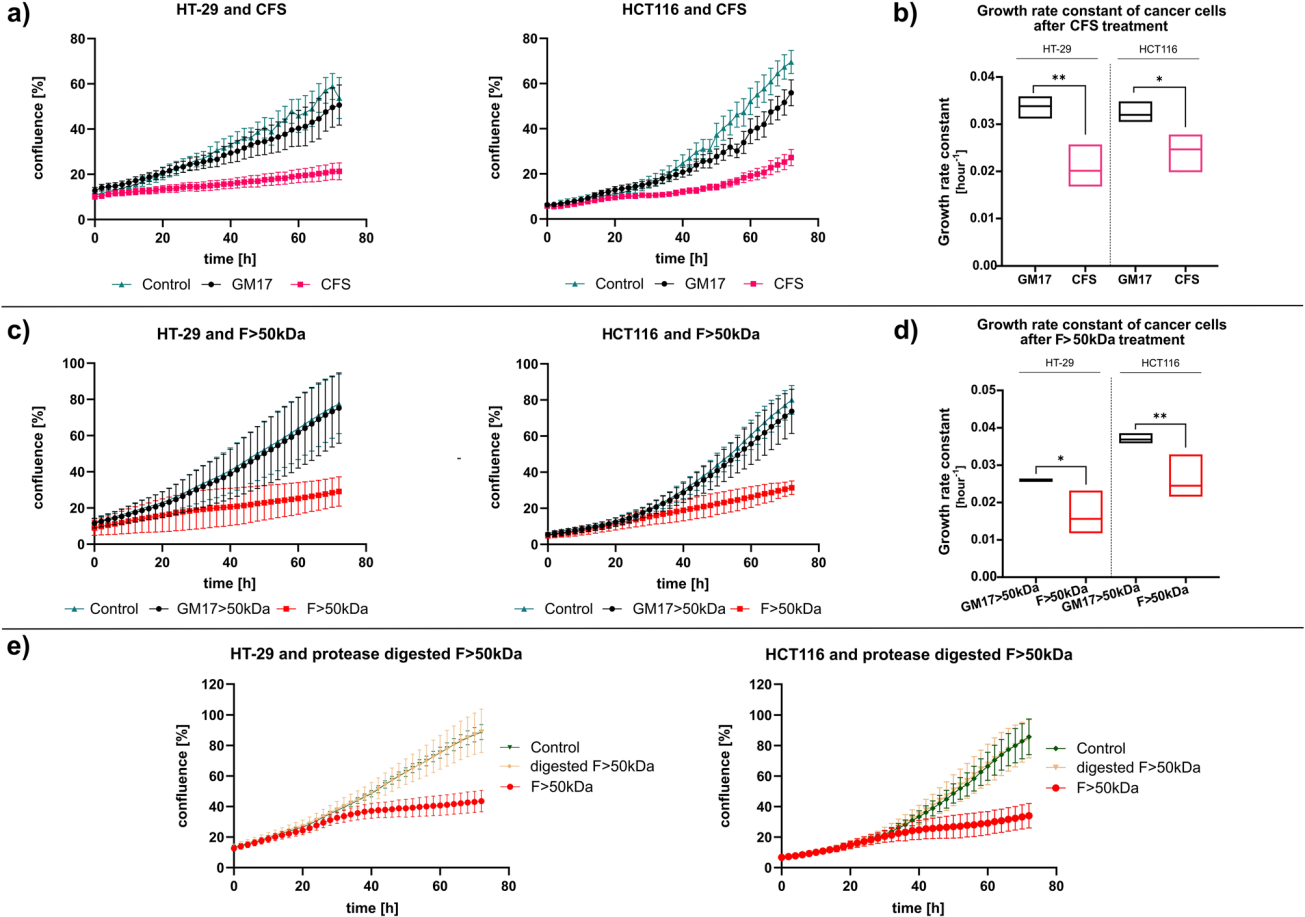
Both CFS and > 50 kDa fraction from *L. lactis* Lc4 strain demonstrate a protein-dependent reduction in cell proliferation. **a, c**) Confluence of HT-29 (left) and HCT116 (right) in time after treatment with 33% (v/v) CFS (**a**) or F > 50 kDa (**c**). GM17 or GM17 > 50 kDa was used as additional control. Graphs represent one biological repetition, performed in 3 technical replicates, error bars represent SD. **b**, **d**) Cell growth rate constant after treatment with CFS (**b**) or F > 50 kDa (**d**) (*n* = 3 independent experiments). Data are presented as mean with min to max range. **e**) Effect of protein digestion on the cytostatic activity of F > 50 kDa fraction. Results represent mean +/- SD for *n* = 2 independent experiments (*p < 0.05, **p < 0.01, by one-sided t-test)

Our preliminary screen was performed using the MTS assay to evaluate the influence on cell metabolism of cell-free supernatants (CFSs) derived from three *Lactococcus lactis* ssp. *lactis* strains, grown under conditions simulating the temperature and oxygen availability of the human gut. The experiments revealed that the *L. lactis* ssp. *lactis* Lc4 strain exhibited the strongest impact on the HT-29 cell line (Supplementary Fig. 1a). We further examined the effect of *L. lactis* Lc4 CFS on the growth of two colorectal cancer cell lines, HT-29 and HCT116, using the IncuCyte S3 live-cell analysis system that enables real-time, continuous monitoring of cell fitness. A substantial reduction of the growth rate of HT-29 and HCT116 cell lines, along with a 40% reduction in confluence compared to the control (GM17 medium), was observed (Fig. 1a, b). To identify the underlying factors responsible for this effect, the *L. lactis* Lc4 CFS was fractionated using defined molecular weight cut-off filters. Only the > 50 kDa fraction of CFS (F > 50 kDa) demonstrated a clear anti-proliferative (cytostatic) phenotype compared to the control GM17 medium fraction > 50 kDa (Fig. 1c, d). The remaining fractions did not significantly affect the growth of the cells as compared to the respective control (Supplementary Fig. 1b, c).

The results suggested that the cytostatic effect could be attributed to compounds present in the F > 50 kDa fraction, such as proteins, polysaccharides, nucleic acids, or cell wall derivatives. Additionally, the F > 50 kDa fraction displayed a high protein content (~500 µg/ml; Bradford measurement [58]). To investigate whether proteins are responsible for the observed cytostatic properties, the F > 50 kDa fraction was treated with proteases (trypsin and chymotrypsin). As a result, a complete loss in cytostatic activity of the F > 50 kDa fraction in both HT-29 and HTC116 cell lines was noted (Fig. 1e). These findings strongly suggested that the active compound(s) present in the F > 50 kDa are likely to be proteins or other heterogeneous molecule(s) containing peptide bonds, such as peptidoglycans.

### Identification of proteins in biologically active fractions

To thoroughly characterize the protein content of the F > 50 kDa fraction, proteomic analyses were performed, resulting in the identification of 654 proteins (Supplementary Table 1). To identify the active protein components responsible for the observed cytostatic effect, we employed various protein purification techniques, including size exclusion chromatography on Superdex 75 (GF75) and Superdex 200 (GF200) columns, as well as ion exchange chromatography (IEX) (see Materials and Methods). The objective was to retain the observed cytostatic phenotype while isolating the active elements. Using these separation techniques, we successfully obtained fractions with comparable cytostatic activity, as depicted in Supplementary Figure S2a,b,c.

**Fig. 2:**
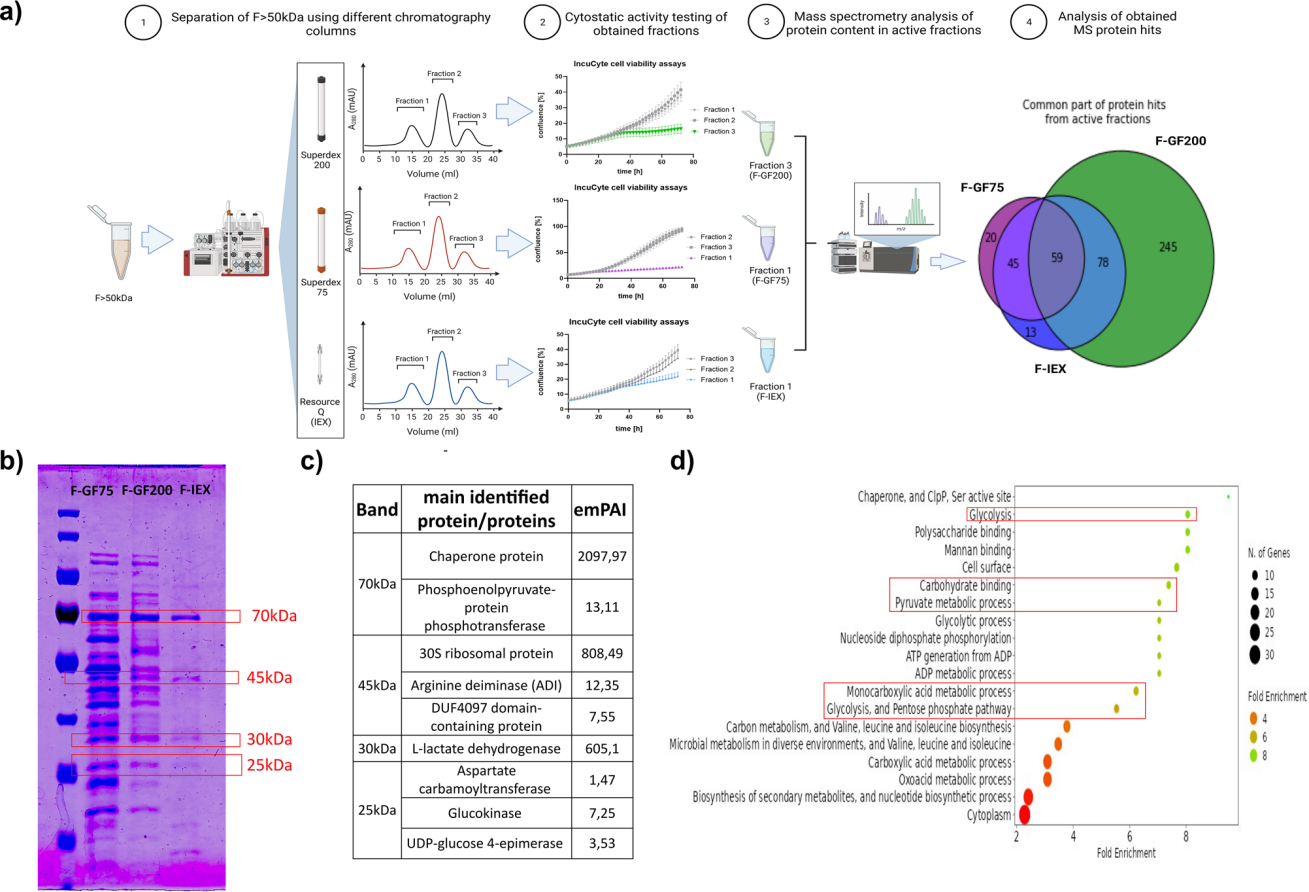
Identification of proteins in fractions responsible for the observed cytostatic effect. (**a**) Diagram depicting the experimental set-up of identifying potentially active proteins in the F > 50 kDa fraction. (**b**) SDS-PAGE analysis of active fractions separated via different chromatography techniques. Bands excised for MS analysis are marked with red rectangles, with their corresponding molecular weight provided. (**c**) Proteins identified by MS within the excised SDS-PAGE bands, along with their respective emPAI index. (**d**) Gene Ontology biological process enrichment analysis performed with the ShinyGO package [59]. Visualized are the top 20 significantly enriched pathways. Pathways associated with glucose degradation and its metabolic processes are marked in red rectangles. Details of the analysis are summarized in the Materials and Methods section, and full results of GO enrichment analysis are presented in Supplementary Table 2. Analysis parameters: FDR cut-off: 0.05, pathway size: Min. 10, Max. 2000; removed redundancy, and abbreviated pathways. Fold Enrichment was defined as the percentage of genes above the FDR cut-off, belonging to a pathway, divided by the corresponding percentage in the background (MS hits in F > 50 kDa).

A comparative proteomics approach was employed by analyzing MS protein hits from active fractions obtained through GF75, GF200, and IEX separation methods to identify proteins common to all active fractions (Fig. 2a). As a result, the number of hits was reduced to 59 proteins. Moreover, an SDS-PAGE-based comparison of protein profiles between the samples from the different purification methods allowed for the selection of four bands that were consistently visible in all active fractions (Fig. 2b). Subsequent MS analysis of these excised bands led to the identification of 13 proteins, including arginine deiminase (ADI) (Fig. 2b, c - band 45 kDa), chaperone protein DnaK (Fig. 2b, c - band 70 kDa), lactate dehydrogenase (Fig. 2b, c - band 35 kDa), phosphoenolpyruvate-protein phosphotransferase (Fig. 2b, c - band 70 kDa), and UDP-glucose 4-epimerase with glucokinase (Fig. 2b, c in band 25 kDa). The complete list of identified proteins from the MS analysis can be found in Supplementary Table 1.

Gene Ontology (GO) term enrichment analysis performed on the common subset of 59 proteins revealed enrichment in chaperones and proteins associated with protein folding. These findings may be attributed to the prolonged cultivation period of the Lc4 strain and stress response to increased temperature and low oxygen availability [74–76]. Additionally, several proteins associated with metabolic pathways related to energy production (e.g. glycolysis, carbohydrate binding, conversion of ADP to ATP) were enriched (Fig. 2d). The GO term and STRING (Search Tool for the Retrieval of Interacting Genes/Proteins) [77] analyses also revealed the presence of all proteins required for the conversion of glucose to lactate (glycolytic pathway), see supplementary material Figure S3.

### Active fractions cause starvation and cell cycle arrest

**Fig. 3:**
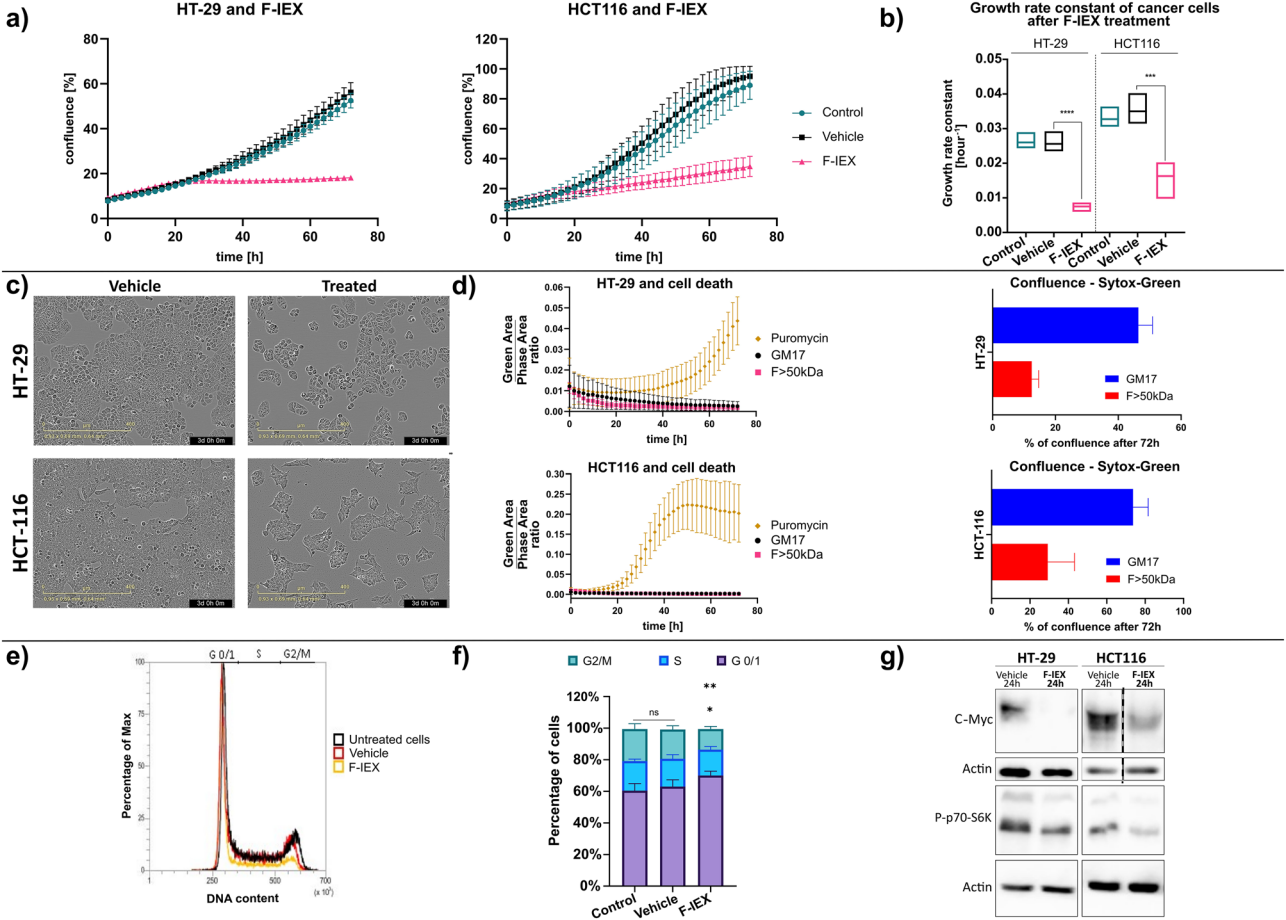
F-IEX fraction induces cell cycle arrest in the G0/1 phase, decreases c-Myc expression, and reduces phosphorylation of the p70 S6 kinase. (**a**) Confluence changes in HT-29 and HCT116 cells over time (0-72 h) after F-IEX (5% v/v) and vehicle (0.35 M NaCl + 10 mM HEPES buffer – 5% v/v) or no treatment (control). Scans were performed every 2 h, each time point represents mean +/- SD (*n* = 3 independent experiments). (**b**) Growth rate constant of F-IEX-treated HT-29 and HCT116 cells. (**c**) Microscopic images from the IncuCyte S3 Live-Cell imaging system of HT-29 and HCT116 cells treated for 72 h with F-IEX (5% v/v) or vehicle (same as in a) ) as a control. (**d**) SYTOX Green-exclusion experiments. Green area-to-phase area ratio as quantification of changes in cells with disrupted membrane integrity during 72 h treatment with F > 50 kDa and GM17 > 50 kDa (5% v/v) and 5 µM of SYTOX Green (left graph) with corresponding confluence (right graph). Puromycin (0.5 µg/ml) was used as a cell death-inducing positive control. (**e**) Cell cycle analysis of HT-29 cells after 24 h treatment with 5% (v/v) F-IEX and vehicle (same as in a). (**f**) Bar-chart quantifying result from panel e. Data presented as mean +/- SD from *n* = 3 independent experiments. (**g**) Western blots showing c-Myc and phosphorylated p70-S6 kinase (P-p70-S6K) levels. HT-29 and HCT116 cells were treated with 5% vehicle (same as in a) or 5% (v/v) of F-IEX for 24 h. Total protein was isolated from the cells, resolved in SDS-PAGE gels, and analyzed using antibodies against indicated proteins. Actin was used as a loading control. *n* = 3 independent experiments. (*p < 0.05, **p < 0.01,***p < 0.001, ****p < 0.0001 from one-sided t-test)

Pro- and postbiotics often exert their inhibitory activity toward cancer cells through the involvement of pro-apoptotic factors [28, 78]. In our study, we observed reduced proliferation of cells treated with pooled semi-purified ion exchange chromatography fractions (F-IEX) (Fig. 3a, b). Additionally, we noticed changes in cell morphology, e.g. sharper edges of HCT116 cells (Fig. 3c). However, no signs of cell death were visible, as assessed by SYTOX green-exclusion experiments (Fig. 3d). Based on these observations, we hypothesized that the active compound(s) could induce cell cycle arrest. The results of proteomic analyses suggested the presence of certain proteins in the active fractions, such as arginine deiminase or glucose-degrading enzymes, including lactate dehydrogenase. These enzymes have the ability to metabolize nutrients leading to cancer cell starvation. We hypothesized this could potentially occur through induction of cell cycle arrest in the G0/G1 phase, which could lead to the observed growth inhibition.

To verify this hypothesis, we conducted cell cycle assays, which showed a reduction of cell number in the G2/M phase (*p* < 0.01) and an increased number of cells in G0/G1 (*p* < 0.05) (Fig. 3e, f). Moreover, western blot analysis revealed a decreased level of the c-Myc protein, a key cell cycle regulator [79, 80], and a reduction in phosphorylation of nutrient-sensitive p70 S6 kinase (*p* < 0.05) in both HT-29 and HCT116 cell lines (Fig. 3g). The p70 S6K is an effector kinase of the mTOR complex 1 (mTORC1), known to be sensitive to the depletion of glucose and certain amino acids, including arginine [81–83]. Taken together, the obtained results strongly suggested that treatment with F-IEX fraction leads to starvation of cancer cells and that nutrient deprivation may be an important cause of the observed growth reduction.

### The cytostatic effect of the F-IEX fractions is not related to glucose-degrading enzymes

Proteomic analysis of the F-IEX revealed the presence of all glycolytic pathway enzymes, necessary to convert glucose to lactate. High activity of glycolytic enzymes would typically result in decreased glucose levels, increased L-lactate levels, and potentially induce cell cycle arrest leading to a reduction of cell proliferation as a result of nutrient depletion. Additionally, L-lactate dehydrogenase, the ultimate enzyme in the glycolytic pathway, which catalyzes the reversible conversion of pyruvate to L-lactate, could deplete its substrate present in cell culture media. Pyruvate is an additional carbon source, which improves the growth of cell lines in vitro [84, 85]. Pyruvate depletion may lead to faster glucose consumption and finally to reduced growth. However, the cells treated with an active F-IEX fraction metabolized glucose slower than the control groups (Supplementary Figure S4a). Additionally, F-IEX- and F > 50 kDa-treated cells produced less lactate compared to respective controls (SupplementaryFigure S4b). Finally, the addition of 3 mM of sodium pyruvate to F-IEX-treated cells did not improve the growth rate of cells (Supplementary Figure S4c). Collectively, these findings strongly suggest that the observed effects are not directly mediated by glucose-metabolizing enzymes.

### Arginine deiminase is responsible for the cytostatic effects of tested fractions

**Fig. 4:**
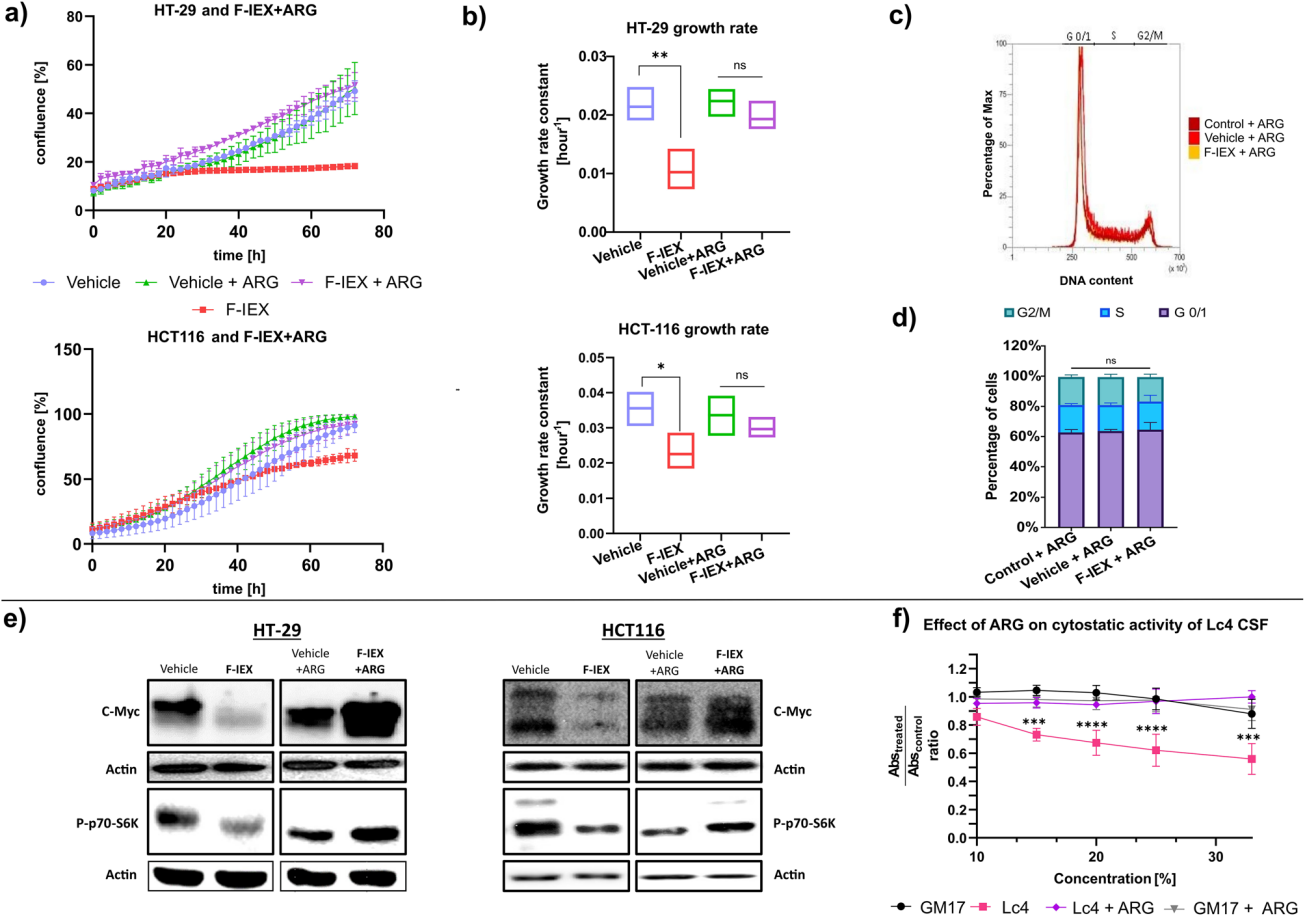
Arginine supplementation rescues the cytostatic effect of F-IEX and *L. lactis* Lc4 CFS. (**a**) Confluence changes of HT-29 (top) and HCT116 (bottom) cells over time (0-72 h) after F-IEX (5% v/v) and vehicle (0.35 M NaCl + 10mM HEPES buffer – 5% v/v) treatment, with and without the addition of 5 mM L-arginine (ARG). Cell proliferation was monitored with the IncuCyte S3 live-cell analysis system. Scans were performed every 2 h. Each time point represents mean +/- SD, *n* = 3. (**b**) Growth rate constant of data presented in panel a. (**c**) Cell cycle analysis of HT-29 cells after 24 h of treatment with 5% (v/v) F-IEX and vehicle (same as in a) with the addition of 5mM L-arginine (ARG). Measured DNA content corresponds to the percentage of cells in each cell cycle phase (G0/1, S, G2/M). (**d**) Bar chart quantifying results from panel c. Data presented as mean +/- SD from *n* = 3 independent experiments. (**e**) Western blots showing c-Myc and phosphorylated p70 S6 kinase (P-p70-S6K) levels. HT-29 and HCT116 cells were treated with 5% vehicle (same as in a) or 5% of F-IEX for 24 h, with and without the addition of 5mM L-arginine. Total protein was isolated from the cells, resolved in SDS-PAGE gels, and analyzed using antibodies against indicated proteins. Actin was used as a loading control. *n* = 3. (**f**) The effect of 5 mM L-arginine addition on the cytostatic activity of Lc4 CFS evaluated on HT-29 cells with the MTS assay (*p < 0.05, **p < 0.01,***p < 0.001, ****p < 0.0001 from one-sided t-test (b, d) or one-way ANOVA with Bonferroni correction (**f**)

As noted above, proteomics and bioinformatics analyses revealed the presence of arginine deiminase (ADI) in the F > 50 kDa fraction, which was also detected in fractions obtained through GF75, GF200, and IEX purification methods. ADI converts L-arginine (ARG) to L-citrulline and ammonia. It was reported that both HT-29 and HTC116 cell lines are sensitive to arginine deprivation [86]. To investigate whether the observed phenotype following treatment with F-IEX is due to reduced arginine levels in the medium, the antiproliferative activity of F-IEX was examined in the presence or absence of arginine. The addition of arginine entirely abolished the cytostatic activity of the tested fraction; in cells treated with F-IEX + ARG, neither inhibition of proliferation (Fig. 4a, b) nor cell cycle arrest (Fig. 4c, d) was observed. Moreover, c-Myc protein levels and p70 S6K phosphorylation were not reduced (Fig. 4e). Additionally, we observed cytostatic phenotype only for those fractions from IEX purification, where ADI enzymatic activity was present (Supplementary Figure S5).

**Fig. 5:**
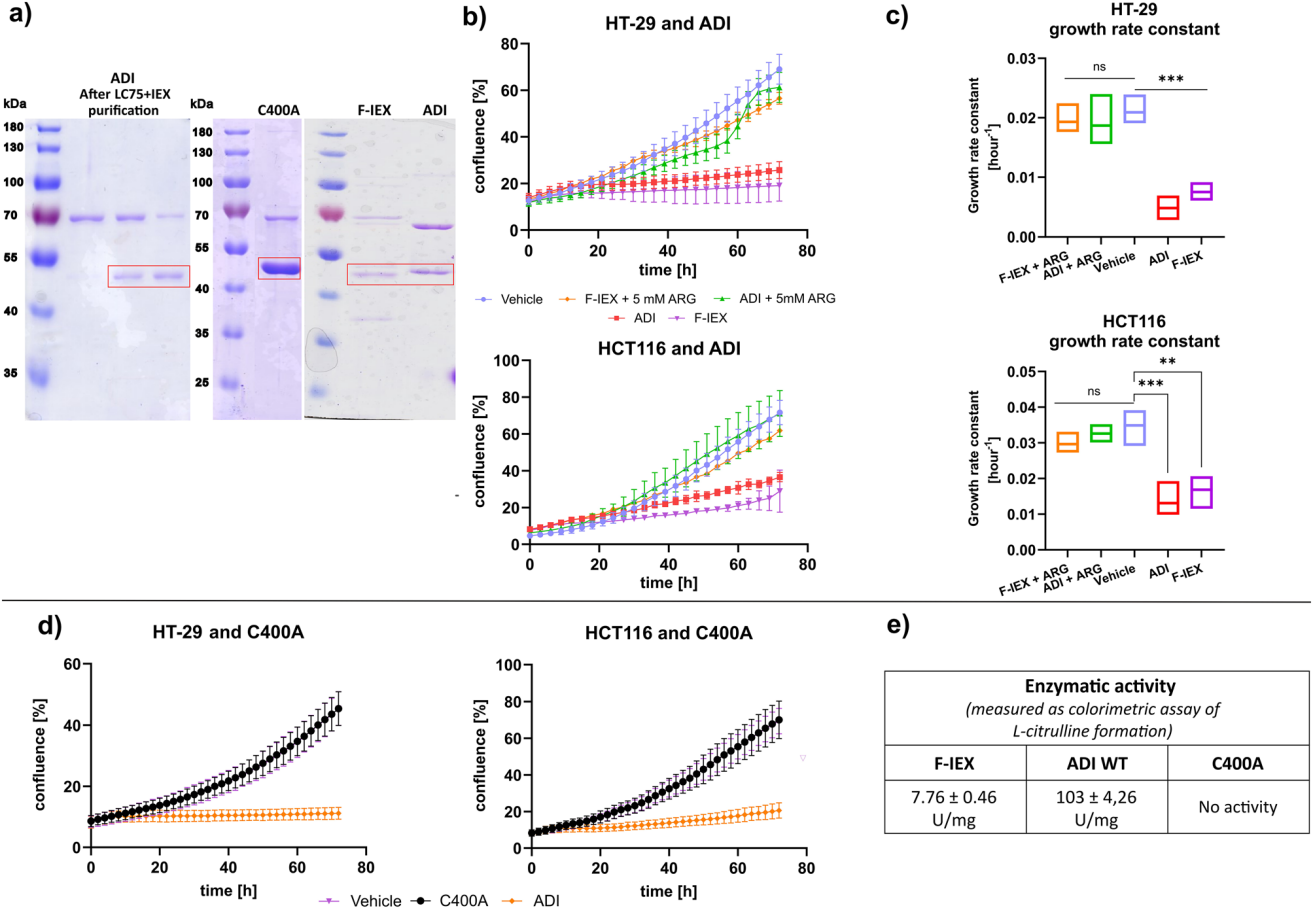
Heterologously expressed recombinant wild-type ADI protein exhibited the same cytostatic phenotype as F-IEX. (**a**) SDS-PAGE gels analysis of purified proteins. Left gel: ADI WT protein after two steps of purification on Superdex75 (GF75) and Resource Q (IEX) columns (three consecutive fractions collected after IEX separation are shown). Middle gel: Catalytically inert mutant of ADI – C400A. Right gel: Direct comparison of F-IEX and heterologously expressed recombinant ADI WT protein. The red rectangles show the bands corresponding to the calculated size of respective ADI variants (ADI WT or C400A). (**b**) Representative graphs of confluence changes of HT-29 (top) and HCT-116 (bottom) cells over time (0-72 h) after ADI (0.66% v/v), F-IEX (5% v/v) and vehicle (0.35 M NaCl + 10mM HEPES buffer) treatment with or without the addition of 5 mM L-arginine (ARG). The proliferation of cells was monitored using the IncuCyte S3 live-cell analysis system. Scans were performed every 2 h. Each time point presents a mean +/- SD. (**c**) Growth rate constant of data presented in b) (*n* = 3 independent experiments). (**d**) Confluence changes of HT-29 (left) and HCT116 (right) cells over time (0-72 h) after ADI (0.66% v/v), vehicle (same as in b) and C400A catalytic mutant (0.66% v/v) treatment. The proliferation of cells was monitored using the IncuCyte S3 live-cell analysis system. Scans were performed every 2 h. Each time point presents a mean +/- SD. (*n* = 3 independent experiments). (**e**) Table with enzymatic activity values of F-IEX, heterologously expressed recombinant ADI WT protein, and ADI C400A catalytic mutant (*p < 0.05, **p < 0.01,***p < 0.001, ****p < 0.0001 from one-sided t-test)

The results presented above strongly indicate that the cytostatic activity of both F > 50 kDa and F-IEX fractions, as well as the full CFS, can be attributed to arginine deiminase enzymatic activity. The ADI protein contains two domains: a catalytic domain (fan) and a five-helix bundle domain (clip) [87, 88]. The catalytic triad of amino acids (Cys-His-Asp/Glu) is responsible for nucleophilic attack and arginine-citrulline transformation. To confirm the role of ADI in the observed phenotype, heterologous expression of the *L. lactis* arginine deiminase (*arcA*) gene in *E. coli* BL21-RIL strain was performed, allowing for successful purification of recombinant wild-type ADI (ADI WT) and its catalytically inactive variant with C400A amino acid substitution (ADI C400A), as previously described [89]. Both protein variants had a molecular mass of around 48 kDa (46 kDa monomer + 2 kDa HisTag) (Fig. 5a, Supplementary material Figure S6). The obtained ADI WT exerted the same cytostatic phenotype in cells as the F-IEX fraction. The concurrent ARG addition to both HT-29 and HCT116 cell lines reversed the observed cytostatic effect of ADI WT (Fig. 5b, c). In contrast, the ADI C400A mutant protein did not display the cytostatic phenotype, consistent with the lack of enzymatic activity, as compared to ADI WT and the F-IEX fraction (Fig. 5d, e).

### The presence of arginine deiminase in the supernatant is a unique feature of the Lc4 strain

To date, no evidence has been provided that would support the secretion of ADI or its appearance as an extracellular enzyme. The abundant protein profile of the F > 50 kDa indicated the presence of intracellular proteins in the CFS. GO term analysis further confirmed that the majority of proteins present in the F > 50 kDa originated from the cytoplasm. Additionally, a similar protein pattern for F > 50 kDa and cell lysate was observed in the SDS-PAGE gel (Supplementary Fig. 7a-c).

**Fig. 6:**
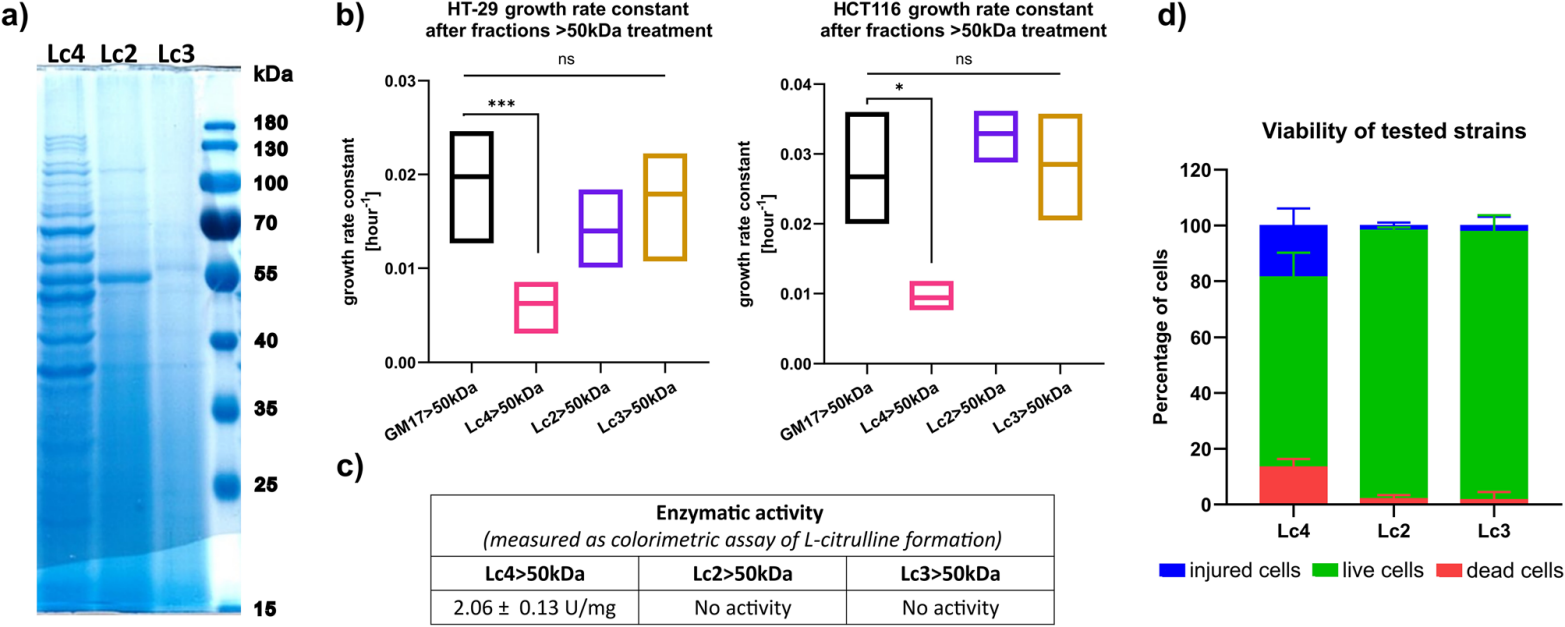
The enzymatic and cytostatic activity of the F > 50 kDa fraction is a unique feature of the Lc4 strain. (**a**) SDS-PAGE gel stained with PageBlue (Thermo Fisher) with protein profiles of F > 50 kDa fractions from Lc4, Lc2, and Lc3 *L. lactis* strains. (**b**) Growth rate constant of HT-29 (left) and HCT116 (right) cells treated with F > 50 kDa fractions from Lc4, Lc2, Lc3, and GM17 > 50 kDa (as a control) (*n* = 3 independent biological replicates). Data presented as a mean with min to max range. (**c**) Enzymatic activity of F > 50 kDa fractions from Lc4, Lc2, and Lc3 strains. Data presented as mean +/- SD from *n* = 3. (**d**) Stacked bar chart presenting the percentage of injured, dead, and live bacterial cells (cultivation condition described in the Materials and Methods section). Data are presented as a mean from *n* = 3 biological replicates (*p < 0.05, **p < 0.01,***p < 0.001, ****p < 0.0001 from one-way ANOVA with Dunnet’s correction)

### ADI release is related to protein leakage and cell lysis of Lc4

Based on the data presented above, we hypothesized that the presence of cytoplasmic proteins in the extracellular space could be due to the controlled leakage and/or cell lysis as a result of the cultivation of the Lc4 strain under stress conditions. All *Lactococcus* strains were cultivated in temperature and oxygen-limiting conditions mimicking those occurring in the human gastrointestinal tract (GI) (37 °C, anaerobic conditions, 24 h of cultivation time - which simulates one type of average gut transit time [90]). These conditions are regarded as stressful for lactococci, which are mesophilic bacteria with optimum temperature growth of around 30 °C and a high growth rate in rich nutritional media [74, 91]. To verify this assumption, the viability of the Lc4 strain was examined, changes in the protein profile over the growth time course were monitored, and cell morphology was assessed in scanning and transmission electron microscopy (SEM/TEM). Additionally, we verified the survivability and ADI enzymatic activity of the Lc4 strain using a growth medium (Feed) designed for a simulator of the human intestine microbial environment (SHIME®; ProDigest) with and without the pancreatic juice (PJ) (see Materials and Methods section), to check how simulated GI conditions (nutritional availability, presence of bile acids and digestive enzymes) may affect Lc4 fitness.

**Fig. 7:**
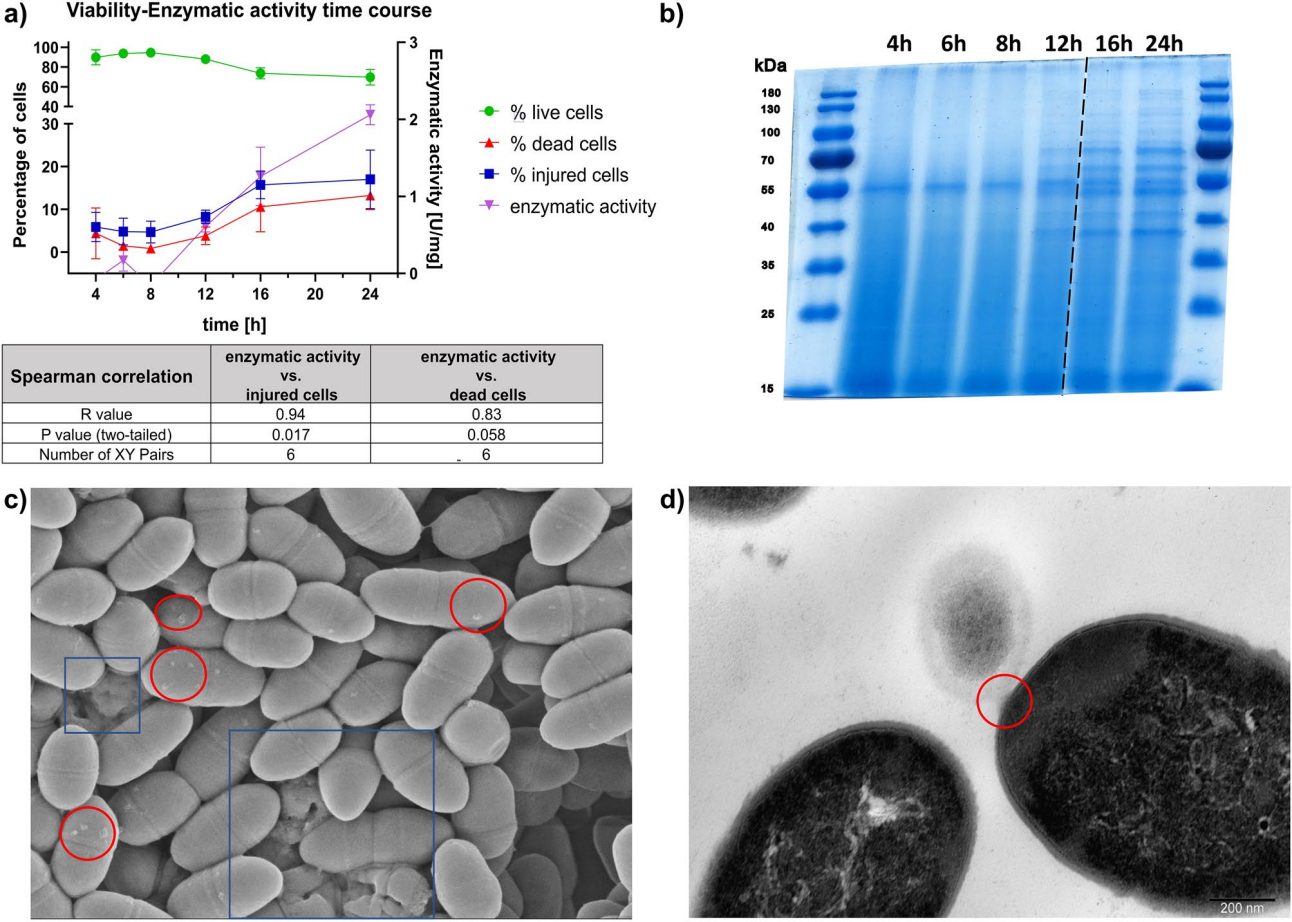
ADI release is the consequence of bacterial cell damage and protein leakage. (**a**) Changes in the viability of *L. lactis* Lc4 cells during cultivation with respect to ADI enzymatic activity. The enzymatic activity, as an amount of L-citrulline produced by ADI, was measured for F > 50 kDa obtained from 25 ml of CFS. Data points are represented as mean +/- SD (*n* = 3 independent biological replicates). (**b**) SDS-PAGE gel analysis of protein profiles of Lc4 F > 50 kDa obtained from different bacterial cultivation time points. Protein concentration was measured with the Bradford method and equal protein amounts of each sample were resolved in SDS-PAGE gel. (**c**) SEM image of Lc4 strain cells after 24 h of cultivation. Blue rectangles indicate cells undergoing the lysis process. Red ovals mark possible cytoplasmic blebs and/or leakage events. (**d**) TEM image of cytoplasm leakage from Lc4 cells. The red oval indicates altered membrane integrity

Time course experiments revealed a positive correlation between the increase in the number of dead and damaged cells during the cultivation period and the ADI enzymatic activity (Fig. 7a). After the initial 8 h of cultivation, no changes in the enzymatic activity of ADI and the protein profile were detected (Fig. 7a, b). However, after 12 h of cultivation, the protein profile started to change. More specifically, bands around 55 kDa became visible and their intensity increased over time, which was accompanied by an increased number of damaged and dead cells. The total reduction of viable cell number between 8 and 12 h of cultivation was around 6%, whereas it reached approximately 25% between 8 and 24 h. Furthermore, a comparable decline of viability between GM17 and Feed or Feed + PJ was observed. Lc4 retains its ability to convert L-arginine to L-citrulline, which suggests that ADI was active in simulated GI conditions (Supplementary Fig. 8).

To confirm the role of disrupted membrane integrity in the ADI release, SEM and TEM analysis of the Lc4 strain was conducted. Scanning electron microscopy of cells cultivated for 24 h revealed several cells undergoing lysis. Interestingly, multiple extracellular bodies attached to the cell envelope were visualized (Fig. 7c), which could potentially represent extracellular vesicles or leaked cytoplasmic contents. However, in TEM images, classical nanovesicle formation was not apparent; instead, cytoplasm blebs and free cytoplasmic bodies between cells were visible (Fig. 7d). Analysis of the images revealed that this feature is commonly present, suggesting it may be a supporting mechanism accompanying cell lysis.

Taken together, these results suggested that the presence of an enzymatically active ADI in CFS is strain-dependent and associated with the ability to adapt to stress conditions.

## Discussion

Cancer starvation emerged as a therapeutic strategy with the potential to complement classical anti-cancer treatments [92–95]. Multiple solid tumors, including CRC, exhibit auxotrophy to arginine [92]. A key factor contributing to tumor vulnerability to arginine depletion is the expression of the *ASS1* (argininosuccinate synthetase 1) gene. Numerous cancer cell lines and tumors of different cancer types have shown reduced levels of ASS1 protein [96–100]. Diminished *ASS1* expression, leading to arginine auxotrophy, unfortunately, correlates with increased drug resistance and an overall poor prognosis of therapy [101]. Despite varying levels of *ASS1* expression among patients [102] and cell lines [86, 102, 103], arginine depletion has been recognized as a therapeutic opportunity for different cancer types [104]. Nevertheless, the debate surrounding the *ASS1*-negative phenotype in colorectal cancer persists, with some studies indicating that *ASS1* overexpression plays a pivotal role in carcinogenesis, metastasis, and disease progression in CRC [105, 106]. Conversely, data from the Human Protein Atlas reveal that an *ASS1*-low expression phenotype occurs in 20% of patients, which is negatively correlated with five-year survival rates and indicative of poor prognosis, especially in men [102].

ADI has previously shown promise as an anticancer agent. Its PEGylated form derived from *Mycoplasma hominis* (ADI-PEG20) has reached the phase III of clinical trials for *ASS1*-negative cancers [107, 108], however, concerns about efficacy and immunogenicity have been raised. Reduced efficacy was primarily associated with the re-expression of the *ASS1* gene, leading to resistance during cancer therapy. This resistance mechanism involves c-Myc binding directly to the *ASS1* gene promoter region and inducing its expression [109].

In our study, ADI derived from *L. lactis* ssp. *lactis* Lc4 CFS led to a substantial reduction in c-Myc protein levels in both HT-29 and HCT116 cancer cell lines. To our best knowledge, there is only one study published where ADI originating from *L. lactis* was used, showing similarly reduced levels of c-Myc in SNU-1 stomach adenocarcinoma cells [110]. Interestingly, the reduced levels of c-Myc protein were not observed [86] when ADI-PEG20 was used [86]. This could be attributed to differences in enzymatic activity between ADI-PEG20 and *L. lactis* ADI, various posttranslational modifications, or differences in experimental procedures. Notably, we observed higher specific enzymatic activity of the purified recombinant ADI from Lc4, compared to *M. hominis* ADI, both at pH 6.4 (103 U/mg vs. 42 U/mg) and at physiological pH 7.4 (16.5 U/mg vs. 3.9 U/mg) in 37℃ [111, 112]. These findings hold promise for *L. lactis* ADI as an alternative therapeutic lead.

Arginine deprivation may be beneficial for cancers with the presence of *ASS1*- and argininosuccinate lyase (*ASL*) expression. This feature was previously reported for the HCT116 colorectal cancer cell line, which did not exhibit an *ASS1*-negative phenotype [86]. HCT116 cells are instead sensitive to arginine deprivation due to activation of the unfolded protein response (UPR) and inhibition of mTORC1 signaling. In our study, a substantial reduction of HCT116 cell proliferation was also observed, with an accompanying reduction of phosphorylation of p70 S6 kinase, which is the main effector kinase of mTORC1. This phenotype was observed earlier (after 24 h of treatment) when compared to [86], and similar to [113], which also reported positive cytostatic effects of ADI-PEG20. However, it is essential to note that the HCT116 cell line is considered to have a low level of ASS1 enzyme, and conducting detailed comparisons between *L. lactis* ADI and ADI-PEG20 on other cell lines exhibiting higher *ASS1* expression levels poses a topic for future investigation.

Another aspect that influences the efficacy and safety profile of ADI-PEG20 is its immunogenic character. Nonmodified ADI protein is highly immunogenic due to its pathogenic origin. Although the PEGylation process effectively reduced immunogenicity, this problem still persists [114], leading to reduced half-life and therapy effectiveness (faster clearance). Hence the need for the development of more effective, less immunogenic molecules [115]. Based on the different characteristics of the source bacteria (*M. hominis* - pathogen versus *L. lactis* - commensal bacteria), we suggest ADI originating from *L. lactis* as an interesting alternative to ADI-PEG20 for future development.

Our results show that the *L. lactis* Lc4 strain has a unique ability to release ADI to the supernatant, in stress-simulating conditions, as compared to Lc2 and Lc3 strains. The ADI pathway in bacteria consists of three enzymes, which catalyze the conversion of L-arginine to L-citrulline (ADI) and L-citrulline to L-ornithine (ornithine transcarbamylase - OTC) and carbamate kinase - which produces ATP [116, 117]. The ADI pathway is responsible for ammonia and energy production and has multiple functions, such as maintaining cellular pH, adapting to low pH environments [118], influencing biofilm formation [119], and overall adaptation to harsh conditions [116] and may not be expressed or active in all lactococci [118, 120]. LC/MS analysis of amino acid content in CFSs (Supplementary Fig. 9) revealed that the ADI-dependent pathway is fully functional in all tested strains. In the Lc4 strain, the ADI release is not likely connected to different regulation and expression of these proteins under stress conditions [121–124].

Additionally, *L. lactis* Lc4 strain viability analysis revealed a substantial (around 30%) drop of live cells after 24 h in stress-simulating conditions (Supplementary Fig. 10b) possibly caused by a combination of cell injury (~21%) and cell death (~10%). Bacterial cells undergoing lysis were also observed with SEM images, suggesting that the cell membrane integrity was altered. The possible trigger of this change could be related to cytoplasmic blebs, observed in SEM/TEM images. Staining with thiazole orange (TO) + PI has limitations and may create false positive results [125], e.g. when tested bacteria have the ability to release DNA into extracellular space [126], which could be present in the observed cytoplasmic blebs.

Based on those findings, we hypothesized that both mechanisms, i.e. autolysis and the ability to release cytoplasmic content, are jointly responsible for the strain-dependent cytostatic activity of Lc4 CFS (Fig. 7). Autolysis is the desired functionality of *L. lactis* strains, which are common bacteria in starter cultures during cheese ripening. SEM and TEM images of LAB cells from the cheese ripening process show morphological changes similar to those noted in our study. Characteristic cytoplasmic blebs were connected to changes in the environmental availability of nutrients, oxygen, and overall stress response [127].

Releasing the ADI to CFS opens new possibilities to develop a well-defined postbiotic drug. CFS could be used directly, or the L. lactis Lc4 strain may serve as a live vector, capable of delivering and releasing ADI at a targeted site of action. In the context of CRC, delivering bioactive proteins orally remains challenging due to the presence of digestive enzymes and varying pH levels. However, our data from viability in SHIME growth media, suggest that the Lc4 strain could survive and retain enzymatic activity in the GI tract. L. lactis strains and other GRAS bacteria, therefore, which are resistant to the mentioned environmental factors, could be promising alternatives even for high molecular mass proteins vulnerable to digestion [128, 129]. Several studies have demonstrated that large proteins, native or heterologously expressed, could be active and impart beneficial effects on the host in vivo via the digestive tract [52–54, 130]. Furthermore, the ability of a strain to release active proteins with low or non-immunogenic effect, independent from phagocytosis and endosome/lysosome host digestion, would also be a desirable feature of a live vector application beyond the intestine [54].

Another potential mechanism facilitated by the oral administration of the Lc4 strain as a live vector is the reduction of free arginine reservoirs and the total amount absorbed from the intestines. L-arginine, a semi-essential amino acid, plays diverse roles in the human body, including stem cell proliferation, immune response regulation, nitric oxide (NO) production, polyamine synthesis, and tumor immune cell infiltration [104]. However, the influence of arginine and its metabolites on CRC appears to be bidirectional [131] and still needs to be experimentally evaluated due to contradictory results. Some studies have reported a positive influence of ARG supplementation on CRC patients [132] or immunotherapy responses [131] others reported increased polyamine synthesis [133] positive associations between CRC prevalence and consumption of ARG [104, 134]. Recent findings have shown that CRC tumors overexpress arginine transporters [104], and elevated plasma L-arginine levels are positively correlated with CRC occurrence [135]. These findings suggest new opportunities for using the Lc4 strain as a prophylaxis intervention (as probiotic or postbiotic). The Lc4 strain could lower the ARG reservoir and prevent carcinogenesis, and/or reduce excessive NO-mediated inflammatory response in the intestines - which is an additional known factor in CRC [136–138].

L-arginine and ADI-pathway are crucial in various other gut-inhabiting organisms, including commensal and pathogenic bacteria [139–141]. Several studies reported a positive influence of ARG supplementation on microbiome composition and intestine fitness during colitis [142]. Conversely, ARG may facilitate the evasion and expansion of various bacterial, fungal, and parasitic pathobionts especially during dysbiosis [141]. To our best knowledge, there is no data evaluating the interaction between ARG and microbiome composition during CRC. Potentially, by lowering and/or competing for ARG in intestines, the Lc4 strain could reduce biofilm formation, expression of virulence factors and dampen the evasion of pathogenic bacteria, including *Escherichia coli* and * Shigella *spp which could be negatively associated with CRC progression [139, 143].

The nature of these findings, combined with differences in genetic and protein backgrounds, underscores the complexity of CRC as an individual disease, where the role of arginine depends on various factors, such as the overall host health and microbiome, tumor stage and progression, and molecular and genetic characteristics of the disease. This conclusion highlights the need for further investigation into the L-arginine metabolism in CRC and the efficacy of Lc4-ADI/Lc4 strain in relevant experimental settings. Our results confirmed that ADI originating from *L. lactis* Lc4 strain may be a promising candidate for *ASS1*-negative malignancies, with the potential to reduce the growth of cancers with *ASS1* and *ASL* overexpression, which awaits examination in future studies. The presented findings show promise for dairy-originating LABs as a source of bioactive molecules and probiotics/postbiotics with a potentially broad therapeutic application.

## Conclusions

In conclusion, we employed an extended proteomic and phenotypic approach to perform the anti-proliferative screening of *L. lactis* strains from natural sources. Our results unveiled arginine deiminase as a metabolic factor responsible for the cytostatic activity of *L. lactis* Lc4 CFS. We identified a specific LAB strain, originating from a dairy source, with the ability to autolyze and release cytoplasm, which could have potential as a promising pro-health agent. These results pave the way to develop a well-defined ADI-rich-CFS postbiotic, with promising beneficial effects in CRC therapy. Further research should be conducted for *L. lactis* Lc4 strain with respect to its application as a therapeutic in the GI tract.

### Electronic supplementary material

Below is the link to the electronic supplementary material.

Supplementary Material 1

Supplementary Material 2

Supplementary Material 3

Supplementary Material 4

## Data Availability

The authors confirm that the data supporting the findings of this study are available within the article and its supplementary materials.
