# Supplementary material for: The strain-dependent cytostatic activity of Lactococcus lactis on CRC cell lines is mediated through the release of arginine deiminase
Source: Microb Cell Fact. 2024 Mar 14;23:82. doi: 10.1186/s12934-024-02345-w (PMC10938756; doi:10.1186/s12934-024-02345-w)
Supplement: Supplementary file 1 — Supplementary Material 1 [file 12934_2024_2345_MOESM1_ESM.pdf]

Proteins identified in F>50kDa fraction

Family	Member	Database	Accession	Score	Mass	Num. of matches	Num. of significant matches	Num. of sequences	Num. of significant sequences	emPAI	Description
23	1	lactococcus	Q9CEN7	5874	135106	127	127	48	48	5.16	DNA-directed RNA polymerase subunit beta' OS=Lactococcus lactis subsp. lactis (strain IL1403) OX=272623 GN=rpoC PE=3 SV=2
4	1	lactococcus	P0A3J0	41995	64947	586	586	42	42	21.65	Chaperone protein DnaK OS=Lactococcus lactis subsp. lactis (strain IL1403) OX=272623 GN=dnaK PE=3 SV=1
22	1	lactococcus	Q9CIQ1	5880	95414	147	147	37	37	4.88	Aminopeptidase N OS=Lactococcus lactis subsp. lactis (strain IL1403) OX=272623 GN=pepN PE=3 SV=1
44	1	lactococcus	Q9CEN6	4140	133208	88	88	36	36	2.57	DNA-directed RNA polymerase subunit beta OS=Lactococcus lactis subsp. lactis (strain IL1403) OX=272623 GN=rpoB PE=3 SV=1
2	1	lactococcus	P37282	56962	57166	865	865	35	35	24.69	60 kDa chaperonin OS=Lactococcus lactis subsp. lactis (strain IL1403) OX=272623 GN=groL PE=3 SV=2
32	1	lactococcus	Q9CI09	5143	83277	91	91	34	34	5.22	ATP-dependent Clp protease ATP-binding subunit ClpE OS=Lactococcus lactis subsp. lactis (strain IL1403) OX=272623 GN=clpE PE=3 SV=1
21	1	lactococcus	Q9CE12	5959	62780	127	127	32	32	8.81	Arginine--tRNA ligase OS=Lactococcus lactis subsp. lactis (strain IL1403) OX=272623 GN=argS PE=3 SV=1
39	1	lactococcus	Q9CFV2	4459	118138	89	89	31	31	2.63	Carbamoyl-phosphate synthase large chain OS=Lactococcus lactis subsp. lactis (strain IL1403) OX=272623 GN=carB PE=3 SV=1
43	1	lactococcus	Q9CE01	4169	87733	92	92	31	31	3.46	Xaa-Pro dipeptidyl-peptidase OS=Lactococcus lactis subsp. lactis (strain IL1403) OX=272623 GN=pepX PE=3 SV=1
41	1	lactococcus	Q9CHE0	4350	75775	85	85	30	30	06.03	Methionine--tRNA ligase OS=Lactococcus lactis subsp. lactis (strain IL1403) OX=272623 GN=metG PE=3 SV=1
47	1	lactococcus	Q9CEH8	3815	106672	82	82	29	29	2.42	Isoleucine--tRNA ligase OS=Lactococcus lactis subsp. lactis (strain IL1403) OX=272623 GN=ileS PE=3 SV=1
35	1	lactococcus	O32797	4714	89341	86	86	29	29	4.24	Formate acetyltransferase OS=Lactococcus lactis subsp. lactis (strain IL1403) OX=272623 GN=pfl PE=3 SV=1
1	1	lactococcus	Q9CHS7	79441	46929	1111	1111	28	28	16.64	Enolase 1 OS=Lactococcus lactis subsp. lactis (strain IL1403) OX=272623 GN=eno1 PE=3 SV=1
28	1	lactococcus	Q9CEV7	5425	69840	110	110	28	28	5.52	Oligoendopeptidase F homolog OS=Lactococcus lactis subsp. lactis (strain IL1403) OX=272623 GN=pepF PE=3 SV=1
29	1	lactococcus	Q9CHU6	5359	52484	103	103	28	28	11.05	6-phosphogluconate dehydrogenase, decarboxylating OS=Lactococcus lactis subsp. lactis (strain IL1403) OX=272623 GN=gnd PE=3 SV=1
16	1	lactococcus	Q9CJ82	10592	62613	240	240	27	27	8.89	Phosphoenolpyruvate-protein phosphotransferase OS=Lactococcus lactis subsp. lactis (strain IL1403) OX=272623 GN=ptsI PE=3 SV=1
7	1	lactococcus	Q9CIW1	26701	42044	428	428	27	27	15.45	Phosphoglycerate kinase OS=Lactococcus lactis subsp. lactis (strain IL1403) OX=272623 GN=pgk PE=3 SV=1
65	1	lactococcus	Q9CDZ7	2903	55427	70	70	26	26	8.81	Glutamate--tRNA ligase OS=Lactococcus lactis subsp. lactis (strain IL1403) OX=272623 GN=gltX PE=3 SV=1
5	1	lactococcus	Q07637	38029	54267	678	678	26	26	13.03	Pyruvate kinase OS=Lactococcus lactis subsp. lactis (strain IL1403) OX=272623 GN=pyk PE=3 SV=2
54	1	lactococcus	Q9CIY6	3611	52839	69	69	25	25	11.84	Inosine-5'-monophosphate dehydrogenase OS=Lactococcus lactis subsp. lactis (strain IL1403) OX=272623 GN=guaB PE=3 SV=1
30	1	lactococcus	Q9CFJ0	5239	56837	94	94	25	25	7.60	GMP synthase [glutamine-hydrolyzing] OS=Lactococcus lactis subsp. lactis (strain IL1403) OX=272623 GN=guaA PE=3 SV=1
111	1	lactococcus	Q9CG80	1420	81197	36	36	23	23	2.31	DNA topoisomerase 1 OS=Lactococcus lactis subsp. lactis (strain IL1403) OX=272623 GN=topA PE=3 SV=1
57	1	lactococcus	Q07744	3437	71493	72	72	23	23	2.89	Neutral endopeptidase OS=Lactococcus lactis subsp. lactis (strain IL1403) OX=272623 GN=pepO PE=1 SV=3
20	1	lactococcus	Q9CE93	6334	47480	111	111	23	23	7.39	Adenylosuccinate synthetase OS=Lactococcus lactis subsp. lactis (strain IL1403) OX=272623 GN=purA PE=3 SV=1
32	2	lactococcus	Q9CFF3	1573	97275	41	41	23	23	1.84	Chaperone protein ClpB OS=Lactococcus lactis subsp. lactis (strain IL1403) OX=272623 GN=clpB PE=3 SV=1
77	1	lactococcus	Q9CH07	2407	59543	60	60	22	22	3.76	Formate--tetrahydrofolate ligase OS=Lactococcus lactis subsp. lactis (strain IL1403) OX=272623 GN=fhs PE=3 SV=1
46	1	lactococcus	Q9CF40	3852	49702	80	80	22	22	08.05	Adenylosuccinate lyase OS=Lactococcus lactis subsp. lactis (strain IL1403) OX=272623 GN=purB PE=3 SV=1
24	1	lactococcus	Q9CEM7	5747	45943	107	107	22	22	12.01	Peptidase T OS=Lactococcus lactis subsp. lactis (strain IL1403) OX=272623 GN=pepT PE=3 SV=1
40	1	lactococcus	Q9CH96	4406	52003	94	94	22	22	7.24	Dipeptidase OS=Lactococcus lactis subsp. lactis (strain IL1403) OX=272623 GN=pepV PE=4 SV=1

59	1	lactococcus	Q9CHB6	3274	93733	58	58	22	22	2.23	Leucine--tRNA ligase OS=Lactococcus lactis subsp. lactis (strain IL1403) OX=272623 GN=leuS PE=3 SV=1
26	1	lactococcus	Q9CHA0	5737	44683	105	105	21	21	8.57	30S ribosomal protein S1 OS=Lactococcus lactis subsp. lactis (strain IL1403) OX=272623 GN=rpsA PE=4 SV=1
49	1	lactococcus	Q9CDG1	3761	77907	72	72	21	21	2.88	Elongation factor G OS=Lactococcus lactis subsp. lactis (strain IL1403) OX=272623 GN=fusA PE=3 SV=1
58	1	lactococcus	Q9CFC3	3287	53793	71	71	21	21	4.60	Probable dipeptidase B OS=Lactococcus lactis subsp. lactis (strain IL1403) OX=272623 GN=pepDB PE=3 SV=1
92	1	lactococcus	Q9CF56	2170	71681	52	52	20	20	2.88	Transketolase OS=Lactococcus lactis subsp. lactis (strain IL1403) OX=272623 GN=tkt PE=3 SV=1
11	1	lactococcus	P81181	17131	49564	225	225	20	20	4.95	Glucose-6-phosphate isomerase OS=Lactococcus lactis subsp. lactis (strain IL1403) OX=272623 GN=pgi PE=1 SV=3
107	1	lactococcus	Q9CDV8	1547	55233	38	38	20	20	3.96	Threonine synthase OS=Lactococcus lactis subsp. lactis (strain IL1403) OX=272623 GN=thrC PE=3 SV=1
14	1	lactococcus	Q9CDR5	12899	36647	214	214	20	20	14.68	Elongation factor Ts OS=Lactococcus lactis subsp. lactis (strain IL1403) OX=272623 GN=tsf PE=3 SV=1
81	1	lactococcus	Q9CIG5	2354	26749	60	60	19	19	25.71	Two-component system regulator OS=Lactococcus lactis subsp. lactis (strain IL1403) OX=272623 GN=llrC PE=4 SV=1
131	1	lactococcus	Q9CDR3	1058	98298	29	29	19	19	1.26	Aldehyde-alcohol dehydrogenase OS=Lactococcus lactis subsp. lactis (strain IL1403) OX=272623 GN=adhE PE=3 SV=1
37	1	lactococcus	Q9CI15	4609	46902	80	80	18	18	05.01	Trigger factor OS=Lactococcus lactis subsp. lactis (strain IL1403) OX=272623 GN=tig PE=3 SV=1
3	1	lactococcus	Q9CDJ1	49593	47025	665	665	18	18	4.99	Peptidase C51 domain-containing protein OS=Lactococcus lactis subsp. lactis (strain IL1403) OX=272623 GN=usp45 PE=4 SV=1
84	1	lactococcus	Q9CEG3	2313	49975	52	52	18	18	3.56	Aminopeptidase C OS=Lactococcus lactis subsp. lactis (strain IL1403) OX=272623 GN=pepC PE=3 SV=3
87	1	lactococcus	Q9CEX2	2232	47531	50	50	18	18	4.89	Serine--tRNA ligase OS=Lactococcus lactis subsp. lactis (strain IL1403) OX=272623 GN=serS PE=3 SV=1
95	1	lactococcus	Q9CDT4	1971	69254	46	46	17	17	2.38	Proline--tRNA ligase OS=Lactococcus lactis subsp. lactis (strain IL1403) OX=272623 GN=proS PE=3 SV=1
73	1	lactococcus	Q7DAV2	2533	60825	42	42	17	17	2.74	Alpha-acetolactate synthase OS=Lactococcus lactis subsp. lactis (strain IL1403) OX=272623 GN=als PE=3 SV=1
182	1	lactococcus	Q9CDP6	604	100475	18	18	16	16	0.96	Valine--tRNA ligase OS=Lactococcus lactis subsp. lactis (strain IL1403) OX=272623 GN=valS PE=3 SV=1
62	1	lactococcus	Q9CEI0	3087	43185	55	55	16	16	4.24	Elongation factor Tu OS=Lactococcus lactis subsp. lactis (strain IL1403) OX=272623 GN=tuf PE=3 SV=1
31	1	lactococcus	P0DOB5	5149	35829	117	117	16	16	7.24	ATP-dependent 6-phosphofructokinase OS=Lactococcus lactis subsp. lactis (strain IL1403) OX=272623 GN=pfkA PE=3 SV=1
90	1	lactococcus	Q9CE35	2220	43110	57	57	16	16	4.26	Acetate kinase 1 OS=Lactococcus lactis subsp. lactis (strain IL1403) OX=272623 GN=ackA1 PE=3 SV=1
51	1	lactococcus	Q9CJD8	3727	49928	78	78	16	16	3.97	Dihydrolipoyl dehydrogenase OS=Lactococcus lactis subsp. lactis (strain IL1403) OX=272623 GN=pdhD PE=3 SV=1
93	1	lactococcus	Q9CJD7	2040	56282	41	41	16	16	4.20	Dihydrolipoamide acetyltransferase component of pyruvate dehydrogenase complex OS=Lactococcus lactis subsp. lactis (strain IL1403) OX=272623 GN=pdhC PE=3 SV=1
8	1	lactococcus	Q9CED4	26336	32153	423	423	16	16	12.55	Fructose-bisphosphate aldolase OS=Lactococcus lactis subsp. lactis (strain IL1403) OX=272623 GN=fbaA PE=4 SV=1
25	1	lactococcus	Q9CDL9	5742	49800	90	90	16	16	3.98	Glutamine synthetase OS=Lactococcus lactis subsp. lactis (strain IL1403) OX=272623 GN=glnA PE=3 SV=1
122	1	lactococcus	Q9CJE0	1147	43233	30	30	15	15	3.31	Aminotransferase OS=Lactococcus lactis subsp. lactis (strain IL1403) OX=272623 GN=araT PE=3 SV=1
60	1	lactococcus	Q9CIT8	3266	31040	66	66	15	15	6.57	Oxidoreductase OS=Lactococcus lactis subsp. lactis (strain IL1403) OX=272623 GN=ycgG PE=4 SV=1
72	1	lactococcus	P58013	2587	46106	46	46	15	15	4.68	Arginine deiminase OS=Lactococcus lactis subsp. lactis (strain IL1403) OX=272623 GN=arcA PE=3 SV=1
12	1	lactococcus	Q01462	15508	35075	267	267	15	15	6.65	L-lactate dehydrogenase 1 OS=Lactococcus lactis subsp. lactis (strain IL1403) OX=272623 GN=ldh1 PE=3 SV=3
96	1	lactococcus	Q9CED6	1873	41036	49	49	15	15	4.15	DUF4097 domain-containing protein OS=Lactococcus lactis subsp. lactis (strain IL1403) OX=272623 GN=ythC PE=4 SV=1
38	1	lactococcus	Q9CJ45	4512	29202	82	82	15	15	14.28	Transcriptional regulator OS=Lactococcus lactis subsp. lactis (strain IL1403) OX=272623 GN=codY PE=3 SV=1
88	1	lactococcus	Q9CG49	2231	56228	47	47	15	15	2.57	D-alanine--D-alanyl carrier protein ligase OS=Lactococcus lactis subsp. lactis (strain IL1403) OX=272623 GN=dltA PE=3 SV=1
50	1	lactococcus	P58117	3756	23857	78	78	15	15	22.35	Adenylate kinase OS=Lactococcus lactis subsp. lactis (strain IL1403) OX=272623 GN=adk PE=3 SV=1
195	1	lactococcus	Q9CGI5	551	92495	16	16	14	14	0.90	DNA gyrase subunit A OS=Lactococcus lactis subsp. lactis (strain IL1403) OX=272623 GN=gyrA PE=3 SV=1
135	1	lactococcus	Q9CH92	998	47795	25	25	14	14	2.75	Glutathione reductase OS=Lactococcus lactis subsp. lactis (strain IL1403) OX=272623 GN=gshR PE=3 SV=1

33	1	lactococcus	P50918	5140	27027	96	96	14	14	9.25	Triosephosphate isomerase OS=Lactococcus lactis subsp. lactis (strain IL1403) OX=272623 GN=tpiA PE=1 SV=3
80	1	lactococcus	P0C2U0	2354	39477	49	49	14	14	5.10	Ornithine carbamoyltransferase, catabolic OS=Lactococcus lactis subsp. lactis (strain IL1403) OX=272623 GN=arcB PE=3 SV=1
6	1	lactococcus	Q9CDH4	30429	35889	554	554	14	14	9.41	Glyceraldehyde-3-phosphate dehydrogenase OS=Lactococcus lactis subsp. lactis (strain IL1403) OX=272623 GN=gapB PE=3 SV=1
121	1	lactococcus	Q9CH71	1149	83390	25	25	14	14	01.03	Penicillin-binding protein OS=Lactococcus lactis subsp. lactis (strain IL1403) OX=272623 GN=pbpX PE=3 SV=1
55	1	lactococcus	Q9CEX8	3521	35042	76	76	14	14	6.65	EIIAB-Man OS=Lactococcus lactis subsp. lactis (strain IL1403) OX=272623 GN=ptnAB PE=4 SV=1
78	1	lactococcus	Q9CHF8	2400	33567	49	49	14	14	6.41	Malonyl CoA-acyl carrier protein transacylase OS=Lactococcus lactis subsp. lactis (strain IL1403) OX=272623 GN=fabD PE=3 SV=1
18	1	lactococcus	Q9CIM0	8033	26313	146	146	14	14	11.74	2,3-bisphosphoglycerate-dependent phosphoglycerate mutase OS=Lactococcus lactis subsp. lactis (strain IL1403) OX=272623 GN=gpmA PE=3 SV=1
89	1	lactococcus	Q9CH05	2230	30636	44	44	14	14	7.93	Amino acid ABC transporter substrate binding protein OS=Lactococcus lactis subsp. lactis (strain IL1403) OX=272623 GN=yjgC PE=4 SV=1
15	1	lactococcus	Q9CF79	11254	34674	219	219	14	14	10.30	Aspartate carbamoyltransferase OS=Lactococcus lactis subsp. lactis (strain IL1403) OX=272623 GN=pyrB PE=3 SV=1
74	1	lactococcus	Q9CF22	2463	35326	47	47	14	14	4.96	Phosphate acetyltransferase OS=Lactococcus lactis subsp. lactis (strain IL1403) OX=272623 GN=pta PE=3 SV=1
61	1	lactococcus	Q9CEF2	3235	18468	63	63	13	13	17.78	Uncharacterized protein OS=Lactococcus lactis subsp. lactis (strain IL1403) OX=272623 GN=ytgH PE=3 SV=1
114	1	lactococcus	Q9CH12	1308	45787	34	34	13	13	2.31	Phosphopentomutase OS=Lactococcus lactis subsp. lactis (strain IL1403) OX=272623 GN=deoB PE=3 SV=1
100	1	lactococcus	Q9CH02	1800	33965	40	40	13	13	6.22	Thioredoxin reductase OS=Lactococcus lactis subsp. lactis (strain IL1403) OX=272623 GN=trxB1 PE=3 SV=1
115	1	lactococcus	Q9CFX6	1294	37121	24	24	13	13	3.89	Glycerol-3-phosphate dehydrogenase [NAD(P)+] OS=Lactococcus lactis subsp. lactis (strain IL1403) OX=272623 GN=gpsA PE=3 SV=1
97	1	lactococcus	Q04506	1870	44052	42	42	13	13	3.61	RNA polymerase sigma factor SigA OS=Lactococcus lactis subsp. lactis (strain IL1403) OX=272623 GN=sigA PE=3 SV=2
101	1	lactococcus	Q9CHG2	1774	35638	43	43	13	13	4.21	Mannose-6-phosphate isomerase OS=Lactococcus lactis subsp. lactis (strain IL1403) OX=272623 GN=pmi PE=3 SV=1
157	1	lactococcus	Q9CID5	724	87351	17	17	12	12	0.88	Trehalose 6-phosphate phosphorylase OS=Lactococcus lactis subsp. lactis (strain IL1403) OX=272623 GN=trePP PE=1 SV=3
10	1	lactococcus	Q9CFM9	19717	36722	285	285	12	12	3.42	Basic membrane protein A OS=Lactococcus lactis subsp. lactis (strain IL1403) OX=272623 GN=bmpA PE=3 SV=1
42	1	lactococcus	Q9LA06	4253	41623	65	65	12	12	3.12	Serine protease Do-like HtrA OS=Lactococcus lactis subsp. lactis (strain IL1403) OX=272623 GN=htrA PE=1 SV=1
132	1	lactococcus	Q9CGF1	1056	35952	23	23	12	12	3.56	GMP reductase OS=Lactococcus lactis subsp. lactis (strain IL1403) OX=272623 GN=guaC PE=3 SV=1
45	1	lactococcus	Q9CDY3	4128	34173	67	67	12	12	3.93	DNA-directed RNA polymerase subunit alpha OS=Lactococcus lactis subsp. lactis (strain IL1403) OX=272623 GN=rpoA PE=3 SV=1
82	1	lactococcus	Q9CEF8	2349	49195	37	37	12	12	2.32	Bifunctional protein GlmU OS=Lactococcus lactis subsp. lactis (strain IL1403) OX=272623 GN=glmU PE=3 SV=1
66	1	lactococcus	Q9CE25	2885	34001	59	59	12	12	3.99	Glucokinase OS=Lactococcus lactis subsp. lactis (strain IL1403) OX=272623 GN=glk PE=3 SV=1
106	1	lactococcus	Q9CGY9	1584	20568	32	32	12	12	19.97	Protein GrpE OS=Lactococcus lactis subsp. lactis (strain IL1403) OX=272623 GN=grpE PE=3 SV=1
56	1	lactococcus	Q9CGM8	3518	22619	67	67	12	12	8.13	Orotate phosphoribosyltransferase OS=Lactococcus lactis subsp. lactis (strain IL1403) OX=272623 GN=pyrE PE=3 SV=1
85	1	lactococcus	Q9CIH3	2264	38339	49	49	12	12	3.65	Glutamyl aminopeptidase OS=Lactococcus lactis subsp. lactis (strain IL1403) OX=272623 GN=pepA PE=3 SV=1
69	1	lactococcus	Q9CI26	2781	32338	56	56	12	12	5.16	Cysteine synthase OS=Lactococcus lactis subsp. lactis (strain IL1403) OX=272623 GN=cysM PE=3 SV=1
137	1	lactococcus	Q9CHE1	957	38145	19	19	12	12	2.75	Exodeoxyribonuclease III OS=Lactococcus lactis subsp. lactis (strain IL1403) OX=272623 GN=exoA PE=3 SV=1
98	1	lactococcus	Q9CDW5	1859	29642	39	39	12	12	7.34	50S ribosomal protein L2 OS=Lactococcus lactis subsp. lactis (strain IL1403) OX=272623 GN=rplB PE=3 SV=1
83	1	lactococcus	Q9CF32	2331	40154	44	44	12	12	4.92	Proline dipeptidase OS=Lactococcus lactis subsp. lactis (strain IL1403) OX=272623 GN=pepQ PE=3 SV=1

222	1	lactococcus	Q9CDJ9	465	57839	14	14	11	11	1.23	Glucose-6-phosphate 1-dehydrogenase OS=Lactococcus lactis subsp. lactis (strain IL1403) OX=272623 GN=zwf PE=3 SV=1
1	2	lactococcus	Q9CIT0	1775	45781	34	34	11	11	02.02	Enolase 2 OS=Lactococcus lactis subsp. lactis (strain IL1403) OX=272623 GN=eno2 PE=3 SV=1
167	1	lactococcus	Q9CGJ6	674	56404	15	15	11	11	1.28	Nicotinate phosphoribosyltransferase OS=Lactococcus lactis subsp. lactis (strain IL1403) OX=272623 GN=ylaF PE=3 SV=1
52	1	lactococcus	Q9CEI4	3694	35348	56	56	11	11	3.17	Putative ribose-phosphate pyrophosphokinase 2 OS=Lactococcus lactis subsp. lactis (strain IL1403) OX=272623 GN=prs2 PE=3 SV=1
143	1	lactococcus	Q9CE66	870	36299	18	18	11	11	2.57	UDP-glucose 4-epimerase OS=Lactococcus lactis subsp. lactis (strain IL1403) OX=272623 GN=galE PE=3 SV=1
116	1	lactococcus	Q9CIL2	1283	59707	27	27	11	11	1.33	Oligopeptide ABC transporter substrate binding protein OS=Lactococcus lactis subsp. lactis (strain IL1403) OX=272623 GN=optA PE=3 SV=1
144	1	lactococcus	Q9CJ07	869	33123	21	21	11	11	3.58	dTDP-4-dehydrorhamnose reductase OS=Lactococcus lactis subsp. lactis (strain IL1403) OX=272623 GN=rmlC PE=3 SV=1
64	1	lactococcus	Q9CHB8	2961	35615	47	47	11	11	3.63	Ribose-phosphate pyrophosphokinase 1 OS=Lactococcus lactis subsp. lactis (strain IL1403) OX=272623 GN=prs1 PE=3 SV=1
76	1	lactococcus	P0A4K2	2457	40912	47	47	11	11	02.09	Cystathionine beta-lyase OS=Lactococcus lactis subsp. lactis (strain IL1403) OX=272623 GN=metC PE=3 SV=1
120	1	lactococcus	Q9CFW8	1174	33125	19	19	11	11	4.20	Dihydroorotate dehydrogenase B (NAD(+)), catalytic subunit OS=Lactococcus lactis subsp. lactis (strain IL1403) OX=272623 GN=pyrDB PE=1 SV=1
71	1	lactococcus	Q9CF33	2592	36624	45	45	11	11	3.98	Catabolite control protein A OS=Lactococcus lactis subsp. lactis (strain IL1403) OX=272623 GN=ccpA PE=1 SV=1
209	1	lactococcus	Q9CFG0	501	57196	12	12	10	10	1.25	Bifunctional purine biosynthesis protein PurH OS=Lactococcus lactis subsp. lactis (strain IL1403) OX=272623 GN=purH PE=3 SV=1
6	2	lactococcus	P52987	1326	36080	28	28	10	10	03.04	Glyceraldehyde-3-phosphate dehydrogenase OS=Lactococcus lactis subsp. lactis (strain IL1403) OX=272623 GN=gap PE=3 SV=2
176	1	lactococcus	Q9CEI6	639	84710	16	16	10	10	0.65	Polyribonucleotide nucleotidyltransferase OS=Lactococcus lactis subsp. lactis (strain IL1403) OX=272623 GN=pnp PE=3 SV=1
9	1	lactococcus	Q9CG42	23837	12401	397	397	10	10	26.38	50S ribosomal protein L7/L12 OS=Lactococcus lactis subsp. lactis (strain IL1403) OX=272623 GN=rplL PE=3 SV=1
188	1	lactococcus	Q9CHL1	564	43072	13	13	10	10	1.66	Meth_synt_2 domain-containing protein OS=Lactococcus lactis subsp. lactis (strain IL1403) OX=272623 GN=yhcE PE=4 SV=1
130	1	lactococcus	Q9CJD5	1063	41299	22	22	10	10	02.07	Pyruvate dehydrogenase E1 component subunit alpha OS=Lactococcus lactis subsp. lactis (strain IL1403) OX=272623 GN=pdhA PE=4 SV=1
156	1	lactococcus	Q9CDR4	729	28521	18	18	10	10	4.82	30S ribosomal protein S2 OS=Lactococcus lactis subsp. lactis (strain IL1403) OX=272623 GN=rpsB PE=3 SV=1
79	1	lactococcus	O86271	2376	37449	43	43	10	10	02.08	Uncharacterized protein YwcC OS=Lactococcus lactis subsp. lactis (strain IL1403) OX=272623 GN=ywcC PE=3 SV=1
169	1	lactococcus	Q9CHK0	670	62531	13	13	10	10	1.10	2-succinyl-5-enolpyruvyl-6-hydroxy-3-cyclohexene-1-carboxylate synthase OS=Lactococcus lactis subsp. lactis (strain IL1403) OX=272623 GN=menD PE=3 SV=1
110	1	lactococcus	Q9CJJ1	1488	42238	29	29	10	10	1.70	Beta sliding clamp OS=Lactococcus lactis subsp. lactis (strain IL1403) OX=272623 GN=dnaN PE=3 SV=1
166	1	lactococcus	Q9CE18	676	42909	18	18	10	10	1.66	Aminotransferase OS=Lactococcus lactis subsp. lactis (strain IL1403) OX=272623 GN=arcT PE=3 SV=1
138	1	lactococcus	Q9CHB9	940	51036	20	20	10	10	1.28	Signal recognition particle receptor FtsY OS=Lactococcus lactis subsp. lactis (strain IL1403) OX=272623 GN=ftsY PE=3 SV=1
119	1	lactococcus	Q9CI02	1257	26458	34	34	10	10	5.69	Enoyl-[acyl-carrier-protein] reductase [NADH] OS=Lactococcus lactis subsp. lactis (strain IL1403) OX=272623 GN=fabI PE=3 SV=1
123	1	lactococcus	Q9CEF7	1138	27955	24	24	10	10	05.03	Pyrroline-5-carboxylate reductase OS=Lactococcus lactis subsp. lactis (strain IL1403) OX=272623 GN=proC PE=3 SV=1
286	1	lactococcus	Q9CHL2	296	35889	9	9	9	9	1.87	Zinc-type alcohol dehydrogenase-like protein OS=Lactococcus lactis subsp. lactis (strain IL1403) OX=272623 GN=qor PE=3 SV=1
219	1	lactococcus	Q9CJ71	475	33774	13	13	9	9	02.06	R3H domain-containing protein OS=Lactococcus lactis subsp. lactis (strain IL1403) OX=272623 GN=ybdD PE=4 SV=1
289	1	lactococcus	Q9CHM9	286	54422	9	9	9	9	01.01	Glycogen synthase OS=Lactococcus lactis subsp. lactis (strain IL1403) OX=272623 GN=glgA PE=3 SV=2
34	1	lactococcus	Q9CDX2	4798	12914	77	77	9	9	23.51	50S ribosomal protein L14 OS=Lactococcus lactis subsp. lactis (strain IL1403) OX=272623 GN=rplN PE=3 SV=1

213	1	lactococcus	Q9CDS3	490	36724	16	16	9	9	1.80	Uncharacterized protein OS=Lactococcus lactis subsp. lactis (strain IL1403) OX=272623 GN=ywaI PE=4 SV=1
17	1	lactococcus	Q9CG71	9370	20199	124	124	9	9	6.86	Uncharacterized protein OS=Lactococcus lactis subsp. lactis (strain IL1403) OX=272623 GN=ymgG PE=3 SV=1
152	1	lactococcus	Q9CI76	779	42237	14	14	9	9	1.45	Amino acids and amines aminotransferase OS=Lactococcus lactis subsp. lactis (strain IL1403) OX=272623 GN=yeiG PE=3 SV=1
94	1	lactococcus	Q9CE39	2035	20735	36	36	9	9	8.10	Ribosome-recycling factor OS=Lactococcus lactis subsp. lactis (strain IL1403) OX=272623 GN=frr PE=3 SV=1
187	1	lactococcus	Q9CFF0	569	54973	14	14	9	9	0.99	Amidophosphoribosyltransferase OS=Lactococcus lactis subsp. lactis (strain IL1403) OX=272623 GN=purF PE=3 SV=1
63	1	lactococcus	Q9CHQ3	3072	22068	73	73	9	9	13.07	ATP-dependent Clp protease proteolytic subunit OS=Lactococcus lactis subsp. lactis (strain IL1403) OX=272623 GN=clpP PE=3 SV=1
154	1	lactococcus	Q9CFM7	752	23549	20	20	9	9	3.94	Deoxyribose-phosphate aldolase OS=Lactococcus lactis subsp. lactis (strain IL1403) OX=272623 GN=deoC PE=3 SV=1
105	1	lactococcus	Q9CH10	1684	25411	35	35	9	9	4.18	Purine nucleoside phosphorylase DeoD-type OS=Lactococcus lactis subsp. lactis (strain IL1403) OX=272623 GN=deoD PE=3 SV=1
174	1	lactococcus	Q9CIR9	646	40226	17	17	9	9	2.16	Adenosine deaminase OS=Lactococcus lactis subsp. lactis (strain IL1403) OX=272623 GN=add PE=3 SV=1
67	1	lactococcus	Q9CG41	2820	18076	49	49	9	9	6.90	50S ribosomal protein L10 OS=Lactococcus lactis subsp. lactis (strain IL1403) OX=272623 GN=rplJ PE=3 SV=1
155	1	lactococcus	Q9CJC5	732	61435	17	17	9	9	0.99	ABC transporter ATP binding protein OS=Lactococcus lactis subsp. lactis (strain IL1403) OX=272623 GN=yahG PE=4 SV=1
126	1	lactococcus	Q9CE17	1128	34097	20	20	9	9	02.03	Carbamate kinase OS=Lactococcus lactis subsp. lactis (strain IL1403) OX=272623 GN=arcC2 PE=3 SV=1
113	1	lactococcus	Q9CE23	1363	16642	25	25	9	9	14.49	Non-heme iron-binding ferritin OS=Lactococcus lactis subsp. lactis (strain IL1403) OX=272623 GN=dpsA PE=3 SV=1
140	1	lactococcus	Q9CJD6	921	35188	17	17	9	9	2.30	PDH E1 component beta subunit OS=Lactococcus lactis subsp. lactis (strain IL1403) OX=272623 GN=pdhB PE=4 SV=1
108	1	lactococcus	Q9CG22	1500	36969	30	30	9	9	2.12	Branched-chain-amino-acid aminotransferase OS=Lactococcus lactis subsp. lactis (strain IL1403) OX=272623 GN=bcaT PE=3 SV=1
128	1	lactococcus	Q9CHK2	1093	30998	25	25	9	9	2.87	1,4-dihydroxy-2-naphthoyl-CoA synthase OS=Lactococcus lactis subsp. lactis (strain IL1403) OX=272623 GN=menB PE=3 SV=1
133	1	lactococcus	Q9CJD1	1038	38027	23	23	9	9	02.02	Tryptophan--tRNA ligase OS=Lactococcus lactis subsp. lactis (strain IL1403) OX=272623 GN=trpS PE=3 SV=1
272	1	lactococcus	Q9CDJ4	332	27800	8	8	8	8	2.34	Aspartate racemase OS=Lactococcus lactis subsp. lactis (strain IL1403) OX=272623 GN=racD PE=3 SV=1
48	1	lactococcus	P0A4J1	3789	23240	78	78	8	8	3.22	Superoxide dismutase [Mn] OS=Lactococcus lactis subsp. lactis (strain IL1403) OX=272623 GN=sodA PE=3 SV=1
196	1	lactococcus	Q9CE62	549	37572	12	12	8	8	1.74	Aldose 1-epimerase OS=Lactococcus lactis subsp. lactis (strain IL1403) OX=272623 GN=galM PE=3 SV=1
70	1	lactococcus	Q9CHS8	2691	21270	56	56	8	8	4.82	Ribosome hibernation promoting factor OS=Lactococcus lactis subsp. lactis (strain IL1403) OX=272623 GN=ygdA PE=3 SV=1
232	1	lactococcus	Q9CHQ7	441	126649	10	10	8	8	0.31	Pyruvate carboxylase OS=Lactococcus lactis subsp. lactis (strain IL1403) OX=272623 GN=pycA PE=4 SV=1
91	1	lactococcus	Q9CF77	2189	19819	62	62	8	8	4.35	Pyrimidine operon regulatory protein OS=Lactococcus lactis subsp. lactis (strain IL1403) OX=272623 GN=pyrR PE=3 SV=1
171	1	lactococcus	Q9CHW7	663	44762	15	15	8	8	1.12	Serine hydroxymethyltransferase OS=Lactococcus lactis subsp. lactis (strain IL1403) OX=272623 GN=glyA PE=3 SV=1
117	1	lactococcus	Q9CDZ9	1271	31050	22	22	8	8	2.37	Amino acid ABC transporter substrate binding protein OS=Lactococcus lactis subsp. lactis (strain IL1403) OX=272623 GN=yvdF PE=4 SV=1
228	1	lactococcus	Q9CFI1	455	52952	12	12	8	8	01.05	Sugar ABC transporter substrate binding protein OS=Lactococcus lactis subsp. lactis (strain IL1403) OX=272623 GN=ypcG PE=4 SV=1
109	1	lactococcus	Q9CEG1	1495	28302	30	30	8	8	2.27	5'-methylthioadenosine/S-adenosylhomocysteine nucleosidase OS=Lactococcus lactis subsp. lactis (strain IL1403) OX=272623 GN=pfs PE=4 SV=1
183	1	lactococcus	Q9CJH5	599	50338	13	13	8	8	0.95	GW domain-containing protein OS=Lactococcus lactis subsp. lactis (strain IL1403) OX=272623 GN=yacG PE=4 SV=1
134	1	lactococcus	Q9CEK4	1009	15736	24	24	8	8	9.64	Usp domain-containing protein OS=Lactococcus lactis subsp. lactis (strain IL1403) OX=272623 GN=ytaA PE=3 SV=1

247	1	lactococcus	Q9CG26	399	47434	11	11	8	8	1.22	Diaminopimelate decarboxylase OS=Lactococcus lactis subsp. lactis (strain IL1403) OX=272623 GN=lysA PE=3 SV=1
118	1	lactococcus	Q9CH46	1270	21574	24	24	8	8	4.71	Peptidyl-prolyl cis-trans isomerase OS=Lactococcus lactis subsp. lactis (strain IL1403) OX=272623 GN=ppiB PE=3 SV=1
148	1	lactococcus	Q9CF59	820	39406	15	15	8	8	1.61	Aspartate-semialdehyde dehydrogenase OS=Lactococcus lactis subsp. lactis (strain IL1403) OX=272623 GN=asd PE=3 SV=1
136	1	lactococcus	Q9CHV0	974	34520	15	15	8	8	1.65	HPr kinase/phosphorylase OS=Lactococcus lactis subsp. lactis (strain IL1403) OX=272623 GN=hprK PE=3 SV=1
215	1	lactococcus	Q9CEC2	488	27279	14	14	8	8	2.41	Uncharacterized protein OS=Lactococcus lactis subsp. lactis (strain IL1403) OX=272623 GN=ytjH PE=4 SV=1
197	1	lactococcus	Q9CIF8	542	34313	12	12	8	8	1.66	Mevalonate kinase OS=Lactococcus lactis subsp. lactis (strain IL1403) OX=272623 GN=yeaG PE=4 SV=1
234	1	lactococcus	Q9CHT3	439	61175	10	10	8	8	0.74	Endolytic murein transglycosylase OS=Lactococcus lactis subsp. lactis (strain IL1403) OX=272623 GN=ygcC PE=3 SV=1
175	1	lactococcus	Q9CGH2	641	52866	13	13	8	8	01.05	Fumarate reductase flavoprotein subunit OS=Lactococcus lactis subsp. lactis (strain IL1403) OX=272623 GN=frdC PE=3 SV=1
223	1	lactococcus	Q9CFC7	465	36580	12	12	8	8	1.50	Oxidoreductase OS=Lactococcus lactis subsp. lactis (strain IL1403) OX=272623 GN=ypjF PE=4 SV=1
194	1	lactococcus	Q9CHT2	551	17263	11	11	8	8	5.81	Transcription elongation factor GreA OS=Lactococcus lactis subsp. lactis (strain IL1403) OX=272623 GN=greA PE=3 SV=1
216	1	lactococcus	Q9CDH8	482	49938	10	10	8	8	0.96	tRNA modification GTPase MnmE OS=Lactococcus lactis subsp. lactis (strain IL1403) OX=272623 GN=mnmE PE=3 SV=1
146	1	lactococcus	Q9CGY7	836	67403	18	18	8	8	0.65	Myosin-crossreactive antigen OS=Lactococcus lactis subsp. lactis (strain IL1403) OX=272623 GN=mycA PE=4 SV=1
184	1	lactococcus	Q9CIR6	580	61569	14	14	8	8	0.85	Ribonuclease J OS=Lactococcus lactis subsp. lactis (strain IL1403) OX=272623 GN=yciH PE=3 SV=1
177	1	lactococcus	Q9CGJ4	637	30263	15	15	8	8	02.02	NH(3)-dependent NAD(+) synthetase OS=Lactococcus lactis subsp. lactis (strain IL1403) OX=272623 GN=nadE PE=3 SV=1
372	1	lactococcus	Q9CII7	162	56579	7	7	7	7	0.68	Lysine--tRNA ligase OS=Lactococcus lactis subsp. lactis (strain IL1403) OX=272623 GN=lysS PE=3 SV=1
102	1	lactococcus	Q9CIU0	1771	17686	23	23	7	7	4.19	S-ribosylhomocysteine lyase OS=Lactococcus lactis subsp. lactis (strain IL1403) OX=272623 GN=luxS PE=3 SV=1
330	1	lactococcus	Q9CGI4	214	39698	8	8	7	7	1.10	FAD:protein FMN transferase OS=Lactococcus lactis subsp. lactis (strain IL1403) OX=272623 GN=apbE PE=3 SV=1
103	1	lactococcus	Q9CF87	1707	26668	24	24	7	7	2.51	Two-component system regulator OS=Lactococcus lactis subsp. lactis (strain IL1403) OX=272623 GN=llrA PE=4 SV=1
315	1	lactococcus	Q9CEW0	233	96422	8	8	7	7	0.36	Alanine--tRNA ligase OS=Lactococcus lactis subsp. lactis (strain IL1403) OX=272623 GN=alaS PE=3 SV=1
251	1	lactococcus	Q9CIU1	388	40276	13	13	7	7	01.08	Oxidoreductase OS=Lactococcus lactis subsp. lactis (strain IL1403) OX=272623 GN=ycgD PE=4 SV=1
297	1	lactococcus	Q9CF63	266	63531	8	8	7	7	0.59	Ribonuclease J OS=Lactococcus lactis subsp. lactis (strain IL1403) OX=272623 GN=yqgA PE=3 SV=1
124	1	lactococcus	Q9CHL0	1132	28018	22	22	7	7	2.85	Lactamase_B domain-containing protein OS=Lactococcus lactis subsp. lactis (strain IL1403) OX=272623 GN=yhcG PE=4 SV=1
284	1	lactococcus	Q9CJ46	300	45604	9	9	7	7	0.91	Aspartate aminotransferase OS=Lactococcus lactis subsp. lactis (strain IL1403) OX=272623 GN=aspC PE=4 SV=1
190	1	lactococcus	Q9CHG7	560	42878	14	14	7	7	1.19	Transcription termination/antitermination protein NusA OS=Lactococcus lactis subsp. lactis (strain IL1403) OX=272623 GN=nusA PE=3 SV=1
127	1	lactococcus	Q9CIH9	1102	21929	23	23	7	7	4.52	FMN-dependent NADH-azoreductase 2 OS=Lactococcus lactis subsp. lactis (strain IL1403) OX=272623 GN=azoR2 PE=3 SV=1
240	1	lactococcus	Q9CJ25	425	36391	9	9	7	7	1.24	Prenyl transferase OS=Lactococcus lactis subsp. lactis (strain IL1403) OX=272623 GN=preA PE=3 SV=1
141	1	lactococcus	Q9CF61	893	32262	16	16	7	7	1.48	4-hydroxy-tetrahydrodipicolinate synthase OS=Lactococcus lactis subsp. lactis (strain IL1403) OX=272623 GN=dapA PE=3 SV=1
153	1	lactococcus	Q9CFY8	756	34651	15	15	7	7	1.64	2-dehydropantoate 2-reductase OS=Lactococcus lactis subsp. lactis (strain IL1403) OX=272623 GN=LL1323 PE=3 SV=1
265	1	lactococcus	Q9CGT6	349	65708	8	8	7	7	0.57	Glutamine--fructose-6-phosphate aminotransferase [isomerizing] OS=Lactococcus lactis subsp. lactis (strain IL1403) OX=272623 GN=glmS PE=3 SV=2
178	1	lactococcus	Q9CF30	634	43307	15	15	7	7	0.97	THUMP domain-containing protein OS=Lactococcus lactis subsp. lactis (strain IL1403) OX=272623 GN=yqjE PE=4 SV=1

227	1	lactococcus	Q9CIH5	455	47221	12	12	7	7	0.87	Tyrosine--tRNA ligase OS=Lactococcus lactis subsp. lactis (strain IL1403) OX=272623 GN=tyrS PE=3 SV=1
162	1	lactococcus	Q9CES8	705	39465	18	18	7	7	1.11	3-dehydroquinate synthase OS=Lactococcus lactis subsp. lactis (strain IL1403) OX=272623 GN=aroB PE=3 SV=1
269	1	lactococcus	Q9CI05	341	28532	8	8	7	7	1.79	Glutamate or arginine ABC transporter substrate binding protein OS=Lactococcus lactis subsp. lactis (strain IL1403) OX=272623 GN=gltS PE=4 SV=1
180	1	lactococcus	Q9CFX9	619	41289	17	17	7	7	1.26	N-acetylglucosamine-6-phosphate deacetylase OS=Lactococcus lactis subsp. lactis (strain IL1403) OX=272623 GN=nagA PE=3 SV=1
235	1	lactococcus	Q9CFB0	437	39827	9	9	7	7	01.09	Acetyl coenzyme A acetyltransferase OS=Lactococcus lactis subsp. lactis (strain IL1403) OX=272623 GN=thiL PE=3 SV=1
142	1	lactococcus	Q9CGZ5	884	46926	16	16	7	7	1.24	UPF0210 protein YjhD OS=Lactococcus lactis subsp. lactis (strain IL1403) OX=272623 GN=yjhD PE=3 SV=1
274	1	lactococcus	Q9CHJ1	332	49608	8	8	7	7	0.81	Aspartokinase OS=Lactococcus lactis subsp. lactis (strain IL1403) OX=272623 GN=thrA PE=3 SV=1
179	1	lactococcus	Q9CFW7	623	29135	15	15	7	7	2.17	Dihydroorotate dehydrogenase B (NAD(+)), electron transfer subunit OS=Lactococcus lactis subsp. lactis (strain IL1403) OX=272623 GN=pyrK PE=1 SV=1
231	1	lactococcus	Q9CID7	446	17310	14	14	7	7	4.36	PTS EIIA type-1 domain-containing protein OS=Lactococcus lactis subsp. lactis (strain IL1403) OX=272623 GN=yedE PE=4 SV=1
163	1	lactococcus	Q9CHZ6	702	19453	18	18	7	7	7.52	Pantothenate metabolism flavoprotein OS=Lactococcus lactis subsp. lactis (strain IL1403) OX=272623 GN=dfpA PE=4 SV=1
241	1	lactococcus	Q9CFB1	424	44969	11	11	7	7	0.93	3-hydroxy-3-methylglutaryl coenzyme A reductase OS=Lactococcus lactis subsp. lactis (strain IL1403) OX=272623 GN=mvaA PE=3 SV=1
181	1	lactococcus	Q9CDM9	613	18339	15	15	7	7	3.90	Single-stranded DNA-binding protein 2 OS=Lactococcus lactis subsp. lactis (strain IL1403) OX=272623 GN=ssb2 PE=3 SV=1
170	1	lactococcus	Q9CHU7	665	20647	14	14	7	7	04.02	Uncharacterized protein OS=Lactococcus lactis subsp. lactis (strain IL1403) OX=272623 GN=ygaJ PE=4 SV=1
354	1	lactococcus	Q9CID9	184	48442	7	7	6	6	0.69	Phosphoglucosamine mutase OS=Lactococcus lactis subsp. lactis (strain IL1403) OX=272623 GN=glmM PE=3 SV=1
13	1	lactococcus	Q9CI64	14120	9670	225	225	6	6	11.60	DNA-binding protein HU OS=Lactococcus lactis subsp. lactis (strain IL1403) OX=272623 GN=hup PE=1 SV=1
301	1	lactococcus	Q9CGE3	255	40334	8	8	6	6	0.87	Bifunctional protein FolKE OS=Lactococcus lactis subsp. lactis (strain IL1403) OX=272623 GN=folKE PE=3 SV=1
36	1	lactococcus	P37283	4654	10214	95	95	6	6	35.67	10 kDa chaperonin OS=Lactococcus lactis subsp. lactis (strain IL1403) OX=272623 GN=groS PE=3 SV=1
326	1	lactococcus	Q9CFH4	219	93304	6	6	6	6	0.31	Probable phosphoketolase OS=Lactococcus lactis subsp. lactis (strain IL1403) OX=272623 GN=LL1502 PE=3 SV=1
255	1	lactococcus	Q9CIM6	381	34380	9	9	6	6	01.08	Ferrichrome ABC transporter substrate binding protein OS=Lactococcus lactis subsp. lactis (strain IL1403) OX=272623 GN=fhuD PE=4 SV=1
299	1	lactococcus	Q9CIR4	256	26428	8	8	6	6	1.59	TsaD domain-containing protein OS=Lactococcus lactis subsp. lactis (strain IL1403) OX=272623 GN=ycjB PE=4 SV=1
99	1	lactococcus	Q9CHZ8	1854	28260	25	25	6	6	1.43	Phosphomannomutase OS=Lactococcus lactis subsp. lactis (strain IL1403) OX=272623 GN=yfgH PE=3 SV=1
304	1	lactococcus	Q9CEJ0	254	51006	8	8	6	6	0.64	Cysteine--tRNA ligase OS=Lactococcus lactis subsp. lactis (strain IL1403) OX=272623 GN=cysS PE=3 SV=1
207	1	lactococcus	Q9CF06	512	25833	10	10	6	6	1.64	Two-component system regulator OS=Lactococcus lactis subsp. lactis (strain IL1403) OX=272623 GN=llrF PE=4 SV=1
314	1	lactococcus	Q9CGB8	236	58771	6	6	6	6	0.54	Uncharacterized protein OS=Lactococcus lactis subsp. lactis (strain IL1403) OX=272623 GN=yljF PE=4 SV=1
218	1	lactococcus	Q9CEH7	477	34931	9	9	6	6	01.06	Uncharacterized protein OS=Lactococcus lactis subsp. lactis (strain IL1403) OX=272623 GN=ytdB PE=4 SV=1
158	1	lactococcus	Q9CEE3	715	23608	16	16	6	6	2.44	Guanylate kinase OS=Lactococcus lactis subsp. lactis (strain IL1403) OX=272623 GN=gmk PE=3 SV=1
267	1	lactococcus	Q9CEB5	348	87280	8	8	6	6	0.34	Phenylalanine--tRNA ligase beta subunit OS=Lactococcus lactis subsp. lactis (strain IL1403) OX=272623 GN=pheT PE=3 SV=1
203	1	lactococcus	Q9CHF6	527	42631	12	12	6	6	1.00	3-oxoacyl-[acyl-carrier-protein] synthase 2 OS=Lactococcus lactis subsp. lactis (strain IL1403) OX=272623 GN=fabF PE=3 SV=1
244	1	lactococcus	Q9CHF2	409	31934	12	12	6	6	1.51	Acetyl-coenzyme A carboxylase carboxyl transferase subunit beta OS=Lactococcus lactis subsp. lactis (strain IL1403) OX=272623 GN=accD PE=3 SV=1
151	1	lactococcus	Q9CGW8	784	37683	14	14	6	6	0.95	Ribonucleoside-diphosphate reductase OS=Lactococcus lactis subsp. lactis (strain IL1403) OX=272623 GN=nrdF PE=3 SV=1

276	1	lactococcus	Q9CIN7	322	31145	9	9	6	6	1.56	Lipoprotein OS=Lactococcus lactis subsp. lactis (strain IL1403) OX=272623 GN=plpB PE=3 SV=1
210	1	lactococcus	Q9CJC4	497	46241	9	9	6	6	0.73	O-acetylhomoserine sulfhydrylase OS=Lactococcus lactis subsp. lactis (strain IL1403) OX=272623 GN=cysD PE=3 SV=1
224	1	lactococcus	Q9CDX9	458	12383	9	9	6	6	6.29	50S ribosomal protein L18 OS=Lactococcus lactis subsp. lactis (strain IL1403) OX=272623 GN=rplR PE=3 SV=1
164	1	lactococcus	Q9CEW9	689	24507	12	12	6	6	1.78	Phosphate-specific transport system accessory protein PhoU OS=Lactococcus lactis subsp. lactis (strain IL1403) OX=272623 GN=phoU PE=3 SV=1
253	1	lactococcus	Q9CFJ6	385	28403	8	8	6	6	1.81	Oxidoreductase OS=Lactococcus lactis subsp. lactis (strain IL1403) OX=272623 GN=ypaI PE=3 SV=1
200	1	lactococcus	Q9CFW9	539	26181	10	10	6	6	1.61	Orotidine 5'-phosphate decarboxylase OS=Lactococcus lactis subsp. lactis (strain IL1403) OX=272623 GN=pyrF PE=3 SV=1
239	1	lactococcus	Q9CDP9	428	30307	7	7	6	6	1.29	NAD(P)-bd_dom domain-containing protein OS=Lactococcus lactis subsp. lactis (strain IL1403) OX=272623 GN=yweD PE=4 SV=1
149	1	lactococcus	Q9CHG0	807	34868	13	13	6	6	01.06	3-oxoacyl-[acyl-carrier-protein] synthase 3 OS=Lactococcus lactis subsp. lactis (strain IL1403) OX=272623 GN=fabH PE=3 SV=1
268	1	lactococcus	Q9CF20	346	36267	10	10	6	6	1.00	Oxidoreductase OS=Lactococcus lactis subsp. lactis (strain IL1403) OX=272623 GN=yrbA PE=4 SV=1
208	1	lactococcus	Q9CFW5	504	43118	11	11	6	6	0.80	Band_7_1 domain-containing protein OS=Lactococcus lactis subsp. lactis (strain IL1403) OX=272623 GN=yniH PE=4 SV=1
226	1	lactococcus	Q9CHF1	456	28529	12	12	6	6	1.41	Acetyl-CoA carboxytransferase OS=Lactococcus lactis subsp. lactis (strain IL1403) OX=272623 GN=accA PE=4 SV=1
160	1	lactococcus	Q9CIV8	712	36265	17	17	6	6	1.00	PTS-dependent dihydroxyacetone kinase, dihydroxyacetone-binding subunit DhaK OS=Lactococcus lactis subsp. lactis (strain IL1403) OX=272623 GN=dhaK PE=1 SV=2
282	1	lactococcus	Q9CEN1	300	53939	7	7	6	6	0.60	UDP-N-acetylmuramyl-tripeptide synthetase OS=Lactococcus lactis subsp. lactis (strain IL1403) OX=272623 GN=murE PE=3 SV=1
205	1	lactococcus	Q9CG46	516	27417	11	11	6	6	1.50	Hydroxyethylthiazole kinase OS=Lactococcus lactis subsp. lactis (strain IL1403) OX=272623 GN=thiM PE=3 SV=1
245	1	lactococcus	Q9CED2	405	74241	8	8	6	6	0.41	Threonine--tRNA ligase OS=Lactococcus lactis subsp. lactis (strain IL1403) OX=272623 GN=thrS PE=3 SV=1
211	1	lactococcus	Q9CE04	495	29412	12	12	6	6	1.35	Uncharacterized protein OS=Lactococcus lactis subsp. lactis (strain IL1403) OX=272623 GN=yvdB PE=4 SV=1
276	2	lactococcus	Q9CIN8	297	31321	7	7	6	6	1.23	Lipoprotein OS=Lactococcus lactis subsp. lactis (strain IL1403) OX=272623 GN=plpA PE=3 SV=1
199	1	lactococcus	Q9CEM5	541	34048	9	9	6	6	01.09	Probable manganese-dependent inorganic pyrophosphatase OS=Lactococcus lactis subsp. lactis (strain IL1403) OX=272623 GN=ppaC PE=3 SV=1
230	1	lactococcus	Q9CE49	452	15069	12	12	6	6	4.18	HIT domain-containing protein OS=Lactococcus lactis subsp. lactis (strain IL1403) OX=272623 GN=yuhI PE=4 SV=1
201	1	lactococcus	Q9CHE9	531	8544	11	11	6	6	26.74	UPF0337 protein YhjA OS=Lactococcus lactis subsp. lactis (strain IL1403) OX=272623 GN=yhjA PE=3 SV=1
248	1	lactococcus	Q9CGM0	395	52260	8	8	6	6	0.62	HD domain-containing protein OS=Lactococcus lactis subsp. lactis (strain IL1403) OX=272623 GN=ykiG PE=4 SV=1
249	1	lactococcus	Q9CF73	391	45490	7	7	6	6	0.74	Gamma-glutamyl phosphate reductase OS=Lactococcus lactis subsp. lactis (strain IL1403) OX=272623 GN=proA PE=3 SV=1
399	1	lactococcus	Q9CJ29	138	50677	5	5	5	5	0.51	Metallophos domain-containing protein OS=Lactococcus lactis subsp. lactis (strain IL1403) OX=272623 GN=ybiB PE=3 SV=1
19	1	lactococcus	Q9CE46	7452	14696	95	95	5	5	8.56	50S ribosomal protein L11 OS=Lactococcus lactis subsp. lactis (strain IL1403) OX=272623 GN=rplK PE=3 SV=1
338	1	lactococcus	Q9CF31	205	72305	6	6	5	5	0.34	Mid-cell-anchored protein Z OS=Lactococcus lactis subsp. lactis (strain IL1403) OX=272623 GN=yqjD PE=3 SV=1
75	1	lactococcus	Q9CDM8	2458	11242	46	46	5	5	5.23	30S ribosomal protein S6 OS=Lactococcus lactis subsp. lactis (strain IL1403) OX=272623 GN=rpsF PE=3 SV=1
373	1	lactococcus	Q02146	162	75365	5	5	5	5	0.32	Ribonuclease R 2 OS=Lactococcus lactis subsp. lactis (strain IL1403) OX=272623 GN=rnr2 PE=3 SV=3
159	1	lactococcus	Q9CJ10	714	22343	14	14	5	5	02.07	dTDP-4-keto-6-deoxyglucose-3,5-epimerase OS=Lactococcus lactis subsp. lactis (strain IL1403) OX=272623 GN=cpsM PE=4 SV=1
290	1	lactococcus	Q9CHD6	283	23061	7	7	5	5	1.47	NAD(P)-bd_dom domain-containing protein OS=Lactococcus lactis subsp. lactis (strain IL1403) OX=272623 GN=yiaD PE=4 SV=1
53	1	lactococcus	Q9CGM2	3675	13660	40	40	5	5	3.53	Fe-S_biosyn domain-containing protein OS=Lactococcus lactis subsp. lactis (strain IL1403) OX=272623 GN=ykiE PE=4 SV=1

362	1	lactococcus	Q9CIS2	175	23150	6	6	5	5	1.46	30S ribosomal protein S4 OS=Lactococcus lactis subsp. lactis (strain IL1403) OX=272623 GN=rpsD PE=3 SV=1
172	1	lactococcus	Q9CEK7	656	43200	8	8	5	5	0.79	Aminotransferase OS=Lactococcus lactis subsp. lactis (strain IL1403) OX=272623 GN=aspB PE=3 SV=1
329	1	lactococcus	Q9CG54	216	25018	6	6	5	5	1.31	Metalloregulator OS=Lactococcus lactis subsp. lactis (strain IL1403) OX=272623 GN=ymiA PE=3 SV=1
112	1	lactococcus	Q9CE86	1372	31883	21	21	5	5	1.51	33 kDa chaperonin OS=Lactococcus lactis subsp. lactis (strain IL1403) OX=272623 GN=hslO PE=3 SV=1
364	1	lactococcus	Q9CHX1	173	24249	5	5	5	5	1.37	NADPH-flavin oxidoreductase OS=Lactococcus lactis subsp. lactis (strain IL1403) OX=272623 GN=yfiJ PE=3 SV=1
261	1	lactococcus	Q9CES5	363	78328	8	8	5	5	0.38	Glutamine ABC transporter permease and substrate binding protein protein OS=Lactococcus lactis subsp. lactis (strain IL1403) OX=272623 GN=glnP PE=1 SV=1
294	1	lactococcus	Q9CEN0	272	36123	6	6	5	5	0.79	Alcohol dehydrogenase OS=Lactococcus lactis subsp. lactis (strain IL1403) OX=272623 GN=adhA PE=3 SV=1
68	1	lactococcus	Q9CF37	2784	11803	42	42	5	5	31.82	Thioredoxin OS=Lactococcus lactis subsp. lactis (strain IL1403) OX=272623 GN=trxA PE=3 SV=1
344	1	lactococcus	Q9CEB6	198	35813	5	5	5	5	0.80	3D domain-containing protein OS=Lactococcus lactis subsp. lactis (strain IL1403) OX=272623 GN=yuaE PE=4 SV=1
191	1	lactococcus	Q9CFA9	558	42733	13	13	5	5	0.64	Hydroxymethylglutaryl-CoA synthase OS=Lactococcus lactis subsp. lactis (strain IL1403) OX=272623 GN=hmcM PE=3 SV=1
309	1	lactococcus	Q9CJ08	246	39483	6	6	5	5	0.70	dTDP-glucose 4,6-dehydratase OS=Lactococcus lactis subsp. lactis (strain IL1403) OX=272623 GN=rmlB PE=3 SV=1
129	1	lactococcus	Q9CIF0	1093	11349	21	21	5	5	17.15	PTS system cellobiose-specific EIIB component OS=Lactococcus lactis subsp. lactis (strain IL1403) OX=272623 GN=ptcB PE=1 SV=1
346	1	lactococcus	Q9CGY0	194	35767	5	5	5	5	0.80	Putative gluconeogenesis factor OS=Lactococcus lactis subsp. lactis (strain IL1403) OX=272623 GN=yjiF PE=3 SV=1
214	1	lactococcus	Q9CGH1	489	24473	10	10	5	5	1.35	Uncharacterized protein OS=Lactococcus lactis subsp. lactis (strain IL1403) OX=272623 GN=yldE PE=4 SV=1
287	1	lactococcus	Q48662	291	59572	6	6	5	5	0.42	Malolactic enzyme OS=Lactococcus lactis subsp. lactis (strain IL1403) OX=272623 GN=mleS PE=3 SV=2
139	1	lactococcus	Q9CE53	931	22523	23	23	5	5	1.52	Copper homeostasis protein CutC OS=Lactococcus lactis subsp. lactis (strain IL1403) OX=272623 GN=yuhE PE=3 SV=1
311	1	lactococcus	Q9CF39	243	26049	6	6	5	5	1.23	3-dehydroquinate dehydratase OS=Lactococcus lactis subsp. lactis (strain IL1403) OX=272623 GN=aroD PE=3 SV=1
145	1	lactococcus	Q9CIL9	855	20728	16	16	5	5	2.33	Alkyl hydroperoxide reductase C OS=Lactococcus lactis subsp. lactis (strain IL1403) OX=272623 GN=ahpC PE=3 SV=1
295	1	lactococcus	Q9CES0	272	50879	6	6	5	5	0.51	ATP synthase subunit beta OS=Lactococcus lactis subsp. lactis (strain IL1403) OX=272623 GN=atpD PE=3 SV=1
125	1	lactococcus	Q9CF28	1132	14970	15	15	5	5	4.24	Uncharacterized protein OS=Lactococcus lactis subsp. lactis (strain IL1403) OX=272623 GN=yraB PE=4 SV=1
310	1	lactococcus	Q9CEJ7	244	20545	9	9	5	5	3.14	Translation initiation factor IF-3 OS=Lactococcus lactis subsp. lactis (strain IL1403) OX=272623 GN=infC PE=3 SV=1
254	1	lactococcus	Q9CFF4	383	36254	8	8	5	5	0.78	Phosphoribosylformylglycinamidine cyclo-ligase OS=Lactococcus lactis subsp. lactis (strain IL1403) OX=272623 GN=purM PE=3 SV=1
283	1	lactococcus	Q9CF80	300	39817	8	8	5	5	0.88	Carbamoyl-phosphate synthase small chain OS=Lactococcus lactis subsp. lactis (strain IL1403) OX=272623 GN=carA PE=3 SV=1
185	1	lactococcus	Q9CHF9	570	8396	15	15	5	5	10.06	Acyl carrier protein OS=Lactococcus lactis subsp. lactis (strain IL1403) OX=272623 GN=acpP PE=3 SV=1
285	1	lactococcus	Q9CE96	299	21380	6	6	5	5	1.65	Uncharacterized protein OS=Lactococcus lactis subsp. lactis (strain IL1403) OX=272623 GN=yucF PE=4 SV=1
217	1	lactococcus	Q9CEF6	478	10337	12	12	5	5	6.25	30S ribosomal protein S15 OS=Lactococcus lactis subsp. lactis (strain IL1403) OX=272623 GN=rpsO PE=3 SV=1
165	1	lactococcus	Q9CHN6	685	20640	15	15	5	5	2.36	Elongation factor P OS=Lactococcus lactis subsp. lactis (strain IL1403) OX=272623 GN=efp PE=3 SV=1
273	1	lactococcus	Q9CHT4	332	21374	10	10	5	5	1.65	Probable DNA-directed RNA polymerase subunit delta OS=Lactococcus lactis subsp. lactis (strain IL1403) OX=272623 GN=rpoE PE=3 SV=1
206	1	lactococcus	Q9CDH3	512	19496	9	9	5	5	1.90	DUF536 domain-containing protein OS=Lactococcus lactis subsp. lactis (strain IL1403) OX=272623 GN=yxdB PE=4 SV=1
246	1	lactococcus	Q9CFC0	405	28473	10	10	5	5	01.08	4-hydroxy-tetrahydrodipicolinate reductase OS=Lactococcus lactis subsp. lactis (strain IL1403) OX=272623 GN=dapB PE=3 SV=1

150	1	lactococcus	Q9CH83	789	8959	12	12	5	5	8.60	Exodeoxyribonuclease 7 small subunit OS=Lactococcus lactis subsp. lactis (strain IL1403) OX=272623 GN=xseB PE=3 SV=1
262	1	lactococcus	Q9CF85	360	9331	8	8	5	5	7.88	50S ribosomal protein L31 type B OS=Lactococcus lactis subsp. lactis (strain IL1403) OX=272623 GN=rpmE2 PE=3 SV=1
193	1	lactococcus	Q9CEK2	552	37608	9	9	5	5	0.75	Penicillin acylase OS=Lactococcus lactis subsp. lactis (strain IL1403) OX=272623 GN=pacB PE=3 SV=1
229	1	lactococcus	Q9CHN7	453	39567	11	11	5	5	0.70	Aminopeptidase P OS=Lactococcus lactis subsp. lactis (strain IL1403) OX=272623 GN=pepP PE=3 SV=1
161	1	lactococcus	Q9CDK4	708	31394	13	13	5	5	1.23	3-hydroxyisobutyrate dehydrogenase OS=Lactococcus lactis subsp. lactis (strain IL1403) OX=272623 GN=ywjF PE=4 SV=1
277	1	lactococcus	Q9CGM3	319	28542	7	7	5	5	01.08	GTP cyclohydrolase 1 type 2 homolog OS=Lactococcus lactis subsp. lactis (strain IL1403) OX=272623 GN=ykiD PE=3 SV=1
202	1	lactococcus	Q9CEV9	527	33857	7	7	5	5	0.86	Foldase protein PrsA OS=Lactococcus lactis subsp. lactis (strain IL1403) OX=272623 GN=prsA PE=1 SV=1
250	1	lactococcus	Q9CEC9	389	23262	8	8	5	5	1.45	Uracil phosphoribosyltransferase OS=Lactococcus lactis subsp. lactis (strain IL1403) OX=272623 GN=upp PE=3 SV=1
186	1	lactococcus	Q9CEF4	570	24211	11	11	5	5	1.37	Protein serine/threonine phosphatase OS=Lactococcus lactis subsp. lactis (strain IL1403) OX=272623 GN=pppL PE=4 SV=1
278	1	lactococcus	Q9CEE4	311	13426	9	9	5	5	3.64	DNA-directed RNA polymerase subunit omega OS=Lactococcus lactis subsp. lactis (strain IL1403) OX=272623 GN=rpoZ PE=3 SV=1
198	1	lactococcus	Q9CHI6	541	16365	12	12	5	5	2.56	50S ribosomal protein L9 OS=Lactococcus lactis subsp. lactis (strain IL1403) OX=272623 GN=rplI PE=3 SV=1
204	1	lactococcus	Q9CE47	525	24035	12	12	5	5	1.38	50S ribosomal protein L1 OS=Lactococcus lactis subsp. lactis (strain IL1403) OX=272623 GN=rplA PE=3 SV=1
433	1	lactococcus	Q9CIV3	115	24332	4	4	4	4	0.99	Transcriptional regulator OS=Lactococcus lactis subsp. lactis (strain IL1403) OX=272623 GN=ycfA PE=4 SV=1
86	1	lactococcus	Q9CFE6	2254	9735	45	45	4	4	11.26	Phosphoribosylformylglycinamidine synthase subunit PurS OS=Lactococcus lactis subsp. lactis (strain IL1403) OX=272623 GN=yphF PE=3 SV=1
409	1	lactococcus	Q9CHB2	129	30640	4	4	4	4	0.73	Non-heme chloride peroxidase OS=Lactococcus lactis subsp. lactis (strain IL1403) OX=272623 GN=cpo PE=4 SV=1
104	1	lactococcus	Q9CF19	1704	20548	24	24	4	4	1.25	Cpl-7 domain-containing protein OS=Lactococcus lactis subsp. lactis (strain IL1403) OX=272623 GN=yrbB PE=4 SV=1
443	1	lactococcus	Q9CGF3	108	28162	4	4	4	4	0.81	Acid sugar phosphatase OS=Lactococcus lactis subsp. lactis (strain IL1403) OX=272623 GN=ylfH PE=3 SV=1
238	1	lactococcus	Q9CDX0	429	7854	11	11	4	4	6.78	50S ribosomal protein L29 OS=Lactococcus lactis subsp. lactis (strain IL1403) OX=272623 GN=rpmC PE=3 SV=1
319	1	lactococcus	Q9CJ43	225	52039	4	4	4	4	0.38	Glutamyl-tRNA(Gln) amidotransferase subunit A OS=Lactococcus lactis subsp. lactis (strain IL1403) OX=272623 GN=gatA PE=3 SV=1
147	1	lactococcus	Q9CJ64	835	10163	12	12	4	4	3.96	UPF0297 protein YbeA OS=Lactococcus lactis subsp. lactis (strain IL1403) OX=272623 GN=ybeA PE=3 SV=1
448	1	lactococcus	Q9CEI2	105	41108	4	4	4	4	0.51	Pyridoxal-phosphate dependent aminotransferase NifS OS=Lactococcus lactis subsp. lactis (strain IL1403) OX=272623 GN=nifS PE=4 SV=1
225	1	lactococcus	Q9CEP5	457	45333	7	7	4	4	0.59	Cysteine desulfurase OS=Lactococcus lactis subsp. lactis (strain IL1403) OX=272623 GN=yseI PE=3 SV=1
385	1	lactococcus	Q9CEB4	149	39432	5	5	4	4	0.53	Phenylalanine--tRNA ligase alpha subunit OS=Lactococcus lactis subsp. lactis (strain IL1403) OX=272623 GN=pheS PE=3 SV=1
173	1	lactococcus	Q9CEI3	655	16148	12	12	4	4	1.80	Universal stress protein OS=Lactococcus lactis subsp. lactis (strain IL1403) OX=272623 GN=ytcD PE=3 SV=1
446	1	lactococcus	Q9CE73	107	47273	4	4	4	4	0.43	Protease OS=Lactococcus lactis subsp. lactis (strain IL1403) OX=272623 GN=yueE PE=4 SV=1
260	1	lactococcus	Q9CGL7	367	11430	8	8	4	4	3.20	50S ribosomal protein L21 OS=Lactococcus lactis subsp. lactis (strain IL1403) OX=272623 GN=rplU PE=3 SV=1
316	1	lactococcus	P0A4A9	226	13483	7	7	4	4	3.64	30S ribosomal protein S13 OS=Lactococcus lactis subsp. lactis (strain IL1403) OX=272623 GN=rpsM PE=3 SV=1
212	1	lactococcus	Q9CHF7	494	25582	8	8	4	4	1.27	3-oxoacyl-[acyl-carrier-protein] reductase OS=Lactococcus lactis subsp. lactis (strain IL1403) OX=272623 GN=fabG1 PE=3 SV=1
402	1	lactococcus	Q9CEU5	136	8348	5	5	4	4	06.01	30S ribosomal protein S20 OS=Lactococcus lactis subsp. lactis (strain IL1403) OX=272623 GN=rpsT PE=3 SV=1
242	1	lactococcus	Q9CI68	420	30586	9	9	4	4	0.98	DegV domain-containing protein YejH OS=Lactococcus lactis subsp. lactis (strain IL1403) OX=272623 GN=yejH PE=3 SV=2

324	1	lactococcus	Q9CIS6	222	23348	5	5	4	4	01.05	Uncharacterized protein OS=Lactococcus lactis subsp. lactis (strain IL1403) OX=272623 GN=ychG PE=4 SV=1
168	1	lactococcus	Q9CFB2	671	10275	13	13	4	4	3.88	30S ribosomal protein S16 OS=Lactococcus lactis subsp. lactis (strain IL1403) OX=272623 GN=rpsP PE=3 SV=1
357	1	lactococcus	Q9CIL7	179	77851	4	4	4	4	0.24	Penicillin-binding protein 2B OS=Lactococcus lactis subsp. lactis (strain IL1403) OX=272623 GN=pbp2B PE=3 SV=1
271	1	lactococcus	Q9CEI5	334	39952	6	6	4	4	0.52	Uncharacterized protein OS=Lactococcus lactis subsp. lactis (strain IL1403) OX=272623 GN=ytcC PE=4 SV=1
298	1	lactococcus	Q9CDX4	258	19994	6	6	4	4	1.30	50S ribosomal protein L5 OS=Lactococcus lactis subsp. lactis (strain IL1403) OX=272623 GN=rplE PE=3 SV=1
257	1	lactococcus	P0A3Z9	373	30388	7	7	4	4	0.74	Pur operon repressor OS=Lactococcus lactis subsp. lactis (strain IL1403) OX=272623 GN=purR PE=3 SV=1
411	1	lactococcus	Q9CHJ6	129	34642	4	4	4	4	0.62	Uncharacterized protein OS=Lactococcus lactis subsp. lactis (strain IL1403) OX=272623 GN=yheB PE=4 SV=1
279	1	lactococcus	Q9CEH3	307	25682	5	5	4	4	0.92	Pyridoxal phosphate homeostasis protein OS=Lactococcus lactis subsp. lactis (strain IL1403) OX=272623 GN=ytdF PE=3 SV=1
349	1	lactococcus	Q9CFX1	187	26875	6	6	4	4	0.86	Ribosomal RNA small subunit methyltransferase G OS=Lactococcus lactis subsp. lactis (strain IL1403) OX=272623 GN=rsmG PE=3 SV=1
259	1	lactococcus	Q9CGU6	368	16778	7	7	4	4	1.69	6,7-dimethyl-8-ribityllumazine synthase OS=Lactococcus lactis subsp. lactis (strain IL1403) OX=272623 GN=ribH PE=3 SV=1
380	1	lactococcus	Q9CI23	153	71891	4	4	4	4	0.26	DD-transpeptidase OS=Lactococcus lactis subsp. lactis (strain IL1403) OX=272623 GN=ponA PE=4 SV=1
305	1	lactococcus	Q9CJF3	252	7604	6	6	4	4	7.23	Prophage ps1 protein 18 OS=Lactococcus lactis subsp. lactis (strain IL1403) OX=272623 GN=ps118 PE=4 SV=1
408	1	lactococcus	Q9CI20	130	14950	4	4	4	4	02.02	Global transcriptional regulator Spx 1 OS=Lactococcus lactis subsp. lactis (strain IL1403) OX=272623 GN=spx1 PE=3 SV=1
336	1	lactococcus	Q9CH36	208	72551	5	5	4	4	0.34	DNA gyrase subunit B OS=Lactococcus lactis subsp. lactis (strain IL1403) OX=272623 GN=gyrB PE=3 SV=1
359	1	lactococcus	Q9CDX8	178	19245	5	5	4	4	1.38	50S ribosomal protein L6 OS=Lactococcus lactis subsp. lactis (strain IL1403) OX=272623 GN=rplF PE=3 SV=1
291	1	lactococcus	Q9CF86	275	20461	8	8	4	4	1.26	Uncharacterized protein OS=Lactococcus lactis subsp. lactis (strain IL1403) OX=272623 GN=yqdA PE=4 SV=1
412	1	lactococcus	Q9CFE8	129	80059	5	5	4	4	0.24	Phosphoribosylformylglycinamidine synthase subunit PurL OS=Lactococcus lactis subsp. lactis (strain IL1403) OX=272623 GN=purL PE=3 SV=1
340	1	lactococcus	Q9CJ34	205	40698	5	5	4	4	0.51	N-acetyltransferase domain-containing protein OS=Lactococcus lactis subsp. lactis (strain IL1403) OX=272623 GN=ybhD PE=4 SV=1
388	1	lactococcus	Q9CGE2	146	40505	4	4	4	4	0.51	Dihydropteroate pyrophosphorylase OS=Lactococcus lactis subsp. lactis (strain IL1403) OX=272623 GN=folP PE=3 SV=1
312	1	lactococcus	Q9CJ97	242	34778	6	6	4	4	0.83	Ribosomal protein L11 methyltransferase OS=Lactococcus lactis subsp. lactis (strain IL1403) OX=272623 GN=prmA PE=3 SV=1
363	1	lactococcus	Q9CE09	174	22096	4	4	4	4	1.13	Uncharacterized protein OS=Lactococcus lactis subsp. lactis (strain IL1403) OX=272623 GN=yvcA PE=4 SV=1
325	1	lactococcus	Q9CHY3	220	10875	6	6	4	4	3.51	ABM domain-containing protein OS=Lactococcus lactis subsp. lactis (strain IL1403) OX=272623 GN=yfhK PE=4 SV=1
377	1	lactococcus	Q9CDI7	155	24457	4	4	4	4	0.98	Ribose-5-phosphate isomerase A OS=Lactococcus lactis subsp. lactis (strain IL1403) OX=272623 GN=rpiA PE=3 SV=1
293	1	lactococcus	Q9CFX8	272	44283	7	7	4	4	0.46	Uncharacterized protein OS=Lactococcus lactis subsp. lactis (strain IL1403) OX=272623 GN=ynhC PE=3 SV=1
386	1	lactococcus	Q9CH08	147	11934	4	4	4	4	2.94	UPF0145 protein YjfJ OS=Lactococcus lactis subsp. lactis (strain IL1403) OX=272623 GN=yjfJ PE=3 SV=1
331	1	lactococcus	Q9CE63	213	56658	5	5	4	4	0.35	Galactose-1-phosphate uridylyltransferase OS=Lactococcus lactis subsp. lactis (strain IL1403) OX=272623 GN=galT PE=3 SV=1
381	1	lactococcus	Q9CFX2	152	35654	4	4	4	4	0.60	Heptaprenyl diphosphate synthase component II OS=Lactococcus lactis subsp. lactis (strain IL1403) OX=272623 GN=ispB PE=3 SV=1
303	1	lactococcus	P35514	254	41014	5	5	4	4	0.51	Chaperone protein DnaJ OS=Lactococcus lactis subsp. lactis (strain IL1403) OX=272623 GN=dnaJ PE=3 SV=2
376	1	lactococcus	Q9CFX5	158	34851	4	4	4	4	0.62	UTP--glucose-1-phosphate uridylyltransferase OS=Lactococcus lactis subsp. lactis (strain IL1403) OX=272623 GN=hasC PE=3 SV=1

321	1	lactococcus	Q59487	225	19716	6	6	4	4	1.32	Dihydrofolate reductase OS=Lactococcus lactis subsp. lactis (strain IL1403) OX=272623 GN=folA PE=3 SV=2
300	1	lactococcus	P71447	256	24194	6	6	4	4	0.99	Beta-phosphoglucomutase OS=Lactococcus lactis subsp. lactis (strain IL1403) OX=272623 GN=pgmB PE=1 SV=2
320	1	lactococcus	Q9CG20	225	54168	5	5	4	4	0.37	Glutamate decarboxylase OS=Lactococcus lactis subsp. lactis (strain IL1403) OX=272623 GN=gadB PE=1 SV=1
306	1	lactococcus	Q9CEB9	251	23527	7	7	4	4	01.03	Ribulose-phosphate 3-epimerase OS=Lactococcus lactis subsp. lactis (strain IL1403) OX=272623 GN=rpe PE=3 SV=1
322	1	lactococcus	Q9CHY2	224	24525	5	5	4	4	0.98	Uncharacterized protein OS=Lactococcus lactis subsp. lactis (strain IL1403) OX=272623 GN=yfhL PE=4 SV=1
296	1	lactococcus	Q9CDF8	269	47102	5	5	4	4	0.43	Serine-type D-Ala-D-Ala carboxypeptidase OS=Lactococcus lactis subsp. lactis (strain IL1403) OX=272623 GN=dacA PE=3 SV=1
192	1	lactococcus	Q9CIE9	555	12806	8	8	3	3	1.63	PTS system cellobiose-specific EIIA component OS=Lactococcus lactis subsp. lactis (strain IL1403) OX=272623 GN=ptcA PE=2 SV=1
221	1	lactococcus	Q9CIQ2	471	17518	7	7	3	3	01.03	Thiol peroxidase OS=Lactococcus lactis subsp. lactis (strain IL1403) OX=272623 GN=tpx PE=4 SV=1
499	1	lactococcus	Q9CFU9	79	96248	3	3	3	3	0.14	Calcium-transporting ATPase 1 OS=Lactococcus lactis subsp. lactis (strain IL1403) OX=272623 GN=yoaB PE=1 SV=1
243	1	lactococcus	Q9CGI9	411	24804	8	8	3	3	0.66	Alpha-acetolactate decarboxylase OS=Lactococcus lactis subsp. lactis (strain IL1403) OX=272623 GN=aldC PE=3 SV=1
398	1	lactococcus	Q9CG64	138	55410	3	3	3	3	0.26	Glycerol kinase OS=Lactococcus lactis subsp. lactis (strain IL1403) OX=272623 GN=glpK PE=3 SV=1
270	1	lactococcus	Q9CI10	339	13213	6	6	3	3	1.55	Uncharacterized protein OS=Lactococcus lactis subsp. lactis (strain IL1403) OX=272623 GN=yffA PE=4 SV=1
466	1	lactococcus	Q9CDG0	94	17672	3	3	3	3	01.03	30S ribosomal protein S7 OS=Lactococcus lactis subsp. lactis (strain IL1403) OX=272623 GN=rpsG PE=3 SV=1
264	1	lactococcus	Q9CEP3	350	28407	8	8	3	3	01.09	ABC transporter ATP-binding protein OS=Lactococcus lactis subsp. lactis (strain IL1403) OX=272623 GN=ysfB PE=3 SV=1
347	1	lactococcus	Q9CFB3	192	9012	4	4	3	3	2.88	UPF0109 protein L9737 OS=Lactococcus lactis subsp. lactis (strain IL1403) OX=272623 GN=yqbA PE=3 SV=1
252	1	lactococcus	Q9CHG1	387	16890	9	9	3	3	1.67	Transcriptional regulator OS=Lactococcus lactis subsp. lactis (strain IL1403) OX=272623 GN=rmaG PE=4 SV=1
516	1	lactococcus	Q9CHT8	73	34106	3	3	3	3	0.45	Ribonuclease Z OS=Lactococcus lactis subsp. lactis (strain IL1403) OX=272623 GN=rnz PE=3 SV=1
263	1	lactococcus	Q9CEE1	356	26574	6	6	3	3	0.61	Glucosamine_iso domain-containing protein OS=Lactococcus lactis subsp. lactis (strain IL1403) OX=272623 GN=ytgG PE=4 SV=1
365	1	lactococcus	Q9CGD1	171	40590	3	3	3	3	0.36	Spermidine/putrescine ABC transporter substrate binding protein OS=Lactococcus lactis subsp. lactis (strain IL1403) OX=272623 GN=potD PE=3 SV=1
258	1	lactococcus	Q9CDI9	369	31429	7	7	3	3	0.71	Cell shape-determining protein MreC OS=Lactococcus lactis subsp. lactis (strain IL1403) OX=272623 GN=mreC PE=3 SV=1
438	1	lactococcus	Q9CEY1	111	24533	3	3	3	3	0.67	Cytidylate kinase OS=Lactococcus lactis subsp. lactis (strain IL1403) OX=272623 GN=cmk PE=3 SV=1
266	1	lactococcus	Q9CDX7	348	14676	6	6	3	3	02.09	30S ribosomal protein S8 OS=Lactococcus lactis subsp. lactis (strain IL1403) OX=272623 GN=rpsH PE=3 SV=1
318	1	lactococcus	Q9CDW6	226	10564	5	5	3	3	3.72	30S ribosomal protein S19 OS=Lactococcus lactis subsp. lactis (strain IL1403) OX=272623 GN=rpsS PE=3 SV=1
510	1	lactococcus	Q9CJ31	75	54686	3	3	3	3	0.26	Beta-glucosidase A OS=Lactococcus lactis subsp. lactis (strain IL1403) OX=272623 GN=bglS PE=3 SV=1
418	1	lactococcus	Q9CIQ7	125	37403	4	4	3	3	0.40	Uncharacterized protein OS=Lactococcus lactis subsp. lactis (strain IL1403) OX=272623 GN=ycjI PE=4 SV=1
479	1	lactococcus	P19368	87	32524	4	4	3	3	0.47	Thymidylate synthase OS=Lactococcus lactis subsp. lactis (strain IL1403) OX=272623 GN=thyA PE=3 SV=2
333	1	lactococcus	Q9CII4	210	34336	4	4	3	3	0.44	L-lactate dehydrogenase 2 OS=Lactococcus lactis subsp. lactis (strain IL1403) OX=272623 GN=ldh2 PE=3 SV=1
517	1	lactococcus	Q9CGN6	73	24359	3	3	3	3	0.67	Redox-sensing transcriptional repressor Rex OS=Lactococcus lactis subsp. lactis (strain IL1403) OX=272623 GN=rex PE=3 SV=2
360	1	lactococcus	Q9CFG3	178	24193	3	3	3	3	0.68	Transcriptional regulator OS=Lactococcus lactis subsp. lactis (strain IL1403) OX=272623 GN=ypfD PE=4 SV=1
427	1	lactococcus	Q9CDV3	118	81924	3	3	3	3	0.17	DD-transpeptidase OS=Lactococcus lactis subsp. lactis (strain IL1403) OX=272623 GN=pbp2A PE=4 SV=1

288	1	lactococcus	Q9CIS4	289	43165	6	6	3	3	0.34	N-acetyldiaminopimelate deacetylase OS=Lactococcus lactis subsp. lactis (strain IL1403) OX=272623 GN=yciA PE=3 SV=1
470	1	lactococcus	Q9CGY1	92	33616	3	3	3	3	0.45	Nucleotide-binding protein YjiE OS=Lactococcus lactis subsp. lactis (strain IL1403) OX=272623 GN=yjiE PE=3 SV=1
403	1	lactococcus	Q9CFX7	135	24380	4	4	3	3	0.67	Uncharacterized protein OS=Lactococcus lactis subsp. lactis (strain IL1403) OX=272623 GN=ynhD PE=4 SV=1
453	1	lactococcus	Q9CH33	102	17714	3	3	3	3	01.02	Transcriptional regulator OS=Lactococcus lactis subsp. lactis (strain IL1403) OX=272623 GN=rmaH PE=4 SV=1
337	1	lactococcus	Q9CIY2	207	11364	5	5	3	3	1.97	CRM domain-containing protein OS=Lactococcus lactis subsp. lactis (strain IL1403) OX=272623 GN=yccF PE=4 SV=1
493	1	lactococcus	Q9CH81	81	31334	3	3	3	3	0.49	Farnesyl diphosphate synthase OS=Lactococcus lactis subsp. lactis (strain IL1403) OX=272623 GN=ispA PE=3 SV=1
383	1	lactococcus	Q9CIL8	150	55338	4	4	3	3	0.26	Alkyl hydroperoxide reductase OS=Lactococcus lactis subsp. lactis (strain IL1403) OX=272623 GN=ahpF PE=3 SV=1
442	1	lactococcus	Q9CJD2	109	25328	3	3	3	3	0.64	HD domain-containing protein OS=Lactococcus lactis subsp. lactis (strain IL1403) OX=272623 GN=yagB PE=4 SV=1
307	1	lactococcus	Q9CEJ9	250	13657	5	5	3	3	1.48	50S ribosomal protein L20 OS=Lactococcus lactis subsp. lactis (strain IL1403) OX=272623 GN=rplT PE=3 SV=1
484	1	lactococcus	Q9CGJ9	85	9211	3	3	3	3	2.76	UPF0291 protein YlaC OS=Lactococcus lactis subsp. lactis (strain IL1403) OX=272623 GN=ylcA PE=3 SV=1
414	1	lactococcus	Q9CFA8	128	25604	3	3	3	3	0.63	Glucosamine-6-phosphate deaminase OS=Lactococcus lactis subsp. lactis (strain IL1403) OX=272623 GN=nagB PE=3 SV=1
449	1	lactococcus	Q9CEU9	105	25093	3	3	3	3	0.65	LUD_dom domain-containing protein OS=Lactococcus lactis subsp. lactis (strain IL1403) OX=272623 GN=yrjD PE=4 SV=1
327	1	lactococcus	Q9CIP0	219	17444	8	8	3	3	01.04	Cys-tRNA(Pro)/Cys-tRNA(Cys) deacylase OS=Lactococcus lactis subsp. lactis (strain IL1403) OX=272623 GN=ydbF PE=3 SV=1
480	1	lactococcus	Q7DAU1	86	29668	3	3	3	3	0.53	Lipase OS=Lactococcus lactis subsp. lactis (strain IL1403) OX=272623 GN=yscE PE=4 SV=1
367	1	lactococcus	Q9CIC6	169	31554	4	4	3	3	0.49	Prophage pi1 protein 03, transcriptional regulator OS=Lactococcus lactis subsp. lactis (strain IL1403) OX=272623 GN=pi103 PE=4 SV=1
430	1	lactococcus	Q9CDW8	115	24019	3	3	3	3	0.69	30S ribosomal protein S3 OS=Lactococcus lactis subsp. lactis (strain IL1403) OX=272623 GN=rpsC PE=3 SV=1
292	1	lactococcus	Q9CIX8	274	12845	5	5	3	3	1.61	Ribosomal silencing factor RsfS OS=Lactococcus lactis subsp. lactis (strain IL1403) OX=272623 GN=yccJ PE=3 SV=1
444	1	lactococcus	Q9CE80	108	66549	3	3	3	3	0.21	Aspartate--tRNA ligase OS=Lactococcus lactis subsp. lactis (strain IL1403) OX=272623 GN=aspS PE=3 SV=1
406	1	lactococcus	Q9CJ38	130	7119	4	4	3	3	4.46	Major cold shock protein OS=Lactococcus lactis subsp. lactis (strain IL1403) OX=272623 GN=cspE PE=4 SV=1
431	1	lactococcus	Q9CEH0	115	31563	3	3	3	3	0.49	DegV domain-containing protein YteA OS=Lactococcus lactis subsp. lactis (strain IL1403) OX=272623 GN=yteA PE=3 SV=1
351	1	lactococcus	Q9CDZ4	186	18525	4	4	3	3	0.96	N-acetyltransferase domain-containing protein OS=Lactococcus lactis subsp. lactis (strain IL1403) OX=272623 GN=yveC PE=4 SV=1
452	1	lactococcus	Q9CG35	102	28704	4	4	3	3	0.55	Pseudouridine synthase OS=Lactococcus lactis subsp. lactis (strain IL1403) OX=272623 GN=rluB PE=3 SV=1
382	1	lactococcus	Q9CDY0	151	17585	5	5	3	3	01.03	30S ribosomal protein S5 OS=Lactococcus lactis subsp. lactis (strain IL1403) OX=272623 GN=rpsE PE=3 SV=1
432	1	lactococcus	Q9CFC2	115	45844	3	3	3	3	0.32	CCA-adding enzyme OS=Lactococcus lactis subsp. lactis (strain IL1403) OX=272623 GN=cca PE=3 SV=2
313	1	lactococcus	Q9CID4	242	30997	5	5	3	3	0.50	Sugar hydrolase OS=Lactococcus lactis subsp. lactis (strain IL1403) OX=272623 GN=yeeB PE=1 SV=1
445	1	lactococcus	Q9CEG9	107	26498	3	3	3	3	0.61	tRNA-guanosine methyltransferase OS=Lactococcus lactis subsp. lactis (strain IL1403) OX=272623 GN=trmH PE=4 SV=1
419	1	lactococcus	Q9CG29	125	22412	4	4	3	3	0.75	dITP/XTP pyrophosphatase OS=Lactococcus lactis subsp. lactis (strain IL1403) OX=272623 GN=ynbD PE=3 SV=1
450	1	lactococcus	Q9CIK7	104	59465	3	3	3	3	0.24	Peptide chain release factor 3 OS=Lactococcus lactis subsp. lactis (strain IL1403) OX=272623 GN=prfC PE=3 SV=1
332	1	lactococcus	Q9CET0	212	23367	4	4	3	3	0.71	UPF0637 protein YsbB OS=Lactococcus lactis subsp. lactis (strain IL1403) OX=272623 GN=ysbB PE=3 SV=1
361	1	lactococcus	P95676	177	26336	5	5	3	3	0.61	Alpha-acetolactate decarboxylase OS=Lactococcus lactis subsp. lactis (strain IL1403) OX=272623 GN=aldB PE=3 SV=1

302	1	lactococcus	Q9CHG3	255	13408	7	7	3	3	1.51	Ribosome-binding factor A OS=Lactococcus lactis subsp. lactis (strain IL1403) OX=272623 GN=rbfA PE=3 SV=1
401	1	lactococcus	Q9CDS1	136	98671	3	3	3	3	0.14	DNA polymerase I OS=Lactococcus lactis subsp. lactis (strain IL1403) OX=272623 GN=polA PE=3 SV=1
343	1	lactococcus	Q9CEU2	200	31039	5	5	3	3	0.50	Prephenate dehydratase OS=Lactococcus lactis subsp. lactis (strain IL1403) OX=272623 GN=pheA PE=4 SV=1
384	1	lactococcus	Q9CEP8	149	52658	3	3	3	3	0.27	Uncharacterized protein OS=Lactococcus lactis subsp. lactis (strain IL1403) OX=272623 GN=yseF PE=3 SV=1
308	1	lactococcus	Q9CHJ5	248	35035	5	5	3	3	0.43	L-asparaginase OS=Lactococcus lactis subsp. lactis (strain IL1403) OX=272623 GN=ansB PE=3 SV=1
410	1	lactococcus	Q9CDM1	129	16717	4	4	3	3	1.69	Uncharacterized protein OS=Lactococcus lactis subsp. lactis (strain IL1403) OX=272623 GN=ywiD PE=4 SV=1
328	1	lactococcus	Q02522	217	20681	5	5	3	3	0.83	Hypoxanthine-guanine phosphoribosyltransferase OS=Lactococcus lactis subsp. lactis (strain IL1403) OX=272623 GN=hpt PE=3 SV=1
366	1	lactococcus	Q9CIF5	169	38708	6	6	3	3	0.38	Isopentenyl-diphosphate delta-isomerase OS=Lactococcus lactis subsp. lactis (strain IL1403) OX=272623 GN=fni PE=3 SV=1
348	1	lactococcus	Q9CH84	188	47139	4	4	3	3	0.31	Exodeoxyribonuclease 7 large subunit OS=Lactococcus lactis subsp. lactis (strain IL1403) OX=272623 GN=xseA PE=3 SV=1
407	1	lactococcus	Q9CIE3	130	37340	4	4	3	3	0.40	Transcriptional regulator OS=Lactococcus lactis subsp. lactis (strain IL1403) OX=272623 GN=yecE PE=3 SV=1
335	1	lactococcus	Q9CHV6	209	32101	6	6	3	3	0.69	Methionine aminopeptidase OS=Lactococcus lactis subsp. lactis (strain IL1403) OX=272623 GN=pepM PE=3 SV=1
378	1	lactococcus	Q9CIM5	155	34691	4	4	3	3	0.44	Fhu operon transcriptional regulator OS=Lactococcus lactis subsp. lactis (strain IL1403) OX=272623 GN=fhuR PE=3 SV=1
339	1	lactococcus	Q9CEE0	205	43037	3	3	3	3	0.34	S-adenosylmethionine synthase OS=Lactococcus lactis subsp. lactis (strain IL1403) OX=272623 GN=metK PE=3 SV=1
415	1	lactococcus	Q9CHV7	128	13404	3	3	3	3	1.51	Uncharacterized protein OS=Lactococcus lactis subsp. lactis (strain IL1403) OX=272623 GN=yfjH PE=4 SV=1
353	1	lactococcus	Q9CGK1	184	36184	5	5	3	3	0.42	Glycine--tRNA ligase alpha subunit OS=Lactococcus lactis subsp. lactis (strain IL1403) OX=272623 GN=glyQ PE=3 SV=1
368	1	lactococcus	Q9CIJ2	168	29956	4	4	3	3	0.52	Peptidyl-prolyl cis-trans isomerase OS=Lactococcus lactis subsp. lactis (strain IL1403) OX=272623 GN=ppiA PE=3 SV=1
345	1	lactococcus	Q9CIW6	197	7014	5	5	3	3	4.60	30S ribosomal protein S21 OS=Lactococcus lactis subsp. lactis (strain IL1403) OX=272623 GN=rpsU PE=3 SV=1
404	1	lactococcus	Q9CIT4	131	46564	3	3	3	3	0.31	Probable N-acetylmuramidase OS=Lactococcus lactis subsp. lactis (strain IL1403) OX=272623 GN=acmA PE=3 SV=1
350	1	lactococcus	Q9CJ54	187	43636	3	3	3	3	0.34	Queuine tRNA-ribosyltransferase OS=Lactococcus lactis subsp. lactis (strain IL1403) OX=272623 GN=tgt PE=3 SV=1
374	1	lactococcus	Q9CG32	161	28240	4	4	3	3	0.56	Tyrosine recombinase XerD-like OS=Lactococcus lactis subsp. lactis (strain IL1403) OX=272623 GN=ynbA PE=1 SV=2
375	1	lactococcus	Q9CHA6	160	17399	4	4	3	3	01.04	Transcriptional regulator OS=Lactococcus lactis subsp. lactis (strain IL1403) OX=272623 GN=copR PE=1 SV=1
189	1	lactococcus	Q9CDV7	561	21097	8	8	2	2	0.81	Transcription termination/antitermination protein NusG OS=Lactococcus lactis subsp. lactis (strain IL1403) OX=272623 GN=nusG PE=3 SV=1
220	1	lactococcus	Q9CHI4	473	9828	9	9	2	2	2.45	Uncharacterized protein OS=Lactococcus lactis subsp. lactis (strain IL1403) OX=272623 GN=yhfC PE=4 SV=1
533	1	lactococcus	Q9CEL0	65	19180	2	2	2	2	0.54	Uncharacterized protein OS=Lactococcus lactis subsp. lactis (strain IL1403) OX=272623 GN=ysjF PE=4 SV=1
236	1	lactococcus	Q9CDW9	429	15347	7	7	2	2	0.72	50S ribosomal protein L16 OS=Lactococcus lactis subsp. lactis (strain IL1403) OX=272623 GN=rplP PE=3 SV=1
334	1	lactococcus	Q9CDQ6	210	19116	6	6	2	2	0.55	Phosphopantetheine adenylyltransferase OS=Lactococcus lactis subsp. lactis (strain IL1403) OX=272623 GN=coaD PE=3 SV=1
275	1	lactococcus	Q48661	323	22125	6	6	2	2	0.76	Peptide deformylase OS=Lactococcus lactis subsp. lactis (strain IL1403) OX=272623 GN=def PE=3 SV=3
472	1	lactococcus	Q9CIH6	92	18297	3	3	2	2	0.57	Uncharacterized protein OS=Lactococcus lactis subsp. lactis (strain IL1403) OX=272623 GN=ydiG PE=4 SV=1
233	1	lactococcus	Q9CIH1	440	12127	8	8	2	2	1.77	Thioredoxin H-type OS=Lactococcus lactis subsp. lactis (strain IL1403) OX=272623 GN=trxH PE=4 SV=1

342	1	lactococcus	Q9CJ51	202	16651	3	3	2	2	0.65	CoA_binding domain-containing protein OS=Lactococcus lactis subsp. lactis (strain IL1403) OX=272623 GN=ybfE PE=4 SV=1
281	1	lactococcus	Q9CHX8	303	18734	5	5	2	2	0.56	Uncharacterized protein OS=Lactococcus lactis subsp. lactis (strain IL1403) OX=272623 GN=yfiD PE=4 SV=1
501	1	lactococcus	Q9CDW3	79	22292	2	2	2	2	0.45	50S ribosomal protein L4 OS=Lactococcus lactis subsp. lactis (strain IL1403) OX=272623 GN=rplD PE=3 SV=1
237	1	lactococcus	Q9CIV6	429	13386	6	6	2	2	0.85	PTS-dependent dihydroxyacetone kinase, phosphotransferase subunit DhaM OS=Lactococcus lactis subsp. lactis (strain IL1403) OX=272623 GN=dhaM PE=1 SV=1
421	1	lactococcus	Q9CER8	123	54337	2	2	2	2	0.17	ATP synthase subunit alpha OS=Lactococcus lactis subsp. lactis (strain IL1403) OX=272623 GN=atpA PE=3 SV=1
435	1	lactococcus	Q9CG30	113	19156	3	3	2	2	0.54	Phosphoesterase OS=Lactococcus lactis subsp. lactis (strain IL1403) OX=272623 GN=ynbC PE=3 SV=1
352	1	lactococcus	Q9CEK0	185	66050	6	6	2	2	0.14	Oligopeptide-binding protein OppA OS=Lactococcus lactis subsp. lactis (strain IL1403) OX=272623 GN=oppA PE=3 SV=1
557	1	lactococcus	Q9CH65	56	13111	2	2	2	2	0.88	50S ribosomal protein L19 OS=Lactococcus lactis subsp. lactis (strain IL1403) OX=272623 GN=rplS PE=3 SV=1
355	1	lactococcus	Q9CEX6	184	33671	3	3	2	2	0.28	Mannose-specific PTS system component IID OS=Lactococcus lactis subsp. lactis (strain IL1403) OX=272623 GN=ptnD PE=4 SV=1
461	1	lactococcus	Q9CFL7	95	6085	2	2	2	2	2.69	Uncharacterized protein OS=Lactococcus lactis subsp. lactis (strain IL1403) OX=272623 GN=yohD PE=4 SV=1
391	1	lactococcus	Q9CFL6	144	23053	2	2	2	2	0.44	GntR family transcriptional regulator OS=Lactococcus lactis subsp. lactis (strain IL1403) OX=272623 GN=busR PE=4 SV=1
525	1	lactococcus	Q9CGD8	68	46891	2	2	2	2	0.20	Homoserine dehydrogenase OS=Lactococcus lactis subsp. lactis (strain IL1403) OX=272623 GN=hom PE=3 SV=1
356	1	lactococcus	Q9CJC2	180	16671	3	3	2	2	0.65	Usp domain-containing protein OS=Lactococcus lactis subsp. lactis (strain IL1403) OX=272623 GN=yahB PE=3 SV=1
426	1	lactococcus	Q9CEJ8	119	7871	4	4	2	2	3.66	50S ribosomal protein L35 OS=Lactococcus lactis subsp. lactis (strain IL1403) OX=272623 GN=rpmI PE=3 SV=1
413	1	lactococcus	Q9CDW1	128	11734	4	4	2	2	01.01	30S ribosomal protein S10 OS=Lactococcus lactis subsp. lactis (strain IL1403) OX=272623 GN=rpsJ PE=3 SV=1
545	1	lactococcus	Q9CEG8	60	42078	2	2	2	2	0.22	DAO domain-containing protein OS=Lactococcus lactis subsp. lactis (strain IL1403) OX=272623 GN=yteB PE=4 SV=1
379	1	lactococcus	Q9CI77	153	19486	4	4	2	2	0.53	UPF0340 protein LL0489 OS=Lactococcus lactis subsp. lactis (strain IL1403) OX=272623 GN=yeiF PE=3 SV=1
492	1	lactococcus	P0A489	81	5993	2	2	2	2	2.78	50S ribosomal protein L33 3 OS=Lactococcus lactis subsp. lactis (strain IL1403) OX=272623 GN=rpmG3 PE=3 SV=1
396	1	lactococcus	Q9CGX1	140	41543	2	2	2	2	0.22	Peptide chain release factor 2 OS=Lactococcus lactis subsp. lactis (strain IL1403) OX=272623 GN=prfB PE=3 SV=1
513	1	lactococcus	Q9CJ95	74	35388	2	2	2	2	0.27	NodB homology domain-containing protein OS=Lactococcus lactis subsp. lactis (strain IL1403) OX=272623 GN=ybaG PE=4 SV=1
369	1	lactococcus	Q9CDH9	165	27487	3	3	2	2	0.36	Uncharacterized protein OS=Lactococcus lactis subsp. lactis (strain IL1403) OX=272623 GN=yxcD PE=4 SV=1
460	1	lactococcus	Q9CH06	97	21003	2	2	2	2	0.49	NLPC_P60 domain-containing protein OS=Lactococcus lactis subsp. lactis (strain IL1403) OX=272623 GN=yjgB PE=3 SV=1
417	1	lactococcus	Q9CDM3	126	18447	2	2	2	2	0.57	GAF domain-containing protein OS=Lactococcus lactis subsp. lactis (strain IL1403) OX=272623 GN=ywiB PE=4 SV=1
567	1	lactococcus	Q9CEE8	53	19522	2	2	2	2	0.53	3-dmu-9_3-mt domain-containing protein OS=Lactococcus lactis subsp. lactis (strain IL1403) OX=272623 GN=yteG PE=4 SV=1
358	1	lactococcus	Q9CGF0	179	21793	4	4	2	2	0.47	Xanthine phosphoribosyltransferase OS=Lactococcus lactis subsp. lactis (strain IL1403) OX=272623 GN=xpt PE=3 SV=1
477	1	lactococcus	Q9CDK3	88	12991	2	2	2	2	0.89	Uncharacterized protein OS=Lactococcus lactis subsp. lactis (strain IL1403) OX=272623 GN=ywjG PE=4 SV=1
405	1	lactococcus	P58002	131	103647	3	3	2	2	0.09	Translation initiation factor IF-2 OS=Lactococcus lactis subsp. lactis (strain IL1403) OX=272623 GN=infB PE=3 SV=1
521	1	lactococcus	Q9CDW2	71	21919	2	2	2	2	0.46	50S ribosomal protein L3 OS=Lactococcus lactis subsp. lactis (strain IL1403) OX=272623 GN=rplC PE=3 SV=1

370	1	lactococcus	Q9CH79	163	16861	2	2	2	2	0.64	Arginine repressor OS=Lactococcus lactis subsp. lactis (strain IL1403) OX=272623 GN=ahrC PE=3 SV=1
439	1	lactococcus	Q9CGL5	110	10049	4	4	2	2	1.25	50S ribosomal protein L27 OS=Lactococcus lactis subsp. lactis (strain IL1403) OX=272623 GN=rpmA PE=3 SV=1
416	1	lactococcus	Q9CDU5	128	16403	3	3	2	2	0.66	Zinc transport transcriptional regulator OS=Lactococcus lactis subsp. lactis (strain IL1403) OX=272623 GN=zitR PE=1 SV=1
539	1	lactococcus	Q9CG79	63	50098	2	2	2	2	0.18	Methylenetetrahydrofolate--tRNA-(uracil-5-)-methyltransferase TrmFO OS=Lactococcus lactis subsp. lactis (strain IL1403) OX=272623 GN=trmFO PE=3 SV=1
371	1	lactococcus	Q9CEP4	163	45436	3	3	2	2	0.20	Uncharacterized protein OS=Lactococcus lactis subsp. lactis (strain IL1403) OX=272623 GN=ysfA PE=3 SV=1
485	1	lactococcus	Q01998	84	43856	2	2	2	2	0.21	Tryptophan synthase beta chain OS=Lactococcus lactis subsp. lactis (strain IL1403) OX=272623 GN=trpB PE=3 SV=1
397	1	lactococcus	Q9CFC8	139	21410	3	3	2	2	0.48	Peptide methionine sulfoxide reductase MsrA 1 OS=Lactococcus lactis subsp. lactis (strain IL1403) OX=272623 GN=msrA1 PE=3 SV=1
498	1	lactococcus	Q9CGW7	80	81720	2	2	2	2	0.11	Ribonucleoside-diphosphate reductase OS=Lactococcus lactis subsp. lactis (strain IL1403) OX=272623 GN=nrdE PE=3 SV=1
424	1	lactococcus	Q9CES3	121	69799	2	2	2	2	0.13	Uncharacterized protein OS=Lactococcus lactis subsp. lactis (strain IL1403) OX=272623 GN=yscA PE=4 SV=1
451	1	lactococcus	Q9CDG6	103	16185	2	2	2	2	0.67	50S ribosomal protein L13 OS=Lactococcus lactis subsp. lactis (strain IL1403) OX=272623 GN=rplM PE=3 SV=1
393	1	lactococcus	Q9CDZ8	143	41638	3	3	2	2	0.22	Pyridine nucleotide-disulfide oxidoreductase OS=Lactococcus lactis subsp. lactis (strain IL1403) OX=272623 GN=yvdG PE=4 SV=1
559	1	lactococcus	Q9CI70	55	121588	2	2	2	2	0.07	DNA polymerase III subunit alpha OS=Lactococcus lactis subsp. lactis (strain IL1403) OX=272623 GN=dnaE PE=3 SV=1
420	1	lactococcus	Q9CH94	125	40847	3	3	2	2	0.23	Alanine racemase OS=Lactococcus lactis subsp. lactis (strain IL1403) OX=272623 GN=alr PE=3 SV=1
467	1	lactococcus	Q9CEK6	93	16219	3	3	2	2	0.67	DUF5590 domain-containing protein OS=Lactococcus lactis subsp. lactis (strain IL1403) OX=272623 GN=ysjH PE=4 SV=1
400	1	lactococcus	Q9CDS4	137	8614	5	5	2	2	03.08	Uncharacterized protein OS=Lactococcus lactis subsp. lactis (strain IL1403) OX=272623 GN=ywaH PE=4 SV=1
529	1	lactococcus	Q9CGW5	67	8389	2	2	2	2	1.62	Glutaredoxin-like protein NrdH OS=Lactococcus lactis subsp. lactis (strain IL1403) OX=272623 GN=nrdH PE=3 SV=2
392	1	lactococcus	Q9CHD9	143	8813	4	4	2	2	1.53	Uncharacterized protein OS=Lactococcus lactis subsp. lactis (strain IL1403) OX=272623 GN=yiaA PE=4 SV=1
429	1	lactococcus	Q9CFK0	116	14680	2	2	2	2	0.76	Ferric uptake regulator OS=Lactococcus lactis subsp. lactis (strain IL1403) OX=272623 GN=fur PE=3 SV=1
394	1	lactococcus	Q9CEI1	142	13146	3	3	2	2	0.88	Uncharacterized protein OS=Lactococcus lactis subsp. lactis (strain IL1403) OX=272623 GN=ytcE PE=4 SV=1
552	1	lactococcus	Q9CDG3	58	32231	2	2	2	2	0.30	Ribonuclease HIII OS=Lactococcus lactis subsp. lactis (strain IL1403) OX=272623 GN=rnhC PE=3 SV=1
495	1	lactococcus	Q9CHW3	81	23477	2	2	2	2	0.43	Phosphoserine phosphatase OS=Lactococcus lactis subsp. lactis (strain IL1403) OX=272623 GN=serB PE=1 SV=1
507	1	lactococcus	Q9CJ30	76	16711	2	2	2	2	0.64	Deoxyuridine 5'-triphosphate nucleotidohydrolase OS=Lactococcus lactis subsp. lactis (strain IL1403) OX=272623 GN=dut PE=3 SV=1
455	1	lactococcus	Q9CIM1	100	15488	4	4	2	2	0.70	VOC domain-containing protein OS=Lactococcus lactis subsp. lactis (strain IL1403) OX=272623 GN=yddD PE=4 SV=1
569	1	lactococcus	Q9CIU2	52	23810	2	2	2	2	0.42	Uncharacterized protein OS=Lactococcus lactis subsp. lactis (strain IL1403) OX=272623 GN=ycgC PE=4 SV=1
474	1	lactococcus	Q9CFH7	90	50432	2	2	2	2	0.18	Uncharacterized protein OS=Lactococcus lactis subsp. lactis (strain IL1403) OX=272623 GN=ypdC PE=4 SV=1
519	1	lactococcus	Q9CIW0	71	36351	2	2	2	2	0.26	DhaKLM operon coactivator DhaQ OS=Lactococcus lactis subsp. lactis (strain IL1403) OX=272623 GN=dhaQ PE=1 SV=2
436	1	lactococcus	Q9CJ14	111	7215	3	3	2	2	02.05	50S ribosomal protein L28 OS=Lactococcus lactis subsp. lactis (strain IL1403) OX=272623 GN=rpmB PE=3 SV=1
535	1	lactococcus	Q9CEE5	65	89287	2	2	2	2	0.10	Primosomal protein N' OS=Lactococcus lactis subsp. lactis (strain IL1403) OX=272623 GN=priA PE=3 SV=1
481	1	lactococcus	Q9CDX3	86	10870	3	3	2	2	1.12	50S ribosomal protein L24 OS=Lactococcus lactis subsp. lactis (strain IL1403) OX=272623 GN=rplX PE=3 SV=1

502	1	lactococcus	Q9CGJ1	78	29367	2	2	2	2	0.33	Lipid II isoglutaminyl synthase (glutamine-hydrolyzing) subunit GatD OS=Lactococcus lactis subsp. lactis (strain IL1403) OX=272623 GN=cobQ PE=3 SV=1
454	1	lactococcus	Q9CJ63	102	15657	2	2	2	2	0.70	Putative pre-16S rRNA nuclease OS=Lactococcus lactis subsp. lactis (strain IL1403) OX=272623 GN=ybeB PE=3 SV=2
558	1	lactococcus	Q9CI17	55	45789	2	2	2	2	0.20	UDP-N-acetylglucosamine 1-carboxyvinyltransferase 1 OS=Lactococcus lactis subsp. lactis (strain IL1403) OX=272623 GN=murA1 PE=3 SV=1
463	1	lactococcus	Q9CIG9	94	48962	2	2	2	2	0.19	NADH oxidase OS=Lactococcus lactis subsp. lactis (strain IL1403) OX=272623 GN=noxE PE=4 SV=1
531	1	lactococcus	Q9CEH1	66	49412	2	2	2	2	0.19	Cell division protein FtsA OS=Lactococcus lactis subsp. lactis (strain IL1403) OX=272623 GN=ftsA PE=3 SV=1
434	1	lactococcus	Q9CE67	114	35078	2	2	2	2	0.27	Uncharacterized protein OS=Lactococcus lactis subsp. lactis (strain IL1403) OX=272623 GN=yufC PE=4 SV=1
546	1	lactococcus	Q9CG16	60	32112	2	2	2	2	0.30	Ribosome biogenesis GTPase A OS=Lactococcus lactis subsp. lactis (strain IL1403) OX=272623 GN=ylqL PE=3 SV=1
494	1	lactococcus	Q9CIH4	81	86912	2	2	2	2	0.10	DD-transpeptidase OS=Lactococcus lactis subsp. lactis (strain IL1403) OX=272623 GN=pbp1B PE=4 SV=1
514	1	lactococcus	Q9CH37	74	23895	2	2	2	2	0.42	Uncharacterized protein OS=Lactococcus lactis subsp. lactis (strain IL1403) OX=272623 GN=yjcF PE=4 SV=1
456	1	lactococcus	Q01997	99	27714	2	2	2	2	0.35	Tryptophan synthase alpha chain OS=Lactococcus lactis subsp. lactis (strain IL1403) OX=272623 GN=trpA PE=3 SV=1
570	1	lactococcus	Q9CFW2	52	21257	2	2	2	2	0.48	Pseudouridine synthase OS=Lactococcus lactis subsp. lactis (strain IL1403) OX=272623 GN=rluC PE=3 SV=1
478	1	lactococcus	Q9CHW2	88	10929	3	3	2	2	1.12	Acylphosphatase OS=Lactococcus lactis subsp. lactis (strain IL1403) OX=272623 GN=acyP PE=3 SV=1
522	1	lactococcus	Q9CEK9	70	50854	2	2	2	2	0.18	Asparagine--tRNA ligase OS=Lactococcus lactis subsp. lactis (strain IL1403) OX=272623 GN=asnS PE=3 SV=1
440	1	lactococcus	Q9CDJ2	110	72280	2	2	2	2	0.12	Asparagine synthetase OS=Lactococcus lactis subsp. lactis (strain IL1403) OX=272623 GN=asnH PE=3 SV=1
541	1	lactococcus	Q9CEY2	63	52517	2	2	2	2	0.17	Transcription regulator OS=Lactococcus lactis subsp. lactis (strain IL1403) OX=272623 GN=yrfE PE=3 SV=1
487	1	lactococcus	Q9CIX4	83	25835	2	2	2	2	0.38	Probable transcriptional regulatory protein YcdB OS=Lactococcus lactis subsp. lactis (strain IL1403) OX=272623 GN=ycdB PE=3 SV=1
500	1	lactococcus	Q9CEX5	79	14301	2	2	2	2	0.78	Uncharacterized protein OS=Lactococcus lactis subsp. lactis (strain IL1403) OX=272623 GN=yrgE PE=4 SV=1
428	1	lactococcus	Q9CDF9	116	15114	2	2	2	2	0.73	30S ribosomal protein S12 OS=Lactococcus lactis subsp. lactis (strain IL1403) OX=272623 GN=rpsL PE=3 SV=1
554	1	lactococcus	P46469	58	75513	2	2	2	2	0.12	ATP-dependent zinc metalloprotease FtsH OS=Lactococcus lactis subsp. lactis (strain IL1403) OX=272623 GN=ftsH PE=3 SV=1
469	1	lactococcus	Q9CI79	92	28919	2	2	2	2	0.34	Phosphomethylpyrimidine kinase OS=Lactococcus lactis subsp. lactis (strain IL1403) OX=272623 GN=thiD2 PE=3 SV=1
518	1	lactococcus	Q9CHV8	71	11891	2	2	2	2	1.00	Transcriptional regulator OS=Lactococcus lactis subsp. lactis (strain IL1403) OX=272623 GN=yfjG PE=4 SV=1
441	1	lactococcus	Q9CF42	109	32727	2	2	2	2	0.29	Ribokinase OS=Lactococcus lactis subsp. lactis (strain IL1403) OX=272623 GN=rbsK PE=3 SV=1
542	1	lactococcus	Q9CH00	61	92285	2	2	2	2	0.10	Ribonuclease R 1 OS=Lactococcus lactis subsp. lactis (strain IL1403) OX=272623 GN=rnr1 PE=3 SV=1
496	1	lactococcus	Q9CJ44	80	11206	2	2	2	2	01.08	Glutamyl-tRNA(Gln) amidotransferase subunit C OS=Lactococcus lactis subsp. lactis (strain IL1403) OX=272623 GN=gatC PE=3 SV=1
508	1	lactococcus	Q9CFZ5	76	35569	2	2	2	2	0.27	Metal ABC transporter substrate-binding lipoprotein OS=Lactococcus lactis subsp. lactis (strain IL1403) OX=272623 GN=mtsA PE=3 SV=1
465	1	lactococcus	Q9CFI9	94	31341	2	2	2	2	0.31	Fructokinase OS=Lactococcus lactis subsp. lactis (strain IL1403) OX=272623 GN=scrK PE=4 SV=1
520	1	lactococcus	Q9CH60	71	15734	2	2	2	2	0.69	Universal stress protein OS=Lactococcus lactis subsp. lactis (strain IL1403) OX=272623 GN=yjaB PE=3 SV=1
482	1	lactococcus	Q9CE24	86	14416	2	2	2	2	0.77	Rhodanese domain-containing protein OS=Lactococcus lactis subsp. lactis (strain IL1403) OX=272623 GN=yvaB PE=4 SV=1
509	1	lactococcus	Q9CE78	76	49065	2	2	2	2	0.19	Histidine--tRNA ligase OS=Lactococcus lactis subsp. lactis (strain IL1403) OX=272623 GN=hisS PE=3 SV=1
462	1	lactococcus	Q9CHH6	95	48835	2	2	2	2	0.19	GTPase Der OS=Lactococcus lactis subsp. lactis (strain IL1403) OX=272623 GN=der PE=3 SV=1
486	1	lactococcus	Q9CDG2	83	23535	2	2	2	2	0.43	Signal peptidase I OS=Lactococcus lactis subsp. lactis (strain IL1403) OX=272623 GN=sipL PE=3 SV=1
27	1	lactococcus	Q9CJ83	5598	9174	127	127	1	1	0.56	Phosphocarrier protein HPr OS=Lactococcus lactis subsp. lactis (strain IL1403) OX=272623 GN=ptsH PE=1 SV=1

256	1	lactococcus	Q9CJA6	375	6659	9	9	1	1	0.83	50S ribosomal protein L32 OS=Lactococcus lactis subsp. lactis (strain IL1403) OX=272623 GN=rpmF PE=3 SV=2
317	1	lactococcus	Q9CDT0	226	11953	3	3	1	1	0.99	Uncharacterized protein OS=Lactococcus lactis subsp. lactis (strain IL1403) OX=272623 GN=ywaB PE=3 SV=1
280	1	lactococcus	P0A3K4	305	8190	5	5	1	1	0.64	Translation initiation factor IF-1 OS=Lactococcus lactis subsp. lactis (strain IL1403) OX=272623 GN=infA PE=3 SV=2
323	1	lactococcus	Q9CDM0	224	7492	4	4	1	1	0.72	Uncharacterized protein OS=Lactococcus lactis subsp. lactis (strain IL1403) OX=272623 GN=ywiE PE=4 SV=1
561	1	lactococcus	Q9CE10	54	50065	1	1	1	1	0.09	UDP-N-acetylmuramate--L-alanine ligase OS=Lactococcus lactis subsp. lactis (strain IL1403) OX=272623 GN=murC PE=3 SV=2
389	1	lactococcus	Q9CGZ6	145	9276	3	3	1	1	0.55	UPF0237 protein YjhC OS=Lactococcus lactis subsp. lactis (strain IL1403) OX=272623 GN=yjhC PE=3 SV=1
447	1	lactococcus	Q9CDW4	106	10737	2	2	1	1	0.46	50S ribosomal protein L23 OS=Lactococcus lactis subsp. lactis (strain IL1403) OX=272623 GN=rplW PE=3 SV=1
341	1	lactococcus	Q9CDM4	203	24158	3	3	1	1	0.19	APH domain-containing protein OS=Lactococcus lactis subsp. lactis (strain IL1403) OX=272623 GN=ywiA PE=4 SV=1
527	1	lactococcus	Q9CH85	67	31120	1	1	1	1	0.14	Bifunctional protein FolD OS=Lactococcus lactis subsp. lactis (strain IL1403) OX=272623 GN=folD PE=3 SV=2
387	1	lactococcus	Q9CHF3	146	49652	2	2	1	1	0.09	Biotin carboxylase OS=Lactococcus lactis subsp. lactis (strain IL1403) OX=272623 GN=accC PE=4 SV=1
425	1	lactococcus	P0A493	120	4556	3	3	1	1	4.56	50S ribosomal protein L36 OS=Lactococcus lactis subsp. lactis (strain IL1403) OX=272623 GN=rpmJ PE=3 SV=1
422	1	lactococcus	Q9CIH0	123	22296	2	2	1	1	0.21	tRNA-binding domain-containing protein OS=Lactococcus lactis subsp. lactis (strain IL1403) OX=272623 GN=ydjD PE=4 SV=1
543	1	lactococcus	Q9CE41	61	32293	1	1	1	1	0.14	S1 motif domain-containing protein OS=Lactococcus lactis subsp. lactis (strain IL1403) OX=272623 GN=yuiC PE=3 SV=1
390	1	lactococcus	P58121	145	15367	2	2	1	1	0.31	50S ribosomal protein L15 OS=Lactococcus lactis subsp. lactis (strain IL1403) OX=272623 GN=rplO PE=3 SV=1
471	1	lactococcus	Q9CH56	92	12509	1	1	1	1	0.39	FeS_assembly_P domain-containing protein OS=Lactococcus lactis subsp. lactis (strain IL1403) OX=272623 GN=yjaF PE=4 SV=1
395	1	lactococcus	Q9CIH8	141	15869	2	2	1	1	0.30	Uncharacterized protein OS=Lactococcus lactis subsp. lactis (strain IL1403) OX=272623 GN=ydiE PE=3 SV=1
503	1	lactococcus	Q9CIU6	78	15044	1	1	1	1	0.32	Uncharacterized protein OS=Lactococcus lactis subsp. lactis (strain IL1403) OX=272623 GN=ycfH PE=4 SV=1
423	1	lactococcus	Q9CDV0	121	14121	3	3	1	1	0.34	FMN-binding protein OS=Lactococcus lactis subsp. lactis (strain IL1403) OX=272623 GN=yviC PE=4 SV=1
457	1	lactococcus	Q9CEX7	99	27637	1	1	1	1	0.16	Mannose-specific PTS system component IIC OS=Lactococcus lactis subsp. lactis (strain IL1403) OX=272623 GN=ptnC PE=4 SV=1
563	1	lactococcus	Q9CDG7	53	14090	1	1	1	1	0.34	30S ribosomal protein S9 OS=Lactococcus lactis subsp. lactis (strain IL1403) OX=272623 GN=rpsI PE=3 SV=1
489	1	lactococcus	Q9CEY9	82	11948	1	1	1	1	0.41	Transcription regulator OS=Lactococcus lactis subsp. lactis (strain IL1403) OX=272623 GN=yrfA PE=4 SV=1
523	1	lactococcus	Q9CHU0	70	33051	1	1	1	1	0.14	PHB domain-containing protein OS=Lactococcus lactis subsp. lactis (strain IL1403) OX=272623 GN=ygbE PE=4 SV=1
437	1	lactococcus	Q9CG03	111	7523	1	1	1	1	0.71	Uncharacterized protein OS=Lactococcus lactis subsp. lactis (strain IL1403) OX=272623 GN=yneC PE=4 SV=1
537	1	lactococcus	Q9CDP4	64	9644	1	1	1	1	0.53	Uncharacterized protein OS=Lactococcus lactis subsp. lactis (strain IL1403) OX=272623 GN=ywfB PE=4 SV=1
468	1	lactococcus	Q9CE11	93	16929	1	1	1	1	0.28	Arginine repressor OS=Lactococcus lactis subsp. lactis (strain IL1403) OX=272623 GN=argR PE=3 SV=1
497	1	lactococcus	P0A2Z0	80	17992	1	1	1	1	0.26	ATP synthase subunit b OS=Lactococcus lactis subsp. lactis (strain IL1403) OX=272623 GN=atpF PE=3 SV=1
458	1	lactococcus	Q9CHZ4	99	6794	1	1	1	1	0.81	Probable tautomerase LL0574 OS=Lactococcus lactis subsp. lactis (strain IL1403) OX=272623 GN=LL0574 PE=3 SV=3
555	1	lactococcus	Q9CED5	58	49545	1	1	1	1	0.09	N-acetylmuramidase OS=Lactococcus lactis subsp. lactis (strain IL1403) OX=272623 GN=acmB PE=3 SV=1
483	1	lactococcus	Q9CJ47	86	58336	1	1	1	1	0.07	Uncharacterized protein OS=Lactococcus lactis subsp. lactis (strain IL1403) OX=272623 GN=ybgB PE=4 SV=1
526	1	lactococcus	Q9CEL7	68	19795	1	1	1	1	0.23	Alpha-ribazole-5'-phosphate phosphatase OS=Lactococcus lactis subsp. lactis (strain IL1403) OX=272623 GN=cobC PE=4 SV=1

459	1	lactococcus	Q9CDW7	98	12462	2	2	1	1	0.39	50S ribosomal protein L22 OS=Lactococcus lactis subsp. lactis (strain IL1403) OX=272623 GN=rplV PE=3 SV=1
551	1	lactococcus	Q9CJ89	59	50060	1	1	1	1	0.09	Cationic amino acid transporter OS=Lactococcus lactis subsp. lactis (strain IL1403) OX=272623 GN=ctrA PE=4 SV=1
475	1	lactococcus	Q9CGM5	89	24799	1	1	1	1	0.18	Endonuclease III OS=Lactococcus lactis subsp. lactis (strain IL1403) OX=272623 GN=nth PE=3 SV=1
511	1	lactococcus	Q9CHI8	74	25228	1	1	1	1	0.18	Uncharacterized protein OS=Lactococcus lactis subsp. lactis (strain IL1403) OX=272623 GN=yhfA PE=4 SV=1
490	1	lactococcus	Q9CHT5	82	18793	2	2	1	1	0.25	Adenine phosphoribosyltransferase OS=Lactococcus lactis subsp. lactis (strain IL1403) OX=272623 GN=apt PE=3 SV=2
565	1	lactococcus	Q9CGI3	53	32414	1	1	1	1	0.14	Uncharacterized protein OS=Lactococcus lactis subsp. lactis (strain IL1403) OX=272623 GN=ylcC PE=4 SV=1
464	1	lactococcus	Q9CGW3	94	71674	2	2	1	1	0.06	DNA topoisomerase 4 subunit B OS=Lactococcus lactis subsp. lactis (strain IL1403) OX=272623 GN=parE PE=3 SV=1
515	1	lactococcus	Q9CGM4	73	25962	1	1	1	1	0.17	Uncharacterized protein OS=Lactococcus lactis subsp. lactis (strain IL1403) OX=272623 GN=ykiC PE=4 SV=1
488	1	lactococcus	Q9CJF1	83	8228	1	1	1	1	0.64	Prophage ps1 protein 20 OS=Lactococcus lactis subsp. lactis (strain IL1403) OX=272623 GN=ps120 PE=4 SV=1
534	1	lactococcus	Q9CE74	65	31851	1	1	1	1	0.14	Uncharacterized protein OS=Lactococcus lactis subsp. lactis (strain IL1403) OX=272623 GN=yueD PE=4 SV=1
473	1	lactococcus	Q9CDY2	91	13279	2	2	1	1	0.36	30S ribosomal protein S11 OS=Lactococcus lactis subsp. lactis (strain IL1403) OX=272623 GN=rpsK PE=3 SV=1
505	1	lactococcus	Q9CHN9	77	23605	2	2	1	1	0.42	Ribonuclease M5 OS=Lactococcus lactis subsp. lactis (strain IL1403) OX=272623 GN=rnmV PE=3 SV=1
491	1	lactococcus	Q9CGK0	82	75670	1	1	1	1	0.06	Glycine--tRNA ligase beta subunit OS=Lactococcus lactis subsp. lactis (strain IL1403) OX=272623 GN=glyS PE=3 SV=1
560	1	lactococcus	Q9CHU2	55	33602	1	1	1	1	0.13	tRNA dimethylallyltransferase OS=Lactococcus lactis subsp. lactis (strain IL1403) OX=272623 GN=miaA PE=3 SV=1
476	1	lactococcus	Q9CGZ2	89	27461	1	1	1	1	0.16	D-alanyl-D-alanine carboxypeptidase OS=Lactococcus lactis subsp. lactis (strain IL1403) OX=272623 GN=dacB PE=4 SV=1
530	1	lactococcus	Q9CES4	67	27100	2	2	1	1	0.17	Glutamine ABC transporter ATP-binding protein OS=Lactococcus lactis subsp. lactis (strain IL1403) OX=272623 GN=glnQ PE=4 SV=1
544	1	lactococcus	Q9CFK4	60	41291	1	1	1	1	0.11	Transcriptional regulator OS=Lactococcus lactis subsp. lactis (strain IL1403) OX=272623 GN=rmaC PE=4 SV=1
512	1	lactococcus	Q9CEV0	74	54564	1	1	1	1	0.08	Iron-binding oxidase subunit OS=Lactococcus lactis subsp. lactis (strain IL1403) OX=272623 GN=yrjC PE=4 SV=1
568	1	lactococcus	Q9CDM2	53	10771	1	1	1	1	0.46	Chorismate mutase domain-containing protein OS=Lactococcus lactis subsp. lactis (strain IL1403) OX=272623 GN=ywiC PE=4 SV=1
524	1	lactococcus	Q9CH57	69	85096	1	1	1	1	0.05	Uncharacterized protein OS=Lactococcus lactis subsp. lactis (strain IL1403) OX=272623 GN=yjaE PE=4 SV=1
540	1	lactococcus	Q9CIG4	63	23984	1	1	1	1	0.19	Thymidylate kinase OS=Lactococcus lactis subsp. lactis (strain IL1403) OX=272623 GN=tmk PE=3 SV=1
504	1	lactococcus	Q9CDN0	78	9365	1	1	1	1	0.55	30S ribosomal protein S18 OS=Lactococcus lactis subsp. lactis (strain IL1403) OX=272623 GN=rpsR PE=3 SV=1
556	1	lactococcus	Q9CDY4	57	14247	2	2	1	1	0.34	50S ribosomal protein L17 OS=Lactococcus lactis subsp. lactis (strain IL1403) OX=272623 GN=rplQ PE=3 SV=1
528	1	lactococcus	Q9CIY0	67	22472	1	1	1	1	0.20	HD domain-containing protein OS=Lactococcus lactis subsp. lactis (strain IL1403) OX=272623 GN=yccH PE=4 SV=1
553	1	lactococcus	Q9CJ80	58	11102	2	2	1	1	0.45	Nucleoid-associated protein LL0120 OS=Lactococcus lactis subsp. lactis (strain IL1403) OX=272623 GN=ybcG PE=3 SV=1
506	1	lactococcus	Q9CF10	77	55013	2	2	1	1	0.08	Phospho-beta-glucosidase OS=Lactococcus lactis subsp. lactis (strain IL1403) OX=272623 GN=yrcA PE=3 SV=1
564	1	lactococcus	Q9CE84	53	36619	1	1	1	1	0.12	tRNA-dihydrouridine synthase OS=Lactococcus lactis subsp. lactis (strain IL1403) OX=272623 GN=yudI PE=3 SV=1
532	1	lactococcus	Q9CE85	66	19645	1	1	1	1	0.24	RibD_C domain-containing protein OS=Lactococcus lactis subsp. lactis (strain IL1403) OX=272623 GN=yudH PE=4 SV=1
538	1	lactococcus	Q9CH99	64	25844	1	1	1	1	0.18	Uridine phosphorylase OS=Lactococcus lactis subsp. lactis (strain IL1403) OX=272623 GN=udp PE=3 SV=1

562	1	lactococcus	Q9CEM1	54	16745	1	1	1	1	0.28	N-acetyltransferase domain-containing protein OS=Lactococcus lactis subsp. lactis (strain IL1403) OX=272623 GN=ysiC PE=3 SV=1
547	1	lactococcus	Q9CEB7	59	35083	1	1	1	1	0.13	Small ribosomal subunit biogenesis GTPase RsgA OS=Lactococcus lactis subsp. lactis (strain IL1403) OX=272623 GN=rsgA PE=3 SV=1
571	1	lactococcus	Q9CDV5	52	5748	1	1	1	1	0.99	50S ribosomal protein L33 2 OS=Lactococcus lactis subsp. lactis (strain IL1403) OX=272623 GN=rpmG2 PE=3 SV=1
536	1	lactococcus	Q9CI25	65	37506	1	1	1	1	0.12	Peptidoglycan hydrolase OS=Lactococcus lactis subsp. lactis (strain IL1403) OX=272623 GN=acmD PE=3 SV=1
566	1	lactococcus	Q9CGZ9	53	21568	1	1	1	1	0.21	Isochorismatase domain-containing protein OS=Lactococcus lactis subsp. lactis (strain IL1403) OX=272623 GN=yjgF PE=4 SV=1
548	1	lactococcus	Q9CEW7	59	30272	1	1	1	1	0.15	Phosphate import ATP-binding protein PstB 2 OS=Lactococcus lactis subsp. lactis (strain IL1403) OX=272623 GN=pstB2 PE=3 SV=1
572	1	lactococcus	Q9CDX5	51	7314	1	1	1	1	0.73	30S ribosomal protein S14 type Z OS=Lactococcus lactis subsp. lactis (strain IL1403) OX=272623 GN=rpsZ PE=3 SV=1
549	1	lactococcus	Q9CDL8	59	14158	1	1	1	1	0.34	Glutamine synthetase repressor OS=Lactococcus lactis subsp. lactis (strain IL1403) OX=272623 GN=glnR PE=4 SV=1
573	1	lactococcus	Q9CHP1	51	28913	1	1	1	1	0.16	Uncharacterized protein OS=Lactococcus lactis subsp. lactis (strain IL1403) OX=272623 GN=ygiI PE=4 SV=1
550	1	lactococcus	Q9CF90	59	12560	1	1	1	1	0.39	Nitrogen regulatory protein P-II OS=Lactococcus lactis subsp. lactis (strain IL1403) OX=272623 GN=glnB PE=3 SV=1
574	1	lactococcus	Q9CGK5	51	23850	1	1	1	1	0.19	Uncharacterized protein OS=Lactococcus lactis subsp. lactis (strain IL1403) OX=272623 GN=ykjI PE=3 SV=1
575	1	lactococcus	P0C2U4	51	41452	1	1	1	1	0.11	Protein RecA, chromosomal OS=Lactococcus lactis subsp. lactis (strain IL1403) OX=272623 GN=recA PE=3 SV=1
576	1	lactococcus	Q9CIS3	50	41735	1	1	1	1	0.11	Endo-1,4-beta-xylanase D OS=Lactococcus lactis subsp. lactis (strain IL1403) OX=272623 GN=xynD PE=4 SV=1
577	1	lactococcus	Q9CHG8	50	17535	1	1	1	1	0.27	Ribosome maturation factor RimP OS=Lactococcus lactis subsp. lactis (strain IL1403) OX=272623 GN=rimP PE=3 SV=1
578	1	lactococcus	Q9CIL6	49	22079	1	1	1	1	0.21	Recombination protein RecR OS=Lactococcus lactis subsp. lactis (strain IL1403) OX=272623 GN=recR PE=3 SV=1
579	1	lactococcus	Q9CDI8	48	17069	1	1	1	1	0.27	Nudix hydrolase domain-containing protein OS=Lactococcus lactis subsp. lactis (strain IL1403) OX=272623 GN=yxbC PE=3 SV=1
580	1	lactococcus	Q9CGE0	48	47547	1	1	1	1	0.09	Folylpolyglutamate synthase OS=Lactococcus lactis subsp. lactis (strain IL1403) OX=272623 GN=folC PE=3 SV=1
581	1	lactococcus	Q9CGZ3	48	25516	1	1	1	1	0.18	Phosphoglycerate mutase OS=Lactococcus lactis subsp. lactis (strain IL1403) OX=272623 GN=yjhF PE=4 SV=1
582	1	lactococcus	Q9CG78	48	41372	1	1	1	1	0.11	Tyrosine recombinase XerS OS=Lactococcus lactis subsp. lactis (strain IL1403) OX=272623 GN=xerS PE=3 SV=1
583	1	lactococcus	Q9CDL2	48	21153	1	1	1	1	0.22	Holliday junction ATP-dependent DNA helicase RuvA OS=Lactococcus lactis subsp. lactis (strain IL1403) OX=272623 GN=ruvA PE=3 SV=1
584	1	lactococcus	Q9CHF5	48	16362	1	1	1	1	0.29	Biotin carboxyl carrier protein of acetyl-CoA carboxylase OS=Lactococcus lactis subsp. lactis (strain IL1403) OX=272623 GN=accB PE=4 SV=1
585	1	lactococcus	Q02137	48	62811	2	2	1	1	0.07	Acetolactate synthase large subunit OS=Lactococcus lactis subsp. lactis (strain IL1403) OX=272623 GN=ilvB PE=3 SV=2
586	1	lactococcus	Q9CID8	47	27894	1	1	1	1	0.16	GntR family transcriptional regulator OS=Lactococcus lactis subsp. lactis (strain IL1403) OX=272623 GN=rgrA PE=4 SV=1
587	1	lactococcus	Q9CG73	47	35124	1	1	1	1	0.13	N(5)-(carboxyethyl)ornithine synthase OS=Lactococcus lactis subsp. lactis (strain IL1403) OX=272623 GN=ceo PE=3 SV=1
588	1	lactococcus	Q9CJH7	47	14091	1	1	1	1	0.34	Uncharacterized protein OS=Lactococcus lactis subsp. lactis (strain IL1403) OX=272623 GN=yacC PE=4 SV=1
589	1	lactococcus	Q9CG24	46	164886	1	1	1	1	0.03	Glutamate synthase large subunit OS=Lactococcus lactis subsp. lactis (strain IL1403) OX=272623 GN=gltB PE=3 SV=1
590	1	lactococcus	Q9CIV7	46	20565	1	1	1	1	0.22	PTS-dependent dihydroxyacetone kinase, ADP-binding subunit DhaL OS=Lactococcus lactis subsp. lactis (strain IL1403) OX=272623 GN=dhaL PE=1 SV=1
591	1	lactococcus	Q9CGV8	46	92766	1	1	1	1	0.05	DNA topoisomerase 4 subunit A OS=Lactococcus lactis subsp. lactis (strain IL1403) OX=272623 GN=parC PE=3 SV=1

592	1	lactococcus	Q9CGM7	46	45740	1	1	1	1	0.10	Dihydroorotase OS=Lactococcus lactis subsp. lactis (strain IL1403) OX=272623 GN=pyrC PE=3 SV=2
593	1	lactococcus	Q9CET9	45	39471	1	1	1	1	0.11	Prephenate dehydrogenase OS=Lactococcus lactis subsp. lactis (strain IL1403) OX=272623 GN=tyrA PE=3 SV=1
594	1	lactococcus	Q9CJ09	45	9810	1	1	1	1	0.52	Uncharacterized protein OS=Lactococcus lactis subsp. lactis (strain IL1403) OX=272623 GN=ybjF PE=4 SV=1
595	1	lactococcus	Q9CHX6	44	14969	1	1	1	1	0.32	Uncharacterized protein OS=Lactococcus lactis subsp. lactis (strain IL1403) OX=272623 GN=yfiE PE=3 SV=1
596	1	lactococcus	Q9CIC9	44	49483	1	1	1	1	0.09	Transcriptional regulator OS=Lactococcus lactis subsp. lactis (strain IL1403) OX=272623 GN=yeeG PE=3 SV=1
597	1	lactococcus	Q9CF91	44	49424	1	1	1	1	0.09	UDP-N-acetylmuramoylalanine--D-glutamate ligase OS=Lactococcus lactis subsp. lactis (strain IL1403) OX=272623 GN=murD PE=3 SV=1
598	1	lactococcus	Q9CGY5	44	25910	1	1	1	1	0.18	Lactose transport regulator OS=Lactococcus lactis subsp. lactis (strain IL1403) OX=272623 GN=lacR PE=4 SV=1
599	1	lactococcus	Q9CEX3	44	13766	1	1	1	1	0.35	Uncharacterized protein OS=Lactococcus lactis subsp. lactis (strain IL1403) OX=272623 GN=yrgG PE=4 SV=1
600	1	lactococcus	Q9CIX3	44	24806	1	1	1	1	0.18	GyrI-like domain-containing protein OS=Lactococcus lactis subsp. lactis (strain IL1403) OX=272623 GN=ycdC PE=4 SV=1
601	1	lactococcus	Q9CIJ4	43	30454	1	1	1	1	0.15	NAD kinase OS=Lactococcus lactis subsp. lactis (strain IL1403) OX=272623 GN=nadK PE=3 SV=1
602	1	lactococcus	Q9CGZ4	43	18586	1	1	1	1	0.25	Uncharacterized protein OS=Lactococcus lactis subsp. lactis (strain IL1403) OX=272623 GN=yjhE PE=4 SV=1
603	1	lactococcus	Q9CFY4	43	61247	1	1	1	1	0.07	Rqc2 homolog RqcH OS=Lactococcus lactis subsp. lactis (strain IL1403) OX=272623 GN=yngB PE=3 SV=1
604	1	lactococcus	Q9CE60	43	44126	1	1	1	1	0.10	Transcriptional regulator OS=Lactococcus lactis subsp. lactis (strain IL1403) OX=272623 GN=nadR PE=4 SV=1
605	1	lactococcus	Q9CH17	42	16902	1	1	1	1	0.28	Glycerol-3-phosphate cytidyltransferase OS=Lactococcus lactis subsp. lactis (strain IL1403) OX=272623 GN=tagD2 PE=4 SV=1
606	1	lactococcus	Q9CJC6	42	30553	1	1	1	1	0.15	Uncharacterized protein OS=Lactococcus lactis subsp. lactis (strain IL1403) OX=272623 GN=yahA PE=4 SV=1
607	1	lactococcus	Q9CJ87	41	18683	1	1	1	1	0.25	Transcriptional regulator OS=Lactococcus lactis subsp. lactis (strain IL1403) OX=272623 GN=rmaD PE=4 SV=1
608	1	lactococcus	Q9CH03	41	28021	1	1	1	1	0.16	Amino acid ABC transporter ATP binding protein OS=Lactococcus lactis subsp. lactis (strain IL1403) OX=272623 GN=yjgE PE=4 SV=1
609	1	lactococcus	Q9CJI9	41	138639	1	1	1	1	0.03	ATP-dependent helicase/nuclease subunit A OS=Lactococcus lactis subsp. lactis (strain IL1403) OX=272623 GN=addA PE=3 SV=1
610	1	lactococcus	Q9CE71	40	13591	1	1	1	1	0.36	S4 RNA-binding domain-containing protein OS=Lactococcus lactis subsp. lactis (strain IL1403) OX=272623 GN=yufA PE=4 SV=1
611	1	lactococcus	Q9CFU3	40	15351	1	1	1	1	0.31	Usp domain-containing protein OS=Lactococcus lactis subsp. lactis (strain IL1403) OX=272623 GN=yobA PE=3 SV=1
612	1	lactococcus	Q9CIV4	40	52069	1	1	1	1	0.08	Probable dipeptidase A OS=Lactococcus lactis subsp. lactis (strain IL1403) OX=272623 GN=pepDA PE=3 SV=1
613	1	lactococcus	Q9CFG8	39	55962	1	1	1	1	0.08	Xylulose kinase OS=Lactococcus lactis subsp. lactis (strain IL1403) OX=272623 GN=xylB PE=3 SV=1
614	1	lactococcus	Q9CFY3	38	55535	1	1	1	1	0.08	Sugar ABC transporter ATP binding protein OS=Lactococcus lactis subsp. lactis (strain IL1403) OX=272623 GN=yngE PE=4 SV=1
615	1	lactococcus	Q9CE56	38	34627	1	1	1	1	0.13	Protease OS=Lactococcus lactis subsp. lactis (strain IL1403) OX=272623 GN=yuhB PE=4 SV=1
616	1	lactococcus	Q9CG40	38	7278	1	1	1	1	0.73	Uncharacterized protein OS=Lactococcus lactis subsp. lactis (strain IL1403) OX=272623 GN=ynaA PE=4 SV=1
617	1	lactococcus	Q9CFB6	38	19700	1	1	1	1	0.24	Ribosome maturation factor RimM OS=Lactococcus lactis subsp. lactis (strain IL1403) OX=272623 GN=rimM PE=3 SV=1
618	1	lactococcus	Q9CDX1	37	10137	1	1	1	1	0.50	30S ribosomal protein S17 OS=Lactococcus lactis subsp. lactis (strain IL1403) OX=272623 GN=rpsQ PE=3 SV=1
619	1	lactococcus	Q9CED9	37	28239	1	1	1	1	0.16	Bifunctional protein BirA OS=Lactococcus lactis subsp. lactis (strain IL1403) OX=272623 GN=birA2 PE=4 SV=1
620	1	lactococcus	Q07733	37	37360	1	1	1	1	0.12	Oligopeptide transport ATP-binding protein OppD OS=Lactococcus lactis subsp. lactis (strain IL1403) OX=272623 GN=oppD PE=3 SV=2
621	1	lactococcus	Q9CE61	37	32253	1	1	1	1	0.14	Oxidoreductase OS=Lactococcus lactis subsp. lactis (strain IL1403) OX=272623 GN=yugB PE=4 SV=1

622	1	lactococcus	Q9CEM4	36	30540	1	1	1	1	0.15	Pyruvate formate-lyase-activating enzyme OS=Lactococcus lactis subsp. lactis (strain IL1403) OX=272623 GN=pflA PE=3 SV=1
623	1	lactococcus	Q9CIK9	36	38855	1	1	1	1	0.11	Oligopeptide ABC trasporter ATP binding protein OS=Lactococcus lactis subsp. lactis (strain IL1403) OX=272623 GN=optD PE=3 SV=1
624	1	lactococcus	Q9CEW4	36	30588	1	1	1	1	0.15	Phosphate-binding protein OS=Lactococcus lactis subsp. lactis (strain IL1403) OX=272623 GN=pstE PE=3 SV=1
625	1	lactococcus	Q9CIN6	36	31534	1	1	1	1	0.14	Lipoprotein OS=Lactococcus lactis subsp. lactis (strain IL1403) OX=272623 GN=plpC PE=3 SV=1
626	1	lactococcus	Q9CEV1	35	28746	1	1	1	1	0.16	Oxidoreductase OS=Lactococcus lactis subsp. lactis (strain IL1403) OX=272623 GN=yrjB PE=4 SV=1
627	1	lactococcus	O34133	35	13750	1	1	1	1	0.35	Putative regulator AldR OS=Lactococcus lactis subsp. lactis (strain IL1403) OX=272623 GN=aldR PE=3 SV=2
628	1	lactococcus	Q9CDQ9	35	80431	1	1	1	1	0.05	Elp3 domain-containing protein OS=Lactococcus lactis subsp. lactis (strain IL1403) OX=272623 GN=ywdD PE=4 SV=1
629	1	lactococcus	Q9CEC1	35	36191	1	1	1	1	0.12	HD_domain domain-containing protein OS=Lactococcus lactis subsp. lactis (strain IL1403) OX=272623 GN=yuaA PE=4 SV=1
630	1	lactococcus	Q9CH20	34	93633	1	1	1	1	0.05	Teichoic acid biosynthesis protein OS=Lactococcus lactis subsp. lactis (strain IL1403) OX=272623 GN=tagX PE=3 SV=1
631	1	lactococcus	Q9CFW0	34	32145	1	1	1	1	0.14	DUF3324 domain-containing protein OS=Lactococcus lactis subsp. lactis (strain IL1403) OX=272623 GN=ynjC PE=4 SV=1
632	1	lactococcus	Q9CID1	34	16044	1	1	1	1	0.29	t(6)A37 threonylcarbamoyladenosine biosynthesis protein TsaE OS=Lactococcus lactis subsp. lactis (strain IL1403) OX=272623 GN=yeeE PE=3 SV=1
633	1	lactococcus	Q9CF75	33	11297	1	1	1	1	0.44	Transcriptional regulator OS=Lactococcus lactis subsp. lactis (strain IL1403) OX=272623 GN=rarA PE=4 SV=1
634	1	lactococcus	Q9CGV9	33	17283	1	1	1	1	0.27	N-acetyltransferase domain-containing protein OS=Lactococcus lactis subsp. lactis (strain IL1403) OX=272623 GN=ykbA PE=4 SV=1
635	1	lactococcus	Q9CJF6	33	14525	1	1	1	1	0.33	Prophage ps1 protein 15, transcriptional regulator OS=Lactococcus lactis subsp. lactis (strain IL1403) OX=272623 GN=ps115 PE=4 SV=1
636	1	lactococcus	Q9CE36	32	43197	1	1	1	1	0.10	Acetate kinase 2 OS=Lactococcus lactis subsp. lactis (strain IL1403) OX=272623 GN=ackA2 PE=3 SV=1
637	1	lactococcus	Q9CER9	32	32023	1	1	1	1	0.14	ATP synthase gamma chain OS=Lactococcus lactis subsp. lactis (strain IL1403) OX=272623 GN=atpG PE=3 SV=1
638	1	lactococcus	Q9CJH1	32	43692	1	1	1	1	0.10	Mannitol-1-phosphate 5-dehydrogenase OS=Lactococcus lactis subsp. lactis (strain IL1403) OX=272623 GN=mtlD PE=3 SV=1
639	1	lactococcus	Q9CI66	32	43621	1	1	1	1	0.10	Glyco_hydro_3 domain-containing protein OS=Lactococcus lactis subsp. lactis (strain IL1403) OX=272623 GN=yejJ PE=3 SV=1
640	1	lactococcus	Q9CG27	32	8973	1	1	1	1	0.57	UPF0154 protein YnbE OS=Lactococcus lactis subsp. lactis (strain IL1403) OX=272623 GN=ynbE PE=3 SV=1
641	1	lactococcus	Q9CDJ3	32	47681	1	1	1	1	0.09	ATP-grasp domain-containing protein OS=Lactococcus lactis subsp. lactis (strain IL1403) OX=272623 GN=yxbA PE=4 SV=1
642	1	lactococcus	Q9CJC3	32	30846	1	1	1	1	0.15	Short-chain type dehydrogenase OS=Lactococcus lactis subsp. lactis (strain IL1403) OX=272623 GN=yahI PE=3 SV=1
643	1	lactococcus	Q9CE89	32	19197	1	1	1	1	0.24	Uncharacterized protein OS=Lactococcus lactis subsp. lactis (strain IL1403) OX=272623 GN=yudD PE=4 SV=1
644	1	lactococcus	Q9CII1	32	45333	1	1	1	1	0.10	Probable tRNA sulfurtransferase OS=Lactococcus lactis subsp. lactis (strain IL1403) OX=272623 GN=thiI PE=3 SV=1
645	1	lactococcus	Q9CEK5	31	89889	1	1	1	1	0.05	3'-5' exonuclease DinG OS=Lactococcus lactis subsp. lactis (strain IL1403) OX=272623 GN=dinG PE=3 SV=1
646	1	lactococcus	Q9CHK5	31	15149	1	1	1	1	0.32	4HBT domain-containing protein OS=Lactococcus lactis subsp. lactis (strain IL1403) OX=272623 GN=yhdA PE=1 SV=1
647	1	lactococcus	Q9CH49	31	36752	1	1	1	1	0.12	Sensor histidine kinase OS=Lactococcus lactis subsp. lactis (strain IL1403) OX=272623 GN=kinD PE=4 SV=1
648	1	lactococcus	Q9CH58	30	20708	1	1	1	1	0.22	Transcriptional regulator OS=Lactococcus lactis subsp. lactis (strain IL1403) OX=272623 GN=yjaD PE=4 SV=1
649	1	lactococcus	Q9CF70	30	8958	1	1	1	1	0.57	DUF1858 domain-containing protein OS=Lactococcus lactis subsp. lactis (strain IL1403) OX=272623 GN=yqfB PE=4 SV=1

Proteins identified in active fractions obtained from Superdex 75 column

Family	Member	Database	Accession	Score	Mass	Num. of matches	Num. of significant matches	Num. of sequences	Num. of significant sequences	emPAI	Description
1	1	lactococcus	P37282	33057	57166	540	540	22	22	4.46	60 kDa chaperonin OS=Lactococcus lactis subsp. lactis (strain IL1403) OX=272623 GN=groL PE=3 SV=2
4	1	lactococcus	P0A3J0	8388	64947	219	219	14	14	1.48	Chaperone protein DnaK OS=Lactococcus lactis subsp. lactis (strain IL1403) OX=272623 GN=dnaK PE=3 SV=1
2	1	lactococcus	Q9CHS7	18793	46929	330	330	12	12	2.21	Enolase 1 OS=Lactococcus lactis subsp. lactis (strain IL1403) OX=272623 GN=eno1 PE=3 SV=1
9	1	lactococcus	Q9CHA0	1953	44683	62	62	9	9	1.56	30S ribosomal protein S1 OS=Lactococcus lactis subsp. lactis (strain IL1403) OX=272623 GN=rpsA PE=4 SV=1
12	1	lactococcus	Q9CHE0	1638	75775	70	70	8	8	0.56	Methionine--tRNA ligase OS=Lactococcus lactis subsp. lactis (strain IL1403) OX=272623 GN=metG PE=3 SV=1
14	1	lactococcus	Q9CEN6	914	133208	33	33	8	8	0.29	DNA-directed RNA polymerase subunit beta OS=Lactococcus lactis subsp. lactis (strain IL1403) OX=272623 GN=rpoB PE=3 SV=1
13	1	lactococcus	Q9CFC3	923	53793	25	25	7	7	0.73	Probable dipeptidase B OS=Lactococcus lactis subsp. lactis (strain IL1403) OX=272623 GN=pepDB PE=3 SV=1
31	1	lactococcus	Q9CEN7	474	135106	17	17	6	6	0.25	DNA-directed RNA polymerase subunit beta' OS=Lactococcus lactis subsp. lactis (strain IL1403) OX=272623 GN=rpoC PE=3 SV=2
15	1	lactococcus	Q9CEM7	896	45943	15	15	6	6	0.73	Peptidase T OS=Lactococcus lactis subsp. lactis (strain IL1403) OX=272623 GN=pepT PE=3 SV=1
33	1	lactococcus	Q9CH07	436	59543	16	16	6	6	0.53	Formate--tetrahydrofolate ligase OS=Lactococcus lactis subsp. lactis (strain IL1403) OX=272623 GN=fhs PE=3 SV=1
7	1	lactococcus	Q9CEV7	2547	69840	73	73	6	6	0.44	Oligoendopeptidase F homolog OS=Lactococcus lactis subsp. lactis (strain IL1403) OX=272623 GN=pepF PE=3 SV=1
34	1	lactococcus	Q04506	419	44052	18	18	6	6	0.77	RNA polymerase sigma factor SigA OS=Lactococcus lactis subsp. lactis (strain IL1403) OX=272623 GN=sigA PE=3 SV=2
20	1	lactococcus	O32797	765	89341	25	25	6	6	0.33	Formate acetyltransferase OS=Lactococcus lactis subsp. lactis (strain IL1403) OX=272623 GN=pfl PE=3 SV=1
3	1	lactococcus	Q9CG71	11972	20199	124	124	6	6	2.44	Uncharacterized protein OS=Lactococcus lactis subsp. lactis (strain IL1403) OX=272623 GN=ymgG PE=3 SV=1
16	1	lactococcus	Q9CFV2	866	118138	17	17	6	6	0.24	Carbamoyl-phosphate synthase large chain OS=Lactococcus lactis subsp. lactis (strain IL1403) OX=272623 GN=carB PE=3 SV=1
8	1	lactococcus	Q9CI09	2132	83277	53	53	6	6	0.36	ATP-dependent Clp protease ATP-binding subunit ClpE OS=Lactococcus lactis subsp. lactis (strain IL1403) OX=272623 GN=clpE PE=3 SV=1
21	1	lactococcus	Q9CDS3	732	36724	21	21	6	6	0.99	Uncharacterized protein OS=Lactococcus lactis subsp. lactis (strain IL1403) OX=272623 GN=ywaI PE=4 SV=1
40	1	lactococcus	Q9CF40	320	49702	11	11	5	5	0.53	Adenylosuccinate lyase OS=Lactococcus lactis subsp. lactis (strain IL1403) OX=272623 GN=purB PE=3 SV=1
5	1	lactococcus	Q01462	6271	35075	86	86	5	5	0.82	L-lactate dehydrogenase 1 OS=Lactococcus lactis subsp. lactis (strain IL1403) OX=272623 GN=ldh1 PE=3 SV=3
26	1	lactococcus	Q9CJD7	583	56282	10	10	5	5	0.45	Dihydrolipoamide acetyltransferase component of pyruvate dehydrogenase complex OS=Lactococcus lactis subsp. lactis (strain IL1403) OX=272623 GN=pdhC PE=3 SV=1
6	1	lactococcus	Q9CEF2	6234	18468	75	75	5	5	02.09	Uncharacterized protein OS=Lactococcus lactis subsp. lactis (strain IL1403) OX=272623 GN=ytgH PE=3 SV=1
68	1	lactococcus	Q9CEH8	134	106672	6	6	4	4	0.17	Isoleucine--tRNA ligase OS=Lactococcus lactis subsp. lactis (strain IL1403) OX=272623 GN=ileS PE=3 SV=1
37	1	lactococcus	P37283	383	10214	18	18	4	4	3.96	10 kDa chaperonin OS=Lactococcus lactis subsp. lactis (strain IL1403) OX=272623 GN=groS PE=3 SV=1
78	1	lactococcus	P58013	109	46106	5	5	4	4	0.44	Arginine deiminase OS=Lactococcus lactis subsp. lactis (strain IL1403) OX=272623 GN=arcA PE=3 SV=1
28	1	lactococcus	P81181	553	49564	14	14	4	4	0.40	Glucose-6-phosphate isomerase OS=Lactococcus lactis subsp. lactis (strain IL1403) OX=272623 GN=pgi PE=1 SV=3
46	1	lactococcus	Q9CFX8	273	44283	6	6	4	4	0.46	Uncharacterized protein OS=Lactococcus lactis subsp. lactis (strain IL1403) OX=272623 GN=ynhC PE=3 SV=1
8	2	lactococcus	Q9CFF3	607	97275	12	12	4	4	0.19	Chaperone protein ClpB OS=Lactococcus lactis subsp. lactis (strain IL1403) OX=272623 GN=clpB PE=3 SV=1
30	1	lactococcus	Q9CHB6	545	93733	16	16	4	4	0.20	Leucine--tRNA ligase OS=Lactococcus lactis subsp. lactis (strain IL1403) OX=272623 GN=leuS PE=3 SV=1

70	1	lactococcus	Q9CEX8	133	35042	3	3	3	3	0.43	EIIAB-Man OS=Lactococcus lactis subsp. lactis (strain IL1403) OX=272623 GN=ptnAB PE=4 SV=1
49	1	lactococcus	Q9CDH4	240	35889	8	8	3	3	0.42	Glyceraldehyde-3-phosphate dehydrogenase OS=Lactococcus lactis subsp. lactis (strain IL1403) OX=272623 GN=gapB PE=3 SV=1
89	1	lactococcus	Q9CDR5	80	36647	4	4	3	3	0.41	Elongation factor Ts OS=Lactococcus lactis subsp. lactis (strain IL1403) OX=272623 GN=tsf PE=3 SV=1
2	2	lactococcus	Q9CIT0	3540	45781	81	81	3	3	0.32	Enolase 2 OS=Lactococcus lactis subsp. lactis (strain IL1403) OX=272623 GN=eno2 PE=3 SV=1
65	1	lactococcus	Q9CHG7	143	42878	5	5	3	3	0.34	Transcription termination/antitermination protein NusA OS=Lactococcus lactis subsp. lactis (strain IL1403) OX=272623 GN=nusA PE=3 SV=1
38	1	lactococcus	Q9CEX2	359	47531	14	14	3	3	0.30	Serine--tRNA ligase OS=Lactococcus lactis subsp. lactis (strain IL1403) OX=272623 GN=serS PE=3 SV=1
84	1	lactococcus	Q9CHE1	87	38145	3	3	3	3	0.39	Exodeoxyribonuclease III OS=Lactococcus lactis subsp. lactis (strain IL1403) OX=272623 GN=exoA PE=3 SV=1
18	1	lactococcus	Q9CI15	809	46902	15	15	3	3	0.31	Trigger factor OS=Lactococcus lactis subsp. lactis (strain IL1403) OX=272623 GN=tig PE=3 SV=1
75	1	lactococcus	Q9CEG3	125	49975	5	5	3	3	0.29	Aminopeptidase C OS=Lactococcus lactis subsp. lactis (strain IL1403) OX=272623 GN=pepC PE=3 SV=3
56	1	lactococcus	Q9CH05	198	30636	6	6	3	3	0.51	Amino acid ABC transporter substrate binding protein OS=Lactococcus lactis subsp. lactis (strain IL1403) OX=272623 GN=yjgC PE=4 SV=1
11	1	lactococcus	Q9CDL9	1666	49800	34	34	3	3	0.29	Glutamine synthetase OS=Lactococcus lactis subsp. lactis (strain IL1403) OX=272623 GN=glnA PE=3 SV=1
42	1	lactococcus	Q9CIQ1	308	95414	6	6	3	3	0.14	Aminopeptidase N OS=Lactococcus lactis subsp. lactis (strain IL1403) OX=272623 GN=pepN PE=3 SV=1
19	1	lactococcus	Q9CFW7	789	29135	14	14	3	3	0.54	Dihydroorotate dehydrogenase B (NAD(+)), electron transfer subunit OS=Lactococcus lactis subsp. lactis (strain IL1403) OX=272623 GN=pyrK PE=1 SV=1
51	1	lactococcus	Q9CH83	222	8959	8	8	3	3	2.88	Exodeoxyribonuclease 7 small subunit OS=Lactococcus lactis subsp. lactis (strain IL1403) OX=272623 GN=xseB PE=3 SV=1
10	1	lactococcus	Q07637	1747	54267	22	22	3	3	0.47	Pyruvate kinase OS=Lactococcus lactis subsp. lactis (strain IL1403) OX=272623 GN=pyk PE=3 SV=2
39	1	lactococcus	Q9CEI0	350	43185	9	9	3	3	0.48	Elongation factor Tu OS=Lactococcus lactis subsp. lactis (strain IL1403) OX=272623 GN=tuf PE=3 SV=1
25	1	lactococcus	Q9CGY9	587	20568	14	14	3	3	0.84	Protein GrpE OS=Lactococcus lactis subsp. lactis (strain IL1403) OX=272623 GN=grpE PE=3 SV=1
59	1	lactococcus	Q9CH10	175	25411	8	8	3	3	0.64	Purine nucleoside phosphorylase DeoD-type OS=Lactococcus lactis subsp. lactis (strain IL1403) OX=272623 GN=deoD PE=3 SV=1
22	1	lactococcus	Q9CFJ0	720	56837	12	12	3	3	0.25	GMP synthase [glutamine-hydrolyzing] OS=Lactococcus lactis subsp. lactis (strain IL1403) OX=272623 GN=guaA PE=3 SV=1
52	1	lactococcus	Q9CG80	206	81197	5	5	3	3	0.17	DNA topoisomerase 1 OS=Lactococcus lactis subsp. lactis (strain IL1403) OX=272623 GN=topA PE=3 SV=1
29	1	lactococcus	Q9CJ82	553	62613	13	13	3	3	0.22	Phosphoenolpyruvate-protein phosphotransferase OS=Lactococcus lactis subsp. lactis (strain IL1403) OX=272623 GN=ptsI PE=3 SV=1
57	1	lactococcus	Q9CJJ1	191	42238	6	6	3	3	0.35	Beta sliding clamp OS=Lactococcus lactis subsp. lactis (strain IL1403) OX=272623 GN=dnaN PE=3 SV=1
53	1	lactococcus	Q9CDT4	205	69254	9	9	3	3	0.20	Proline--tRNA ligase OS=Lactococcus lactis subsp. lactis (strain IL1403) OX=272623 GN=proS PE=3 SV=1
60	1	lactococcus	Q9CJ45	172	29202	7	7	3	3	0.54	Transcriptional regulator OS=Lactococcus lactis subsp. lactis (strain IL1403) OX=272623 GN=codY PE=3 SV=1
96	1	lactococcus	Q9CE01	71	87733	2	2	2	2	0.10	Xaa-Pro dipeptidyl-peptidase OS=Lactococcus lactis subsp. lactis (strain IL1403) OX=272623 GN=pepX PE=3 SV=1
17	1	lactococcus	Q9CIM0	857	26313	16	16	2	2	0.37	2,3-bisphosphoglycerate-dependent phosphoglycerate mutase OS=Lactococcus lactis subsp. lactis (strain IL1403) OX=272623 GN=gpmA PE=3 SV=1
93	1	lactococcus	Q9CE93	74	47480	2	2	2	2	0.19	Adenylosuccinate synthetase OS=Lactococcus lactis subsp. lactis (strain IL1403) OX=272623 GN=purA PE=3 SV=1
23	1	lactococcus	Q9CG42	662	12401	15	15	2	2	0.94	50S ribosomal protein L7/L12 OS=Lactococcus lactis subsp. lactis (strain IL1403) OX=272623 GN=rplL PE=3 SV=1
118	1	lactococcus	Q9CEE0	49	43037	2	2	2	2	0.22	S-adenosylmethionine synthase OS=Lactococcus lactis subsp. lactis (strain IL1403) OX=272623 GN=metK PE=3 SV=1
32	1	lactococcus	Q9CDG1	457	77907	8	8	2	2	0.11	Elongation factor G OS=Lactococcus lactis subsp. lactis (strain IL1403) OX=272623 GN=fusA PE=3 SV=1
62	1	lactococcus	Q9CDZ7	164	55427	5	5	2	2	0.16	Glutamate--tRNA ligase OS=Lactococcus lactis subsp. lactis (strain IL1403) OX=272623 GN=gltX PE=3 SV=1
35	1	lactococcus	Q9CHB9	388	51036	8	8	2	2	0.18	Signal recognition particle receptor FtsY OS=Lactococcus lactis subsp. lactis (strain IL1403) OX=272623 GN=ftsY PE=3 SV=1
104	1	lactococcus	Q9CEB5	62	87280	2	2	2	2	0.10	Phenylalanine--tRNA ligase beta subunit OS=Lactococcus lactis subsp. lactis (strain IL1403) OX=272623 GN=pheT PE=3 SV=1

47	1	lactococcus	Q9CHU6	262	52484	6	6	2	2	0.17	6-phosphogluconate dehydrogenase, decarboxylating OS=Lactococcus lactis subsp. lactis (strain IL1403) OX=272623 GN=gnd PE=3 SV=1
79	1	lactococcus	Q9CF22	100	35326	4	4	2	2	0.27	Phosphate acetyltransferase OS=Lactococcus lactis subsp. lactis (strain IL1403) OX=272623 GN=pta PE=3 SV=1
45	1	lactococcus	Q9CJD8	291	49928	8	8	2	2	0.18	Dihydrolipoyl dehydrogenase OS=Lactococcus lactis subsp. lactis (strain IL1403) OX=272623 GN=pdhD PE=3 SV=1
127	1	lactococcus	Q9CF20	44	36267	2	2	2	2	0.26	Oxidoreductase OS=Lactococcus lactis subsp. lactis (strain IL1403) OX=272623 GN=yrbA PE=4 SV=1
55	1	lactococcus	Q9CJD5	198	41299	7	7	2	2	0.23	Pyruvate dehydrogenase E1 component subunit alpha OS=Lactococcus lactis subsp. lactis (strain IL1403) OX=272623 GN=pdhA PE=4 SV=1
72	1	lactococcus	Q9CDY3	131	34173	5	5	2	2	0.28	DNA-directed RNA polymerase subunit alpha OS=Lactococcus lactis subsp. lactis (strain IL1403) OX=272623 GN=rpoA PE=3 SV=1
36	1	lactococcus	Q9CF56	387	71681	6	6	2	2	0.13	Transketolase OS=Lactococcus lactis subsp. lactis (strain IL1403) OX=272623 GN=tkt PE=3 SV=1
98	1	lactococcus	Q9CIU0	70	17686	2	2	2	2	0.60	S-ribosylhomocysteine lyase OS=Lactococcus lactis subsp. lactis (strain IL1403) OX=272623 GN=luxS PE=3 SV=1
48	1	lactococcus	Q9CE23	255	16642	5	5	2	2	0.65	Non-heme iron-binding ferritin OS=Lactococcus lactis subsp. lactis (strain IL1403) OX=272623 GN=dpsA PE=3 SV=1
87	1	lactococcus	Q9CFW8	81	33125	2	2	2	2	0.29	Dihydroorotate dehydrogenase B (NAD(+)), catalytic subunit OS=Lactococcus lactis subsp. lactis (strain IL1403) OX=272623 GN=pyrDB PE=1 SV=1
58	1	lactococcus	Q9CDP6	187	100475	4	4	2	2	0.09	Valine--tRNA ligase OS=Lactococcus lactis subsp. lactis (strain IL1403) OX=272623 GN=valS PE=3 SV=1
121	1	lactococcus	Q9CH36	47	72551	2	2	2	2	0.12	DNA gyrase subunit B OS=Lactococcus lactis subsp. lactis (strain IL1403) OX=272623 GN=gyrB PE=3 SV=1
49	2	lactococcus	P52987	50	36080	2	2	2	2	0.26	Glyceraldehyde-3-phosphate dehydrogenase OS=Lactococcus lactis subsp. lactis (strain IL1403) OX=272623 GN=gap PE=3 SV=2
66	1	lactococcus	Q9CIY6	138	52839	5	5	2	2	0.17	Inosine-5'-monophosphate dehydrogenase OS=Lactococcus lactis subsp. lactis (strain IL1403) OX=272623 GN=guaB PE=3 SV=1
61	1	lactococcus	Q9CE96	168	21380	7	7	2	2	0.48	Uncharacterized protein OS=Lactococcus lactis subsp. lactis (strain IL1403) OX=272623 GN=yucF PE=4 SV=1
106	1	lactococcus	Q9CF28	59	14970	3	3	2	2	0.74	Uncharacterized protein OS=Lactococcus lactis subsp. lactis (strain IL1403) OX=272623 GN=yraB PE=4 SV=1
85	1	lactococcus	Q9CHU7	85	20647	2	2	2	2	0.50	Uncharacterized protein OS=Lactococcus lactis subsp. lactis (strain IL1403) OX=272623 GN=ygaJ PE=4 SV=1
128	1	lactococcus	Q9CHF2	42	31934	2	2	2	2	0.30	Acetyl-coenzyme A carboxylase carboxyl transferase subunit beta OS=Lactococcus lactis subsp. lactis (strain IL1403) OX=272623 GN=accD PE=3 SV=1
76	1	lactococcus	Q9CIR6	122	61569	5	5	2	2	0.15	Ribonuclease J OS=Lactococcus lactis subsp. lactis (strain IL1403) OX=272623 GN=yciH PE=3 SV=1
112	1	lactococcus	Q9CDR3	53	98298	2	2	2	2	0.09	Aldehyde-alcohol dehydrogenase OS=Lactococcus lactis subsp. lactis (strain IL1403) OX=272623 GN=adhE PE=3 SV=1
64	1	lactococcus	Q9CHQ7	150	126649	5	5	2	2	0.07	Pyruvate carboxylase OS=Lactococcus lactis subsp. lactis (strain IL1403) OX=272623 GN=pycA PE=4 SV=1
77	1	lactococcus	P0C2U0	122	39477	3	3	2	2	0.24	Ornithine carbamoyltransferase, catabolic OS=Lactococcus lactis subsp. lactis (strain IL1403) OX=272623 GN=arcB PE=3 SV=1
24	1	lactococcus	Q9CEF7	657	27955	16	16	1	1	0.16	Pyrroline-5-carboxylate reductase OS=Lactococcus lactis subsp. lactis (strain IL1403) OX=272623 GN=proC PE=3 SV=1
27	1	lactococcus	Q9CI02	576	26458	11	11	1	1	0.17	Enoyl-[acyl-carrier-protein] reductase [NADH] OS=Lactococcus lactis subsp. lactis (strain IL1403) OX=272623 GN=fabI PE=3 SV=1
114	1	lactococcus	Q9CIP3	52	8399	1	1	1	1	0.62	PC4 domain-containing protein OS=Lactococcus lactis subsp. lactis (strain IL1403) OX=272623 GN=ydbC PE=1 SV=1
41	1	lactococcus	Q9CED6	315	41036	6	6	1	1	0.11	DUF4097 domain-containing protein OS=Lactococcus lactis subsp. lactis (strain IL1403) OX=272623 GN=ythC PE=4 SV=1
90	1	lactococcus	Q9CES5	80	78328	11	11	1	1	0.06	Glutamine ABC transporter permease and substrate binding protein protein OS=Lactococcus lactis subsp. lactis (strain IL1403) OX=272623 GN=glnP PE=1 SV=1
43	1	lactococcus	Q9CHT4	296	21374	6	6	1	1	0.21	Probable DNA-directed RNA polymerase subunit delta OS=Lactococcus lactis subsp. lactis (strain IL1403) OX=272623 GN=rpoE PE=3 SV=1
94	1	lactococcus	Q9CH92	74	47795	3	3	1	1	0.09	Glutathione reductase OS=Lactococcus lactis subsp. lactis (strain IL1403) OX=272623 GN=gshR PE=3 SV=1
50	1	lactococcus	Q9CHF9	225	8396	5	5	1	1	0.62	Acyl carrier protein OS=Lactococcus lactis subsp. lactis (strain IL1403) OX=272623 GN=acpP PE=3 SV=1

67	1	lactococcus	Q9CIU1	136	40276	3	3	1	1	0.11	Oxidoreductase OS=Lactococcus lactis subsp. lactis (strain IL1403) OX=272623 GN=ycgD PE=4 SV=1
44	1	lactococcus	Q9CEF8	295	49195	3	3	1	1	0.09	Bifunctional protein GlmU OS=Lactococcus lactis subsp. lactis (strain IL1403) OX=272623 GN=glmU PE=3 SV=1
122	1	lactococcus	Q9CHK0	46	62531	2	2	1	1	0.07	2-succinyl-5-enolpyruvyl-6-hydroxy-3-cyclohexene-1-carboxylate synthase OS=Lactococcus lactis subsp. lactis (strain IL1403) OX=272623 GN=menD PE=3 SV=1
54	1	lactococcus	Q9CHF6	199	42631	5	5	1	1	0.10	3-oxoacyl-[acyl-carrier-protein] synthase 2 OS=Lactococcus lactis subsp. lactis (strain IL1403) OX=272623 GN=fabF PE=3 SV=1
80	1	lactococcus	Q9CEK0	97	66050	3	3	1	1	0.07	Oligopeptide-binding protein OppA OS=Lactococcus lactis subsp. lactis (strain IL1403) OX=272623 GN=oppA PE=3 SV=1
100	1	lactococcus	Q9CG41	66	18076	1	1	1	1	0.26	50S ribosomal protein L10 OS=Lactococcus lactis subsp. lactis (strain IL1403) OX=272623 GN=rplJ PE=3 SV=1
71	1	lactococcus	Q9CI76	132	42237	2	2	1	1	0.10	Amino acids and amines aminotransferase OS=Lactococcus lactis subsp. lactis (strain IL1403) OX=272623 GN=yeiG PE=3 SV=1
116	1	lactococcus	Q9CHQ3	50	22068	2	2	1	1	0.21	ATP-dependent Clp protease proteolytic subunit OS=Lactococcus lactis subsp. lactis (strain IL1403) OX=272623 GN=clpP PE=3 SV=1
86	1	lactococcus	Q9CJ43	84	52039	1	1	1	1	0.08	Glutamyl-tRNA(Gln) amidotransferase subunit A OS=Lactococcus lactis subsp. lactis (strain IL1403) OX=272623 GN=gatA PE=3 SV=1
102	1	lactococcus	Q9CIH3	63	38339	2	2	1	1	0.12	Glutamyl aminopeptidase OS=Lactococcus lactis subsp. lactis (strain IL1403) OX=272623 GN=pepA PE=3 SV=1
63	1	lactococcus	Q9CGT1	156	20738	4	4	1	1	0.22	HTH cro/C1-type domain-containing protein OS=Lactococcus lactis subsp. lactis (strain IL1403) OX=272623 GN=pi204 PE=4 SV=1
124	1	lactococcus	Q9CFD0	46	27521	1	1	1	1	0.16	Uncharacterized protein OS=Lactococcus lactis subsp. lactis (strain IL1403) OX=272623 GN=ypjC PE=4 SV=1
82	1	lactococcus	P0A4K2	92	40912	4	4	1	1	0.11	Cystathionine beta-lyase OS=Lactococcus lactis subsp. lactis (strain IL1403) OX=272623 GN=metC PE=3 SV=1
95	1	lactococcus	Q9CHF7	71	25582	2	2	1	1	0.18	3-oxoacyl-[acyl-carrier-protein] reductase OS=Lactococcus lactis subsp. lactis (strain IL1403) OX=272623 GN=fabG1 PE=3 SV=1
73	1	lactococcus	Q9CIS4	130	43165	3	3	1	1	0.10	N-acetyldiaminopimelate deacetylase OS=Lactococcus lactis subsp. lactis (strain IL1403) OX=272623 GN=yciA PE=3 SV=1
119	1	lactococcus	Q9CGL7	49	11430	2	2	1	1	0.43	50S ribosomal protein L21 OS=Lactococcus lactis subsp. lactis (strain IL1403) OX=272623 GN=rplU PE=3 SV=1
91	1	lactococcus	Q9CF33	75	36624	1	1	1	1	0.12	Catabolite control protein A OS=Lactococcus lactis subsp. lactis (strain IL1403) OX=272623 GN=ccpA PE=1 SV=1
103	1	lactococcus	Q9CF31	63	72305	1	1	1	1	0.06	Mid-cell-anchored protein Z OS=Lactococcus lactis subsp. lactis (strain IL1403) OX=272623 GN=yqjD PE=3 SV=1
69	1	lactococcus	Q9CGM2	133	13660	6	6	1	1	0.35	Fe-S_biosyn domain-containing protein OS=Lactococcus lactis subsp. lactis (strain IL1403) OX=272623 GN=ykiE PE=4 SV=1
126	1	lactococcus	Q9CEY2	44	52517	1	1	1	1	0.08	Transcription regulator OS=Lactococcus lactis subsp. lactis (strain IL1403) OX=272623 GN=yrfE PE=3 SV=1
81	1	lactococcus	P0DOB5	93	35829	2	2	1	1	0.12	ATP-dependent 6-phosphofructokinase OS=Lactococcus lactis subsp. lactis (strain IL1403) OX=272623 GN=pfkA PE=3 SV=1
99	1	lactococcus	Q9CJI9	68	138639	1	1	1	1	0.03	ATP-dependent helicase/nuclease subunit A OS=Lactococcus lactis subsp. lactis (strain IL1403) OX=272623 GN=addA PE=3 SV=1
74	1	lactococcus	Q9CHB8	127	35615	2	2	1	1	0.27	Ribose-phosphate pyrophosphokinase 1 OS=Lactococcus lactis subsp. lactis (strain IL1403) OX=272623 GN=prs1 PE=3 SV=1
115	1	lactococcus	Q9CI23	50	71891	1	1	1	1	0.06	DD-transpeptidase OS=Lactococcus lactis subsp. lactis (strain IL1403) OX=272623 GN=ponA PE=4 SV=1
88	1	lactococcus	Q9CE12	80	62780	1	1	1	1	0.07	Arginine--tRNA ligase OS=Lactococcus lactis subsp. lactis (strain IL1403) OX=272623 GN=argS PE=3 SV=1
107	1	lactococcus	Q9CDK3	58	12991	2	2	1	1	0.37	Uncharacterized protein OS=Lactococcus lactis subsp. lactis (strain IL1403) OX=272623 GN=ywjG PE=4 SV=1
83	1	lactococcus	Q9CE11	90	16929	1	1	1	1	0.28	Arginine repressor OS=Lactococcus lactis subsp. lactis (strain IL1403) OX=272623 GN=argR PE=3 SV=1
123	1	lactococcus	Q9CFN1	46	59188	2	2	1	1	0.07	Histidine kinase OS=Lactococcus lactis subsp. lactis (strain IL1403) OX=272623 GN=kinB PE=4 SV=1
92	1	lactococcus	Q9CEJ9	75	13657	3	3	1	1	0.35	50S ribosomal protein L20 OS=Lactococcus lactis subsp. lactis (strain IL1403) OX=272623 GN=rplT PE=3 SV=1

97	1	lactococcus	Q9CED4	71	32153	4	4	1	1	0.14	Fructose-bisphosphate aldolase OS=Lactococcus lactis subsp. lactis (strain IL1403) OX=272623 GN=fbaA PE=4 SV=1
120	1	lactococcus	Q9CGB8	49	58771	1	1	1	1	0.07	Uncharacterized protein OS=Lactococcus lactis subsp. lactis (strain IL1403) OX=272623 GN=yljF PE=4 SV=1
105	1	lactococcus	Q9CGF1	61	35952	1	1	1	1	0.12	GMP reductase OS=Lactococcus lactis subsp. lactis (strain IL1403) OX=272623 GN=guaC PE=3 SV=1
129	1	lactococcus	Q9CI10	41	13213	2	2	1	1	0.37	Uncharacterized protein OS=Lactococcus lactis subsp. lactis (strain IL1403) OX=272623 GN=yffA PE=4 SV=1
101	1	lactococcus	Q9CHI4	64	9828	1	1	1	1	0.51	Uncharacterized protein OS=Lactococcus lactis subsp. lactis (strain IL1403) OX=272623 GN=yhfC PE=4 SV=1
117	1	lactococcus	Q9CIW1	49	42044	1	1	1	1	0.11	Phosphoglycerate kinase OS=Lactococcus lactis subsp. lactis (strain IL1403) OX=272623 GN=pgk PE=3 SV=1
113	1	lactococcus	Q9CJD6	52	35188	2	2	1	1	0.13	PDH E1 component beta subunit OS=Lactococcus lactis subsp. lactis (strain IL1403) OX=272623 GN=pdhB PE=4 SV=1
125	1	lactococcus	P0A3Z9	46	30388	1	1	1	1	0.15	Pur operon repressor OS=Lactococcus lactis subsp. lactis (strain IL1403) OX=272623 GN=purR PE=3 SV=1
108	1	lactococcus	Q9CEH7	57	34931	1	1	1	1	0.13	Uncharacterized protein OS=Lactococcus lactis subsp. lactis (strain IL1403) OX=272623 GN=ytdB PE=4 SV=1
130	1	lactococcus	Q9CIG1	40	12717	1	1	1	1	0.38	Initiation-control protein YabA OS=Lactococcus lactis subsp. lactis (strain IL1403) OX=272623 GN=yeaD PE=3 SV=1
109	1	lactococcus	Q9CF73	56	45490	1	1	1	1	0.10	Gamma-glutamyl phosphate reductase OS=Lactococcus lactis subsp. lactis (strain IL1403) OX=272623 GN=proA PE=3 SV=1
131	1	lactococcus	Q9CG78	40	41372	1	1	1	1	0.11	Tyrosine recombinase XerS OS=Lactococcus lactis subsp. lactis (strain IL1403) OX=272623 GN=xerS PE=3 SV=1
110	1	lactococcus	Q9CEJ7	55	20545	1	1	1	1	0.22	Translation initiation factor IF-3 OS=Lactococcus lactis subsp. lactis (strain IL1403) OX=272623 GN=infC PE=3 SV=1
132	1	lactococcus	Q9CIJ5	38	24924	1	1	1	1	0.18	RelA_SpoT domain-containing protein OS=Lactococcus lactis subsp. lactis (strain IL1403) OX=272623 GN=ydgI PE=4 SV=1
111	1	lactococcus	Q9CIH5	54	47221	1	1	1	1	0.09	Tyrosine--tRNA ligase OS=Lactococcus lactis subsp. lactis (strain IL1403) OX=272623 GN=tyrS PE=3 SV=1
133	1	lactococcus	Q9CF30	37	43307	1	1	1	1	0.10	THUMP domain-containing protein OS=Lactococcus lactis subsp. lactis (strain IL1403) OX=272623 GN=yqjE PE=4 SV=1
134	1	lactococcus	Q9CIF5	37	38708	1	1	1	1	0.11	Isopentenyl-diphosphate delta-isomerase OS=Lactococcus lactis subsp. lactis (strain IL1403) OX=272623 GN=fni PE=3 SV=1
135	1	lactococcus	Q9CG14	37	27690	1	1	1	1	0.16	Abhydrolase_5 domain-containing protein OS=Lactococcus lactis subsp. lactis (strain IL1403) OX=272623 GN=yndB PE=4 SV=1
136	1	lactococcus	Q9CGH2	36	52866	1	1	1	1	0.08	Fumarate reductase flavoprotein subunit OS=Lactococcus lactis subsp. lactis (strain IL1403) OX=272623 GN=frdC PE=3 SV=1
137	1	lactococcus	Q9CGQ2	36	14050	2	2	1	1	0.34	Prophage pi2 protein 37 OS=Lactococcus lactis subsp. lactis (strain IL1403) OX=272623 GN=pi237 PE=4 SV=1
138	1	lactococcus	Q9CEK5	36	89889	1	1	1	1	0.05	3'-5' exonuclease DinG OS=Lactococcus lactis subsp. lactis (strain IL1403) OX=272623 GN=dinG PE=3 SV=1
139	1	lactococcus	Q9CE40	36	18150	1	1	1	1	0.26	N-acetyltransferase domain-containing protein OS=Lactococcus lactis subsp. lactis (strain IL1403) OX=272623 GN=yuiD PE=4 SV=1
140	1	lactococcus	Q9CFM9	36	36722	1	1	1	1	0.12	Basic membrane protein A OS=Lactococcus lactis subsp. lactis (strain IL1403) OX=272623 GN=bmpA PE=3 SV=1
141	1	lactococcus	Q9CF87	36	26668	1	1	1	1	0.17	Two-component system regulator OS=Lactococcus lactis subsp. lactis (strain IL1403) OX=272623 GN=llrA PE=4 SV=1
142	1	lactococcus	Q9CIV3	35	24332	1	1	1	1	0.19	Transcriptional regulator OS=Lactococcus lactis subsp. lactis (strain IL1403) OX=272623 GN=ycfA PE=4 SV=1
143	1	lactococcus	Q9CH33	34	17714	1	1	1	1	0.26	Transcriptional regulator OS=Lactococcus lactis subsp. lactis (strain IL1403) OX=272623 GN=rmaH PE=4 SV=1
144	1	lactococcus	Q9CIH4	34	86912	1	1	1	1	0.05	DD-transpeptidase OS=Lactococcus lactis subsp. lactis (strain IL1403) OX=272623 GN=pbp1B PE=4 SV=1
145	1	lactococcus	Q9CFZ7	34	72033	1	1	1	1	0.06	Aminodeoxychorismate synthase OS=Lactococcus lactis subsp. lactis (strain IL1403) OX=272623 GN=pabB PE=3 SV=1
146	1	lactococcus	Q9CHF1	33	28529	1	1	1	1	0.16	Acetyl-CoA carboxytransferase OS=Lactococcus lactis subsp. lactis (strain IL1403) OX=272623 GN=accA PE=4 SV=1
147	1	lactococcus	Q9CDJ2	33	72280	1	1	1	1	0.06	Asparagine synthetase OS=Lactococcus lactis subsp. lactis (strain IL1403) OX=272623 GN=asnH PE=3 SV=1

148	1	lactococcus	Q01998	33	43856	1	1	1	1	0.10	Tryptophan synthase beta chain OS=Lactococcus lactis subsp. lactis (strain IL1403) OX=272623 GN=trpB PE=3 SV=1
149	1	lactococcus	Q9CGF0	33	21793	1	1	1	1	0.21	Xanthine phosphoribosyltransferase OS=Lactococcus lactis subsp. lactis (strain IL1403) OX=272623 GN=xpt PE=3 SV=1
150	1	lactococcus	Q9CH94	33	40847	1	1	1	1	0.11	Alanine racemase OS=Lactococcus lactis subsp. lactis (strain IL1403) OX=272623 GN=alr PE=3 SV=1
151	1	lactococcus	Q9CHM9	32	54422	1	1	1	1	0.08	Glycogen synthase OS=Lactococcus lactis subsp. lactis (strain IL1403) OX=272623 GN=glgA PE=3 SV=2
152	1	lactococcus	Q9CH49	31	36752	1	1	1	1	0.12	Sensor histidine kinase OS=Lactococcus lactis subsp. lactis (strain IL1403) OX=272623 GN=kinD PE=4 SV=1
153	1	lactococcus	Q9CDH8	30	49938	1	1	1	1	0.09	tRNA modification GTPase MnmE OS=Lactococcus lactis subsp. lactis (strain IL1403) OX=272623 GN=mnmE PE=3 SV=1
154	1	lactococcus	Q9CJ12	30	32110	1	1	1	1	0.14	Glucose-1-phosphate thymidylyltransferase OS=Lactococcus lactis subsp. lactis (strain IL1403) OX=272623 GN=rmlA PE=3 SV=1

Proteins identified in active fractions obtained from Superdex 200 column

Family	Member	Database	Accession	Score	Mass	Num. of matches	Num. of significant matches	Num. of sequences	Num. of significant sequences	emPAI	Description
1	1	lactococcus	P0A3J0	67022	64947	1649	1647	184	184	431056.72	Chaperone protein DnaK OS=Lactococcus lactis subsp. lactis (strain IL1403) OX=272623 GN=dnaK PE=3 SV=1
2	1	lactococcus	Q9CDJ1	54418	47025	1097	1095	87	87	3696.51	Peptidase C51 domain-containing protein OS=Lactococcus lactis subsp. lactis (strain IL1403) OX=272623 GN=usp45 PE=4 SV=1
3	1	lactococcus	Q9CIW1	19897	42044	436	434	55	55	294.52	Phosphoglycerate kinase OS=Lactococcus lactis subsp. lactis (strain IL1403) OX=272623 GN=pgk PE=3 SV=1
4	1	lactococcus	Q9CF79	18634	34674	545	545	37	37	686.91	Aspartate carbamoyltransferase OS=Lactococcus lactis subsp. lactis (strain IL1403) OX=272623 GN=pyrB PE=3 SV=1
5	1	lactococcus	Q9CIM0	17195	26313	546	546	54	54	9952.40	2,3-bisphosphoglycerate-dependent phosphoglycerate mutase OS=Lactococcus lactis subsp. lactis (strain IL1403) OX=272623 GN=gpmA PE=3 SV=1
6	1	lactococcus	P37283	16602	10214	460	460	20	20	9803.60	10 kDa chaperonin OS=Lactococcus lactis subsp. lactis (strain IL1403) OX=272623 GN=groS PE=3 SV=1
7	1	lactococcus	Q01462	15034	35075	411	411	58	58	1461.96	L-lactate dehydrogenase 1 OS=Lactococcus lactis subsp. lactis (strain IL1403) OX=272623 GN=ldh1 PE=3 SV=3
8	1	lactococcus	Q9CEM7	14335	45943	406	406	57	57	658.84	Peptidase T OS=Lactococcus lactis subsp. lactis (strain IL1403) OX=272623 GN=pepT PE=3 SV=1
9	1	lactococcus	Q9CHS7	13002	46929	327	327	44	44	86.83	Enolase 1 OS=Lactococcus lactis subsp. lactis (strain IL1403) OX=272623 GN=eno1 PE=3 SV=1
9	2	lactococcus	Q9CIT0	306	45781	5	5	2	2	0.20	Enolase 2 OS=Lactococcus lactis subsp. lactis (strain IL1403) OX=272623 GN=eno2 PE=3 SV=1
10	1	lactococcus	Q9CI15	11858	46902	310	310	44	44	95.05	Trigger factor OS=Lactococcus lactis subsp. lactis (strain IL1403) OX=272623 GN=tig PE=3 SV=1
11	1	lactococcus	Q9CEX8	10638	35042	325	325	51	51	2092.63	EIIAB-Man OS=Lactococcus lactis subsp. lactis (strain IL1403) OX=272623 GN=ptnAB PE=4 SV=1
12	1	lactococcus	Q9CHU6	9846	52484	262	262	53	53	121.36	6-phosphogluconate dehydrogenase, decarboxylating OS=Lactococcus lactis subsp. lactis (strain IL1403) OX=272623 GN=gnd PE=3 SV=1
13	1	lactococcus	Q9CEX2	9312	47531	273	272	48	47	140.79	Serine--tRNA ligase OS=Lactococcus lactis subsp. lactis (strain IL1403) OX=272623 GN=serS PE=3 SV=1
14	1	lactococcus	Q9CED4	9263	32153	272	272	36	36	180.72	Fructose-bisphosphate aldolase OS=Lactococcus lactis subsp. lactis (strain IL1403) OX=272623 GN=fbaA PE=4 SV=1
15	1	lactococcus	Q07637	9126	54267	229	229	40	40	47.22	Pyruvate kinase OS=Lactococcus lactis subsp. lactis (strain IL1403) OX=272623 GN=pyk PE=3 SV=2
16	1	lactococcus	Q9CJ82	9110	62613	267	267	53	53	109.93	Phosphoenolpyruvate-protein phosphotransferase OS=Lactococcus lactis subsp. lactis (strain IL1403) OX=272623 GN=ptsI PE=3 SV=1
17	1	lactococcus	P0DOB5	7840	35829	237	237	36	36	94.56	ATP-dependent 6-phosphofructokinase OS=Lactococcus lactis subsp. lactis (strain IL1403) OX=272623 GN=pfkA PE=3 SV=1
18	1	lactococcus	Q9CFJ0	7519	56837	198	198	40	40	33.98	GMP synthase [glutamine-hydrolyzing] OS=Lactococcus lactis subsp. lactis (strain IL1403) OX=272623 GN=guaA PE=3 SV=1
19	1	lactococcus	Q9CI09	7430	83277	204	204	46	46	12.94	ATP-dependent Clp protease ATP-binding subunit ClpE OS=Lactococcus lactis subsp. lactis (strain IL1403) OX=272623 GN=clpE PE=3 SV=1
19	2	lactococcus	Q9CFF3	3749	97275	111	110	35	35	45065	Chaperone protein ClpB OS=Lactococcus lactis subsp. lactis (strain IL1403) OX=272623 GN=clpB PE=3 SV=1
20	1	lactococcus	P37282	7070	57166	170	169	45	45	41.73	60 kDa chaperonin OS=Lactococcus lactis subsp. lactis (strain IL1403) OX=272623 GN=groL PE=3 SV=2
21	1	lactococcus	Q9CIQ1	7011	95414	231	231	51	51	10.38	Aminopeptidase N OS=Lactococcus lactis subsp. lactis (strain IL1403) OX=272623 GN=pepN PE=3 SV=1
22	1	lactococcus	Q9CE01	6957	87733	227	226	56	55	16.09	Xaa-Pro dipeptidyl-peptidase OS=Lactococcus lactis subsp. lactis (strain IL1403) OX=272623 GN=pepX PE=3 SV=1
23	1	lactococcus	Q9CEV7	6619	69840	205	205	38	38	10.88	Oligoendopeptidase F homolog OS=Lactococcus lactis subsp. lactis (strain IL1403) OX=272623 GN=pepF PE=3 SV=1
24	1	lactococcus	Q9CDH4	6540	35889	188	188	44	44	306.65	Glyceraldehyde-3-phosphate dehydrogenase OS=Lactococcus lactis subsp. lactis (strain IL1403) OX=272623 GN=gapB PE=3 SV=1
24	2	lactococcus	P52987	2743	36080	92	92	22	22	17.27	Glyceraldehyde-3-phosphate dehydrogenase OS=Lactococcus lactis subsp. lactis (strain IL1403) OX=272623 GN=gap PE=3 SV=2

25	1	lactococcus	Q9CHA0	6370	44683	188	188	42	42	74.18	30S ribosomal protein S1 OS=Lactococcus lactis subsp. lactis (strain IL1403) OX=272623 GN=rpsA PE=4 SV=1
26	1	lactococcus	P58013	6263	46106	170	169	28	28	28.23	Arginine deiminase OS=Lactococcus lactis subsp. lactis (strain IL1403) OX=272623 GN=arcA PE=3 SV=1
27	1	lactococcus	Q9CDG1	6224	77907	198	198	41	41	18.61	Elongation factor G OS=Lactococcus lactis subsp. lactis (strain IL1403) OX=272623 GN=fusA PE=3 SV=1
28	1	lactococcus	Q9CJD8	6180	49928	159	159	34	34	21.52	Dihydrolipoyl dehydrogenase OS=Lactococcus lactis subsp. lactis (strain IL1403) OX=272623 GN=pdhD PE=3 SV=1
29	1	lactococcus	P81181	6158	49564	200	200	37	37	26.24	Glucose-6-phosphate isomerase OS=Lactococcus lactis subsp. lactis (strain IL1403) OX=272623 GN=pgi PE=1 SV=3
30	1	lactococcus	Q9CDZ7	6156	55427	197	197	41	41	29.53	Glutamate--tRNA ligase OS=Lactococcus lactis subsp. lactis (strain IL1403) OX=272623 GN=gltX PE=3 SV=1
30	2	lactococcus	Q9CET0	77	23367	5	5	2	2	0.43	UPF0637 protein YsbB OS=Lactococcus lactis subsp. lactis (strain IL1403) OX=272623 GN=ysbB PE=3 SV=1
31	1	lactococcus	Q9CE23	6135	16642	167	167	22	22	499.82	Non-heme iron-binding ferritin OS=Lactococcus lactis subsp. lactis (strain IL1403) OX=272623 GN=dpsA PE=3 SV=1
32	1	lactococcus	Q9CI64	5846	9670	184	183	23	23	16286.02	DNA-binding protein HU OS=Lactococcus lactis subsp. lactis (strain IL1403) OX=272623 GN=hup PE=1 SV=1
33	1	lactococcus	Q9CF56	5382	71681	141	141	33	33	8.90	Transketolase OS=Lactococcus lactis subsp. lactis (strain IL1403) OX=272623 GN=tkt PE=3 SV=1
34	1	lactococcus	Q9CEH8	5229	106672	181	181	52	52	7.82	Isoleucine--tRNA ligase OS=Lactococcus lactis subsp. lactis (strain IL1403) OX=272623 GN=ileS PE=3 SV=1
35	1	lactococcus	Q9CHB6	5133	93733	160	160	41	41	8.51	Leucine--tRNA ligase OS=Lactococcus lactis subsp. lactis (strain IL1403) OX=272623 GN=leuS PE=3 SV=1
36	1	lactococcus	Q9CE35	5128	43110	156	156	30	30	65.21	Acetate kinase 1 OS=Lactococcus lactis subsp. lactis (strain IL1403) OX=272623 GN=ackA1 PE=3 SV=1
37	1	lactococcus	Q9CE12	4919	62780	161	161	44	44	23.97	Arginine--tRNA ligase OS=Lactococcus lactis subsp. lactis (strain IL1403) OX=272623 GN=argS PE=3 SV=1
38	1	lactococcus	Q9CF40	4824	49702	162	162	33	33	23.85	Adenylosuccinate lyase OS=Lactococcus lactis subsp. lactis (strain IL1403) OX=272623 GN=purB PE=3 SV=1
39	1	lactococcus	Q9CE93	4756	47480	148	148	29	29	14.53	Adenylosuccinate synthetase OS=Lactococcus lactis subsp. lactis (strain IL1403) OX=272623 GN=purA PE=3 SV=1
41	1	lactococcus	O32797	4203	89341	101	101	41	41	45158	Formate acetyltransferase OS=Lactococcus lactis subsp. lactis (strain IL1403) OX=272623 GN=pfl PE=3 SV=1
42	1	lactococcus	Q9CG41	3874	18076	114	114	26	26	486.61	50S ribosomal protein L10 OS=Lactococcus lactis subsp. lactis (strain IL1403) OX=272623 GN=rplJ PE=3 SV=1
43	1	lactococcus	Q9CG42	3851	12401	98	98	19	19	740.37	50S ribosomal protein L7/L12 OS=Lactococcus lactis subsp. lactis (strain IL1403) OX=272623 GN=rplL PE=3 SV=1
44	1	lactococcus	Q9CF22	3790	35326	118	118	28	28	49.31	Phosphate acetyltransferase OS=Lactococcus lactis subsp. lactis (strain IL1403) OX=272623 GN=pta PE=3 SV=1
45	1	lactococcus	Q9CH10	3752	25411	114	114	22	22	59.61	Purine nucleoside phosphorylase DeoD-type OS=Lactococcus lactis subsp. lactis (strain IL1403) OX=272623 GN=deoD PE=3 SV=1
46	1	lactococcus	Q9CEN7	3701	135106	125	125	45	45	45025	DNA-directed RNA polymerase subunit beta' OS=Lactococcus lactis subsp. lactis (strain IL1403) OX=272623 GN=rpoC PE=3 SV=2
47	1	lactococcus	Q9CDR5	3565	36647	100	100	24	24	26.65	Elongation factor Ts OS=Lactococcus lactis subsp. lactis (strain IL1403) OX=272623 GN=tsf PE=3 SV=1
48	1	lactococcus	P0C2U0	3391	39477	114	114	20	20	14.81	Ornithine carbamoyltransferase, catabolic OS=Lactococcus lactis subsp. lactis (strain IL1403) OX=272623 GN=arcB PE=3 SV=1
49	1	lactococcus	Q9CJ45	3105	29202	94	94	20	20	46.82	Transcriptional regulator OS=Lactococcus lactis subsp. lactis (strain IL1403) OX=272623 GN=codY PE=3 SV=1
50	1	lactococcus	Q9CIL2	3100	59707	88	88	31	31	45193	Oligopeptide ABC transporter substrate binding protein OS=Lactococcus lactis subsp. lactis (strain IL1403) OX=272623 GN=optA PE=3 SV=1
51	1	lactococcus	Q07744	3031	71493	86	86	32	32	5.60	Neutral endopeptidase OS=Lactococcus lactis subsp. lactis (strain IL1403) OX=272623 GN=pepO PE=1 SV=3
52	1	lactococcus	Q9CED6	2940	41036	100	100	30	30	22.77	DUF4097 domain-containing protein OS=Lactococcus lactis subsp. lactis (strain IL1403) OX=272623 GN=ythC PE=4 SV=1

53	1	lactococcus	Q9CJJ1	2842	42238	96	96	20	20	45111	Beta sliding clamp OS=Lactococcus lactis subsp. lactis (strain IL1403) OX=272623 GN=dnaN PE=3 SV=1
54	1	lactococcus	Q9CFM9	2792	36722	59	59	20	20	8.80	Basic membrane protein A OS=Lactococcus lactis subsp. lactis (strain IL1403) OX=272623 GN=bmpA PE=3 SV=1
55	1	lactococcus	Q9CFW8	2652	33125	78	78	20	20	17.39	Dihydroorotate dehydrogenase B (NAD(+)), catalytic subunit OS=Lactococcus lactis subsp. lactis (strain IL1403) OX=272623 GN=pyrDB PE=1 SV=1
56	1	lactococcus	Q9CH92	2619	47795	94	94	27	27	11.78	Glutathione reductase OS=Lactococcus lactis subsp. lactis (strain IL1403) OX=272623 GN=gshR PE=3 SV=1
57	1	lactococcus	Q9CHB9	2583	51036	63	63	26	26	45162	Signal recognition particle receptor FtsY OS=Lactococcus lactis subsp. lactis (strain IL1403) OX=272623 GN=ftsY PE=3 SV=1
58	1	lactococcus	Q9CG71	2506	20199	84	84	24	24	170.43	Uncharacterized protein OS=Lactococcus lactis subsp. lactis (strain IL1403) OX=272623 GN=ymgG PE=3 SV=1
58	2	lactococcus	Q9CEF2	1063	18468	35	35	16	16	44.98	Uncharacterized protein OS=Lactococcus lactis subsp. lactis (strain IL1403) OX=272623 GN=ytgH PE=3 SV=1
59	1	lactococcus	Q9CDT4	2434	69254	66	66	25	25	4.49	Proline--tRNA ligase OS=Lactococcus lactis subsp. lactis (strain IL1403) OX=272623 GN=proS PE=3 SV=1
60	1	lactococcus	Q9CHQ3	2272	22068	79	79	15	15	34.92	ATP-dependent Clp protease proteolytic subunit OS=Lactococcus lactis subsp. lactis (strain IL1403) OX=272623 GN=clpP PE=3 SV=1
61	1	lactococcus	Q9CGF1	2258	35952	63	63	22	22	17.43	GMP reductase OS=Lactococcus lactis subsp. lactis (strain IL1403) OX=272623 GN=guaC PE=3 SV=1
62	1	lactococcus	Q9CEI0	2238	43185	60	60	17	17	3.74	Elongation factor Tu OS=Lactococcus lactis subsp. lactis (strain IL1403) OX=272623 GN=tuf PE=3 SV=1
63	1	lactococcus	Q9CGT6	2233	65708	54	54	18	18	44995	Glutamine--fructose-6-phosphate aminotransferase [isomerizing] OS=Lactococcus lactis subsp. lactis (strain IL1403) OX=272623 GN=glmS PE=3 SV=2
64	1	lactococcus	Q9CHF6	2173	42631	60	60	12	12	2.97	3-oxoacyl-[acyl-carrier-protein] synthase 2 OS=Lactococcus lactis subsp. lactis (strain IL1403) OX=272623 GN=fabF PE=3 SV=1
65	1	lactococcus	Q9CI02	2082	26458	62	62	21	21	36.92	Enoyl-[acyl-carrier-protein] reductase [NADH] OS=Lactococcus lactis subsp. lactis (strain IL1403) OX=272623 GN=fabI PE=3 SV=1
66	1	lactococcus	Q7DAV2	2059	60825	51	51	16	16	2.73	Alpha-acetolactate synthase OS=Lactococcus lactis subsp. lactis (strain IL1403) OX=272623 GN=als PE=3 SV=1
67	1	lactococcus	Q9CF61	1962	32262	41	41	11	11	45001	4-hydroxy-tetrahydrodipicolinate synthase OS=Lactococcus lactis subsp. lactis (strain IL1403) OX=272623 GN=dapA PE=3 SV=1
68	1	lactococcus	Q9CHT4	1914	21374	50	50	13	13	21.38	Probable DNA-directed RNA polymerase subunit delta OS=Lactococcus lactis subsp. lactis (strain IL1403) OX=272623 GN=rpoE PE=3 SV=1
69	1	lactococcus	Q9CDR4	1898	28521	43	43	13	13	12.98	30S ribosomal protein S2 OS=Lactococcus lactis subsp. lactis (strain IL1403) OX=272623 GN=rpsB PE=3 SV=1
70	1	lactococcus	Q9CF32	1857	40154	47	47	12	12	4.32	Proline dipeptidase OS=Lactococcus lactis subsp. lactis (strain IL1403) OX=272623 GN=pepQ PE=3 SV=1
71	1	lactococcus	P0A4K2	1854	40912	63	63	18	18	5.33	Cystathionine beta-lyase OS=Lactococcus lactis subsp. lactis (strain IL1403) OX=272623 GN=metC PE=3 SV=1
72	1	lactococcus	Q9CH71	1774	83390	54	54	25	25	2.73	Penicillin-binding protein OS=Lactococcus lactis subsp. lactis (strain IL1403) OX=272623 GN=pbpX PE=3 SV=1
73	1	lactococcus	Q9CEN6	1771	133208	70	70	31	31	1.76	DNA-directed RNA polymerase subunit beta OS=Lactococcus lactis subsp. lactis (strain IL1403) OX=272623 GN=rpoB PE=3 SV=1
74	1	lactococcus	Q9CIY6	1723	52839	51	50	19	19	3.92	Inosine-5'-monophosphate dehydrogenase OS=Lactococcus lactis subsp. lactis (strain IL1403) OX=272623 GN=guaB PE=3 SV=1
75	1	lactococcus	Q9CGZ5	1716	46926	52	52	18	18	8.37	UPF0210 protein YjhD OS=Lactococcus lactis subsp. lactis (strain IL1403) OX=272623 GN=yjhD PE=3 SV=1
76	1	lactococcus	Q9CHE0	1705	75775	51	51	24	24	45018	Methionine--tRNA ligase OS=Lactococcus lactis subsp. lactis (strain IL1403) OX=272623 GN=metG PE=3 SV=1
77	1	lactococcus	Q9CF33	1691	36624	41	41	13	13	45070	Catabolite control protein A OS=Lactococcus lactis subsp. lactis (strain IL1403) OX=272623 GN=ccpA PE=1 SV=1
78	1	lactococcus	Q9CFV2	1603	118138	49	49	22	22	44954	Carbamoyl-phosphate synthase large chain OS=Lactococcus lactis subsp. lactis (strain IL1403) OX=272623 GN=carB PE=3 SV=1
79	1	lactococcus	Q9CHU7	1583	20647	52	52	17	17	29.70	Uncharacterized protein OS=Lactococcus lactis subsp. lactis (strain IL1403) OX=272623 GN=ygaJ PE=4 SV=1

80	1	lactococcus	Q9CFG0	1575	57196	46	46	21	21	4.44	Bifunctional purine biosynthesis protein PurH OS=Lactococcus lactis subsp. lactis (strain IL1403) OX=272623 GN=purH PE=3 SV=1
81	1	lactococcus	Q9CH05	1557	30636	54	54	19	19	19.18	Amino acid ABC transporter substrate binding protein OS=Lactococcus lactis subsp. lactis (strain IL1403) OX=272623 GN=yjgC PE=4 SV=1
82	1	lactococcus	Q9CGY9	1544	20568	40	40	9	9	12.91	Protein GrpE OS=Lactococcus lactis subsp. lactis (strain IL1403) OX=272623 GN=grpE PE=3 SV=1
83	1	lactococcus	Q9CHK2	1544	30998	42	42	13	13	5.63	1,4-dihydroxy-2-naphthoyl-CoA synthase OS=Lactococcus lactis subsp. lactis (strain IL1403) OX=272623 GN=menB PE=3 SV=1
84	1	lactococcus	Q9CJC4	1494	46241	35	35	10	10	1.48	O-acetylhomoserine sulfhydrylase OS=Lactococcus lactis subsp. lactis (strain IL1403) OX=272623 GN=cysD PE=3 SV=1
85	1	lactococcus	Q9CJD7	1449	56282	45	45	16	16	2.57	Dihydrolipoamide acetyltransferase component of pyruvate dehydrogenase complex OS=Lactococcus lactis subsp. lactis (strain IL1403) OX=272623 GN=pdhC PE=3 SV=1
86	1	lactococcus	Q9LA06	1401	41623	33	33	16	16	4.55	Serine protease Do-like HtrA OS=Lactococcus lactis subsp. lactis (strain IL1403) OX=272623 GN=htrA PE=1 SV=1
87	1	lactococcus	Q9CFX9	1341	41289	40	40	13	13	45000	N-acetylglucosamine-6-phosphate deacetylase OS=Lactococcus lactis subsp. lactis (strain IL1403) OX=272623 GN=nagA PE=3 SV=1
88	1	lactococcus	Q9CIS6	1326	23348	48	48	15	15	16.47	Uncharacterized protein OS=Lactococcus lactis subsp. lactis (strain IL1403) OX=272623 GN=ychG PE=4 SV=1
89	1	lactococcus	Q9CDP6	1321	100475	48	48	18	18	1.42	Valine--tRNA ligase OS=Lactococcus lactis subsp. lactis (strain IL1403) OX=272623 GN=valS PE=3 SV=1
90	1	lactococcus	Q9CDS3	1288	36724	50	50	19	19	8.80	Uncharacterized protein OS=Lactococcus lactis subsp. lactis (strain IL1403) OX=272623 GN=ywaI PE=4 SV=1
91	1	lactococcus	Q9CFC7	1238	36580	32	32	10	10	2.95	Oxidoreductase OS=Lactococcus lactis subsp. lactis (strain IL1403) OX=272623 GN=ypjF PE=4 SV=1
92	1	lactococcus	Q9CFW7	1228	29135	31	31	10	10	45006	Dihydroorotate dehydrogenase B (NAD(+)), electron transfer subunit OS=Lactococcus lactis subsp. lactis (strain IL1403) OX=272623 GN=pyrK PE=1 SV=1
93	1	lactococcus	Q9CDZ9	1210	31050	38	38	15	15	7.62	Amino acid ABC transporter substrate binding protein OS=Lactococcus lactis subsp. lactis (strain IL1403) OX=272623 GN=yvdF PE=4 SV=1
94	1	lactococcus	Q9CIF8	1177	34313	37	37	14	14	4.53	Mevalonate kinase OS=Lactococcus lactis subsp. lactis (strain IL1403) OX=272623 GN=yeaG PE=4 SV=1
95	1	lactococcus	Q9CJD6	1158	35188	32	32	10	10	2.71	PDH E1 component beta subunit OS=Lactococcus lactis subsp. lactis (strain IL1403) OX=272623 GN=pdhB PE=4 SV=1
96	1	lactococcus	Q9CE25	1156	34001	26	26	11	11	3.39	Glucokinase OS=Lactococcus lactis subsp. lactis (strain IL1403) OX=272623 GN=glk PE=3 SV=1
97	1	lactococcus	Q9CF28	1120	14970	37	37	13	13	46.37	Uncharacterized protein OS=Lactococcus lactis subsp. lactis (strain IL1403) OX=272623 GN=yraB PE=4 SV=1
98	1	lactococcus	Q9CIU0	1111	17686	36	36	11	11	9.47	S-ribosylhomocysteine lyase OS=Lactococcus lactis subsp. lactis (strain IL1403) OX=272623 GN=luxS PE=3 SV=1
99	1	lactococcus	Q9CGH2	1107	52866	31	31	15	15	2.57	Fumarate reductase flavoprotein subunit OS=Lactococcus lactis subsp. lactis (strain IL1403) OX=272623 GN=frdC PE=3 SV=1
100	1	lactococcus	Q9CF59	1095	39406	24	24	7	7	44937	Aspartate-semialdehyde dehydrogenase OS=Lactococcus lactis subsp. lactis (strain IL1403) OX=272623 GN=asd PE=3 SV=1
101	1	lactococcus	Q9CFF0	1084	54973	28	28	12	12	1.71	Amidophosphoribosyltransferase OS=Lactococcus lactis subsp. lactis (strain IL1403) OX=272623 GN=purF PE=3 SV=1
102	1	lactococcus	Q9CDY3	1027	34173	27	27	11	11	2.85	DNA-directed RNA polymerase subunit alpha OS=Lactococcus lactis subsp. lactis (strain IL1403) OX=272623 GN=rpoA PE=3 SV=1
103	1	lactococcus	Q9CDL9	1027	49800	24	24	11	11	1.75	Glutamine synthetase OS=Lactococcus lactis subsp. lactis (strain IL1403) OX=272623 GN=glnA PE=3 SV=1
104	1	lactococcus	Q9CIH5	988	47221	37	37	9	9	1.44	Tyrosine--tRNA ligase OS=Lactococcus lactis subsp. lactis (strain IL1403) OX=272623 GN=tyrS PE=3 SV=1
105	1	lactococcus	Q9CES3	981	69799	22	22	9	9	0.83	Uncharacterized protein OS=Lactococcus lactis subsp. lactis (strain IL1403) OX=272623 GN=yscA PE=4 SV=1
106	1	lactococcus	Q9CJ71	924	33774	26	26	13	13	5.44	R3H domain-containing protein OS=Lactococcus lactis subsp. lactis (strain IL1403) OX=272623 GN=ybdD PE=4 SV=1
107	1	lactococcus	Q9CJE0	903	43233	28	28	12	12	44978	Aminotransferase OS=Lactococcus lactis subsp. lactis (strain IL1403) OX=272623 GN=araT PE=3 SV=1

107	2	lactococcus	Q9CDS1	214	98671	4	4	4	4	0.19	DNA polymerase I OS=Lactococcus lactis subsp. lactis (strain IL1403) OX=272623 GN=polA PE=3 SV=1
108	1	lactococcus	Q9CH57	881	85096	17	17	11	11	0.72	Uncharacterized protein OS=Lactococcus lactis subsp. lactis (strain IL1403) OX=272623 GN=yjaE PE=4 SV=1
109	1	lactococcus	Q9CH94	874	40847	26	26	10	10	44967	Alanine racemase OS=Lactococcus lactis subsp. lactis (strain IL1403) OX=272623 GN=alr PE=3 SV=1
110	1	lactococcus	Q9CFA9	873	42733	25	25	8	8	1.67	Hydroxymethylglutaryl-CoA synthase OS=Lactococcus lactis subsp. lactis (strain IL1403) OX=272623 GN=hmcM PE=3 SV=1
111	1	lactococcus	Q9CFW5	854	43118	25	25	9	9	1.41	Band_7_1 domain-containing protein OS=Lactococcus lactis subsp. lactis (strain IL1403) OX=272623 GN=yniH PE=4 SV=1
112	1	lactococcus	Q9CEH7	852	34931	24	24	14	14	4.35	Uncharacterized protein OS=Lactococcus lactis subsp. lactis (strain IL1403) OX=272623 GN=ytdB PE=4 SV=1
113	1	lactococcus	Q9CH02	850	33965	26	26	10	10	2.88	Thioredoxin reductase OS=Lactococcus lactis subsp. lactis (strain IL1403) OX=272623 GN=trxB1 PE=3 SV=1
114	1	lactococcus	Q9CGY7	846	67403	26	26	12	12	44938	Myosin-crossreactive antigen OS=Lactococcus lactis subsp. lactis (strain IL1403) OX=272623 GN=mycA PE=4 SV=1
115	1	lactococcus	Q9CHT8	844	34106	22	22	7	7	1.67	Ribonuclease Z OS=Lactococcus lactis subsp. lactis (strain IL1403) OX=272623 GN=rnz PE=3 SV=1
116	1	lactococcus	Q9CEB6	840	35813	18	18	12	12	44992	3D domain-containing protein OS=Lactococcus lactis subsp. lactis (strain IL1403) OX=272623 GN=yuaE PE=4 SV=1
117	1	lactococcus	Q9CFI1	839	52952	18	18	11	11	1.40	Sugar ABC transporter substrate binding protein OS=Lactococcus lactis subsp. lactis (strain IL1403) OX=272623 GN=ypcG PE=4 SV=1
118	1	lactococcus	Q9CID5	834	87351	28	28	15	15	44943	Trehalose 6-phosphate phosphorylase OS=Lactococcus lactis subsp. lactis (strain IL1403) OX=272623 GN=trePP PE=1 SV=3
119	1	lactococcus	Q9CJD5	824	41299	28	28	12	12	2.39	Pyruvate dehydrogenase E1 component subunit alpha OS=Lactococcus lactis subsp. lactis (strain IL1403) OX=272623 GN=pdhA PE=4 SV=1
120	1	lactococcus	Q9CGM2	824	13660	25	25	4	4	3.52	Fe-S_biosyn domain-containing protein OS=Lactococcus lactis subsp. lactis (strain IL1403) OX=272623 GN=ykiE PE=4 SV=1
121	1	lactococcus	Q9CEB9	815	23527	28	28	6	6	2.46	Ribulose-phosphate 3-epimerase OS=Lactococcus lactis subsp. lactis (strain IL1403) OX=272623 GN=rpe PE=3 SV=1
122	1	lactococcus	Q9CFB1	808	44969	16	16	9	9	1.55	3-hydroxy-3-methylglutaryl coenzyme A reductase OS=Lactococcus lactis subsp. lactis (strain IL1403) OX=272623 GN=mvaA PE=3 SV=1
123	1	lactococcus	Q9CFC3	806	53793	23	23	12	12	1.55	Probable dipeptidase B OS=Lactococcus lactis subsp. lactis (strain IL1403) OX=272623 GN=pepDB PE=3 SV=1
124	1	lactococcus	Q9CI76	799	42237	21	21	6	6	1.44	Amino acids and amines aminotransferase OS=Lactococcus lactis subsp. lactis (strain IL1403) OX=272623 GN=yeiG PE=3 SV=1
125	1	lactococcus	Q9CG80	790	81197	21	21	15	15	44944	DNA topoisomerase 1 OS=Lactococcus lactis subsp. lactis (strain IL1403) OX=272623 GN=topA PE=3 SV=1
126	1	lactococcus	Q9CH36	777	72551	24	24	12	12	44927	DNA gyrase subunit B OS=Lactococcus lactis subsp. lactis (strain IL1403) OX=272623 GN=gyrB PE=3 SV=1
127	1	lactococcus	Q9CEP5	775	45333	32	32	10	10	44987	Cysteine desulfurase OS=Lactococcus lactis subsp. lactis (strain IL1403) OX=272623 GN=yseI PE=3 SV=1
128	1	lactococcus	Q9CH07	770	59543	23	23	10	10	44929	Formate--tetrahydrofolate ligase OS=Lactococcus lactis subsp. lactis (strain IL1403) OX=272623 GN=fhs PE=3 SV=1
129	1	lactococcus	Q9CEJ9	769	13657	27	27	6	6	45216	50S ribosomal protein L20 OS=Lactococcus lactis subsp. lactis (strain IL1403) OX=272623 GN=rplT PE=3 SV=1
130	1	lactococcus	Q9CEN1	764	53939	20	20	11	11	1.36	UDP-N-acetylmuramyl-tripeptide synthetase OS=Lactococcus lactis subsp. lactis (strain IL1403) OX=272623 GN=murE PE=3 SV=1
131	1	lactococcus	Q9CEG3	764	49975	20	20	8	8	0.96	Aminopeptidase C OS=Lactococcus lactis subsp. lactis (strain IL1403) OX=272623 GN=pepC PE=3 SV=3
132	1	lactococcus	Q9CE17	746	34097	17	16	7	7	1.36	Carbamate kinase OS=Lactococcus lactis subsp. lactis (strain IL1403) OX=272623 GN=arcC2 PE=3 SV=1
133	1	lactococcus	Q9CF20	746	36267	25	25	10	10	3.00	Oxidoreductase OS=Lactococcus lactis subsp. lactis (strain IL1403) OX=272623 GN=yrbA PE=4 SV=1
134	1	lactococcus	Q9CIJ2	726	29956	15	14	6	6	44957	Peptidyl-prolyl cis-trans isomerase OS=Lactococcus lactis subsp. lactis (strain IL1403) OX=272623 GN=ppiA PE=3 SV=1

135	1	lactococcus	Q9CHZ6	709	19453	20	20	5	5	2.61	Pantothenate metabolism flavoprotein OS=Lactococcus lactis subsp. lactis (strain IL1403) OX=272623 GN=dfpA PE=4 SV=1
136	1	lactococcus	Q9CGW8	690	37683	20	20	10	10	44962	Ribonucleoside-diphosphate reductase OS=Lactococcus lactis subsp. lactis (strain IL1403) OX=272623 GN=nrdF PE=3 SV=1
137	1	lactococcus	Q9CIV8	689	36265	18	18	4	4	0.59	PTS-dependent dihydroxyacetone kinase, dihydroxyacetone-binding subunit DhaK OS=Lactococcus lactis subsp. lactis (strain IL1403) OX=272623 GN=dhaK PE=1 SV=2
138	1	lactococcus	Q9CI05	681	28532	22	22	10	10	3.33	Glutamate or arginine ABC transporter substrate binding protein OS=Lactococcus lactis subsp. lactis (strain IL1403) OX=272623 GN=gltS PE=4 SV=1
139	1	lactococcus	Q9CIM6	675	34380	21	21	8	8	1.65	Ferrichrome ABC transporter substrate binding protein OS=Lactococcus lactis subsp. lactis (strain IL1403) OX=272623 GN=fhuD PE=4 SV=1
140	1	lactococcus	Q9CEV9	672	33857	18	18	9	9	2.45	Foldase protein PrsA OS=Lactococcus lactis subsp. lactis (strain IL1403) OX=272623 GN=prsA PE=1 SV=1
141	1	lactococcus	Q9CGH1	672	24473	17	17	8	8	3.65	Uncharacterized protein OS=Lactococcus lactis subsp. lactis (strain IL1403) OX=272623 GN=yldE PE=4 SV=1
142	1	lactococcus	Q9CGM8	662	22619	22	22	11	11	6.56	Orotate phosphoribosyltransferase OS=Lactococcus lactis subsp. lactis (strain IL1403) OX=272623 GN=pyrE PE=3 SV=1
143	1	lactococcus	Q9CHV0	648	34520	18	18	9	9	2.37	HPr kinase/phosphorylase OS=Lactococcus lactis subsp. lactis (strain IL1403) OX=272623 GN=hprK PE=3 SV=1
144	1	lactococcus	Q48662	634	59572	18	18	10	10	44943	Malolactic enzyme OS=Lactococcus lactis subsp. lactis (strain IL1403) OX=272623 GN=mleS PE=3 SV=2
145	1	lactococcus	Q9CEF7	629	27955	13	13	6	6	1.45	Pyrroline-5-carboxylate reductase OS=Lactococcus lactis subsp. lactis (strain IL1403) OX=272623 GN=proC PE=3 SV=1
146	1	lactococcus	Q9CJD1	623	38027	19	19	9	9	2.75	Tryptophan--tRNA ligase OS=Lactococcus lactis subsp. lactis (strain IL1403) OX=272623 GN=trpS PE=3 SV=1
147	1	lactococcus	Q9CHG0	604	34868	16	16	8	8	1.62	3-oxoacyl-[acyl-carrier-protein] synthase 3 OS=Lactococcus lactis subsp. lactis (strain IL1403) OX=272623 GN=fabH PE=3 SV=1
148	1	lactococcus	Q9CG22	604	36969	15	15	8	8	1.48	Branched-chain-amino-acid aminotransferase OS=Lactococcus lactis subsp. lactis (strain IL1403) OX=272623 GN=bcaT PE=3 SV=1
149	1	lactococcus	Q9CJ54	601	43636	14	14	5	5	44942	Queuine tRNA-ribosyltransferase OS=Lactococcus lactis subsp. lactis (strain IL1403) OX=272623 GN=tgt PE=3 SV=1
150	1	lactococcus	Q9CIN8	596	31321	18	18	9	9	2.33	Lipoprotein OS=Lactococcus lactis subsp. lactis (strain IL1403) OX=272623 GN=plpA PE=3 SV=1
150	2	lactococcus	Q9CIN7	281	31145	10	10	8	8	1.93	Lipoprotein OS=Lactococcus lactis subsp. lactis (strain IL1403) OX=272623 GN=plpB PE=3 SV=1
151	1	lactococcus	Q9CEK0	579	66050	19	19	7	7	0.56	Oligopeptide-binding protein OppA OS=Lactococcus lactis subsp. lactis (strain IL1403) OX=272623 GN=oppA PE=3 SV=1
152	1	lactococcus	Q9CHK0	563	62531	16	16	10	10	44936	2-succinyl-5-enolpyruvyl-6-hydroxy-3-cyclohexene-1-carboxylate synthase OS=Lactococcus lactis subsp. lactis (strain IL1403) OX=272623 GN=menD PE=3 SV=1
153	1	lactococcus	Q9CDZ8	560	41638	18	18	7	7	44929	Pyridine nucleotide-disulfide oxidoreductase OS=Lactococcus lactis subsp. lactis (strain IL1403) OX=272623 GN=yvdG PE=4 SV=1
155	1	lactococcus	Q9CEB5	550	87280	19	19	11	11	0.88	Phenylalanine--tRNA ligase beta subunit OS=Lactococcus lactis subsp. lactis (strain IL1403) OX=272623 GN=pheT PE=3 SV=1
156	1	lactococcus	Q9CE66	547	36299	20	20	11	11	3.00	UDP-glucose 4-epimerase OS=Lactococcus lactis subsp. lactis (strain IL1403) OX=272623 GN=galE PE=3 SV=1
157	1	lactococcus	Q9CHZ8	537	28260	10	10	4	4	44935	Phosphomannomutase OS=Lactococcus lactis subsp. lactis (strain IL1403) OX=272623 GN=yfgH PE=3 SV=1
158	1	lactococcus	Q9CFH4	532	93304	18	18	12	12	0.72	Probable phosphoketolase OS=Lactococcus lactis subsp. lactis (strain IL1403) OX=272623 GN=LL1502 PE=3 SV=1
159	1	lactococcus	Q9CFX6	530	37121	16	16	9	9	44967	Glycerol-3-phosphate dehydrogenase [NAD(P)+] OS=Lactococcus lactis subsp. lactis (strain IL1403) OX=272623 GN=gpsA PE=3 SV=1
160	1	lactococcus	Q9CE63	528	56658	17	17	8	8	0.81	Galactose-1-phosphate uridylyltransferase OS=Lactococcus lactis subsp. lactis (strain IL1403) OX=272623 GN=galT PE=3 SV=1
161	1	lactococcus	Q9CDQ6	523	19116	10	10	4	4	1.39	Phosphopantetheine adenylyltransferase OS=Lactococcus lactis subsp. lactis (strain IL1403) OX=272623 GN=coaD PE=3 SV=1
162	1	lactococcus	Q9CEN0	523	36123	18	18	8	8	1.53	Alcohol dehydrogenase OS=Lactococcus lactis subsp. lactis (strain IL1403) OX=272623 GN=adhA PE=3 SV=1

163	1	lactococcus	Q9CHF5	516	16362	12	12	4	4	2.55	Biotin carboxyl carrier protein of acetyl-CoA carboxylase OS=Lactococcus lactis subsp. lactis (strain IL1403) OX=272623 GN=accB PE=4 SV=1
164	1	lactococcus	Q9CID4	516	30997	12	12	5	5	0.97	Sugar hydrolase OS=Lactococcus lactis subsp. lactis (strain IL1403) OX=272623 GN=yeeB PE=1 SV=1
165	1	lactococcus	Q9CIL8	514	55338	18	18	10	10	44957	Alkyl hydroperoxide reductase OS=Lactococcus lactis subsp. lactis (strain IL1403) OX=272623 GN=ahpF PE=3 SV=1
166	1	lactococcus	Q9CHJ5	510	35035	13	13	7	7	44957	L-asparaginase OS=Lactococcus lactis subsp. lactis (strain IL1403) OX=272623 GN=ansB PE=3 SV=1
167	1	lactococcus	Q9CI23	498	71891	12	12	8	8	0.69	DD-transpeptidase OS=Lactococcus lactis subsp. lactis (strain IL1403) OX=272623 GN=ponA PE=4 SV=1
168	1	lactococcus	Q9CFX8	497	44283	17	17	10	10	1.58	Uncharacterized protein OS=Lactococcus lactis subsp. lactis (strain IL1403) OX=272623 GN=ynhC PE=3 SV=1
169	1	lactococcus	Q9CIL7	497	77851	8	8	7	7	0.46	Penicillin-binding protein 2B OS=Lactococcus lactis subsp. lactis (strain IL1403) OX=272623 GN=pbp2B PE=3 SV=1
170	1	lactococcus	Q9CE46	493	14696	13	13	4	4	44994	50S ribosomal protein L11 OS=Lactococcus lactis subsp. lactis (strain IL1403) OX=272623 GN=rplK PE=3 SV=1
171	1	lactococcus	Q9CE47	492	24035	15	15	8	8	3.75	50S ribosomal protein L1 OS=Lactococcus lactis subsp. lactis (strain IL1403) OX=272623 GN=rplA PE=3 SV=1
172	1	lactococcus	Q9CJ31	491	54686	20	20	8	8	0.85	Beta-glucosidase A OS=Lactococcus lactis subsp. lactis (strain IL1403) OX=272623 GN=bglS PE=3 SV=1
173	1	lactococcus	Q9CDH3	484	19496	12	12	4	4	1.34	DUF536 domain-containing protein OS=Lactococcus lactis subsp. lactis (strain IL1403) OX=272623 GN=yxdB PE=4 SV=1
174	1	lactococcus	Q9CGB8	482	58771	13	13	11	11	44946	Uncharacterized protein OS=Lactococcus lactis subsp. lactis (strain IL1403) OX=272623 GN=yljF PE=4 SV=1
175	1	lactococcus	Q9CFC0	467	28473	14	14	7	7	44980	4-hydroxy-tetrahydrodipicolinate reductase OS=Lactococcus lactis subsp. lactis (strain IL1403) OX=272623 GN=dapB PE=3 SV=1
176	1	lactococcus	Q9CG20	466	54168	20	20	9	9	44943	Glutamate decarboxylase OS=Lactococcus lactis subsp. lactis (strain IL1403) OX=272623 GN=gadB PE=1 SV=1
177	1	lactococcus	Q9CJC5	464	61435	14	14	8	8	0.85	ABC transporter ATP binding protein OS=Lactococcus lactis subsp. lactis (strain IL1403) OX=272623 GN=yahG PE=4 SV=1
178	1	lactococcus	Q9CGE4	458	13104	19	19	6	6	45139	7,8-dihydroneopterin aldolase OS=Lactococcus lactis subsp. lactis (strain IL1403) OX=272623 GN=folB PE=3 SV=1
179	1	lactococcus	Q9CHE1	454	38145	12	12	8	8	1.41	Exodeoxyribonuclease III OS=Lactococcus lactis subsp. lactis (strain IL1403) OX=272623 GN=exoA PE=3 SV=1
180	1	lactococcus	Q9CIH3	451	38339	16	16	8	8	1.40	Glutamyl aminopeptidase OS=Lactococcus lactis subsp. lactis (strain IL1403) OX=272623 GN=pepA PE=3 SV=1
181	1	lactococcus	Q9CDH9	443	27487	10	10	6	6	1.89	Uncharacterized protein OS=Lactococcus lactis subsp. lactis (strain IL1403) OX=272623 GN=yxcD PE=4 SV=1
182	1	lactococcus	Q9CHF9	441	8396	15	15	4	4	5.82	Acyl carrier protein OS=Lactococcus lactis subsp. lactis (strain IL1403) OX=272623 GN=acpP PE=3 SV=1
183	1	lactococcus	Q9CG26	440	47434	20	20	8	8	44948	Diaminopimelate decarboxylase OS=Lactococcus lactis subsp. lactis (strain IL1403) OX=272623 GN=lysA PE=3 SV=1
184	1	lactococcus	Q9CFB0	433	39827	8	8	5	5	0.69	Acetyl coenzyme A acetyltransferase OS=Lactococcus lactis subsp. lactis (strain IL1403) OX=272623 GN=thiL PE=3 SV=1
185	1	lactococcus	P50918	432	27027	8	8	2	2	0.59	Triosephosphate isomerase OS=Lactococcus lactis subsp. lactis (strain IL1403) OX=272623 GN=tpiA PE=1 SV=3
186	1	lactococcus	Q9CEE4	424	13426	17	17	5	5	45075	DNA-directed RNA polymerase subunit omega OS=Lactococcus lactis subsp. lactis (strain IL1403) OX=272623 GN=rpoZ PE=3 SV=1
187	1	lactococcus	Q9CHW7	422	44762	17	17	12	12	2.39	Serine hydroxymethyltransferase OS=Lactococcus lactis subsp. lactis (strain IL1403) OX=272623 GN=glyA PE=3 SV=1
188	1	lactococcus	Q01997	413	27714	16	16	5	5	1.47	Tryptophan synthase alpha chain OS=Lactococcus lactis subsp. lactis (strain IL1403) OX=272623 GN=trpA PE=3 SV=1
189	1	lactococcus	Q9CE86	409	31883	9	9	4	4	0.93	33 kDa chaperonin OS=Lactococcus lactis subsp. lactis (strain IL1403) OX=272623 GN=hslO PE=3 SV=1
190	1	lactococcus	Q9CHG7	407	42878	18	18	12	12	44981	Transcription termination/antitermination protein NusA OS=Lactococcus lactis subsp. lactis (strain IL1403) OX=272623 GN=nusA PE=3 SV=1

191	1	lactococcus	Q9CEE0	403	43037	11	11	6	6	0.80	S-adenosylmethionine synthase OS=Lactococcus lactis subsp. lactis (strain IL1403) OX=272623 GN=metK PE=3 SV=1
192	1	lactococcus	Q9CFF4	400	36254	11	11	4	4	0.59	Phosphoribosylformylglycinamidine cyclo-ligase OS=Lactococcus lactis subsp. lactis (strain IL1403) OX=272623 GN=purM PE=3 SV=1
193	1	lactococcus	Q9CE96	396	21380	14	14	8	8	3.73	Uncharacterized protein OS=Lactococcus lactis subsp. lactis (strain IL1403) OX=272623 GN=yucF PE=4 SV=1
194	1	lactococcus	Q9CE60	394	44126	13	13	7	7	1.35	Transcriptional regulator OS=Lactococcus lactis subsp. lactis (strain IL1403) OX=272623 GN=nadR PE=4 SV=1
195	1	lactococcus	Q9CHT3	392	61175	17	17	9	9	0.86	Endolytic murein transglycosylase OS=Lactococcus lactis subsp. lactis (strain IL1403) OX=272623 GN=ygcC PE=3 SV=1
196	1	lactococcus	Q9CIM5	390	34691	9	9	5	5	0.83	Fhu operon transcriptional regulator OS=Lactococcus lactis subsp. lactis (strain IL1403) OX=272623 GN=fhuR PE=3 SV=1
197	1	lactococcus	Q9CFZ5	389	35569	11	11	6	6	44929	Metal ABC transporter substrate-binding lipoprotein OS=Lactococcus lactis subsp. lactis (strain IL1403) OX=272623 GN=mtsA PE=3 SV=1
198	1	lactococcus	Q9CJ25	381	36391	9	9	5	5	0.78	Prenyl transferase OS=Lactococcus lactis subsp. lactis (strain IL1403) OX=272623 GN=preA PE=3 SV=1
199	1	lactococcus	Q9CJ97	377	34778	9	9	4	4	0.83	Ribosomal protein L11 methyltransferase OS=Lactococcus lactis subsp. lactis (strain IL1403) OX=272623 GN=prmA PE=3 SV=1
200	1	lactococcus	Q04506	374	44052	13	13	9	9	1.59	RNA polymerase sigma factor SigA OS=Lactococcus lactis subsp. lactis (strain IL1403) OX=272623 GN=sigA PE=3 SV=2
201	1	lactococcus	Q9CGM3	368	28542	19	19	9	9	3.33	GTP cyclohydrolase 1 type 2 homolog OS=Lactococcus lactis subsp. lactis (strain IL1403) OX=272623 GN=ykiD PE=3 SV=1
202	1	lactococcus	Q9CHF1	365	28529	8	8	4	4	0.80	Acetyl-CoA carboxytransferase OS=Lactococcus lactis subsp. lactis (strain IL1403) OX=272623 GN=accA PE=4 SV=1
203	1	lactococcus	Q9CIP3	362	8399	7	7	1	1	0.62	PC4 domain-containing protein OS=Lactococcus lactis subsp. lactis (strain IL1403) OX=272623 GN=ydbC PE=1 SV=1
204	1	lactococcus	Q9CED2	360	74241	15	15	7	7	0.57	Threonine--tRNA ligase OS=Lactococcus lactis subsp. lactis (strain IL1403) OX=272623 GN=thrS PE=3 SV=1
205	1	lactococcus	Q9CDR3	348	98298	14	14	10	10	0.60	Aldehyde-alcohol dehydrogenase OS=Lactococcus lactis subsp. lactis (strain IL1403) OX=272623 GN=adhE PE=3 SV=1
206	1	lactococcus	Q9CIS4	343	43165	10	10	5	5	0.63	N-acetyldiaminopimelate deacetylase OS=Lactococcus lactis subsp. lactis (strain IL1403) OX=272623 GN=yciA PE=3 SV=1
207	1	lactococcus	Q9CDK4	340	31394	10	10	8	8	1.90	3-hydroxyisobutyrate dehydrogenase OS=Lactococcus lactis subsp. lactis (strain IL1403) OX=272623 GN=ywjF PE=4 SV=1
208	1	lactococcus	Q9CIH4	339	86912	11	11	9	9	0.55	DD-transpeptidase OS=Lactococcus lactis subsp. lactis (strain IL1403) OX=272623 GN=pbp1B PE=4 SV=1
209	1	lactococcus	Q9CDV3	338	81924	11	11	6	6	0.43	DD-transpeptidase OS=Lactococcus lactis subsp. lactis (strain IL1403) OX=272623 GN=pbp2A PE=4 SV=1
210	1	lactococcus	Q9CH99	337	25844	10	9	4	3	0.91	Uridine phosphorylase OS=Lactococcus lactis subsp. lactis (strain IL1403) OX=272623 GN=udp PE=3 SV=1
211	1	lactococcus	Q9CGJ4	333	30263	7	7	4	4	0.74	NH(3)-dependent NAD(+) synthetase OS=Lactococcus lactis subsp. lactis (strain IL1403) OX=272623 GN=nadE PE=3 SV=1
212	1	lactococcus	Q9CHN7	313	39567	8	8	7	7	44936	Aminopeptidase P OS=Lactococcus lactis subsp. lactis (strain IL1403) OX=272623 GN=pepP PE=3 SV=1
213	1	lactococcus	Q9CIE3	306	37340	12	12	6	6	0.96	Transcriptional regulator OS=Lactococcus lactis subsp. lactis (strain IL1403) OX=272623 GN=yecE PE=3 SV=1
214	1	lactococcus	Q9CHE9	299	8544	9	9	3	3	45000	UPF0337 protein YhjA OS=Lactococcus lactis subsp. lactis (strain IL1403) OX=272623 GN=yhjA PE=3 SV=1
215	1	lactococcus	Q9CEM5	295	34048	8	8	4	4	0.64	Probable manganese-dependent inorganic pyrophosphatase OS=Lactococcus lactis subsp. lactis (strain IL1403) OX=272623 GN=ppaC PE=3 SV=1
216	1	lactococcus	Q9CII7	294	56579	10	10	6	6	0.68	Lysine--tRNA ligase OS=Lactococcus lactis subsp. lactis (strain IL1403) OX=272623 GN=lysS PE=3 SV=1
217	1	lactococcus	Q9CGE2	292	40505	7	7	7	7	44933	Dihydropteroate pyrophosphorylase OS=Lactococcus lactis subsp. lactis (strain IL1403) OX=272623 GN=folP PE=3 SV=1

218	1	lactococcus	Q9CGJ6	270	56404	8	8	8	8	0.82	Nicotinate phosphoribosyltransferase OS=Lactococcus lactis subsp. lactis (strain IL1403) OX=272623 GN=ylaF PE=3 SV=1
219	1	lactococcus	Q9CH96	269	52003	8	8	5	5	0.63	Dipeptidase OS=Lactococcus lactis subsp. lactis (strain IL1403) OX=272623 GN=pepV PE=4 SV=1
220	1	lactococcus	Q9CIQ7	268	37403	7	7	5	5	0.75	Uncharacterized protein OS=Lactococcus lactis subsp. lactis (strain IL1403) OX=272623 GN=ycjI PE=4 SV=1
221	1	lactococcus	Q9CEK7	257	43200	8	8	7	7	44944	Aminotransferase OS=Lactococcus lactis subsp. lactis (strain IL1403) OX=272623 GN=aspB PE=3 SV=1
222	1	lactococcus	Q9CED5	253	49545	5	5	4	4	0.40	N-acetylmuramidase OS=Lactococcus lactis subsp. lactis (strain IL1403) OX=272623 GN=acmB PE=3 SV=1
223	1	lactococcus	Q9CEX6	250	33671	10	10	6	6	44937	Mannose-specific PTS system component IID OS=Lactococcus lactis subsp. lactis (strain IL1403) OX=272623 GN=ptnD PE=4 SV=1
224	1	lactococcus	Q9CE18	249	42909	14	14	9	9	1.41	Aminotransferase OS=Lactococcus lactis subsp. lactis (strain IL1403) OX=272623 GN=arcT PE=3 SV=1
225	1	lactococcus	Q9CF10	247	55013	10	10	5	5	0.47	Phospho-beta-glucosidase OS=Lactococcus lactis subsp. lactis (strain IL1403) OX=272623 GN=yrcA PE=3 SV=1
225	2	lactococcus	Q9CIE6	174	55206	5	5	3	3	0.26	Phospho-beta-glucosidase OS=Lactococcus lactis subsp. lactis (strain IL1403) OX=272623 GN=bglA PE=3 SV=1
226	1	lactococcus	Q9CEI5	239	39952	7	7	5	5	0.69	Uncharacterized protein OS=Lactococcus lactis subsp. lactis (strain IL1403) OX=272623 GN=ytcC PE=4 SV=1
227	1	lactococcus	Q7DAU1	232	29668	9	9	5	5	44929	Lipase OS=Lactococcus lactis subsp. lactis (strain IL1403) OX=272623 GN=yscE PE=4 SV=1
228	1	lactococcus	Q9CG49	230	56228	4	4	3	3	0.25	D-alanine--D-alanyl carrier protein ligase OS=Lactococcus lactis subsp. lactis (strain IL1403) OX=272623 GN=dltA PE=3 SV=1
229	1	lactococcus	Q9CJ08	228	39483	7	7	5	5	0.89	dTDP-glucose 4,6-dehydratase OS=Lactococcus lactis subsp. lactis (strain IL1403) OX=272623 GN=rmlB PE=3 SV=1
230	1	lactococcus	Q9CHA6	227	17399	6	6	2	2	0.61	Transcriptional regulator OS=Lactococcus lactis subsp. lactis (strain IL1403) OX=272623 GN=copR PE=1 SV=1
232	1	lactococcus	Q9CEP4	213	45436	4	4	2	2	0.20	Uncharacterized protein OS=Lactococcus lactis subsp. lactis (strain IL1403) OX=272623 GN=ysfA PE=3 SV=1
233	1	lactococcus	Q01998	213	43856	6	6	5	5	0.61	Tryptophan synthase beta chain OS=Lactococcus lactis subsp. lactis (strain IL1403) OX=272623 GN=trpB PE=3 SV=1
234	1	lactococcus	Q02146	211	75365	5	5	5	5	0.32	Ribonuclease R 2 OS=Lactococcus lactis subsp. lactis (strain IL1403) OX=272623 GN=rnr2 PE=3 SV=3
235	1	lactococcus	Q9CDI9	210	31429	5	5	3	3	0.49	Cell shape-determining protein MreC OS=Lactococcus lactis subsp. lactis (strain IL1403) OX=272623 GN=mreC PE=3 SV=1
236	1	lactococcus	Q9CHF7	204	25582	4	4	2	2	0.39	3-oxoacyl-[acyl-carrier-protein] reductase OS=Lactococcus lactis subsp. lactis (strain IL1403) OX=272623 GN=fabG1 PE=3 SV=1
237	1	lactococcus	Q9CDF8	202	47102	4	4	3	3	0.31	Serine-type D-Ala-D-Ala carboxypeptidase OS=Lactococcus lactis subsp. lactis (strain IL1403) OX=272623 GN=dacA PE=3 SV=1
238	1	lactococcus	Q9CFE8	200	80059	5	5	4	4	0.23	Phosphoribosylformylglycinamidine synthase subunit PurL OS=Lactococcus lactis subsp. lactis (strain IL1403) OX=272623 GN=purL PE=3 SV=1
239	1	lactococcus	Q9CF73	200	45490	8	8	4	4	0.59	Gamma-glutamyl phosphate reductase OS=Lactococcus lactis subsp. lactis (strain IL1403) OX=272623 GN=proA PE=3 SV=1
240	1	lactococcus	Q9CH79	196	16861	4	4	2	2	0.63	Arginine repressor OS=Lactococcus lactis subsp. lactis (strain IL1403) OX=272623 GN=ahrC PE=3 SV=1
242	1	lactococcus	Q9CIG1	192	12717	3	3	1	1	0.38	Initiation-control protein YabA OS=Lactococcus lactis subsp. lactis (strain IL1403) OX=272623 GN=yeaD PE=3 SV=1
243	1	lactococcus	Q9CDH8	188	49938	10	10	7	7	0.80	tRNA modification GTPase MnmE OS=Lactococcus lactis subsp. lactis (strain IL1403) OX=272623 GN=mnmE PE=3 SV=1
244	1	lactococcus	Q9CG27	183	8973	6	6	1	1	0.57	UPF0154 protein YnbE OS=Lactococcus lactis subsp. lactis (strain IL1403) OX=272623 GN=ynbE PE=3 SV=1
245	1	lactococcus	Q9CF31	182	72305	5	5	5	5	0.34	Mid-cell-anchored protein Z OS=Lactococcus lactis subsp. lactis (strain IL1403) OX=272623 GN=yqjD PE=3 SV=1
246	1	lactococcus	Q9CF80	178	39817	6	6	3	3	0.52	Carbamoyl-phosphate synthase small chain OS=Lactococcus lactis subsp. lactis (strain IL1403) OX=272623 GN=carA PE=3 SV=1

247	1	lactococcus	Q9CF65	177	56808	4	4	3	3	0.25	Signal recognition particle protein OS=Lactococcus lactis subsp. lactis (strain IL1403) OX=272623 GN=ffh PE=3 SV=1
248	1	lactococcus	Q9CEU2	176	31039	9	9	6	6	44950	Prephenate dehydratase OS=Lactococcus lactis subsp. lactis (strain IL1403) OX=272623 GN=pheA PE=4 SV=1
249	1	lactococcus	Q9CDM9	172	18339	2	2	2	2	0.57	Single-stranded DNA-binding protein 2 OS=Lactococcus lactis subsp. lactis (strain IL1403) OX=272623 GN=ssb2 PE=3 SV=1
251	1	lactococcus	Q9CI26	165	32338	5	5	4	4	0.68	Cysteine synthase OS=Lactococcus lactis subsp. lactis (strain IL1403) OX=272623 GN=cysM PE=3 SV=1
252	1	lactococcus	Q9CGK0	164	75670	3	3	3	3	0.18	Glycine--tRNA ligase beta subunit OS=Lactococcus lactis subsp. lactis (strain IL1403) OX=272623 GN=glyS PE=3 SV=1
253	1	lactococcus	Q9CHQ7	163	126649	4	4	4	4	0.14	Pyruvate carboxylase OS=Lactococcus lactis subsp. lactis (strain IL1403) OX=272623 GN=pycA PE=4 SV=1
254	1	lactococcus	Q9CIU1	163	40276	4	4	4	4	0.52	Oxidoreductase OS=Lactococcus lactis subsp. lactis (strain IL1403) OX=272623 GN=ycgD PE=4 SV=1
255	1	lactococcus	Q9CIV6	160	13386	2	2	1	1	0.36	PTS-dependent dihydroxyacetone kinase, phosphotransferase subunit DhaM OS=Lactococcus lactis subsp. lactis (strain IL1403) OX=272623 GN=dhaM PE=1 SV=1
256	1	lactococcus	Q9CEI2	159	41108	5	5	4	4	0.51	Pyridoxal-phosphate dependent aminotransferase NifS OS=Lactococcus lactis subsp. lactis (strain IL1403) OX=272623 GN=nifS PE=4 SV=1
256	2	lactococcus	Q9CER8	47	54337	2	2	2	2	0.17	ATP synthase subunit alpha OS=Lactococcus lactis subsp. lactis (strain IL1403) OX=272623 GN=atpA PE=3 SV=1
257	1	lactococcus	Q9CEF6	159	10337	7	7	3	3	44985	30S ribosomal protein S15 OS=Lactococcus lactis subsp. lactis (strain IL1403) OX=272623 GN=rpsO PE=3 SV=1
258	1	lactococcus	Q9CGZ2	158	27461	3	3	2	2	0.36	D-alanyl-D-alanine carboxypeptidase OS=Lactococcus lactis subsp. lactis (strain IL1403) OX=272623 GN=dacB PE=4 SV=1
259	1	lactococcus	Q9CEY2	155	52517	4	4	2	2	0.17	Transcription regulator OS=Lactococcus lactis subsp. lactis (strain IL1403) OX=272623 GN=yrfE PE=3 SV=1
260	1	lactococcus	Q9CI66	154	43621	3	2	3	2	0.21	Glyco_hydro_3 domain-containing protein OS=Lactococcus lactis subsp. lactis (strain IL1403) OX=272623 GN=yejJ PE=3 SV=1
261	1	lactococcus	Q9CH83	151	8959	4	4	3	3	2.88	Exodeoxyribonuclease 7 small subunit OS=Lactococcus lactis subsp. lactis (strain IL1403) OX=272623 GN=xseB PE=3 SV=1
262	1	lactococcus	Q9CIR6	150	61569	6	6	4	4	0.31	Ribonuclease J OS=Lactococcus lactis subsp. lactis (strain IL1403) OX=272623 GN=yciH PE=3 SV=1
263	1	lactococcus	Q9CDX7	150	14676	3	3	2	2	0.76	30S ribosomal protein S8 OS=Lactococcus lactis subsp. lactis (strain IL1403) OX=272623 GN=rpsH PE=3 SV=1
264	1	lactococcus	Q9CEZ9	150	68130	3	3	2	2	0.13	Neopullulanase OS=Lactococcus lactis subsp. lactis (strain IL1403) OX=272623 GN=dexC PE=4 SV=1
265	1	lactococcus	Q9CHS8	149	21270	3	3	3	3	0.80	Ribosome hibernation promoting factor OS=Lactococcus lactis subsp. lactis (strain IL1403) OX=272623 GN=ygdA PE=3 SV=1
266	1	lactococcus	Q9CDN9	148	100350	3	3	3	3	0.13	GRAM_POS_ANCHORING domain-containing protein OS=Lactococcus lactis subsp. lactis (strain IL1403) OX=272623 GN=ywfG PE=4 SV=1
267	1	lactococcus	Q9CJ47	147	58336	5	5	3	3	0.24	Uncharacterized protein OS=Lactococcus lactis subsp. lactis (strain IL1403) OX=272623 GN=ybgB PE=4 SV=1
268	1	lactococcus	Q9CH60	147	15734	3	3	2	2	0.69	Universal stress protein OS=Lactococcus lactis subsp. lactis (strain IL1403) OX=272623 GN=yjaB PE=3 SV=1
269	1	lactococcus	Q9CDG2	144	23535	4	4	2	2	0.70	Signal peptidase I OS=Lactococcus lactis subsp. lactis (strain IL1403) OX=272623 GN=sipL PE=3 SV=1
270	1	lactococcus	Q9CGL2	144	35935	3	3	2	2	0.26	Phosphate starvation inducible protein OS=Lactococcus lactis subsp. lactis (strain IL1403) OX=272623 GN=phoL PE=4 SV=1
271	1	lactococcus	Q9CDY0	142	17585	5	5	4	4	1.57	30S ribosomal protein S5 OS=Lactococcus lactis subsp. lactis (strain IL1403) OX=272623 GN=rpsE PE=3 SV=1
272	1	lactococcus	Q9CIL9	141	20728	3	3	2	2	0.49	Alkyl hydroperoxide reductase C OS=Lactococcus lactis subsp. lactis (strain IL1403) OX=272623 GN=ahpC PE=3 SV=1
273	1	lactococcus	Q9CGI4	137	39698	5	5	4	4	0.53	FAD:protein FMN transferase OS=Lactococcus lactis subsp. lactis (strain IL1403) OX=272623 GN=apbE PE=3 SV=1
274	1	lactococcus	Q9CG73	136	35124	2	2	2	2	0.27	N(5)-(carboxyethyl)ornithine synthase OS=Lactococcus lactis subsp. lactis (strain IL1403) OX=272623 GN=ceo PE=3 SV=1

275	1	lactococcus	Q9CDW5	136	29642	2	2	2	2	0.33	50S ribosomal protein L2 OS=Lactococcus lactis subsp. lactis (strain IL1403) OX=272623 GN=rplB PE=3 SV=1
276	1	lactococcus	Q9CGN6	135	24359	6	6	5	5	1.36	Redox-sensing transcriptional repressor Rex OS=Lactococcus lactis subsp. lactis (strain IL1403) OX=272623 GN=rex PE=3 SV=2
277	1	lactococcus	Q9CFJ6	130	28403	3	3	1	1	0.34	Oxidoreductase OS=Lactococcus lactis subsp. lactis (strain IL1403) OX=272623 GN=ypaI PE=3 SV=1
278	1	lactococcus	Q9CEI4	128	35348	3	3	3	3	0.43	Putative ribose-phosphate pyrophosphokinase 2 OS=Lactococcus lactis subsp. lactis (strain IL1403) OX=272623 GN=prs2 PE=3 SV=1
279	1	lactococcus	Q9CG46	127	27417	3	3	3	3	0.58	Hydroxyethylthiazole kinase OS=Lactococcus lactis subsp. lactis (strain IL1403) OX=272623 GN=thiM PE=3 SV=1
280	1	lactococcus	Q9CGY3	127	66301	3	3	2	2	0.14	Fructose-specific PTS system enzyme IIBC component OS=Lactococcus lactis subsp. lactis (strain IL1403) OX=272623 GN=fruA PE=4 SV=1
281	1	lactococcus	Q9CJ95	126	35388	4	4	3	3	0.43	NodB homology domain-containing protein OS=Lactococcus lactis subsp. lactis (strain IL1403) OX=272623 GN=ybaG PE=4 SV=1
282	1	lactococcus	Q9CE80	125	66549	4	4	3	3	0.29	Aspartate--tRNA ligase OS=Lactococcus lactis subsp. lactis (strain IL1403) OX=272623 GN=aspS PE=3 SV=1
283	1	lactococcus	Q9CH34	123	22314	3	3	1	1	0.21	DUF4767 domain-containing protein OS=Lactococcus lactis subsp. lactis (strain IL1403) OX=272623 GN=yjdB PE=4 SV=1
284	1	lactococcus	Q9CIC6	123	31554	2	2	1	1	0.14	Prophage pi1 protein 03, transcriptional regulator OS=Lactococcus lactis subsp. lactis (strain IL1403) OX=272623 GN=pi103 PE=4 SV=1
285	1	lactococcus	Q9CHB8	122	35615	4	4	4	4	0.60	Ribose-phosphate pyrophosphokinase 1 OS=Lactococcus lactis subsp. lactis (strain IL1403) OX=272623 GN=prs1 PE=3 SV=1
286	1	lactococcus	Q9CHI9	121	18846	2	2	2	2	0.55	Transcriptional regulator OS=Lactococcus lactis subsp. lactis (strain IL1403) OX=272623 GN=rmaA PE=4 SV=1
287	1	lactococcus	Q9CI77	120	19486	8	8	3	3	0.89	UPF0340 protein LL0489 OS=Lactococcus lactis subsp. lactis (strain IL1403) OX=272623 GN=yeiF PE=3 SV=1
288	1	lactococcus	Q9CEH3	120	25682	3	3	2	2	0.38	Pyridoxal phosphate homeostasis protein OS=Lactococcus lactis subsp. lactis (strain IL1403) OX=272623 GN=ytdF PE=3 SV=1
289	1	lactococcus	Q9CEW0	119	96422	3	3	3	3	0.14	Alanine--tRNA ligase OS=Lactococcus lactis subsp. lactis (strain IL1403) OX=272623 GN=alaS PE=3 SV=1
291	1	lactococcus	Q9CE78	117	49065	5	5	4	4	0.41	Histidine--tRNA ligase OS=Lactococcus lactis subsp. lactis (strain IL1403) OX=272623 GN=hisS PE=3 SV=1
292	1	lactococcus	Q9CDW9	115	15347	3	3	2	2	0.72	50S ribosomal protein L16 OS=Lactococcus lactis subsp. lactis (strain IL1403) OX=272623 GN=rplP PE=3 SV=1
293	1	lactococcus	Q9CDG0	113	17672	2	2	2	2	0.60	30S ribosomal protein S7 OS=Lactococcus lactis subsp. lactis (strain IL1403) OX=272623 GN=rpsG PE=3 SV=1
294	1	lactococcus	Q9CDM0	113	7492	2	2	1	1	0.72	Uncharacterized protein OS=Lactococcus lactis subsp. lactis (strain IL1403) OX=272623 GN=ywiE PE=4 SV=1
295	1	lactococcus	Q9CEP8	112	52658	3	3	3	3	0.27	Uncharacterized protein OS=Lactococcus lactis subsp. lactis (strain IL1403) OX=272623 GN=yseF PE=3 SV=1
296	1	lactococcus	Q9CHG1	111	16890	2	2	2	2	0.63	Transcriptional regulator OS=Lactococcus lactis subsp. lactis (strain IL1403) OX=272623 GN=rmaG PE=4 SV=1
297	1	lactococcus	Q9CFW9	111	26181	3	3	2	2	0.38	Orotidine 5'-phosphate decarboxylase OS=Lactococcus lactis subsp. lactis (strain IL1403) OX=272623 GN=pyrF PE=3 SV=1
298	1	lactococcus	Q9CFL6	111	23053	4	4	4	4	44932	GntR family transcriptional regulator OS=Lactococcus lactis subsp. lactis (strain IL1403) OX=272623 GN=busR PE=4 SV=1
299	1	lactococcus	Q9CHI4	110	9828	3	3	1	1	44954	Uncharacterized protein OS=Lactococcus lactis subsp. lactis (strain IL1403) OX=272623 GN=yhfC PE=4 SV=1
300	1	lactococcus	Q9CF19	110	20548	2	2	2	2	0.50	Cpl-7 domain-containing protein OS=Lactococcus lactis subsp. lactis (strain IL1403) OX=272623 GN=yrbB PE=4 SV=1
301	1	lactococcus	Q9CHU2	109	33602	3	3	3	3	0.45	tRNA dimethylallyltransferase OS=Lactococcus lactis subsp. lactis (strain IL1403) OX=272623 GN=miaA PE=3 SV=1
302	1	lactococcus	Q9CJH5	108	50338	4	4	3	3	0.29	GW domain-containing protein OS=Lactococcus lactis subsp. lactis (strain IL1403) OX=272623 GN=yacG PE=4 SV=1
303	1	lactococcus	Q9CDU6	108	30752	3	3	3	3	0.50	Zinc ABC transporter substrate binding protein OS=Lactococcus lactis subsp. lactis (strain IL1403) OX=272623 GN=zitS PE=3 SV=1

304	1	lactococcus	Q9CID9	107	48442	4	4	3	3	0.42	Phosphoglucosamine mutase OS=Lactococcus lactis subsp. lactis (strain IL1403) OX=272623 GN=glmM PE=3 SV=1
305	1	lactococcus	Q9CID3	107	24568	3	3	1	1	0.41	Uncharacterized protein OS=Lactococcus lactis subsp. lactis (strain IL1403) OX=272623 GN=yeeC PE=4 SV=1
306	1	lactococcus	Q9CEB4	99	39432	3	3	2	2	0.24	Phenylalanine--tRNA ligase alpha subunit OS=Lactococcus lactis subsp. lactis (strain IL1403) OX=272623 GN=pheS PE=3 SV=1
307	1	lactococcus	Q9CF30	94	43307	4	4	3	3	0.34	THUMP domain-containing protein OS=Lactococcus lactis subsp. lactis (strain IL1403) OX=272623 GN=yqjE PE=4 SV=1
308	1	lactococcus	Q9CDX8	92	19245	1	1	1	1	0.24	50S ribosomal protein L6 OS=Lactococcus lactis subsp. lactis (strain IL1403) OX=272623 GN=rplF PE=3 SV=1
309	1	lactococcus	Q9CEY0	91	18403	4	4	2	2	0.57	LysM domain-containing protein OS=Lactococcus lactis subsp. lactis (strain IL1403) OX=272623 GN=yrgA PE=4 SV=1
310	1	lactococcus	Q9CDG6	90	16185	2	2	1	1	0.29	50S ribosomal protein L13 OS=Lactococcus lactis subsp. lactis (strain IL1403) OX=272623 GN=rplM PE=3 SV=1
311	1	lactococcus	Q9CFY8	89	34651	2	2	1	1	0.13	2-dehydropantoate 2-reductase OS=Lactococcus lactis subsp. lactis (strain IL1403) OX=272623 GN=LL1323 PE=3 SV=1
312	1	lactococcus	Q9CI03	87	16832	2	2	2	2	0.63	3-hydroxyacyl-[acyl-carrier-protein] dehydratase FabZ OS=Lactococcus lactis subsp. lactis (strain IL1403) OX=272623 GN=fabZ1 PE=3 SV=1
313	1	lactococcus	P0A4J1	87	23240	1	1	1	1	0.20	Superoxide dismutase [Mn] OS=Lactococcus lactis subsp. lactis (strain IL1403) OX=272623 GN=sodA PE=3 SV=1
314	1	lactococcus	Q9CEF4	87	24211	2	2	1	1	0.19	Protein serine/threonine phosphatase OS=Lactococcus lactis subsp. lactis (strain IL1403) OX=272623 GN=pppL PE=4 SV=1
315	1	lactococcus	Q9CDJ9	86	57839	4	4	4	4	0.34	Glucose-6-phosphate 1-dehydrogenase OS=Lactococcus lactis subsp. lactis (strain IL1403) OX=272623 GN=zwf PE=3 SV=1
316	1	lactococcus	Q9CE41	85	32293	2	2	2	2	0.30	S1 motif domain-containing protein OS=Lactococcus lactis subsp. lactis (strain IL1403) OX=272623 GN=yuiC PE=3 SV=1
317	1	lactococcus	Q9CIR4	85	26428	1	1	1	1	0.17	TsaD domain-containing protein OS=Lactococcus lactis subsp. lactis (strain IL1403) OX=272623 GN=ycjB PE=4 SV=1
318	1	lactococcus	Q9CF42	84	32727	1	1	1	1	0.14	Ribokinase OS=Lactococcus lactis subsp. lactis (strain IL1403) OX=272623 GN=rbsK PE=3 SV=1
319	1	lactococcus	Q9CIC9	83	49483	3	3	3	3	0.29	Transcriptional regulator OS=Lactococcus lactis subsp. lactis (strain IL1403) OX=272623 GN=yeeG PE=3 SV=1
320	1	lactococcus	Q9CFB4	83	36597	5	5	1	1	0.12	Coproporphyrin III ferrochelatase OS=Lactococcus lactis subsp. lactis (strain IL1403) OX=272623 GN=cpfC PE=3 SV=1
321	1	lactococcus	Q9CDW8	83	24019	2	2	2	2	0.42	30S ribosomal protein S3 OS=Lactococcus lactis subsp. lactis (strain IL1403) OX=272623 GN=rpsC PE=3 SV=1
322	1	lactococcus	P0A493	81	4556	2	2	1	1	1.36	50S ribosomal protein L36 OS=Lactococcus lactis subsp. lactis (strain IL1403) OX=272623 GN=rpmJ PE=3 SV=1
323	1	lactococcus	Q9CI75	81	59698	2	2	2	2	0.15	CTP synthase OS=Lactococcus lactis subsp. lactis (strain IL1403) OX=272623 GN=pyrG PE=3 SV=1
324	1	lactococcus	Q9CJ87	81	18683	1	1	1	1	0.25	Transcriptional regulator OS=Lactococcus lactis subsp. lactis (strain IL1403) OX=272623 GN=rmaD PE=4 SV=1
325	1	lactococcus	Q9CIJ5	80	24924	3	3	2	2	0.40	RelA_SpoT domain-containing protein OS=Lactococcus lactis subsp. lactis (strain IL1403) OX=272623 GN=ydgI PE=4 SV=1
326	1	lactococcus	Q9CJI9	80	138639	2	2	2	2	0.06	ATP-dependent helicase/nuclease subunit A OS=Lactococcus lactis subsp. lactis (strain IL1403) OX=272623 GN=addA PE=3 SV=1
327	1	lactococcus	Q9CJ43	80	52039	2	2	2	2	0.18	Glutamyl-tRNA(Gln) amidotransferase subunit A OS=Lactococcus lactis subsp. lactis (strain IL1403) OX=272623 GN=gatA PE=3 SV=1
328	1	lactococcus	Q9CH33	79	17714	1	1	1	1	0.26	Transcriptional regulator OS=Lactococcus lactis subsp. lactis (strain IL1403) OX=272623 GN=rmaH PE=4 SV=1
329	1	lactococcus	Q9CJ34	78	40698	2	2	2	2	0.23	N-acetyltransferase domain-containing protein OS=Lactococcus lactis subsp. lactis (strain IL1403) OX=272623 GN=ybhD PE=4 SV=1
330	1	lactococcus	Q9CEL2	78	12697	1	1	1	1	0.38	MazG domain-containing protein OS=Lactococcus lactis subsp. lactis (strain IL1403) OX=272623 GN=ysjD PE=4 SV=1
331	1	lactococcus	Q9CH85	78	31120	1	1	1	1	0.14	Bifunctional protein FolD OS=Lactococcus lactis subsp. lactis (strain IL1403) OX=272623 GN=folD PE=3 SV=2

332	1	lactococcus	Q9CJ38	77	7119	3	3	2	2	44967	Major cold shock protein OS=Lactococcus lactis subsp. lactis (strain IL1403) OX=272623 GN=cspE PE=4 SV=1
333	1	lactococcus	Q9CFE7	77	24328	1	1	1	1	0.19	Phosphoribosylformylglycinamidine synthase subunit PurQ OS=Lactococcus lactis subsp. lactis (strain IL1403) OX=272623 GN=purQ PE=3 SV=1
334	1	lactococcus	Q9CJ13	76	24461	2	2	2	2	0.41	Uncharacterized protein OS=Lactococcus lactis subsp. lactis (strain IL1403) OX=272623 GN=ybjB PE=4 SV=1
335	1	lactococcus	Q9CGY0	76	35767	3	3	3	3	0.42	Putative gluconeogenesis factor OS=Lactococcus lactis subsp. lactis (strain IL1403) OX=272623 GN=yjiF PE=3 SV=1
336	1	lactococcus	Q9CI68	75	30586	2	2	2	2	0.31	DegV domain-containing protein YejH OS=Lactococcus lactis subsp. lactis (strain IL1403) OX=272623 GN=yejH PE=3 SV=2
337	1	lactococcus	P0A2Z0	74	17992	2	2	2	2	0.59	ATP synthase subunit b OS=Lactococcus lactis subsp. lactis (strain IL1403) OX=272623 GN=atpF PE=3 SV=1
339	1	lactococcus	Q9CFE6	73	9735	2	2	2	2	44956	Phosphoribosylformylglycinamidine synthase subunit PurS OS=Lactococcus lactis subsp. lactis (strain IL1403) OX=272623 GN=yphF PE=3 SV=1
340	1	lactococcus	Q9CDL0	73	26377	1	1	1	1	0.17	Uncharacterized protein OS=Lactococcus lactis subsp. lactis (strain IL1403) OX=272623 GN=ywjA PE=4 SV=1
341	1	lactococcus	Q9CES8	73	39465	1	1	1	1	0.11	3-dehydroquinate synthase OS=Lactococcus lactis subsp. lactis (strain IL1403) OX=272623 GN=aroB PE=3 SV=1
342	1	lactococcus	Q9CEX5	73	14301	1	1	1	1	0.33	Uncharacterized protein OS=Lactococcus lactis subsp. lactis (strain IL1403) OX=272623 GN=yrgE PE=4 SV=1
343	1	lactococcus	Q9CGZ9	72	21568	1	1	1	1	0.21	Isochorismatase domain-containing protein OS=Lactococcus lactis subsp. lactis (strain IL1403) OX=272623 GN=yjgF PE=4 SV=1
344	1	lactococcus	Q9CG25	71	52700	2	2	2	2	0.17	Glutamate synthase small subunit OS=Lactococcus lactis subsp. lactis (strain IL1403) OX=272623 GN=gltD PE=4 SV=1
345	1	lactococcus	P58121	70	15367	1	1	1	1	0.31	50S ribosomal protein L15 OS=Lactococcus lactis subsp. lactis (strain IL1403) OX=272623 GN=rplO PE=3 SV=1
346	1	lactococcus	Q9CET9	70	39471	1	1	1	1	0.11	Prephenate dehydrogenase OS=Lactococcus lactis subsp. lactis (strain IL1403) OX=272623 GN=tyrA PE=3 SV=1
347	1	lactococcus	Q9CEY4	70	38050	2	2	1	1	0.12	Putrescine carbamoyltransferase OS=Lactococcus lactis subsp. lactis (strain IL1403) OX=272623 GN=ptcA PE=3 SV=1
348	1	lactococcus	Q9CIN0	68	31201	2	2	1	1	0.14	ABC transporter permease protein OS=Lactococcus lactis subsp. lactis (strain IL1403) OX=272623 GN=ydcF PE=4 SV=1
349	1	lactococcus	Q9CIS3	68	41735	3	3	2	2	0.22	Endo-1,4-beta-xylanase D OS=Lactococcus lactis subsp. lactis (strain IL1403) OX=272623 GN=xynD PE=4 SV=1
350	1	lactococcus	Q9CGX3	67	21404	1	1	1	1	0.21	UPF0374 protein YjjG OS=Lactococcus lactis subsp. lactis (strain IL1403) OX=272623 GN=yjjG PE=3 SV=1
351	1	lactococcus	Q9CEP7	66	13037	2	2	2	2	0.88	NifU protein OS=Lactococcus lactis subsp. lactis (strain IL1403) OX=272623 GN=nifU PE=4 SV=1
352	1	lactococcus	Q9CGI3	65	32414	1	1	1	1	0.14	Uncharacterized protein OS=Lactococcus lactis subsp. lactis (strain IL1403) OX=272623 GN=ylcC PE=4 SV=1
353	1	lactococcus	Q9CFX5	64	34851	2	2	2	2	0.27	UTP--glucose-1-phosphate uridylyltransferase OS=Lactococcus lactis subsp. lactis (strain IL1403) OX=272623 GN=hasC PE=3 SV=1
354	1	lactococcus	Q9CEF8	64	49195	1	1	1	1	0.09	Bifunctional protein GlmU OS=Lactococcus lactis subsp. lactis (strain IL1403) OX=272623 GN=glmU PE=3 SV=1
355	1	lactococcus	Q9CEW9	64	24507	2	2	2	2	0.41	Phosphate-specific transport system accessory protein PhoU OS=Lactococcus lactis subsp. lactis (strain IL1403) OX=272623 GN=phoU PE=3 SV=1
356	1	lactococcus	Q9CHM1	64	34378	1	1	1	1	0.13	Transcriptional regulator OS=Lactococcus lactis subsp. lactis (strain IL1403) OX=272623 GN=rmaB PE=4 SV=1
357	1	lactococcus	Q9CEE5	63	89287	4	4	3	3	0.15	Primosomal protein N' OS=Lactococcus lactis subsp. lactis (strain IL1403) OX=272623 GN=priA PE=3 SV=1
358	1	lactococcus	Q9CF63	63	63531	1	1	1	1	0.07	Ribonuclease J OS=Lactococcus lactis subsp. lactis (strain IL1403) OX=272623 GN=yqgA PE=3 SV=1
359	1	lactococcus	Q9CHF3	63	49652	1	1	1	1	0.09	Biotin carboxylase OS=Lactococcus lactis subsp. lactis (strain IL1403) OX=272623 GN=accC PE=4 SV=1
360	1	lactococcus	Q9CGY1	62	33616	1	1	1	1	0.13	Nucleotide-binding protein YjiE OS=Lactococcus lactis subsp. lactis (strain IL1403) OX=272623 GN=yjiE PE=3 SV=1

361	1	lactococcus	Q9CEU9	61	25093	2	2	2	2	0.39	LUD_dom domain-containing protein OS=Lactococcus lactis subsp. lactis (strain IL1403) OX=272623 GN=yrjD PE=4 SV=1
362	1	lactococcus	Q9CI63	61	44023	3	3	1	1	0.10	Prophage ps2 probable integrase OS=Lactococcus lactis subsp. lactis (strain IL1403) OX=272623 GN=ps201 PE=3 SV=1
363	1	lactococcus	Q9CEE1	61	26574	1	1	1	1	0.17	Glucosamine_iso domain-containing protein OS=Lactococcus lactis subsp. lactis (strain IL1403) OX=272623 GN=ytgG PE=4 SV=1
364	1	lactococcus	Q9CHW6	61	22331	1	1	1	1	0.21	Transposon-related protein OS=Lactococcus lactis subsp. lactis (strain IL1403) OX=272623 GN=yfjB PE=4 SV=1
365	1	lactococcus	Q9CIE9	60	12806	1	1	1	1	0.38	PTS system cellobiose-specific EIIA component OS=Lactococcus lactis subsp. lactis (strain IL1403) OX=272623 GN=ptcA PE=2 SV=1
366	1	lactococcus	Q9CIV3	59	24332	2	2	1	1	0.19	Transcriptional regulator OS=Lactococcus lactis subsp. lactis (strain IL1403) OX=272623 GN=ycfA PE=4 SV=1
367	1	lactococcus	Q9CHC7	59	82023	1	1	1	1	0.05	Sulfatase domain-containing protein OS=Lactococcus lactis subsp. lactis (strain IL1403) OX=272623 GN=yibC PE=3 SV=1
368	1	lactococcus	Q9CGT1	58	20738	1	1	1	1	0.22	HTH cro/C1-type domain-containing protein OS=Lactococcus lactis subsp. lactis (strain IL1403) OX=272623 GN=pi204 PE=4 SV=1
369	1	lactococcus	Q9CGM7	58	45740	1	1	1	1	0.10	Dihydroorotase OS=Lactococcus lactis subsp. lactis (strain IL1403) OX=272623 GN=pyrC PE=3 SV=2
370	1	lactococcus	Q9CI65	58	24601	1	1	1	1	0.18	Uncharacterized protein OS=Lactococcus lactis subsp. lactis (strain IL1403) OX=272623 GN=yfaA PE=4 SV=1
371	1	lactococcus	Q9CEP3	58	28407	1	1	1	1	0.16	ABC transporter ATP-binding protein OS=Lactococcus lactis subsp. lactis (strain IL1403) OX=272623 GN=ysfB PE=3 SV=1
372	1	lactococcus	Q9CE71	57	13591	1	1	1	1	0.36	S4 RNA-binding domain-containing protein OS=Lactococcus lactis subsp. lactis (strain IL1403) OX=272623 GN=yufA PE=4 SV=1
373	1	lactococcus	Q9CIN6	57	31534	2	2	2	2	0.30	Lipoprotein OS=Lactococcus lactis subsp. lactis (strain IL1403) OX=272623 GN=plpC PE=3 SV=1
374	1	lactococcus	Q9CEJ8	57	7871	2	2	1	1	0.67	50S ribosomal protein L35 OS=Lactococcus lactis subsp. lactis (strain IL1403) OX=272623 GN=rpmI PE=3 SV=1
375	1	lactococcus	Q9CI29	57	41821	1	1	1	1	0.11	Pyridoxal-phosphate dependent aminotransferase OS=Lactococcus lactis subsp. lactis (strain IL1403) OX=272623 GN=nifZ PE=3 SV=1
376	1	lactococcus	P46469	57	75513	2	2	2	2	0.12	ATP-dependent zinc metalloprotease FtsH OS=Lactococcus lactis subsp. lactis (strain IL1403) OX=272623 GN=ftsH PE=3 SV=1
377	1	lactococcus	Q9CF50	57	41029	1	1	1	1	0.11	Mannonate dehydratase OS=Lactococcus lactis subsp. lactis (strain IL1403) OX=272623 GN=uxuA PE=3 SV=1
378	1	lactococcus	Q9CIR5	55	8836	2	2	2	2	1.49	DNA-directed RNA polymerase subunit epsilon OS=Lactococcus lactis subsp. lactis (strain IL1403) OX=272623 GN=rpoY PE=3 SV=1
379	1	lactococcus	Q9CDQ7	55	37357	1	1	1	1	0.12	Endopeptidase La OS=Lactococcus lactis subsp. lactis (strain IL1403) OX=272623 GN=ywdF PE=3 SV=1
380	1	lactococcus	Q9CGM0	54	52260	1	1	1	1	0.08	HD domain-containing protein OS=Lactococcus lactis subsp. lactis (strain IL1403) OX=272623 GN=ykiG PE=4 SV=1
381	1	lactococcus	Q9CHJ6	54	34642	2	2	2	2	0.27	Uncharacterized protein OS=Lactococcus lactis subsp. lactis (strain IL1403) OX=272623 GN=yheB PE=4 SV=1
382	1	lactococcus	Q9CE11	54	16929	1	1	1	1	0.28	Arginine repressor OS=Lactococcus lactis subsp. lactis (strain IL1403) OX=272623 GN=argR PE=3 SV=1
383	1	lactococcus	Q9CEV1	54	28746	1	1	1	1	0.16	Oxidoreductase OS=Lactococcus lactis subsp. lactis (strain IL1403) OX=272623 GN=yrjB PE=4 SV=1
384	1	lactococcus	Q9CGM5	54	24799	1	1	1	1	0.18	Endonuclease III OS=Lactococcus lactis subsp. lactis (strain IL1403) OX=272623 GN=nth PE=3 SV=1
385	1	lactococcus	Q9CH81	53	31334	1	1	1	1	0.14	Farnesyl diphosphate synthase OS=Lactococcus lactis subsp. lactis (strain IL1403) OX=272623 GN=ispA PE=3 SV=1
386	1	lactococcus	Q9CI78	53	17742	2	2	1	1	0.26	Uncharacterized protein OS=Lactococcus lactis subsp. lactis (strain IL1403) OX=272623 GN=yeiE PE=4 SV=1
387	1	lactococcus	Q9CE24	53	14416	2	2	1	1	0.33	Rhodanese domain-containing protein OS=Lactococcus lactis subsp. lactis (strain IL1403) OX=272623 GN=yvaB PE=4 SV=1
388	1	lactococcus	Q9CDJ4	52	27800	2	2	1	1	0.16	Aspartate racemase OS=Lactococcus lactis subsp. lactis (strain IL1403) OX=272623 GN=racD PE=3 SV=1
389	1	lactococcus	Q9CJI7	52	41228	1	1	1	1	0.11	Ribosome-binding ATPase YchF OS=Lactococcus lactis subsp. lactis (strain IL1403) OX=272623 GN=yyaL PE=3 SV=1

390	1	lactococcus	Q9CJC7	50	34764	1	1	1	1	0.13	Phosphate acyltransferase OS=Lactococcus lactis subsp. lactis (strain IL1403) OX=272623 GN=plsX PE=3 SV=1
391	1	lactococcus	Q9CDM1	50	16717	1	1	1	1	0.28	Uncharacterized protein OS=Lactococcus lactis subsp. lactis (strain IL1403) OX=272623 GN=ywiD PE=4 SV=1
392	1	lactococcus	Q9CF77	49	19819	1	1	1	1	0.23	Pyrimidine operon regulatory protein OS=Lactococcus lactis subsp. lactis (strain IL1403) OX=272623 GN=pyrR PE=3 SV=1
393	1	lactococcus	Q9CF53	48	54563	1	1	1	1	0.08	Uronate isomerase OS=Lactococcus lactis subsp. lactis (strain IL1403) OX=272623 GN=uxaC PE=3 SV=1
394	1	lactococcus	Q9CII1	48	45333	1	1	1	1	0.10	Probable tRNA sulfurtransferase OS=Lactococcus lactis subsp. lactis (strain IL1403) OX=272623 GN=thiI PE=3 SV=1
395	1	lactococcus	Q9CJ50	47	28837	1	1	1	1	0.16	Endonuclease_NS domain-containing protein OS=Lactococcus lactis subsp. lactis (strain IL1403) OX=272623 GN=ybfB PE=4 SV=1
396	1	lactococcus	Q9CDV8	47	55233	1	1	1	1	0.08	Threonine synthase OS=Lactococcus lactis subsp. lactis (strain IL1403) OX=272623 GN=thrC PE=3 SV=1
397	1	lactococcus	Q9R7D7	47	43889	1	1	1	1	0.10	Galactokinase OS=Lactococcus lactis subsp. lactis (strain IL1403) OX=272623 GN=galK PE=1 SV=1
398	1	lactococcus	Q9CH31	47	26901	1	1	1	1	0.17	Uncharacterized protein OS=Lactococcus lactis subsp. lactis (strain IL1403) OX=272623 GN=yjdF PE=4 SV=1
399	1	lactococcus	Q9CFB2	47	10275	1	1	1	1	0.49	30S ribosomal protein S16 OS=Lactococcus lactis subsp. lactis (strain IL1403) OX=272623 GN=rpsP PE=3 SV=1
400	1	lactococcus	Q9CDW1	46	11734	1	1	1	1	0.42	30S ribosomal protein S10 OS=Lactococcus lactis subsp. lactis (strain IL1403) OX=272623 GN=rpsJ PE=3 SV=1
401	1	lactococcus	Q9CDX5	46	7314	1	1	1	1	0.73	30S ribosomal protein S14 type Z OS=Lactococcus lactis subsp. lactis (strain IL1403) OX=272623 GN=rpsZ PE=3 SV=1
402	1	lactococcus	Q9CEL0	45	19180	1	1	1	1	0.24	Uncharacterized protein OS=Lactococcus lactis subsp. lactis (strain IL1403) OX=272623 GN=ysjF PE=4 SV=1
403	1	lactococcus	Q02141	45	55907	1	1	1	1	0.08	2-isopropylmalate synthase OS=Lactococcus lactis subsp. lactis (strain IL1403) OX=272623 GN=leuA PE=3 SV=1
404	1	lactococcus	Q9CES5	44	78328	1	1	1	1	0.06	Glutamine ABC transporter permease and substrate binding protein protein OS=Lactococcus lactis subsp. lactis (strain IL1403) OX=272623 GN=glnP PE=1 SV=1
406	1	lactococcus	Q9CJC2	43	16671	1	1	1	1	0.28	Usp domain-containing protein OS=Lactococcus lactis subsp. lactis (strain IL1403) OX=272623 GN=yahB PE=3 SV=1
407	1	lactococcus	Q9CGD8	43	46891	1	1	1	1	0.09	Homoserine dehydrogenase OS=Lactococcus lactis subsp. lactis (strain IL1403) OX=272623 GN=hom PE=3 SV=1
408	1	lactococcus	Q9CI80	43	29127	1	1	1	1	0.15	tRNA pseudouridine synthase A OS=Lactococcus lactis subsp. lactis (strain IL1403) OX=272623 GN=truA PE=3 SV=1
409	1	lactococcus	Q9CJJ0	42	127114	1	1	1	1	0.03	ATP-dependent helicase/deoxyribonuclease subunit B OS=Lactococcus lactis subsp. lactis (strain IL1403) OX=272623 GN=rexB PE=3 SV=1
410	1	lactococcus	Q9CFS7	41	176213	1	1	1	1	0.02	Prophage pi3 protein 14 OS=Lactococcus lactis subsp. lactis (strain IL1403) OX=272623 GN=pi314 PE=4 SV=1
411	1	lactococcus	Q9CHM8	41	91893	1	1	1	1	0.05	Alpha-1,4 glucan phosphorylase OS=Lactococcus lactis subsp. lactis (strain IL1403) OX=272623 GN=glgP PE=3 SV=1
413	1	lactococcus	Q9CIX8	40	12845	1	1	1	1	0.38	Ribosomal silencing factor RsfS OS=Lactococcus lactis subsp. lactis (strain IL1403) OX=272623 GN=yccJ PE=3 SV=1
414	1	lactococcus	Q9CJ41	40	53438	1	1	1	1	0.08	Aspartyl/glutamyl-tRNA(Asn/Gln) amidotransferase subunit B OS=Lactococcus lactis subsp. lactis (strain IL1403) OX=272623 GN=gatB PE=3 SV=2
415	1	lactococcus	Q9CJ44	40	11206	2	2	1	1	0.44	Glutamyl-tRNA(Gln) amidotransferase subunit C OS=Lactococcus lactis subsp. lactis (strain IL1403) OX=272623 GN=gatC PE=3 SV=1
416	1	lactococcus	Q9CEK5	39	89889	1	1	1	1	0.05	3'-5' exonuclease DinG OS=Lactococcus lactis subsp. lactis (strain IL1403) OX=272623 GN=dinG PE=3 SV=1
417	1	lactococcus	O34133	39	13750	1	1	1	1	0.35	Putative regulator AldR OS=Lactococcus lactis subsp. lactis (strain IL1403) OX=272623 GN=aldR PE=3 SV=2
418	1	lactococcus	Q9CEV0	39	54564	1	1	1	1	0.08	Iron-binding oxidase subunit OS=Lactococcus lactis subsp. lactis (strain IL1403) OX=272623 GN=yrjC PE=4 SV=1
419	1	lactococcus	Q9CFU3	39	15351	1	1	1	1	0.31	Usp domain-containing protein OS=Lactococcus lactis subsp. lactis (strain IL1403) OX=272623 GN=yobA PE=3 SV=1

420	1	lactococcus	Q9CEY1	39	24533	1	1	1	1	0.19	Cytidylate kinase OS=Lactococcus lactis subsp. lactis (strain IL1403) OX=272623 GN=cmk PE=3 SV=1
421	1	lactococcus	Q9CIS8	39	31875	1	1	1	1	0.14	Energy-coupling factor transporter ATP-binding protein EcfA2 OS=Lactococcus lactis subsp. lactis (strain IL1403) OX=272623 GN=ecfA2 PE=3 SV=1
422	1	lactococcus	Q9CDY4	38	14247	1	1	1	1	0.34	50S ribosomal protein L17 OS=Lactococcus lactis subsp. lactis (strain IL1403) OX=272623 GN=rplQ PE=3 SV=1
423	1	lactococcus	Q9CDU5	38	16403	1	1	1	1	0.29	Zinc transport transcriptional regulator OS=Lactococcus lactis subsp. lactis (strain IL1403) OX=272623 GN=zitR PE=1 SV=1
424	1	lactococcus	Q9CFW2	38	21257	1	1	1	1	0.22	Pseudouridine synthase OS=Lactococcus lactis subsp. lactis (strain IL1403) OX=272623 GN=rluC PE=3 SV=1
425	1	lactococcus	Q9CHI6	38	16365	1	1	1	1	0.29	50S ribosomal protein L9 OS=Lactococcus lactis subsp. lactis (strain IL1403) OX=272623 GN=rplI PE=3 SV=1
426	1	lactococcus	Q9CIS5	37	26760	1	1	1	1	0.17	2,3,4,5-tetrahydropyridine-2,6-dicarboxylate N-acetyltransferase OS=Lactococcus lactis subsp. lactis (strain IL1403) OX=272623 GN=dapH PE=3 SV=1
427	1	lactococcus	Q9CF85	37	9331	1	1	1	1	0.55	50S ribosomal protein L31 type B OS=Lactococcus lactis subsp. lactis (strain IL1403) OX=272623 GN=rpmE2 PE=3 SV=1

Proteins identified in active fractions obtained from Resource Q (IEX) column

Family	Member	Database	Accession	Score	Mass	Num. of matches	Num. of significant matches	Num. of sequences	Num. of significant sequences	emPAI	Description
1	1	lactococcus	P0A3J0	25457	64947	706	705	78	78	283.24	Chaperone protein DnaK OS=Lactococcus lactis subsp. lactis (strain IL1403) OX=272623 GN=dnaK PE=3 SV=1
4	1	lactococcus	Q9CI09	4823	83277	136	136	44	44	9.85	ATP-dependent Clp protease ATP-binding subunit ClpE OS=Lactococcus lactis subsp. lactis (strain IL1403) OX=272623 GN=clpE PE=3 SV=1
2	1	lactococcus	Q9CHA0	5925	44683	187	187	42	42	74.52	30S ribosomal protein S1 OS=Lactococcus lactis subsp. lactis (strain IL1403) OX=272623 GN=rpsA PE=4 SV=1
7	1	lactococcus	Q9CJ82	3138	62613	112	111	32	31	9.56	Phosphoenolpyruvate-protein phosphotransferase OS=Lactococcus lactis subsp. lactis (strain IL1403) OX=272623 GN=ptsI PE=3 SV=1
3	1	lactococcus	Q01462	5592	35075	195	195	30	30	39.75	L-lactate dehydrogenase 1 OS=Lactococcus lactis subsp. lactis (strain IL1403) OX=272623 GN=ldh1 PE=3 SV=3
11	1	lactococcus	Q9CHS7	2040	46929	61	61	22	22	6.85	Enolase 1 OS=Lactococcus lactis subsp. lactis (strain IL1403) OX=272623 GN=eno1 PE=3 SV=1
20	1	lactococcus	Q9CF40	1344	49702	52	52	21	21	6.62	Adenylosuccinate lyase OS=Lactococcus lactis subsp. lactis (strain IL1403) OX=272623 GN=purB PE=3 SV=1
19	1	lactococcus	Q9CHU6	1355	52484	46	46	21	21	6.43	6-phosphogluconate dehydrogenase, decarboxylating OS=Lactococcus lactis subsp. lactis (strain IL1403) OX=272623 GN=gnd PE=3 SV=1
25	1	lactococcus	P58013	1042	46106	40	40	18	18	6.46	Arginine deiminase OS=Lactococcus lactis subsp. lactis (strain IL1403) OX=272623 GN=arcA PE=3 SV=1
6	1	lactococcus	Q9CF79	3178	34674	91	91	18	18	25.31	Aspartate carbamoyltransferase OS=Lactococcus lactis subsp. lactis (strain IL1403) OX=272623 GN=pyrB PE=3 SV=1
12	1	lactococcus	Q9CF22	1991	35326	64	64	18	18	11.13	Phosphate acetyltransferase OS=Lactococcus lactis subsp. lactis (strain IL1403) OX=272623 GN=pta PE=3 SV=1
13	1	lactococcus	Q9CED6	1965	41036	61	61	18	18	5.31	DUF4097 domain-containing protein OS=Lactococcus lactis subsp. lactis (strain IL1403) OX=272623 GN=ythC PE=4 SV=1
33	1	lactococcus	Q9CH07	606	59543	26	26	16	16	2.11	Formate--tetrahydrofolate ligase OS=Lactococcus lactis subsp. lactis (strain IL1403) OX=272623 GN=fhs PE=3 SV=1
37	1	lactococcus	O32797	529	89341	20	20	14	14	0.94	Formate acetyltransferase OS=Lactococcus lactis subsp. lactis (strain IL1403) OX=272623 GN=pfl PE=3 SV=1
21	1	lactococcus	Q9CHB9	1326	51036	44	44	14	14	2.17	Signal recognition particle receptor FtsY OS=Lactococcus lactis subsp. lactis (strain IL1403) OX=272623 GN=ftsY PE=3 SV=1
29	1	lactococcus	Q9CHU7	839	20647	38	38	14	14	19.57	Uncharacterized protein OS=Lactococcus lactis subsp. lactis (strain IL1403) OX=272623 GN=ygaJ PE=4 SV=1
8	1	lactococcus	Q9CIU0	3137	17686	84	84	13	13	42.00	S-ribosylhomocysteine lyase OS=Lactococcus lactis subsp. lactis (strain IL1403) OX=272623 GN=luxS PE=3 SV=1
17	1	lactococcus	Q9CI15	1490	46902	48	48	13	13	2.51	Trigger factor OS=Lactococcus lactis subsp. lactis (strain IL1403) OX=272623 GN=tig PE=3 SV=1
24	1	lactococcus	P50918	1082	27027	43	42	13	13	10.94	Triosephosphate isomerase OS=Lactococcus lactis subsp. lactis (strain IL1403) OX=272623 GN=tpiA PE=1 SV=3
36	1	lactococcus	Q9CE66	531	36299	20	20	12	12	3.00	UDP-glucose 4-epimerase OS=Lactococcus lactis subsp. lactis (strain IL1403) OX=272623 GN=galE PE=3 SV=1
15	1	lactococcus	P81181	1616	49564	56	56	12	12	1.77	Glucose-6-phosphate isomerase OS=Lactococcus lactis subsp. lactis (strain IL1403) OX=272623 GN=pgi PE=1 SV=3
31	1	lactococcus	Q9CE93	721	47480	23	23	12	12	1.89	Adenylosuccinate synthetase OS=Lactococcus lactis subsp. lactis (strain IL1403) OX=272623 GN=purA PE=3 SV=1
34	1	lactococcus	Q9CIU1	591	40276	23	23	11	11	3.30	Oxidoreductase OS=Lactococcus lactis subsp. lactis (strain IL1403) OX=272623 GN=ycgD PE=4 SV=1
22	1	lactococcus	Q9CE25	1170	34001	35	35	11	11	3.40	Glucokinase OS=Lactococcus lactis subsp. lactis (strain IL1403) OX=272623 GN=glk PE=3 SV=1
57	1	lactococcus	Q9CFJ0	313	56837	12	12	10	10	1.10	GMP synthase [glutamine-hydrolyzing] OS=Lactococcus lactis subsp. lactis (strain IL1403) OX=272623 GN=guaA PE=3 SV=1
14	1	lactococcus	Q9CEF4	1683	24211	52	52	9	9	5.67	Protein serine/threonine phosphatase OS=Lactococcus lactis subsp. lactis (strain IL1403) OX=272623 GN=pppL PE=4 SV=1
47	1	lactococcus	Q9CGJ6	388	56404	15	15	8	8	0.82	Nicotinate phosphoribosyltransferase OS=Lactococcus lactis subsp. lactis (strain IL1403) OX=272623 GN=ylaF PE=3 SV=1

16	1	lactococcus	P37283	1582	10214	50	50	8	8	80.28	10 kDa chaperonin OS=Lactococcus lactis subsp. lactis (strain IL1403) OX=272623 GN=groS PE=3 SV=1
39	1	lactococcus	P0A4K2	497	40912	22	22	8	8	1.27	Cystathionine beta-lyase OS=Lactococcus lactis subsp. lactis (strain IL1403) OX=272623 GN=metC PE=3 SV=1
32	1	lactococcus	Q48661	654	22125	23	23	8	8	4.46	Peptide deformylase OS=Lactococcus lactis subsp. lactis (strain IL1403) OX=272623 GN=def PE=3 SV=3
49	1	lactococcus	Q9CEP5	374	45333	12	12	8	8	1.10	Cysteine desulfurase OS=Lactococcus lactis subsp. lactis (strain IL1403) OX=272623 GN=yseI PE=3 SV=1
35	1	lactococcus	Q9CDL9	586	49800	18	18	8	8	0.97	Glutamine synthetase OS=Lactococcus lactis subsp. lactis (strain IL1403) OX=272623 GN=glnA PE=3 SV=1
68	1	lactococcus	Q9CDP6	189	100475	10	10	7	7	0.34	Valine--tRNA ligase OS=Lactococcus lactis subsp. lactis (strain IL1403) OX=272623 GN=valS PE=3 SV=1
26	1	lactococcus	Q9CG42	1033	12401	25	25	7	7	13.09	50S ribosomal protein L7/L12 OS=Lactococcus lactis subsp. lactis (strain IL1403) OX=272623 GN=rplL PE=3 SV=1
61	1	lactococcus	Q9CDY3	290	34173	11	11	7	7	1.36	DNA-directed RNA polymerase subunit alpha OS=Lactococcus lactis subsp. lactis (strain IL1403) OX=272623 GN=rpoA PE=3 SV=1
44	1	lactococcus	Q9CFB0	421	39827	12	12	7	7	1.32	Acetyl coenzyme A acetyltransferase OS=Lactococcus lactis subsp. lactis (strain IL1403) OX=272623 GN=thiL PE=3 SV=1
64	1	lactococcus	Q07637	253	54267	10	10	7	7	0.72	Pyruvate kinase OS=Lactococcus lactis subsp. lactis (strain IL1403) OX=272623 GN=pyk PE=3 SV=2
45	1	lactococcus	Q9CEI4	412	35348	16	16	7	7	1.30	Putative ribose-phosphate pyrophosphokinase 2 OS=Lactococcus lactis subsp. lactis (strain IL1403) OX=272623 GN=prs2 PE=3 SV=1
50	1	lactococcus	Q9CJ45	369	29202	11	11	7	7	1.73	Transcriptional regulator OS=Lactococcus lactis subsp. lactis (strain IL1403) OX=272623 GN=codY PE=3 SV=1
28	1	lactococcus	Q9CH83	937	8959	27	27	6	6	14.04	Exodeoxyribonuclease 7 small subunit OS=Lactococcus lactis subsp. lactis (strain IL1403) OX=272623 GN=xseB PE=3 SV=1
27	1	lactococcus	Q9CIM0	1025	26313	21	21	6	6	1.59	2,3-bisphosphoglycerate-dependent phosphoglycerate mutase OS=Lactococcus lactis subsp. lactis (strain IL1403) OX=272623 GN=gpmA PE=3 SV=1
58	1	lactococcus	Q9CID9	311	48442	15	15	6	6	1.19	Phosphoglucosamine mutase OS=Lactococcus lactis subsp. lactis (strain IL1403) OX=272623 GN=glmM PE=3 SV=1
30	1	lactococcus	P0A4J1	784	23240	24	24	6	6	1.94	Superoxide dismutase [Mn] OS=Lactococcus lactis subsp. lactis (strain IL1403) OX=272623 GN=sodA PE=3 SV=1
38	1	lactococcus	Q9CI64	515	9670	16	16	6	6	11.57	DNA-binding protein HU OS=Lactococcus lactis subsp. lactis (strain IL1403) OX=272623 GN=hup PE=1 SV=1
59	1	lactococcus	Q9CF28	303	14970	10	10	6	6	4.23	Uncharacterized protein OS=Lactococcus lactis subsp. lactis (strain IL1403) OX=272623 GN=yraB PE=4 SV=1
46	1	lactococcus	Q9CFC0	408	28473	12	12	6	6	1.79	4-hydroxy-tetrahydrodipicolinate reductase OS=Lactococcus lactis subsp. lactis (strain IL1403) OX=272623 GN=dapB PE=3 SV=1
60	1	lactococcus	Q9CH12	299	45787	10	10	6	6	0.74	Phosphopentomutase OS=Lactococcus lactis subsp. lactis (strain IL1403) OX=272623 GN=deoB PE=3 SV=1
85	1	lactococcus	P37282	121	57166	5	5	5	5	0.45	60 kDa chaperonin OS=Lactococcus lactis subsp. lactis (strain IL1403) OX=272623 GN=groL PE=3 SV=2
18	1	lactococcus	Q9CIV6	1380	13386	26	26	5	5	7.56	PTS-dependent dihydroxyacetone kinase, phosphotransferase subunit DhaM OS=Lactococcus lactis subsp. lactis (strain IL1403) OX=272623 GN=dhaM PE=1 SV=1
73	1	lactococcus	Q9CIR6	156	61569	5	5	5	5	0.41	Ribonuclease J OS=Lactococcus lactis subsp. lactis (strain IL1403) OX=272623 GN=yciH PE=3 SV=1
40	1	lactococcus	Q9CE53	495	22523	16	16	5	5	1.52	Copper homeostasis protein CutC OS=Lactococcus lactis subsp. lactis (strain IL1403) OX=272623 GN=yuhE PE=3 SV=1
72	1	lactococcus	Q9CDZ7	159	55427	7	7	5	5	0.46	Glutamate--tRNA ligase OS=Lactococcus lactis subsp. lactis (strain IL1403) OX=272623 GN=gltX PE=3 SV=1
52	1	lactococcus	Q9CIE9	364	12806	16	16	5	5	04.01	PTS system cellobiose-specific EIIA component OS=Lactococcus lactis subsp. lactis (strain IL1403) OX=272623 GN=ptcA PE=2 SV=1
80	1	lactococcus	Q9CIW1	134	42044	7	7	5	5	0.65	Phosphoglycerate kinase OS=Lactococcus lactis subsp. lactis (strain IL1403) OX=272623 GN=pgk PE=3 SV=1
42	1	lactococcus	Q9CE86	457	31883	9	9	5	5	0.93	33 kDa chaperonin OS=Lactococcus lactis subsp. lactis (strain IL1403) OX=272623 GN=hslO PE=3 SV=1

67	1	lactococcus	Q9CJ29	194	50677	7	7	5	5	0.51	Metallophos domain-containing protein OS=Lactococcus lactis subsp. lactis (strain IL1403) OX=272623 GN=ybiB PE=3 SV=1
63	1	lactococcus	Q9CHN6	258	20640	11	11	5	5	2.35	Elongation factor P OS=Lactococcus lactis subsp. lactis (strain IL1403) OX=272623 GN=efp PE=3 SV=1
75	1	lactococcus	Q9CFW8	152	33125	12	12	5	5	0.88	Dihydroorotate dehydrogenase B (NAD(+)), catalytic subunit OS=Lactococcus lactis subsp. lactis (strain IL1403) OX=272623 GN=pyrDB PE=1 SV=1
43	1	lactococcus	Q9CE23	447	16642	11	11	5	5	3.45	Non-heme iron-binding ferritin OS=Lactococcus lactis subsp. lactis (strain IL1403) OX=272623 GN=dpsA PE=3 SV=1
76	1	lactococcus	Q9CH96	144	52003	5	5	5	5	0.50	Dipeptidase OS=Lactococcus lactis subsp. lactis (strain IL1403) OX=272623 GN=pepV PE=4 SV=1
53	1	lactococcus	Q9CGY9	353	20568	14	14	5	5	2.37	Protein GrpE OS=Lactococcus lactis subsp. lactis (strain IL1403) OX=272623 GN=grpE PE=3 SV=1
55	1	lactococcus	Q9CED4	324	32153	14	14	5	5	0.92	Fructose-bisphosphate aldolase OS=Lactococcus lactis subsp. lactis (strain IL1403) OX=272623 GN=fbaA PE=4 SV=1
89	1	lactococcus	Q9CGY7	107	67403	4	4	4	4	0.28	Myosin-crossreactive antigen OS=Lactococcus lactis subsp. lactis (strain IL1403) OX=272623 GN=mycA PE=4 SV=1
23	1	lactococcus	Q9CDS4	1113	8614	31	31	4	4	15.62	Uncharacterized protein OS=Lactococcus lactis subsp. lactis (strain IL1403) OX=272623 GN=ywaH PE=4 SV=1
69	1	lactococcus	Q9CDR5	189	36647	6	6	4	4	0.58	Elongation factor Ts OS=Lactococcus lactis subsp. lactis (strain IL1403) OX=272623 GN=tsf PE=3 SV=1
48	1	lactococcus	Q9CFF0	376	54973	8	8	4	4	0.47	Amidophosphoribosyltransferase OS=Lactococcus lactis subsp. lactis (strain IL1403) OX=272623 GN=purF PE=3 SV=1
83	1	lactococcus	Q9CJD6	127	35188	6	6	4	4	0.61	PDH E1 component beta subunit OS=Lactococcus lactis subsp. lactis (strain IL1403) OX=272623 GN=pdhB PE=4 SV=1
51	1	lactococcus	Q9CH79	368	16861	8	8	4	4	1.67	Arginine repressor OS=Lactococcus lactis subsp. lactis (strain IL1403) OX=272623 GN=ahrC PE=3 SV=1
70	1	lactococcus	Q9CF20	186	36267	8	8	4	4	0.59	Oxidoreductase OS=Lactococcus lactis subsp. lactis (strain IL1403) OX=272623 GN=yrbA PE=4 SV=1
54	1	lactococcus	Q9CDH4	345	35889	12	12	4	4	0.80	Glyceraldehyde-3-phosphate dehydrogenase OS=Lactococcus lactis subsp. lactis (strain IL1403) OX=272623 GN=gapB PE=3 SV=1
94	1	lactococcus	Q9CGM8	100	22619	4	4	4	4	01.09	Orotate phosphoribosyltransferase OS=Lactococcus lactis subsp. lactis (strain IL1403) OX=272623 GN=pyrE PE=3 SV=1
77	1	lactococcus	Q9CIR4	142	26428	6	6	4	4	0.88	TsaD domain-containing protein OS=Lactococcus lactis subsp. lactis (strain IL1403) OX=272623 GN=ycjB PE=4 SV=1
71	1	lactococcus	Q9CGM3	177	28542	5	5	4	4	0.80	GTP cyclohydrolase 1 type 2 homolog OS=Lactococcus lactis subsp. lactis (strain IL1403) OX=272623 GN=ykiD PE=3 SV=1
95	1	lactococcus	Q9CFG0	100	57196	4	4	3	3	0.25	Bifunctional purine biosynthesis protein PurH OS=Lactococcus lactis subsp. lactis (strain IL1403) OX=272623 GN=purH PE=3 SV=1
81	1	lactococcus	Q9CJ64	132	10163	8	8	3	3	3.95	UPF0297 protein YbeA OS=Lactococcus lactis subsp. lactis (strain IL1403) OX=272623 GN=ybeA PE=3 SV=1
104	1	lactococcus	Q9CHQ3	87	22068	3	3	3	3	0.76	ATP-dependent Clp protease proteolytic subunit OS=Lactococcus lactis subsp. lactis (strain IL1403) OX=272623 GN=clpP PE=3 SV=1
79	1	lactococcus	Q9CHE0	136	75775	3	3	3	3	0.18	Methionine--tRNA ligase OS=Lactococcus lactis subsp. lactis (strain IL1403) OX=272623 GN=metG PE=3 SV=1
96	1	lactococcus	Q9CEG3	98	49975	3	3	3	3	0.29	Aminopeptidase C OS=Lactococcus lactis subsp. lactis (strain IL1403) OX=272623 GN=pepC PE=3 SV=3
92	1	lactococcus	Q9CH46	104	21574	3	3	3	3	0.79	Peptidyl-prolyl cis-trans isomerase OS=Lactococcus lactis subsp. lactis (strain IL1403) OX=272623 GN=ppiB PE=3 SV=1
66	1	lactococcus	Q9CDJ1	211	47025	6	6	3	3	0.31	Peptidase C51 domain-containing protein OS=Lactococcus lactis subsp. lactis (strain IL1403) OX=272623 GN=usp45 PE=4 SV=1
87	1	lactococcus	Q9CF32	116	40154	6	6	3	3	0.52	Proline dipeptidase OS=Lactococcus lactis subsp. lactis (strain IL1403) OX=272623 GN=pepQ PE=3 SV=1
90	1	lactococcus	Q9CID5	105	87351	5	5	3	3	0.16	Trehalose 6-phosphate phosphorylase OS=Lactococcus lactis subsp. lactis (strain IL1403) OX=272623 GN=trePP PE=1 SV=3
82	1	lactococcus	Q9CF37	131	11803	9	9	3	3	1.85	Thioredoxin OS=Lactococcus lactis subsp. lactis (strain IL1403) OX=272623 GN=trxA PE=3 SV=1
64	2	lactococcus	Q9CGM2	138	13660	3	3	2	2	0.83	Fe-S_biosyn domain-containing protein OS=Lactococcus lactis subsp. lactis (strain IL1403) OX=272623 GN=ykiE PE=4 SV=1

62	1	lactococcus	Q9CDM0	278	7492	10	10	2	2	1.95	Uncharacterized protein OS=Lactococcus lactis subsp. lactis (strain IL1403) OX=272623 GN=ywiE PE=4 SV=1
111	1	lactococcus	Q9CHF5	71	16362	2	2	2	2	0.66	Biotin carboxyl carrier protein of acetyl-CoA carboxylase OS=Lactococcus lactis subsp. lactis (strain IL1403) OX=272623 GN=accB PE=4 SV=1
78	1	lactococcus	Q9CFF4	139	36254	2	2	2	2	0.26	Phosphoribosylformylglycinamidine cyclo-ligase OS=Lactococcus lactis subsp. lactis (strain IL1403) OX=272623 GN=purM PE=3 SV=1
108	1	lactococcus	Q9CJD7	77	56282	2	2	2	2	0.16	Dihydrolipoamide acetyltransferase component of pyruvate dehydrogenase complex OS=Lactococcus lactis subsp. lactis (strain IL1403) OX=272623 GN=pdhC PE=3 SV=1
65	1	lactococcus	Q9CH02	212	33965	6	6	2	2	0.28	Thioredoxin reductase OS=Lactococcus lactis subsp. lactis (strain IL1403) OX=272623 GN=trxB1 PE=3 SV=1
118	1	lactococcus	P0DOB5	67	35829	2	2	2	2	0.26	ATP-dependent 6-phosphofructokinase OS=Lactococcus lactis subsp. lactis (strain IL1403) OX=272623 GN=pfkA PE=3 SV=1
93	1	lactococcus	Q9CHF9	104	8396	3	3	2	2	1.61	Acyl carrier protein OS=Lactococcus lactis subsp. lactis (strain IL1403) OX=272623 GN=acpP PE=3 SV=1
99	1	lactococcus	Q9CHG0	93	34868	4	4	2	2	0.43	3-oxoacyl-[acyl-carrier-protein] synthase 3 OS=Lactococcus lactis subsp. lactis (strain IL1403) OX=272623 GN=fabH PE=3 SV=1
86	1	lactococcus	Q9CEF7	120	27955	3	3	2	2	0.35	Pyrroline-5-carboxylate reductase OS=Lactococcus lactis subsp. lactis (strain IL1403) OX=272623 GN=proC PE=3 SV=1
115	1	lactococcus	Q9CH56	68	12509	2	2	2	2	0.93	FeS_assembly_P domain-containing protein OS=Lactococcus lactis subsp. lactis (strain IL1403) OX=272623 GN=yjaF PE=4 SV=1
91	1	lactococcus	Q9CG20	104	54168	4	4	2	2	0.26	Glutamate decarboxylase OS=Lactococcus lactis subsp. lactis (strain IL1403) OX=272623 GN=gadB PE=1 SV=1
106	1	lactococcus	Q9CHV0	86	34520	2	2	2	2	0.28	HPr kinase/phosphorylase OS=Lactococcus lactis subsp. lactis (strain IL1403) OX=272623 GN=hprK PE=3 SV=1
88	1	lactococcus	Q9CI02	114	26458	3	3	2	2	0.37	Enoyl-[acyl-carrier-protein] reductase [NADH] OS=Lactococcus lactis subsp. lactis (strain IL1403) OX=272623 GN=fabI PE=3 SV=1
122	1	lactococcus	Q9CHF6	64	42631	2	2	2	2	0.22	3-oxoacyl-[acyl-carrier-protein] synthase 2 OS=Lactococcus lactis subsp. lactis (strain IL1403) OX=272623 GN=fabF PE=3 SV=1
97	1	lactococcus	Q9CDT4	98	69254	2	2	2	2	0.13	Proline--tRNA ligase OS=Lactococcus lactis subsp. lactis (strain IL1403) OX=272623 GN=proS PE=3 SV=1
112	1	lactococcus	Q9CHZ8	70	28260	2	2	2	2	0.34	Phosphomannomutase OS=Lactococcus lactis subsp. lactis (strain IL1403) OX=272623 GN=yfgH PE=3 SV=1
109	1	lactococcus	Q9CDM9	75	18339	3	3	2	2	0.97	Single-stranded DNA-binding protein 2 OS=Lactococcus lactis subsp. lactis (strain IL1403) OX=272623 GN=ssb2 PE=3 SV=1
124	1	lactococcus	Q9CE12	60	62780	2	2	2	2	0.14	Arginine--tRNA ligase OS=Lactococcus lactis subsp. lactis (strain IL1403) OX=272623 GN=argS PE=3 SV=1
101	1	lactococcus	Q9CEH7	92	34931	3	3	2	2	0.27	Uncharacterized protein OS=Lactococcus lactis subsp. lactis (strain IL1403) OX=272623 GN=ytdB PE=4 SV=1
117	1	lactococcus	Q9CEI0	67	43185	2	2	2	2	0.21	Elongation factor Tu OS=Lactococcus lactis subsp. lactis (strain IL1403) OX=272623 GN=tuf PE=3 SV=1
105	1	lactococcus	Q9CED2	86	74241	4	4	2	2	0.12	Threonine--tRNA ligase OS=Lactococcus lactis subsp. lactis (strain IL1403) OX=272623 GN=thrS PE=3 SV=1
113	1	lactococcus	Q9CFV2	68	118138	2	2	2	2	0.07	Carbamoyl-phosphate synthase large chain OS=Lactococcus lactis subsp. lactis (strain IL1403) OX=272623 GN=carB PE=3 SV=1
102	1	lactococcus	Q9CG71	92	20199	3	3	2	2	0.51	Uncharacterized protein OS=Lactococcus lactis subsp. lactis (strain IL1403) OX=272623 GN=ymgG PE=3 SV=1
74	1	lactococcus	Q9CDR4	153	28521	5	5	1	1	0.34	30S ribosomal protein S2 OS=Lactococcus lactis subsp. lactis (strain IL1403) OX=272623 GN=rpsB PE=3 SV=1
84	1	lactococcus	Q9CGE4	124	13104	3	3	1	1	0.37	7,8-dihydroneopterin aldolase OS=Lactococcus lactis subsp. lactis (strain IL1403) OX=272623 GN=folB PE=3 SV=1
120	1	lactococcus	Q9CID4	65	30997	1	1	1	1	0.14	Sugar hydrolase OS=Lactococcus lactis subsp. lactis (strain IL1403) OX=272623 GN=yeeB PE=1 SV=1
107	1	lactococcus	Q9CH85	78	31120	1	1	1	1	0.14	Bifunctional protein FolD OS=Lactococcus lactis subsp. lactis (strain IL1403) OX=272623 GN=folD PE=3 SV=2
114	1	lactococcus	Q9CF59	68	39406	1	1	1	1	0.11	Aspartate-semialdehyde dehydrogenase OS=Lactococcus lactis subsp. lactis (strain IL1403) OX=272623 GN=asd PE=3 SV=1

98	1	lactococcus	Q9CFK0	95	14680	1	1	1	1	0.33	Ferric uptake regulator OS=Lactococcus lactis subsp. lactis (strain IL1403) OX=272623 GN=fur PE=3 SV=1
119	1	lactococcus	Q9CHS8	66	21270	2	2	1	1	0.22	Ribosome hibernation promoting factor OS=Lactococcus lactis subsp. lactis (strain IL1403) OX=272623 GN=ygdA PE=3 SV=1
100	1	lactococcus	Q9CJ14	93	7215	2	2	1	1	0.75	50S ribosomal protein L28 OS=Lactococcus lactis subsp. lactis (strain IL1403) OX=272623 GN=rpmB PE=3 SV=1
110	1	lactococcus	Q9CDZ8	72	41638	3	3	1	1	0.11	Pyridine nucleotide-disulfide oxidoreductase OS=Lactococcus lactis subsp. lactis (strain IL1403) OX=272623 GN=yvdG PE=4 SV=1
125	1	lactococcus	Q9CJ80	58	11102	1	1	1	1	0.45	Nucleoid-associated protein LL0120 OS=Lactococcus lactis subsp. lactis (strain IL1403) OX=272623 GN=ybcG PE=3 SV=1
116	1	lactococcus	Q9CJJ1	67	42238	1	1	1	1	0.10	Beta sliding clamp OS=Lactococcus lactis subsp. lactis (strain IL1403) OX=272623 GN=dnaN PE=3 SV=1
121	1	lactococcus	P0A493	65	4556	1	1	1	1	1.36	50S ribosomal protein L36 OS=Lactococcus lactis subsp. lactis (strain IL1403) OX=272623 GN=rpmJ PE=3 SV=1
126	1	lactococcus	Q9CIH6	56	18297	1	1	1	1	0.25	Uncharacterized protein OS=Lactococcus lactis subsp. lactis (strain IL1403) OX=272623 GN=ydiG PE=4 SV=1
127	1	lactococcus	Q9CIL9	56	20728	1	1	1	1	0.22	Alkyl hydroperoxide reductase C OS=Lactococcus lactis subsp. lactis (strain IL1403) OX=272623 GN=ahpC PE=3 SV=1
128	1	lactococcus	Q9CIP3	54	8399	1	1	1	1	0.62	PC4 domain-containing protein OS=Lactococcus lactis subsp. lactis (strain IL1403) OX=272623 GN=ydbC PE=1 SV=1
129	1	lactococcus	Q07744	53	71493	1	1	1	1	0.06	Neutral endopeptidase OS=Lactococcus lactis subsp. lactis (strain IL1403) OX=272623 GN=pepO PE=1 SV=3
130	1	lactococcus	Q9CF73	53	45490	1	1	1	1	0.10	Gamma-glutamyl phosphate reductase OS=Lactococcus lactis subsp. lactis (strain IL1403) OX=272623 GN=proA PE=3 SV=1
131	1	lactococcus	Q9CF77	52	19819	1	1	1	1	0.23	Pyrimidine operon regulatory protein OS=Lactococcus lactis subsp. lactis (strain IL1403) OX=272623 GN=pyrR PE=3 SV=1
132	1	lactococcus	Q9CJD5	50	41299	1	1	1	1	0.11	Pyruvate dehydrogenase E1 component subunit alpha OS=Lactococcus lactis subsp. lactis (strain IL1403) OX=272623 GN=pdhA PE=4 SV=1
133	1	lactococcus	Q9CIF0	50	11349	1	1	1	1	0.44	PTS system cellobiose-specific EIIB component OS=Lactococcus lactis subsp. lactis (strain IL1403) OX=272623 GN=ptcB PE=1 SV=1
134	1	lactococcus	Q9CE35	49	43110	1	1	1	1	0.10	Acetate kinase 1 OS=Lactococcus lactis subsp. lactis (strain IL1403) OX=272623 GN=ackA1 PE=3 SV=1
135	1	lactococcus	Q9CIN0	49	31201	1	1	1	1	0.14	ABC transporter permease protein OS=Lactococcus lactis subsp. lactis (strain IL1403) OX=272623 GN=ydcF PE=4 SV=1
136	1	lactococcus	Q9CHE9	48	8544	1	1	1	1	0.61	UPF0337 protein YhjA OS=Lactococcus lactis subsp. lactis (strain IL1403) OX=272623 GN=yhjA PE=3 SV=1
137	1	lactococcus	Q9CII7	46	56579	1	1	1	1	0.08	Lysine--tRNA ligase OS=Lactococcus lactis subsp. lactis (strain IL1403) OX=272623 GN=lysS PE=3 SV=1
138	1	lactococcus	Q9CJD8	45	49928	1	1	1	1	0.09	Dihydrolipoyl dehydrogenase OS=Lactococcus lactis subsp. lactis (strain IL1403) OX=272623 GN=pdhD PE=3 SV=1
139	1	lactococcus	Q9CDZ4	45	18525	1	1	1	1	0.25	N-acetyltransferase domain-containing protein OS=Lactococcus lactis subsp. lactis (strain IL1403) OX=272623 GN=yveC PE=4 SV=1
140	1	lactococcus	Q9CHV6	44	32101	1	1	1	1	0.14	Methionine aminopeptidase OS=Lactococcus lactis subsp. lactis (strain IL1403) OX=272623 GN=pepM PE=3 SV=1

Proteins identified in excised band - 70kDa

Family	Member	Database	Accession	Score	Mass	Num. of matches	Num. of significant matches	Num. of sequences	Num. of significant sequences	emPAI	Description
1	1	lactococcus	P0A3J0	17764	64947	359	359	97	97	2097.97	Chaperone protein DnaK OS=Lactococcus lactis subsp. lactis (strain IL1403) OX=272623 GN=dnaK PE=3 SV=1
4	1	lactococcus	Q9CJ82	2784	62635	63	63	34	34	13.11	Phosphoenolpyruvate-protein phosphotransferase OS=Lactococcus lactis subsp. lactis (strain IL1403) OX=272623 GN=ptsI PE=3 SV=1
7	1	lactococcus	Q9CIR6	119	61591	2	2	2	2	0.15	Ribonuclease J OS=Lactococcus lactis subsp. lactis (strain IL1403) OX=272623 GN=yciH PE=3 SV=1
9	1	lactococcus	Q9CH07	78	59576	3	3	2	2	0.15	Formate--tetrahydrofolate ligase OS=Lactococcus lactis subsp. lactis (strain IL1403) OX=272623 GN=fhs PE=3 SV=1
10	1	lactococcus	Q9CI15	57	57850	1	1	1	1	0.08	Glucose-6-phosphate 1-dehydrogenase OS=Lactococcus lactis subsp. lactis (strain IL1403) OX=272623 GN=zwf PE=3 SV=1
11	1	lactococcus	Q9CGT6	48	65742	1	1	1	1	0.06	Glutamine--fructose-6-phosphate aminotransferase [isomerizing] OS=Lactococcus lactis subsp. lactis (strain IL1403) OX=272623 GN=glmS PE=3 SV=2
12	1	lactococcus	Q9CI03	44	16843	1	1	1	1	0.28	3-hydroxyacyl-[acyl-carrier-protein] dehydratase FabZ OS=Lactococcus lactis subsp. lactis (strain IL1403) OX=272623 GN=fabZ1 PE=3 SV=1
13	1	lactococcus	Q9CHL5	37	71704	1	1	1	1	0.06	ABC transporter ATP-binding and permease protein OS=Lactococcus lactis subsp. lactis (strain IL1403) OX=272623 GN=yhcA PE=4 SV=1

Proteins identified in excised band - 45kDa

Family	Member	Database	Accession	Score	Mass	Num. of matches	Num. of significant matches	Num. of sequences	Num. of significant sequences	emPAI	Description
1	1	lactococcus	Q9CHA0	9497	44683	202	202	59	59	808.49	30S ribosomal protein S1 OS=Lactococcus lactis subsp. lactis (strain IL1403) OX=272623 GN=rpsA PE=4 SV=1
5	1	lactococcus	P58013	1970	46128	41	41	24	24	12.35	Arginine deiminase OS=Lactococcus lactis subsp. lactis (strain IL1403) OX=272623 GN=arcA PE=3 SV=1
6	1	lactococcus	Q9CED6	1144	41036	25	25	21	21	7.55	DUF4097 domain-containing protein OS=Lactococcus lactis subsp. lactis (strain IL1403) OX=272623 GN=ythC PE=4 SV=1
8	1	lactococcus	Q01462	100	35086	2	2	2	2	0.26	L-lactate dehydrogenase 1 OS=Lactococcus lactis subsp. lactis (strain IL1403) OX=272623 GN=ldh1 PE=3 SV=3
9	1	lactococcus	P81181	88	49564	1	1	1	1	0.09	Glucose-6-phosphate isomerase OS=Lactococcus lactis subsp. lactis (strain IL1403) OX=272623 GN=pgi PE=1 SV=3
12	1	lactococcus	Q9CDL9	78	49833	2	2	2	2	0.19	Glutamine synthetase OS=Lactococcus lactis subsp. lactis (strain IL1403) OX=272623 GN=glnA PE=3 SV=1
14	1	lactococcus	Q07637	45	54278	1	1	1	1	0.08	Pyruvate kinase OS=Lactococcus lactis subsp. lactis (strain IL1403) OX=272623 GN=pyk PE=3 SV=2
15	1	lactococcus	Q9CEG3	41	49997	1	1	1	1	0.09	Aminopeptidase C OS=Lactococcus lactis subsp. lactis (strain IL1403) OX=272623 GN=pepC PE=3 SV=3
16	1	lactococcus	Q9CEM7	35	45943	1	1	1	1	0.10	Peptidase T OS=Lactococcus lactis subsp. lactis (strain IL1403) OX=272623 GN=pepT PE=3 SV=1
17	1	lactococcus	Q9CIJ5	35	24924	1	1	1	1	0.19	RelA_SpoT domain-containing protein OS=Lactococcus lactis subsp. lactis (strain IL1403) OX=272623 GN=ydgI PE=4 SV=1
18	1	lactococcus	Q9CFA8	31	25604	1	1	1	1	0.17	Glucosamine-6-phosphate deaminase OS=Lactococcus lactis subsp. lactis (strain IL1403) OX=272623 GN=nagB PE=3 SV=1
19	1	lactococcus	Q9CH92	31	47817	1	1	1	1	0.09	Glutathione reductase OS=Lactococcus lactis subsp. lactis (strain IL1403) OX=272623 GN=gshR PE=3 SV=1
20	1	lactococcus	P0A3J0	31	64947	1	1	1	1	0.06	Chaperone protein DnaK OS=Lactococcus lactis subsp. lactis (strain IL1403) OX=272623 GN=dnaK PE=3 SV=1

Proteins identified in excised band - 35kDa											
Family	Member	Database	Accession	Score	Mass	Num. of matches	Num. of significant matches	Num. of sequences	Num. of significant sequences	emPAI	Description
2	1	lactococcus	Q01462	6556	35086	140	140	47	47	605.10	L-lactate dehydrogenase 1 OS=Lactococcus lactis subsp. lactis (strain IL1403) OX=272623 GN=ldh1 PE=3 SV=3
5	1	lactococcus	P0A4K2	332	40912	7	7	5	5	0.65	Cystathionine beta-lyase OS=Lactococcus lactis subsp. lactis (strain IL1403) OX=272623 GN=metC PE=3 SV=1
6	1	lactococcus	P0A3J0	138	64947	4	4	4	4	0.30	Chaperone protein DnaK OS=Lactococcus lactis subsp. lactis (strain IL1403) OX=272623 GN=dnaK PE=3 SV=1
8	1	lactococcus	Q9CE25	98	34056	2	2	2	2	0.26	Glucokinase OS=Lactococcus lactis subsp. lactis (strain IL1403) OX=272623 GN=glk PE=3 SV=1
11	1	lactococcus	Q9CE86	68	31927	1	1	1	1	0.14	33 kDa chaperonin OS=Lactococcus lactis subsp. lactis (strain IL1403) OX=272623 GN=hslO PE=3 SV=1
13	1	lactococcus	Q9CH02	55	33987	1	1	1	1	0.13	Thioredoxin reductase OS=Lactococcus lactis subsp. lactis (strain IL1403) OX=272623 GN=trxB1 PE=3 SV=1
15	1	lactococcus	Q9CF20	48	36278	1	1	1	1	0.12	Oxidoreductase OS=Lactococcus lactis subsp. lactis (strain IL1403) OX=272623 GN=yrbA PE=4 SV=1
16	1	lactococcus	Q9CHL5	40	71704	1	1	1	1	0.06	ABC transporter ATP-binding and permease protein OS=Lactococcus lactis subsp. lactis (strain IL1403) OX=272623 GN=yhcA PE=4 SV=1
17	1	lactococcus	Q9CF01	37	22727	1	1	1	1	0.20	Acetyltransferase OS=Lactococcus lactis subsp. lactis (strain IL1403) OX=272623 GN=maa PE=3 SV=1
18	1	lactococcus	Q9CJ56	36	12406	1	1	1	1	0.42	Uncharacterized protein OS=Lactococcus lactis subsp. lactis (strain IL1403) OX=272623 GN=ybeM PE=4 SV=1
20	1	lactococcus	Q9CF79	34	34707	1	1	1	1	0.13	Aspartate carbamoyltransferase OS=Lactococcus lactis subsp. lactis (strain IL1403) OX=272623 GN=pyrB PE=3 SV=1
21	1	lactococcus	Q9CH06	32	21014	1	1	1	1	0.21	NlpC/P60 domain-containing protein OS=Lactococcus lactis subsp. lactis (strain IL1403) OX=272623 GN=yjgB PE=3 SV=1

Proteins identified in excised band - 25kDa

Family	Member	Database	Accession	Score	Mass	Num. of matches	Num. of significant matches	Num. of sequences	Num. of significant sequences	emPAI	Description
3	1	lactococcus	Q9CF79	2420	126726	44	44	22	22	1.47	Aspartate carbamoyltransferase OS=Lactococcus lactis subsp. lactis (strain IL1403) OX=272623 GN=pyrB PE=4 SV=1
4	1	lactococcus	Q9CE25	1812	34056	33	33	15	15	45132	Glucokinase OS=Lactococcus lactis subsp. lactis (strain IL1403) OX=272623 GN=glk PE=3 SV=1
6	1	lactococcus	Q9CE66	794	36321	17	17	13	13	3.53	UDP-glucose 4-epimerase OS=Lactococcus lactis subsp. lactis (strain IL1403) OX=272623 GN=galE PE=3 SV=1
7	1	lactococcus	P0A3J0	321	64947	6	6	6	6	0.46	Chaperone protein DnaK OS=Lactococcus lactis subsp. lactis (strain IL1403) OX=272623 GN=dnaK PE=3 SV=1
8	1	lactococcus	Q9CFW8	225	53398	8	8	7	7	0.74	Transporter OS=Lactococcus lactis subsp. lactis (strain IL1403) OX=272623 GN=ycdH PE=4 SV=1
9	1	lactococcus	Q01462	220	35086	5	5	5	5	0.79	L-lactate dehydrogenase 1 OS=Lactococcus lactis subsp. lactis (strain IL1403) OX=272623 GN=ldh1 PE=3 SV=3
10	1	lactococcus	Q9CHG0	194	34879	6	6	5	5	0.79	3-oxoacyl-[acyl-carrier-protein] synthase 3 OS=Lactococcus lactis subsp. lactis (strain IL1403) OX=272623 GN=fabH PE=3 SV=1
11	1	lactococcus	Q9CHV0	189	34520	5	5	5	5	0.84	HPr kinase/phosphorylase OS=Lactococcus lactis subsp. lactis (strain IL1403) OX=272623 GN=hprK PE=3 SV=1
12	1	lactococcus	Q9CHV6	97	32178	2	2	2	2	0.30	Methionine aminopeptidase OS=Lactococcus lactis subsp. lactis (strain IL1403) OX=272623 GN=pepM PE=3 SV=1
19	1	lactococcus	Q9CEF4	46	24211	1	1	1	1	0.19	Protein serine/threonine phosphatase OS=Lactococcus lactis subsp. lactis (strain IL1403) OX=272623 GN=pppL PE=4 SV=1
20	1	lactococcus	Q9CJ56	37	12406	1	1	1	1	0.39	Uncharacterized protein OS=Lactococcus lactis subsp. lactis (strain IL1403) OX=272623 GN=ybeM PE=4 SV=1
21	1	lactococcus	Q9CHL5	35	71704	1	1	1	1	0.06	ABC transporter ATP-binding and permease protein OS=Lactococcus lactis subsp. lactis (strain IL1403) OX=272623 GN=yhcA PE=4 SV=1
22	1	lactococcus	Q9CHB8	35	35637	1	1	1	1	0.12	Ribose-phosphate pyrophosphokinase 1 OS=Lactococcus lactis subsp. lactis (strain IL1403) OX=272623 GN=prs1 PE=3 SV=1
23	1	lactococcus	P0DOB5	34	35841	1	1	1	1	0.12	ATP-dependent 6-phosphofructokinase OS=Lactococcus lactis subsp. lactis (strain IL1403) OX=272623 GN=pfkA PE=3 SV=1
24	1	lactococcus	Q9CH06	34	21014	1	1	1	1	0.21	NlpC/P60 domain-containing protein OS=Lactococcus lactis subsp. lactis (strain IL1403) OX=272623 GN=yjgB PE=3 SV=1
25	1	lactococcus	Q9CIU0	33	17708	1	1	1	1	0.26	S-ribosylhomocysteine lyase OS=Lactococcus lactis subsp. lactis (strain IL1403) OX=272623 GN=luxS PE=3 SV=1
26	1	lactococcus	Q9CJD6	30	35188	1	1	1	1	0.12	PDH E1 component beta subunit OS=Lactococcus lactis subsp. lactis (strain IL1403) OX=272623 GN=pdhB PE=4 SV=1
