# Supplementary material for: The strain-dependent cytostatic activity of Lactococcus lactis on CRC cell lines is mediated through the release of arginine deiminase
Source: Microb Cell Fact. 2024 Mar 14;23:82. doi: 10.1186/s12934-024-02345-w (PMC10938756; doi:10.1186/s12934-024-02345-w)
Supplement: Supplementary file 2 — Supplementary Material 2 [file 12934_2024_2345_MOESM2_ESM.pdf]

ShinyGO enrichment analysis						
Enrichment FDR	nGenes	Pathway Genes	Fold Enrichment	Pathway	URL	Genes
0.000159066770523956	6	11	9.48668280871671	Chaperone, and ClpP, Ser active site		L0221 L0273 L178206 L198515 L198893 L72391
5.44280876884372e-05	8	11	8.04930662557781	Glycolysis		L0002 L0003 L0005 L0007 L0009 L0010 L0011 L0012
5.44280876884372e-05	8	11	8.04930662557781	Polysaccharide binding	http://amigo.geneontology.org/amigo/term/GO:0030247	L0003 L0010 L0011 L0273 L0349 L0376 L178206 L198893
5.44280876884372e-05	8	11	8.04930662557781	Mannan binding	http://amigo.geneontology.org/amigo/term/GO:2001065	L0003 L0010 L0011 L0273 L0349 L0376 L178206 L198893
4.34137340339832e-05	9	13	7.66232073011734	Cell surface	http://amigo.geneontology.org/amigo/term/GO:0009986	L0003 L0007 L0010 L0011 L0273 L0349 L0376 L178206 L198893
9.67267806720848e-05	8	15	7.37853107344633	Carbohydrate binding	http://amigo.geneontology.org/amigo/term/GO:0030246	L0003 L0010 L0011 L0273 L0349 L0376 L178206 L198893
0.154266136151785	2	12	7.37853107344633	Bacterial secretion system, and Ribonuclease P		L0206 L0336
0.154266136151785	2	4	7.37853107344633	Gluconeogenesis	http://amigo.geneontology.org/amigo/term/GO:0006094	L0011 L0012
0.154266136151785	2	4	7.37853107344633	Hexose biosynthetic process	http://amigo.geneontology.org/amigo/term/GO:0019319	L0011 L0012
0.154266136151785	2	3	7.37853107344633	NAD metabolic process	http://amigo.geneontology.org/amigo/term/GO:0019674	L0002 L0003
0.154266136151785	2	4	7.37853107344633	Monosaccharide biosynthetic process	http://amigo.geneontology.org/amigo/term/GO:0046364	L0011 L0012
0.154266136151785	2	4	7.37853107344633	Oxidoreductase, N-terminal	https://www.ebi.ac.uk/interpro/entry/InterPro/IPR000683	L109882 L63684
0.154266136151785	2	4	7.37853107344633	Oxidoreductase family, NAD-binding Rossmann fold	http://pfam.xfam.org/family/PF01408	L109882 L63684
0.000285571485144497	7	12	7.04314329738059	Pyruvate metabolic process	http://amigo.geneontology.org/amigo/term/GO:0006090	L0002 L0003 L0007 L0010 L0011 L0012 L0017
0.000285571485144497	7	12	7.04314329738059	Glycolytic process	http://amigo.geneontology.org/amigo/term/GO:0006096	L0002 L0003 L0007 L0010 L0011 L0012 L0017
0.000285571485144497	7	12	7.04314329738059	Nucleoside diphosphate phosphorylation	http://amigo.geneontology.org/amigo/term/GO:0006165	L0002 L0003 L0007 L0010 L0011 L0012 L0017

ShinyGO enrichment analysis						
Enrichment FDR	nGenes	Pathway Genes	Fold Enrichment	Pathway	URL	Genes
0.000285571485144497	7	12	7.04314329738059	ATP generation from ADP	http://amigo.geneontology.org/amigo/term/GO:0006757	L0002 L0003 L0007 L0010 L0011 L0012 L0017
0.000285571485144497	7	12	7.04314329738059	ADP metabolic process	http://amigo.geneontology.org/amigo/term/GO:0046031	L0002 L0003 L0007 L0010 L0011 L0012 L0017
0.000285571485144497	7	12	7.04314329738059	Nucleotide phosphorylation	http://amigo.geneontology.org/amigo/term/GO:0046939	L0002 L0003 L0007 L0010 L0011 L0012 L0017
0.0605059732728799	3	5	6.6406779661017	Glyceraldehyde-3-phosphate dehydrogenase, type I, and Phosphoglycerate kinase		L0005 L0009 L0010
0.0605059732728799	3	6	6.6406779661017	Glucose metabolic process	http://amigo.geneontology.org/amigo/term/GO:0006006	L0011 L0012 L57408
0.0605059732728799	3	7	6.6406779661017	Allosteric enzyme		L0002 L0003 L0017
0.00162869210965844	6	15	6.6406779661017	Chaperone		L0221 L0273 L0336 L178206 L198515 L198893
0.000560624840004574	7	13	6.45621468926554	Purine nucleoside diphosphate metabolic process	http://amigo.geneontology.org/amigo/term/GO:0009135	L0002 L0003 L0007 L0010 L0011 L0012 L0017
0.000560624840004574	7	13	6.45621468926554	Purine ribonucleoside diphosphate metabolic process	http://amigo.geneontology.org/amigo/term/GO:0009179	L0002 L0003 L0007 L0010 L0011 L0012 L0017
0.000560624840004574	7	13	6.45621468926554	Ribonucleoside diphosphate metabolic process	http://amigo.geneontology.org/amigo/term/GO:0009185	L0002 L0003 L0007 L0010 L0011 L0012 L0017
0.021555780038479	4	9	6.32445520581114	Protein binding	http://amigo.geneontology.org/amigo/term/GO:0005515	L0136 L0273 L178206 L198893
0.000124123405660691	9	24	6.22563559322034	Monocarboxylic acid metabolic process	http://amigo.geneontology.org/amigo/term/GO:0032787	L0002 L0003 L0007 L0010 L0011 L0012 L0017 L0046 L0183
0.00104544658174168	7	14	5.95958279009127	Nucleoside diphosphate metabolic process	http://amigo.geneontology.org/amigo/term/GO:0009132	L0002 L0003 L0007 L0010 L0011 L0012 L0017
0.0931138996574932	3	8	5.53389830508475	Hexose metabolic process	http://amigo.geneontology.org/amigo/term/GO:0019318	L0011 L0012 L57408
0.0931138996574932	3	10	5.53389830508475	Stress response		L0221 L0273 L178206
0.000256536977505337	9	20	5.53389830508475	Glycolysis, and Pentose phosphate pathway		L0002 L0003 L0005 L0007 L0009 L0010 L0011 L0012 L0046

ShinyGO enrichment analysis						
**Enrichment FDR**	**nGenes**	**Pathway Genes**	**Fold Enrichment**	**Pathway**	**URL**	**Genes**
0.00285801836692017	7	18	5.16497175141243	ATP metabolic process	http://amigo.geneontology.org/amigo/term/GO:0046034	L0002 L0003 L0007 L0010 L0011 L0012 L0017
0.00151993207027303	8	21	4.91902071563088	Generation of precursor metabolites and energy	http://amigo.geneontology.org/amigo/term/GO:0006091	L0002 L0003 L0007 L0010 L0011 L0012 L0017 L0046
0.140079211881442	3	8	4.74334140435835	Taurine and hypotaurine metabolism, and Naphthalene degradation		L0017 L107797 L57408
0.00597034094300643	7	21	4.55732801595214	Carbohydrate catabolic process	http://amigo.geneontology.org/amigo/term/GO:0016052	L0002 L0003 L0007 L0010 L0011 L0012 L0017
0.0742282997372097	4	24	4.4271186440678	Glutamine family amino acid metabolic process	http://amigo.geneontology.org/amigo/term/GO:0009064	L0118 L0329 L115968 L198033
0.186957212055042	3	14	4.15042372881356	Monosaccharide metabolic process	http://amigo.geneontology.org/amigo/term/GO:0005996	L0011 L0012 L57408
0.0211401213440054	6	26	4.15042372881356	Mixed, incl. chaperone, and peptidase m16, c-terminal		L0221 L0273 L178206 L198515 L198893 L72391
0.0969988082044449	4	11	4.02465331278891	tRNA aminoacylation		L0349 L0351 L0353 L0356
0.0560367340406461	5	23	3.95278450363196	Ligase activity, forming carbon-nitrogen bonds	http://amigo.geneontology.org/amigo/term/GO:0016879	L0118 L115968 L12179 L159505 L198033
0.000159066770523956	14	62	3.7792476229847	Carbon metabolism, and Valine, leucine and isoleucine biosynthesis		L0002 L0003 L0005 L0007 L0009 L0010 L0011 L0012 L0017 L0034 L0035 L0046 L107797 L57408
0.128771048327233	4	30	3.68926553672316	Cell cycle		L0206 L0336 L102051 L117685
0.128771048327233	4	30	3.68926553672316	Cell division		L0206 L0336 L102051 L117685
0.000126006105010435	16	78	3.47224991691592	Microbial metabolism in diverse environments, and Valine, leucine and isoleucine biosynthesis		L0002 L0003 L0005 L0007 L0009 L0010 L0011 L0012 L0017 L0034 L0035 L0046 L0118 L0243 L107797 L57408
0.00823142269647618	9	37	3.43483343074226	Purine ribonucleotide metabolic process	http://amigo.geneontology.org/amigo/term/GO:0009150	L0002 L0003 L0007 L0010 L0011 L0012 L0017 L115968 L12179
0.00985739236130607	9	40	3.32033898305085	Catabolic process	http://amigo.geneontology.org/amigo/term/GO:0009056	L0002 L0003 L0007 L0010 L0011 L0012 L0017 L0255 L0329
0.00985739236130607	9	40	3.32033898305085	Organic substance catabolic process	http://amigo.geneontology.org/amigo/term/GO:1901575	L0002 L0003 L0007 L0010 L0011 L0012 L0017 L0255 L0329

ShinyGO enrichment analysis						
Enrichment FDR	nGenes	Pathway Genes	Fold Enrichment	Pathway	URL	Genes
0.0124216890703823	9	39	3.21323127392017	Purine nucleotide metabolic process	http://amigo.geneontology.org/amigo/term/GO:0006163	L0002 L0003 L0007 L0010 L0011 L0012 L0017 L115968 L12179
0.00474759624332992	11	50	3.20383586083854	Ribonucleotide metabolic process	http://amigo.geneontology.org/amigo/term/GO:0009259	L0002 L0003 L0007 L0010 L0011 L0012 L0017 L115968 L12179 L182555 L198033
0.196473195194474	4	23	3.16222760290557	GTP-binding		L0206 L0243 L0371 L12179
0.0157637129671979	9	40	3.11281779661017	Purine-containing compound metabolic process	http://amigo.geneontology.org/amigo/term/GO:0072521	L0002 L0003 L0007 L0010 L0011 L0012 L0017 L115968 L12179
9.67267806720848e-05	19	122	3.09247258225324	Carboxylic acid metabolic process	http://amigo.geneontology.org/amigo/term/GO:0019752	L0002 L0003 L0007 L0010 L0011 L0012 L0017 L0046 L0118 L0181 L0183 L0329 L0349 L0351 L0353 L0356 L115968 L159505 L198033
9.67267806720848e-05	19	122	3.09247258225324	Oxoacid metabolic process	http://amigo.geneontology.org/amigo/term/GO:0043436	L0002 L0003 L0007 L0010 L0011 L0012 L0017 L0046 L0118 L0181 L0183 L0329 L0349 L0351 L0353 L0356 L115968 L159505 L198033
0.0194235876188599	9	50	3.01848998459168	Carbohydrate metabolic process	http://amigo.geneontology.org/amigo/term/GO:0005975	L0002 L0003 L0007 L0010 L0011 L0012 L0017 L0046 L57408
0.00790234736310965	11	53	2.96940884663084	Ribose phosphate metabolic process	http://amigo.geneontology.org/amigo/term/GO:0019693	L0002 L0003 L0007 L0010 L0011 L0012 L0017 L115968 L12179 L182555 L198033
0.000149145683054915	19	126	2.96180472666508	Organic acid metabolic process	http://amigo.geneontology.org/amigo/term/GO:0006082	L0002 L0003 L0007 L0010 L0011 L0012 L0017 L0046 L0118 L0181 L0183 L0329 L0349 L0351 L0353 L0356 L115968 L159505 L198033
0.0540686552722721	8	45	2.76694915254237	Phosphorylation	http://amigo.geneontology.org/amigo/term/GO:0016310	L0002 L0003 L0007 L0010 L0011 L0012 L0017 L120628
0.0704161453804529	9	54	2.4295163290616	Ligase activity	http://amigo.geneontology.org/amigo/term/GO:0016874	L0118 L0349 L0351 L0353 L0356 L115968 L12179 L159505 L198033
0.000189102868625675	24	191	2.41479198767334	Biosynthesis of secondary metabolites, and nucleotide biosynthetic process		L0002 L0003 L0005 L0007 L0009 L0010 L0011 L0012 L0017 L0034 L0035 L0046 L0118 L0181 L0243 L107797 L115968 L12179 L159505 L182555 L198033 L57408 L65029 L88187
0.00206376276500419	19	149	2.36278804037326	Purine nucleotide binding	http://amigo.geneontology.org/amigo/term/GO:0017076	L0002 L0003 L0010 L0118 L0221 L0243 L0273 L0349 L0351 L0353 L0356 L0371 L115968 L12179 L159505 L178206 L198033 L198515 L198893
0.0461998993894072	11	67	2.34126466753585	Nucleoside phosphate metabolic process	http://amigo.geneontology.org/amigo/term/GO:0006753	L0002 L0003 L0007 L0010 L0011 L0012 L0017 L115968 L12179 L182555 L198033
0.0461998993894072	11	67	2.34126466753585	Nucleotide metabolic process	http://amigo.geneontology.org/amigo/term/GO:0009117	L0002 L0003 L0007 L0010 L0011 L0012 L0017 L115968 L12179 L182555 L198033

ShinyGO enrichment analysis						
Enrichment FDR	nGenes	Pathway Genes	Fold Enrichment	Pathway	URL	Genes
4.34137340339832e-05	32	255	2.28496446145435	Cytoplasm		L0002 L0007 L0010 L0012 L0017 L0118 L0181 L0183 L0206 L0243 L0255 L0273 L0275 L0329 L0336 L0349 L0351 L0353 L0356 L0371 L0376 L102051 L117685 L120628 L12179 L135991 L182555 L198515 L198893 L57408 L72391 L89418
0.175345554458294	7	61	2.27866400797607	NAD(P)-binding domain superfamily	https://www.ebi.ac.uk/interpro/entry/InterPro/IPR036291	L0005 L0017 L0046 L109882 L135991 L161132 L63684
0.00522438988391645	18	148	2.26386748844376	Purine ribonucleotide binding	http://amigo.geneontology.org/amigo/term/GO:0032555	L0002 L0003 L0010 L0118 L0221 L0243 L0349 L0351 L0353 L0356 L0371 L115968 L12179 L159505 L178206 L198033 L198515 L198893
0.00522438988391645	18	148	2.26386748844376	Purine ribonucleoside triphosphate binding	http://amigo.geneontology.org/amigo/term/GO:0035639	L0002 L0003 L0010 L0118 L0221 L0243 L0349 L0351 L0353 L0356 L0371 L115968 L12179 L159505 L178206 L198033 L198515 L198893
0.0103385965382394	16	135	2.24157906028749	Adenyl nucleotide binding	http://amigo.geneontology.org/amigo/term/GO:0030554	L0002 L0003 L0010 L0118 L0221 L0273 L0349 L0351 L0353 L0356 L115968 L159505 L178206 L198033 L198515 L198893
0.0480529310315577	12	81	2.2135593220339	Organophosphate metabolic process	http://amigo.geneontology.org/amigo/term/GO:0019637	L0002 L0003 L0007 L0010 L0011 L0012 L0017 L0046 L115968 L12179 L182555 L198033
0.00627788564940447	18	152	2.2135593220339	Ribonucleotide binding	http://amigo.geneontology.org/amigo/term/GO:0032553	L0002 L0003 L0010 L0118 L0221 L0243 L0349 L0351 L0353 L0356 L0371 L115968 L12179 L159505 L178206 L198033 L198515 L198893
0.0540686552722721	12	88	2.17727146429564	Carbohydrate derivative metabolic process	http://amigo.geneontology.org/amigo/term/GO:1901135	L0002 L0003 L0007 L0010 L0011 L0012 L0017 L0046 L115968 L12179 L182555 L198033
0.125901168867438	9	63	2.16543846720707	Ligase		L0118 L0349 L0351 L0353 L0356 L115968 L12179 L159505 L198033
0.00790234736310965	18	155	2.16543846720707	Carbohydrate derivative binding	http://amigo.geneontology.org/amigo/term/GO:0097367	L0002 L0003 L0010 L0118 L0221 L0243 L0349 L0351 L0353 L0356 L0371 L115968 L12179 L159505 L178206 L198033 L198515 L198893
0.0241287366845417	15	134	2.12842242503259	ATP binding	http://amigo.geneontology.org/amigo/term/GO:0005524	L0002 L0003 L0010 L0118 L0221 L0349 L0351 L0353 L0356 L115968 L159505 L178206 L198033 L198515 L198893
0.0241287366845417	15	134	2.12842242503259	Adenyl ribonucleotide binding	http://amigo.geneontology.org/amigo/term/GO:0032559	L0002 L0003 L0010 L0118 L0221 L0349 L0351 L0353 L0356 L115968 L159505 L178206 L198033 L198515 L198893
0.00575554220372263	20	175	2.10815173527038	Nucleotide binding	http://amigo.geneontology.org/amigo/term/GO:0000166	L0002 L0003 L0010 L0046 L0118 L0221 L0243 L0273 L0349 L0351 L0353 L0356 L0371 L115968 L12179 L159505 L178206 L198033 L198515 L198893
0.00575554220372263	20	175	2.10815173527038	Nucleoside phosphate binding	http://amigo.geneontology.org/amigo/term/GO:1901265	L0002 L0003 L0010 L0046 L0118 L0221 L0243 L0273 L0349 L0351 L0353 L0356 L0371 L115968 L12179 L159505 L178206 L198033 L198515 L198893
0.0867524082210249	11	75	2.09906487434249	Nucleobase-containing small molecule metabolic process	http://amigo.geneontology.org/amigo/term/GO:0055086	L0002 L0003 L0007 L0010 L0011 L0012 L0017 L115968 L12179 L182555 L198033

ShinyGO enrichment analysis						
Enrichment FDR	nGenes	Pathway Genes	Fold Enrichment	Pathway	URL	Genes
0.00842345847682965	19	172	2.08206074844773	Anion binding	http://amigo.geneontology.org/amigo/term/GO:0043168	L0002 L0003 L0010 L0118 L0181 L0221 L0243 L0349 L0351 L0353 L0356 L0371 L115968 L12179 L159505 L178206 L198033 L198515 L198893
0.0605059732728799	13	101	2.05544794188862	Phosphate-containing compound metabolic process	http://amigo.geneontology.org/amigo/term/GO:0006796	L0002 L0003 L0007 L0010 L0011 L0012 L0017 L0046 L115968 L120628 L12179 L182555 L198033
0.00603684990939932	21	191	2.03880463871543	Small molecule binding	http://amigo.geneontology.org/amigo/term/GO:0036094	L0002 L0003 L0010 L0046 L0118 L0181 L0221 L0243 L0273 L0349 L0351 L0353 L0356 L0371 L115968 L12179 L159505 L178206 L198033 L198515 L198893
0.165644206668034	9	93	2.03286060186787	Cellular amino acid metabolic process	http://amigo.geneontology.org/amigo/term/GO:0006520	L0118 L0181 L0329 L0349 L0351 L0353 L0356 L115968 L198033
0.0657863567559312	13	103	2.0264979708761	Phosphorus metabolic process	http://amigo.geneontology.org/amigo/term/GO:0006793	L0002 L0003 L0007 L0010 L0011 L0012 L0017 L0046 L115968 L120628 L12179 L182555 L198033
0.0721387512397699	2	4	11.0677966101695	Protein dimerization activity	http://amigo.geneontology.org/amigo/term/GO:0046983	L0136 L0273
0.0721387512397699	2	3	11.0677966101695	Pyruvate/Phosphoenolpyruvate kinase-like domain superfamily	https://www.ebi.ac.uk/interpro/entry/InterPro/IPR015813	L0003 L120628
0.0721387512397699	2	3	11.0677966101695	Pyruvate kinase-like domain superfamily	https://www.ebi.ac.uk/interpro/entry/InterPro/IPR040442	L0003 L120628
0.00151993207027303	4	5	11.0677966101695	Protein folding		L0273 L178206 L198515 L198893
0.00824275899175663	22	203	1.93247242399785	Small molecule metabolic process	http://amigo.geneontology.org/amigo/term/GO:0044281	L0002 L0003 L0007 L0010 L0011 L0012 L0017 L0046 L0118 L0181 L0183 L0329 L0349 L0351 L0353 L0356 L115968 L12179 L159505 L182555 L198033 L57408
0.0472846362879087	19	260	1.78210284401034	Nucleotide-binding		L0002 L0003 L0005 L0010 L0118 L0206 L0221 L0243 L0349 L0351 L0353 L0356 L0371 L115968 L12179 L159505 L178206 L198033 L198893
0.0231711026362954	22	237	1.77731040455277	Ion binding	http://amigo.geneontology.org/amigo/term/GO:0043167	L0002 L0003 L0007 L0010 L0118 L0181 L0221 L0243 L0349 L0351 L0353 L0356 L0371 L115968 L120628 L12179 L159505 L178206 L198033 L198515 L198893 L65029
0.0668239019019564	18	179	1.74754683318466	Nucleobase-containing compound metabolic process	http://amigo.geneontology.org/amigo/term/GO:0006139	L0002 L0003 L0007 L0010 L0011 L0012 L0017 L0136 L0255 L0275 L0349 L0351 L0353 L0356 L115968 L12179 L182555 L198033
0.0668239019019564	19	209	1.70965963896927	Heterocycle metabolic process	http://amigo.geneontology.org/amigo/term/GO:0046483	L0002 L0003 L0007 L0010 L0011 L0012 L0017 L0136 L0255 L0275 L0349 L0351 L0353 L0356 L115968 L12179 L159505 L182555 L198033
0.00455078862582952	31	347	1.70697360654355	Cellular anatomical entity	http://amigo.geneontology.org/amigo/term/GO:0110165	L0002 L0003 L0007 L0010 L0011 L0012 L0017 L0118 L0181 L0183 L0243 L0255 L0273 L0275 L0329 L0336 L0349 L0351 L0353 L0356 L0371 L0376 L0405 L120628 L12179 L178206 L182555 L198515 L198893 L57408 L72391

ShinyGO enrichment analysis						
Enrichment FDR	nGenes	Pathway Genes	Fold Enrichment	Pathway	URL	Genes
0.082114586914438	19	215	1.66895345708905	Cellular aromatic compound metabolic process	http://amigo.geneontology.org/amigo/term/GO:0006725	L0002 L0003 L0007 L0010 L0011 L0012 L0017 L0136 L0255 L0275 L0349 L0351 L0353 L0356 L115968 L12179 L159505 L182555 L198033
0.0877949002561945	19	217	1.6558120912852	Organic cyclic compound metabolic process	http://amigo.geneontology.org/amigo/term/GO:1901360	L0002 L0003 L0007 L0010 L0011 L0012 L0017 L0136 L0255 L0275 L0349 L0351 L0353 L0356 L115968 L12179 L159505 L182555 L198033
0.00960298366740877	30	367	1.64373216982715	Catalytic activity	http://amigo.geneontology.org/amigo/term/GO:0003824	L0002 L0003 L0007 L0010 L0011 L0012 L0017 L0046 L0118 L0136 L0181 L0255 L0275 L0329 L0336 L0349 L0351 L0353 L0356 L0371 L115968 L120628 L12179 L130687 L159505 L182555 L198033 L57408 L65029 L72391
0.0161980911498783	28	284	1.63967357187696	Intracellular	http://amigo.geneontology.org/amigo/term/GO:0005622	L0002 L0007 L0010 L0012 L0017 L0118 L0181 L0183 L0243 L0255 L0273 L0275 L0329 L0336 L0349 L0351 L0353 L0356 L0371 L0376 L0405 L120628 L12179 L182555 L198515 L198893 L57408 L72391
0.0381796153347889	29	340	1.52841000807103	Binding	http://amigo.geneontology.org/amigo/term/GO:0005488	L0002 L0003 L0007 L0010 L0011 L0046 L0118 L0136 L0181 L0183 L0221 L0243 L0273 L0275 L0349 L0351 L0353 L0356 L0371 L0376 L115968 L120628 L12179 L159505 L178206 L198033 L198515 L198893 L65029
0.0895273037046281	24	280	1.51786924939467	Organic cyclic compound binding	http://amigo.geneontology.org/amigo/term/GO:0097159	L0002 L0003 L0010 L0046 L0118 L0136 L0181 L0221 L0243 L0273 L0275 L0349 L0351 L0353 L0356 L0371 L0376 L115968 L12179 L159505 L178206 L198033 L198515 L198893
0.0895273037046281	24	280	1.51786924939467	Heterocyclic compound binding	http://amigo.geneontology.org/amigo/term/GO:1901363	L0002 L0003 L0010 L0046 L0118 L0136 L0181 L0221 L0243 L0273 L0275 L0349 L0351 L0353 L0356 L0371 L0376 L115968 L12179 L159505 L178206 L198033 L198515 L198893
0.09343880771202	25	286	1.48760707125934	Organonitrogen compound metabolic process	http://amigo.geneontology.org/amigo/term/GO:1901564	L0002 L0003 L0007 L0010 L0011 L0012 L0017 L0118 L0181 L0329 L0336 L0349 L0351 L0353 L0356 L0371 L0376 L0405 L115968 L12179 L130687 L159505 L182555 L198033 L72391
0.0265529694821981	34	455	1.46422211963332	Cellular process	http://amigo.geneontology.org/amigo/term/GO:0009987	L0002 L0003 L0007 L0010 L0011 L0012 L0017 L0046 L0118 L0136 L0181 L0183 L0255 L0273 L0275 L0329 L0336 L0349 L0351 L0353 L0356 L0371 L0376 L0405 L115968 L120628 L12179 L159505 L178206 L182555 L198033 L198515 L198893 L65029
0.0910112343737748	30	387	1.4069232979029	Primary metabolic process	http://amigo.geneontology.org/amigo/term/GO:0044238	L0002 L0003 L0007 L0010 L0011 L0012 L0017 L0046 L0118 L0136 L0181 L0183 L0255 L0275 L0329 L0336 L0349 L0351 L0353 L0356 L0371 L0376 L0405 L115968 L12179 L130687 L182555 L198033 L57408 L72391
0.145843767550386	28	369	1.38347457627119	Nitrogen compound metabolic process	http://amigo.geneontology.org/amigo/term/GO:0006807	L0002 L0003 L0007 L0010 L0011 L0012 L0017 L0118 L0136 L0181 L0255 L0275 L0329 L0336 L0349 L0351 L0353 L0356 L0371 L0376 L0405 L115968 L12179 L130687 L159505 L182555 L198033 L72391

ShinyGO enrichment analysis						
Enrichment FDR	nGenes	Pathway Genes	Fold Enrichment	Pathway	URL	Genes
0.108170462112439	31	417	1.37240677966102	Organic substance metabolic process	http://amigo.geneontology.org/amigo/term/GO:0071704	L0002 L0003 L0007 L0010 L0011 L0012 L0017 L0046 L0118 L0136 L0181 L0183 L0255 L0275 L0329 L0336 L0349 L0351 L0353 L0356 L0371 L0376 L0405 L115968 L12179 L130687 L159505 L182555 L198033 L57408 L72391
0.0983272834895938	32	430	1.36744977422944	Metabolic process	http://amigo.geneontology.org/amigo/term/GO:0008152	L0002 L0003 L0007 L0010 L0011 L0012 L0017 L0046 L0118 L0136 L0181 L0183 L0255 L0275 L0329 L0336 L0349 L0351 L0353 L0356 L0371 L0376 L0405 L115968 L120628 L12179 L130687 L159505 L182555 L198033 L57408 L72391
0.195661790305984	29	403	1.33180955060131	Cellular metabolic process	http://amigo.geneontology.org/amigo/term/GO:0044237	L0002 L0003 L0007 L0010 L0011 L0012 L0017 L0046 L0118 L0136 L0181 L0183 L0255 L0275 L0329 L0336 L0349 L0351 L0353 L0356 L0371 L0376 L0405 L115968 L120628 L12179 L159505 L182555 L198033
