# Supplementary material for: The strain-dependent cytostatic activity of Lactococcus lactis on CRC cell lines is mediated through the release of arginine deiminase
Source: Microb Cell Fact. 2024 Mar 14;23:82. doi: 10.1186/s12934-024-02345-w (PMC10938756; doi:10.1186/s12934-024-02345-w)
Supplement: Supplementary file 3 — Supplementary Material 3 [file 12934_2024_2345_MOESM3_ESM.pdf]

Arginine deiminase sequences	
ArcA nucleotide sequence	atgaacaatggaattaatgttaactcagaaattgggaaattaaaatcagtcctcctccaccgcccaggtgcggaagttgaaaatattaccccagacacaatgaaacaacttttatttgatgatattcca<br>tatctcaaaattgctcaaaaagagcatgatttctttgctcaaactttgcgtgataatggtgcagaaactgtttatatcgaaaatcttgcaacagaagtttttgaaaaatcatctgaaacaaaagaagaatt<br>cttaagtcacttattacacgaagcgggttaccgtccgggacgcacatatgatggtttgactgaatatttgacttcaatgtcaacaaaagatatggtcgaaaaaatctatgctggtgttcgtaaaaatgaat<br>tggatatcaaacgtacagcacttagcgatatggcaggatctgatgcagaaaattatttctacctcaacccattaccaaatgcctacttcacacgtgacccacaagcttcaatgggtgtcggaatgacc<br>attaataaaatgaccttcccagctcgtcaacctgaaagcttgattacagagtatgttatggctaatcacccacgtttcaaagatactccaatctggcgtgatcgtaaccataccactcgtatcgaaggtg<br>gtgatgaattaattcttaacaaaacaacagtagctatcggggtttcagaacgtacttcatctaaaacaattcaaaatcttgctaaagagttatttgcaaacccactttcaacatttgacactgtacttgcgg<br>ttgaaattcctcataaccatgcaatgatgcatttggatacagtatttacaatgattaaccatgatcaatttactgtcttcccaggaattatggatggtgctggtaatatcaatgtcttcattcttcgccctggtaa<br>agatgacgaagttgaaattgaacatttgactgaccttaaagcagcactcaagaaagtcttgaatctttcagaacttgacttaattgaatgtggtgcaggtgatccaattgccgctcctcgcgaacaatg<br>gaatgatggatcaaatacacttgctatcgcacccggtgaaatcgttacttacgaccgtaactatgtaactgttgagttattgaaagagcatggaatcaaagttcatgaaattctttcaagcgaacttggtc<br>gtggccgtggtggtgcccgttgtatgtcacaaccactttggcgtgaagatttgtaa
**ADI protein sequence**	MNNGINVNSEIGKLKSVLLHRPGAEVENITPDTMKQLLFDDIPYLKIAQKEHDFFAQTLRDNGAETVYIENLATEVFEKSSETKEEFLSHLLHE<br>AGYRPGRTYDGLTEYLTSMSTKDMVEKIYAGVRKNELDIKRTALSDMAGSDAENYFYLNPLPNAYFTRDPQASMGVGMTINKMTFPARQP<br>ESLITEYVMANHPRFKDTPIWRDRNHTTRIEGGDELILNKTTVAIGVSERTSSKTIQNLAKELFANPLSTFDTVLAVEIPHNHAMMHLDTVFTMI<br>NHDQFTVFPGIMDGAGNINVFILRPGKDDEVEIEHLTDLKAALKKVLNLSELDLIECGAGDPIAAPREQWNDGSNTLAIAPGEIVTYDRNYVTV<br>ELLKEHGIKVHEILSSELGRGRGGARCMSQPLWREDL
ArtGene ArcA optimized sequence	GGATCCATGAATAATGGCATTAACGTGAATAGCGAAATCGGCAAACTGAAAAGCGTTCTGCTGCATCGTCCGGGTGCAGA<br>AGTTGAAAACATTACACCGGATACCATGAAACAGCTGCTGTTCGATGATATTCCGTATCTGAAAATTGCCCAGAAAGAGC<br>ATGATTTCTTTGCACAGACCCTGCGTGATAATGGTGCAGAAACCGTTTATATTGAAAATCTGGCCACCGAAGTGTTTGAG<br>AAAAGCAGCGAAACCAAAGAAGAATTTCTGAGCCATCTGCTGCACGAAGCAGGTTATCGTCCTGGTCGTACCTATGATGG<br>TCTGACCGAATATCTGACCAGCATGAGCACCAAAGATATGGTGGAAAAGATTTATGCCGGTGTGCGTAAAAACGAGCTGG<br>ATATTAAACGTACCGCACTGAGCGATATGGCAGGTAGTGATGCAGAAAATTACTTTTATCTGAATCCGCTGCCGAACGCA<br>TATTTTACCCGTGATCCGCAGGCAAGCATGGGTGTTGGTATGACCATTAACAAAATGACCTTTCCGGCACGTCAGCCGGA<br>AAGCCTGATTACCGAATATGTTATGGCAAATCATCCGCGTTTTAAAGATACCCCGATTTGGCGTGATCGTAATCACACCA<br>CACGTATTGAAGGTGGTGATGAACTGATTCTGAATAAAACCACCGTTGCAATTGGTGTTAGCGAACGTACCAGCAGCAAA<br>ACCATTCAGAACCTGGCAAAAGAACTGTTTGCAAATCCGCTGAGCACCTTTGATACCGTTCTGGCAGTTGAAATTCCGCA<br>TAATCATGCAATGATGCATCTGGATACCGTGTTCACCATGATTAACCATGATCAGTTTACCGTGTTTCCGGGTATTATGG<br>ATGGTGCAGGTAATATCAATGTGTTTATTCTGCGTCCGGGTAAAGATGATGAAGTGGAAATTGAACATCTGACCGATCTG<br>AAAGCAGCACTGAAGAAAGTTCTGAATCTGAGCGAACTGGATCTGATTGAATGTGGTGCCGGTGATCCGATTGCAGCACC<br>GCGTGAACAGTGGAATGATGGTAGCAATACCCTGGCAATTGCACCGGGTGAAATTGTGACCTATGATCGCAATTATGTTA<br>CCGTGGAACTGCTGAAAGAACATGGCATCAAAGTTCATGAAATTCTGAGCAGCGAATTAGGTCGTGGTCGTGGTGGTGCA<br>CGTTGTATGAGCCAGCCGCTGTGGCGTGAAGATCTGTAACTCGAG

Primers used for ArcA WT cloning and C400A mutagenesis

Cterminal His: (NcoI/XhoI)		
**Name**	**direction**	**sequence**
ARCA_CHIS_F:	Forward	tgtttaactttaagaaggagatataccatggATGAATAATGGCATTAACGTG
ARCA_CHIS_R:	Reverse	atctcagtggtggtggtggtggtgctcgagCAGATCTTCACGCCACAGCGGCTGG

C400A His: (SacI)		
**Name**	**direction**	**sequence**
ARCA_C400A_F	Forward	GAATTAGGTCGTGGTCGTGGTGGAGCTCGTGCTATGAGCCAGCCGCTGTGG
ARCA_C400A_R	Reverse	CCACAGCGGCTGGCTCATAGCACGAGCTCCACCACGACCACGACCTAATTC

Sanger sequencing primers		
**Name**	**direction**	**sequence**
M13fwd	Forward	GTAAAACGACGGCCAGT
M13rev	Reverse	CAGGAAACAGCTATGAC
T7	Forward	TAATACGACTCACTATAGG
T7ter	Reverse	GCTAGTTATTGCTCAGCGG
