# Supplementary material for: The strain-dependent cytostatic activity of Lactococcus lactis on CRC cell lines is mediated through the release of arginine deiminase
Source: Microb Cell Fact. 2024 Mar 14;23:82. doi: 10.1186/s12934-024-02345-w (PMC10938756; doi:10.1186/s12934-024-02345-w)
Supplement: Supplementary file 4 — Supplementary Material 4 [file 12934_2024_2345_MOESM4_ESM.docx]

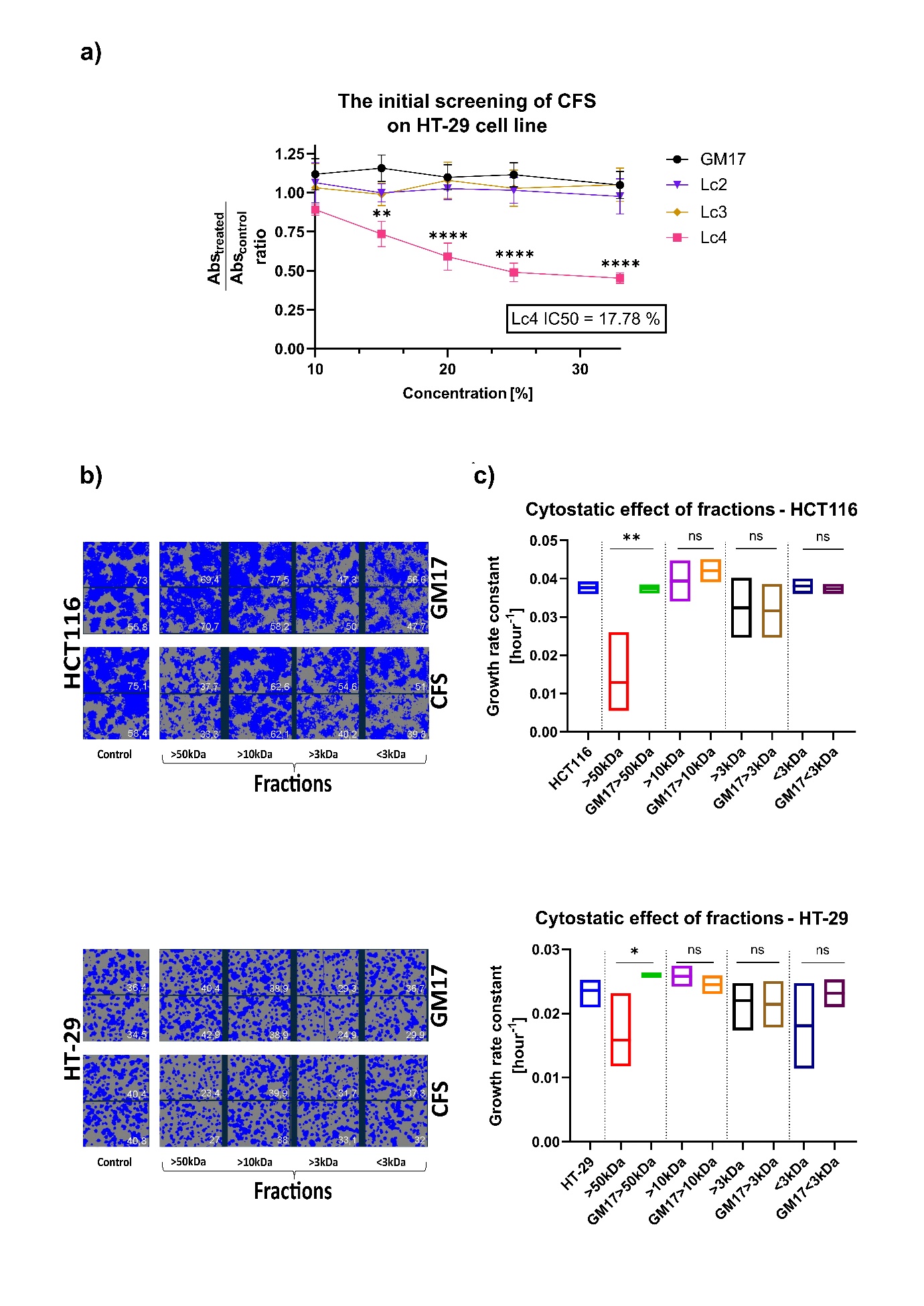


**Supplementary Figure 1. The cytostatic activity of cell-free supernatants of three strains of *L. lactis* and Lc4 strain fractions obtained via molecular filtration.** a) MTS assay results after 72h treatment with CFSs of *L. lactis* strains Lc2, Lc3, Lc4, and GM17 medium (control) on HT-29 cell line. Five concentrations of each supernatant were tested (10%, 15%, 20%, 25%, 33% v/v). For Lc4, the IC50 value was calculated using a four-parameter dose-response curve. Data are presented as mean +/- SD (n=3 independent biological replicates). The ratio was calculated by division of absorbance obtained for cells treated with CFSs at a desired concentration by absorbance of GM17-treated cells at an equal concentration. b) Visual representation of cytostatic effects of Lc4 strain CFS fractions obtained by molecular filtration as a proxy of confluence differences after 72h of experiment. DMEM medium was used as a control. Images were created using IncuCyte rev2 software. c) Cell growth rate constant after treatment with fractions obtained by molecular filtration (n=3 independent experiments). Fractions were diluted to the initial volume before the experiments in the DMEM medium. Cells were treated with a final concentration of 33% (v/v). Data are presented as mean with min to max range. (*p<0.05, **p<0.01, ***p<0.001, ****p<0,0001 by one-way ANOVA with Fisher LSD post-hoc.

**
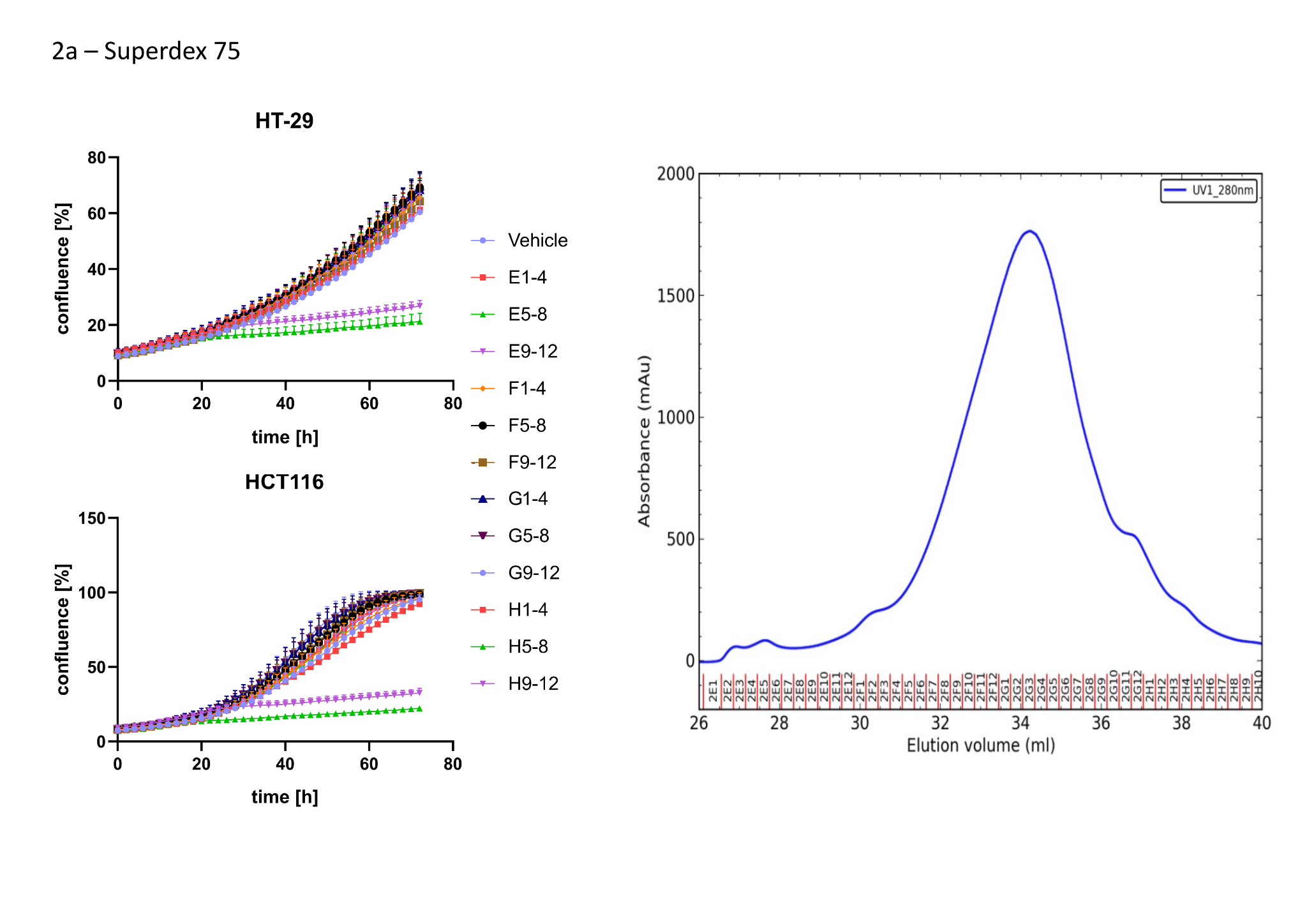
**

**Supplementary Figure S2a. The cytostatic activity of fractions obtained through gel filtration using the Superdex 75 column.** Left side: cytostatic activity of pooled GF75 fractions (on HT-29 and HCT116 cell lines). The final concentration of each pooled fraction was 5% (v/v). Right side: chromatogram of the separation process, with collected fractions marked on the x-axis.


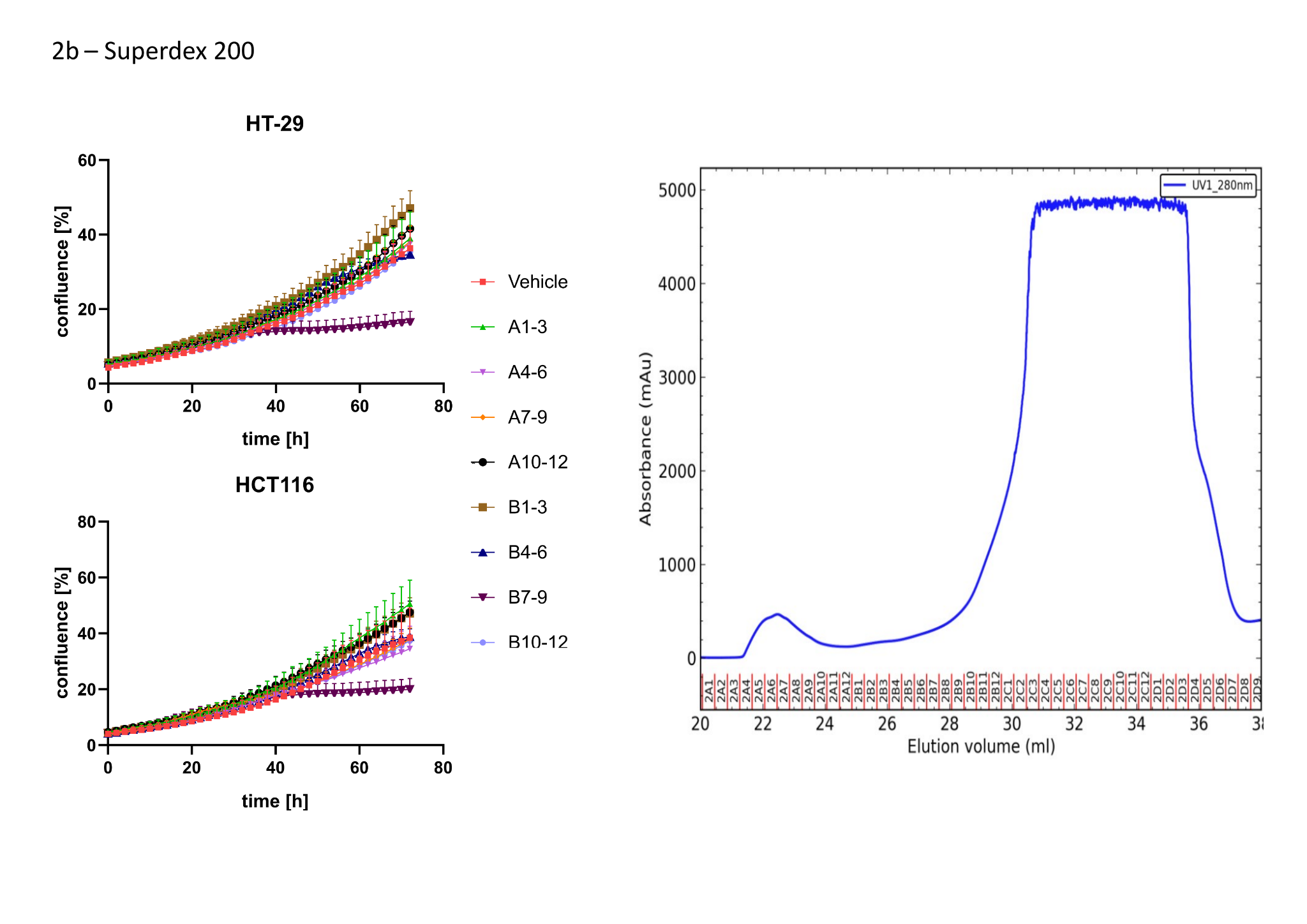


**Supplementary Figure 2b. The cytostatic activity of fractions obtained through gel filtration using the Superdex 200 column.** Left side: cytostatic activity of pooled GF200 fractions (on HT-29 and HCT116 cell lines). The final concentration of each pooled fraction was 5% (v/v). Right side: chromatogram of the separation process, with collected fractions marked on the x-axis.


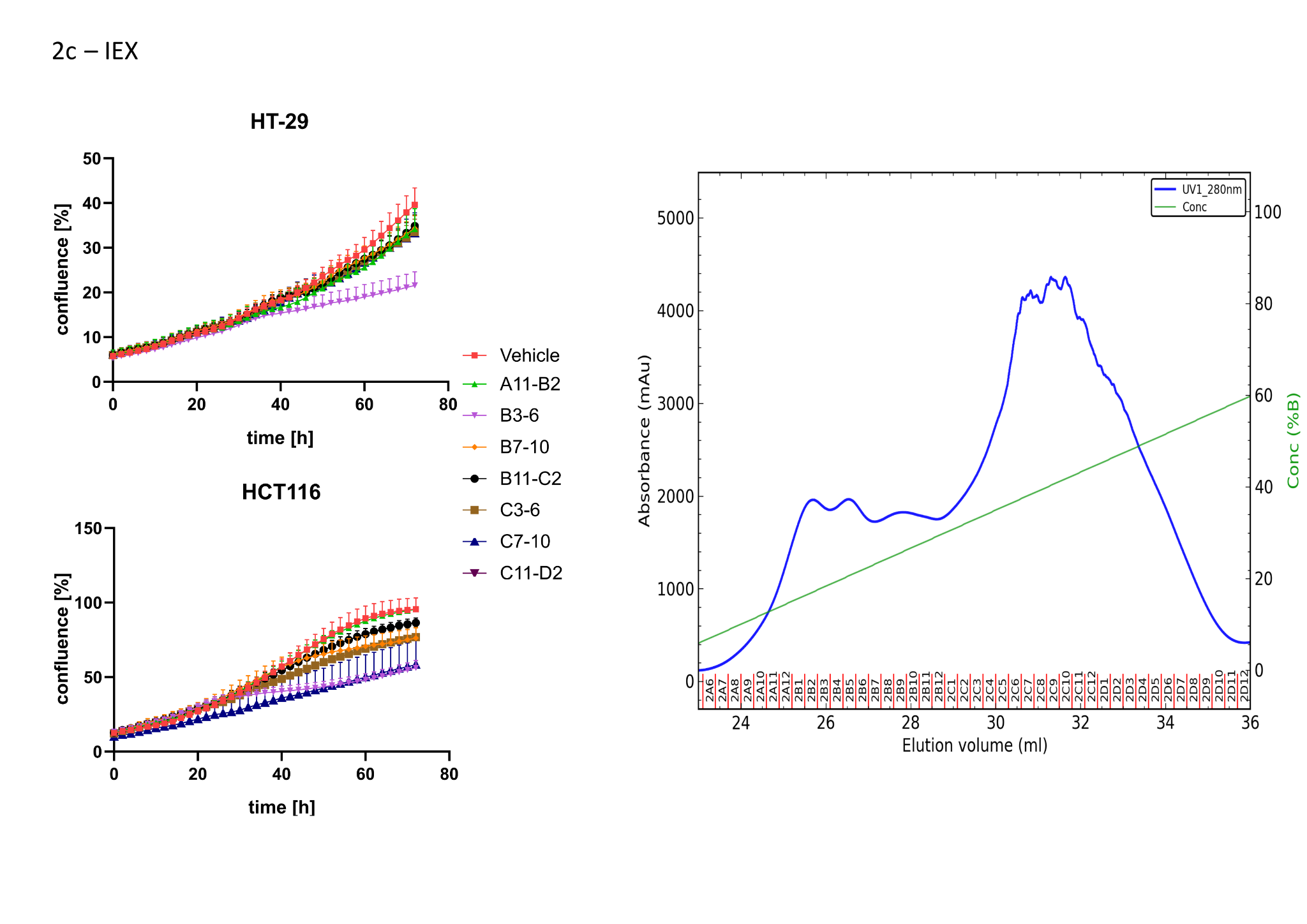


**Supplementary Figure 2c. The cytostatic activity of fractions obtained through ion exchange (IEX) chromatography using Resource Q column.** Left side: cytostatic activity of pooled IEX fractions (on HT-29 and HCT116). The final concentration of each pooled fraction was 5% (v/v). Right side: chromatogram of the separation process, with collected fractions marked on the x-axis


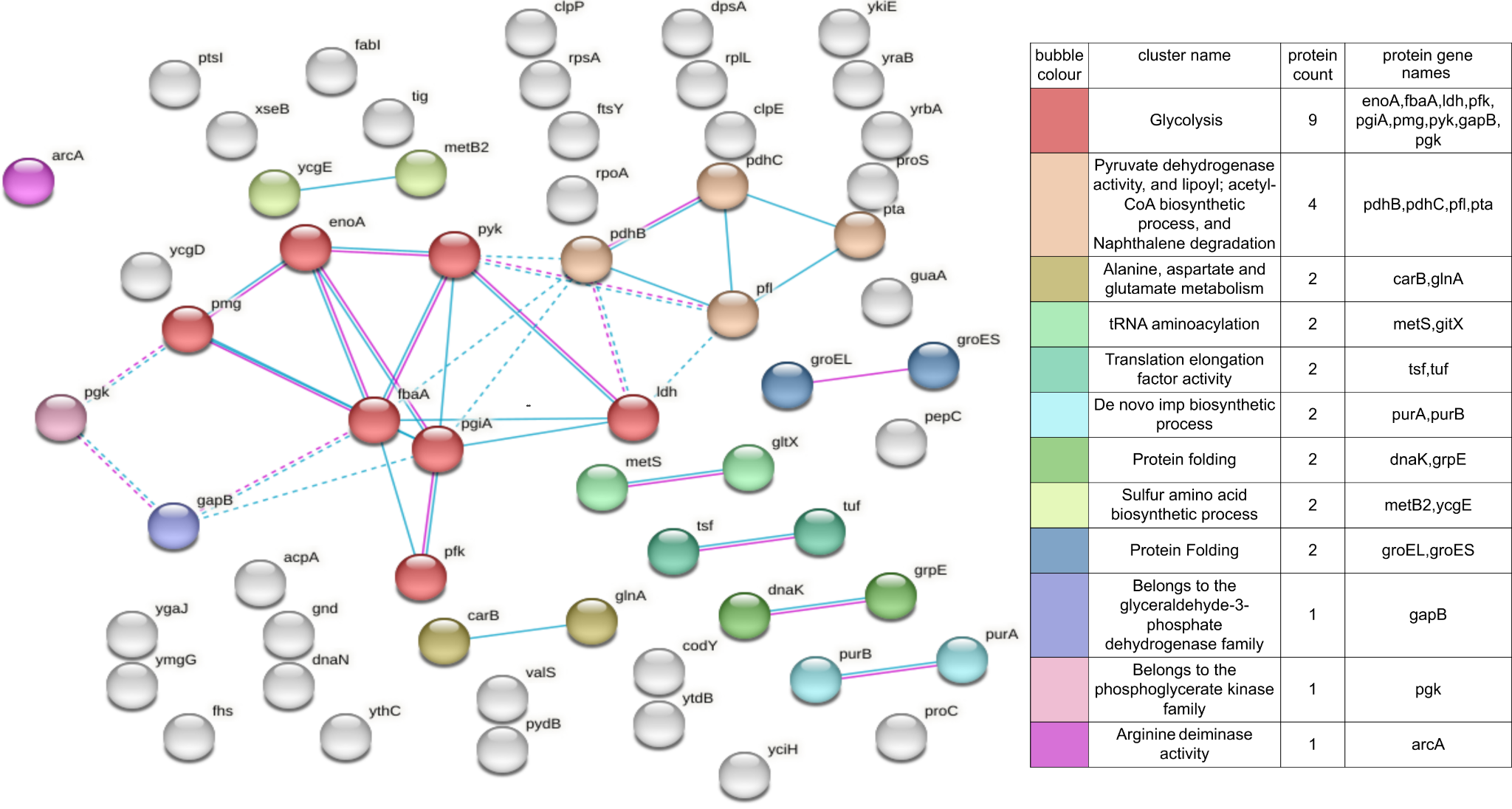


**Supplementary Figure 3. STRING analysis of 59 MS protein hits found common in F-GF200, F-GF75, and F-IEX active fractions.** Left: colored lines depict interactions with high experimental confidence (0.7) (purple line) and curated database mentions (cyan line). Protein genes with no interactions are colored gray, excluding ADI - arginine deiminase, ArcA (purple). Right: Based on STRING interaction analysis, proteins were clustered using the MCL algorithm (STRING module).


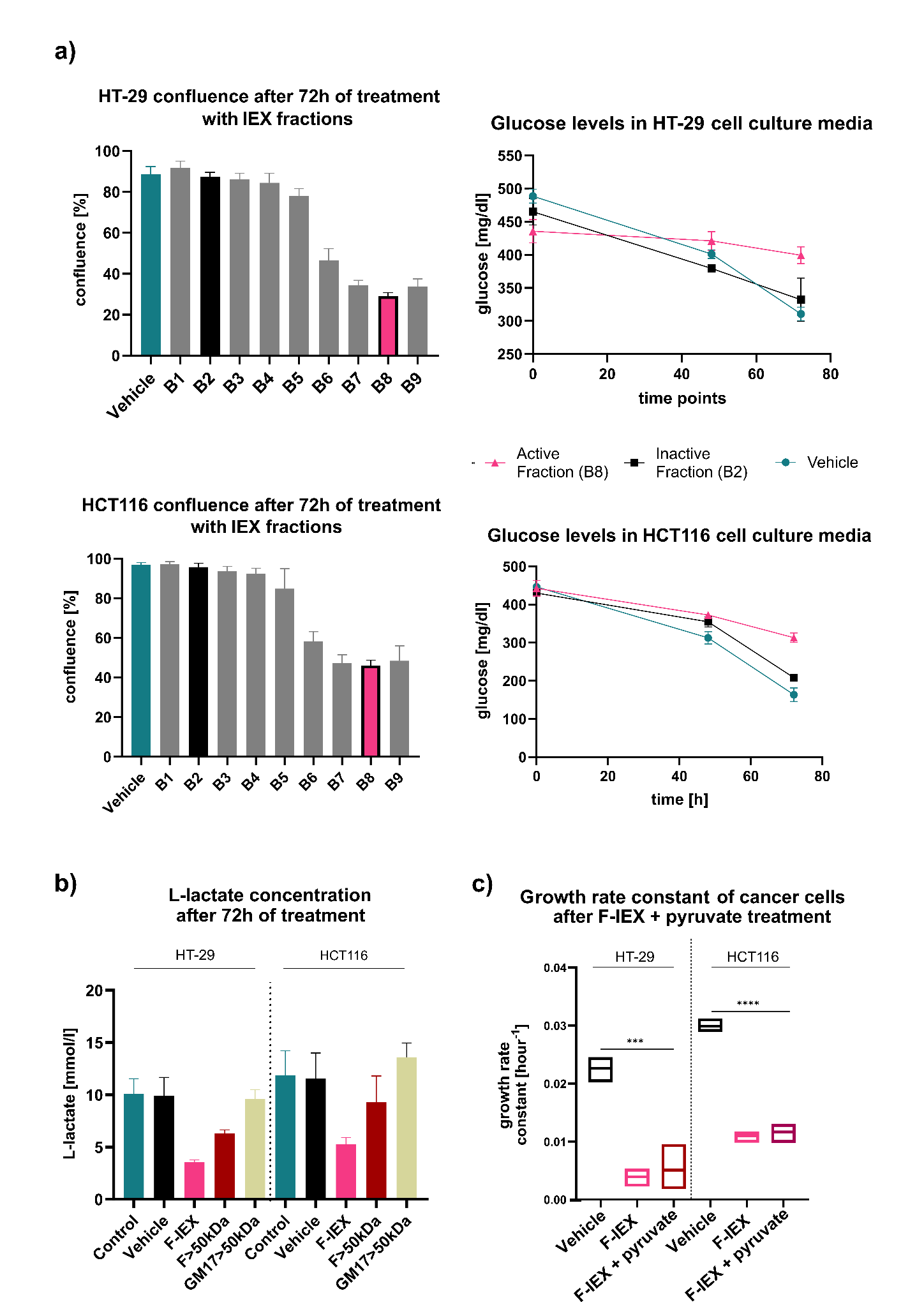


**Supplementary Figure 4. The cytostatic activity of F-IEX is not related to glucose or pyruvate consumption and increased lactate production.** a) The cytostatic activity of various F-IEX fractions on HT-29 (top) and HCT116 (bottom) cells after 72h of treatment (5% v/v), and glucose changes during F-IEX active (B8) and inactive (B2) fractions treatment. Data presented as mean +/- SD from N=2 independent experiments. b) L-lactate levels after 72h of treatment of HT-29 and HCT116 cells with vehicle (0.35 M NaCl + 10 mM HEPES buffer), F-IEX, F>50kDa and GM17>50kDa (5% v/v). c) Cytostatic activity of F-IEX fraction after the addition of 3 mM of pyruvate to HT-29 and HCT116 cells (N=3 independent experiments).


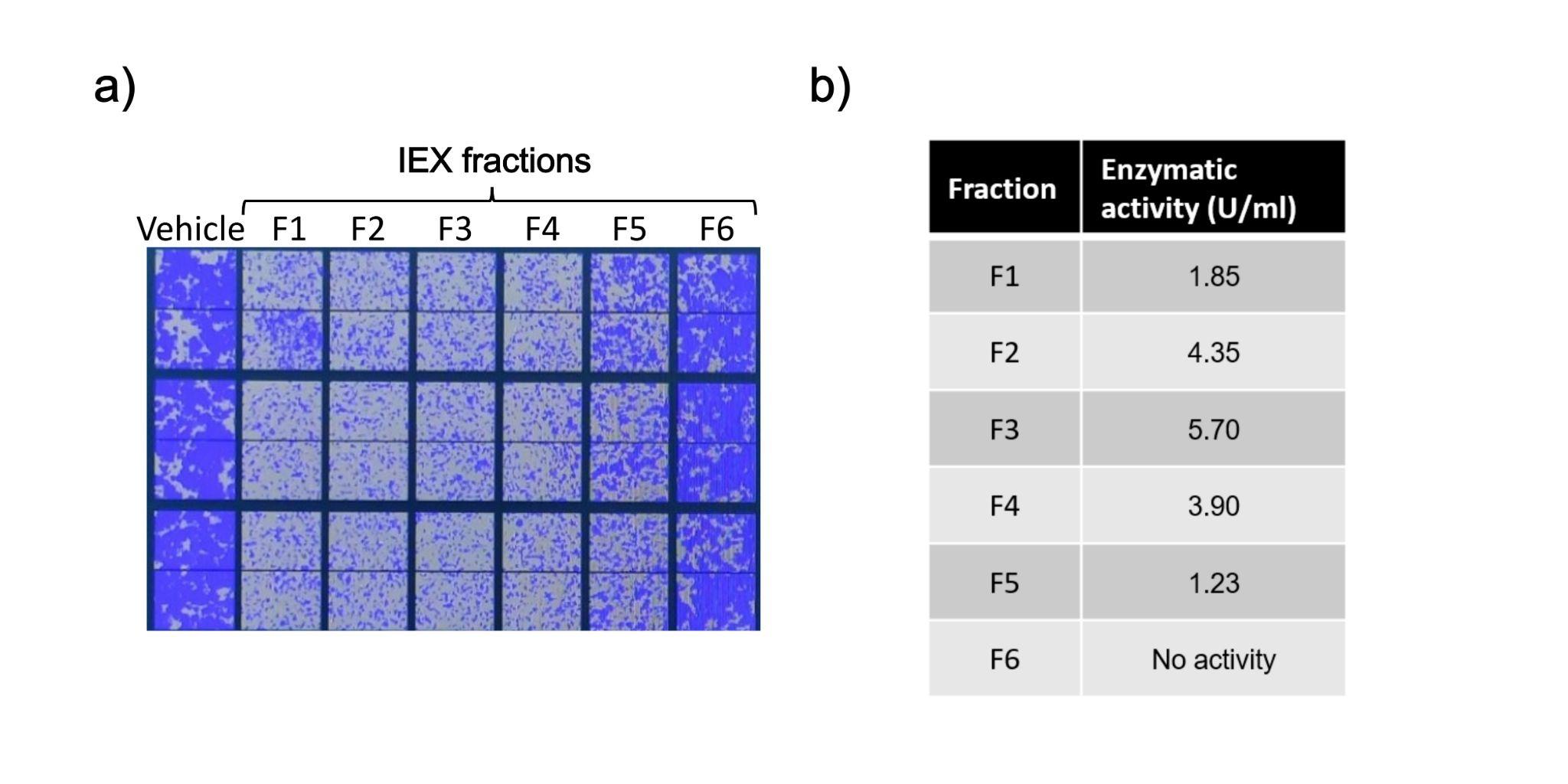


**Supplementary Figure 5. The cytostatic activity of F-IEX fractions is associated with the enzymatic activity of ADI.** a) Visual representation of the cytostatic effects of F-IEX fractions on HCT116 cell line as a proxy of confluence differences after 72h of experiment. Fractions were obtained from the purification of F>50kDa. Images were created using IncuCyte rev2 software. b) ADI enzymatic activity of fractions examined in a).


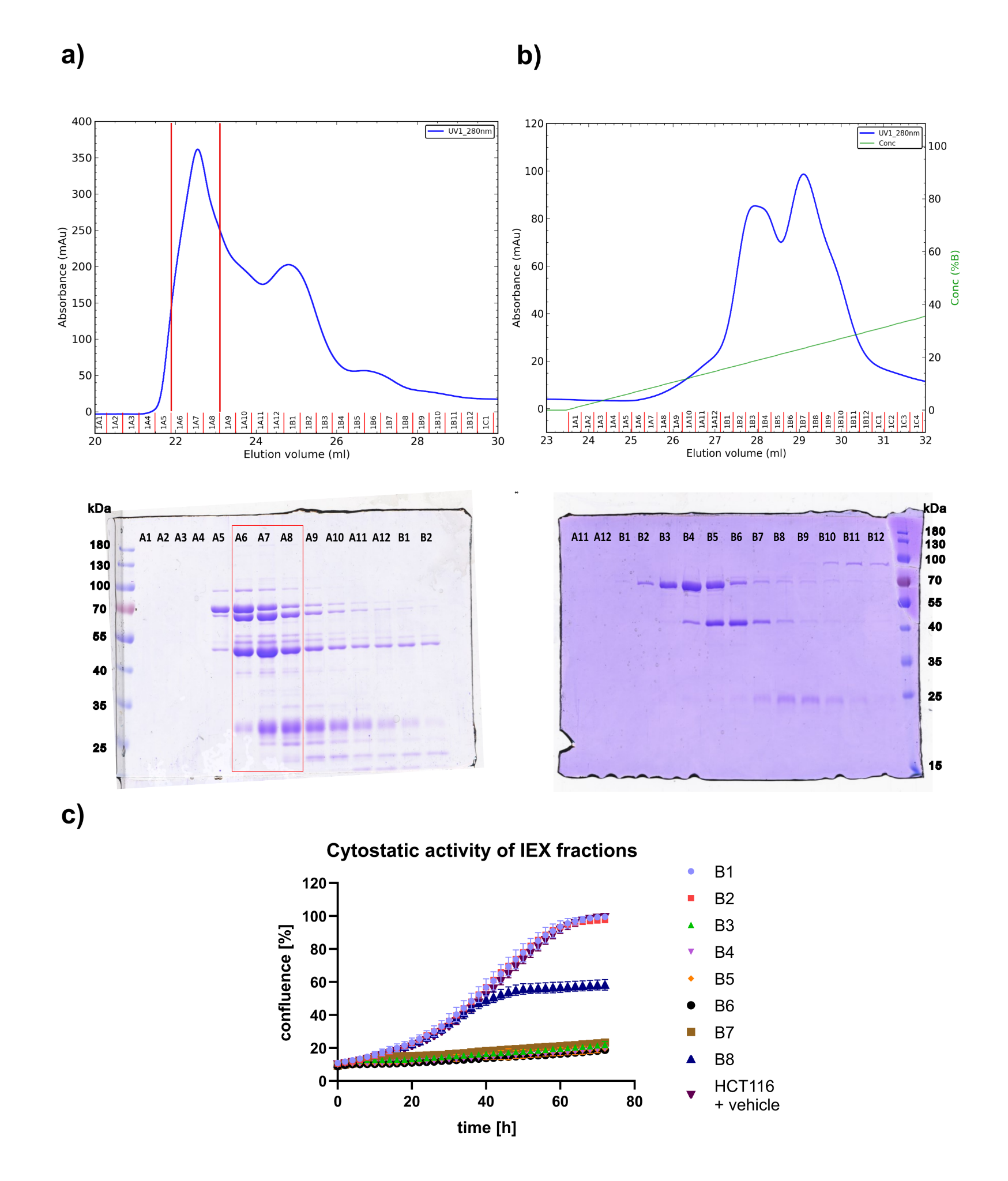


**Supplementary Figure 6. Purification of heterologously expressed recombinant ADI WT protein and cytostatic activity of obtained fractions from the final step of purification via ion exchange chromatography.** a) Second step of ADI WT purification. Representative chromatogram and SDS-PAGE gel analysis of fractions obtained following the purification of 1 ml of eluate from NiNTA affinity resin on Superdex 75 size exclusion column. The red rectangle on the SDS-PAGE electropherogram represents the fractions that were used in the subsequent step of purification presented in panel b. b) Third step of ADI WT purification. Representative chromatogram and SDS-PAGE analysis of fractions obtained following the purification of selected fractions from a) (A6, A7, A8) on Resource Q ion exchange (IEX) column. c) Representative chart of confluence changes of HCT116 cells over time (0-72h) after treatment with fractions obtained in b (1.25% v/v for each fraction) and vehicle (0.35 M NaCl + 10 mM HEPES buffer – 1.25% v/v). Scans were performed using the IncuCyte S3 device every 2h. Each time point presents mean +/- SD.


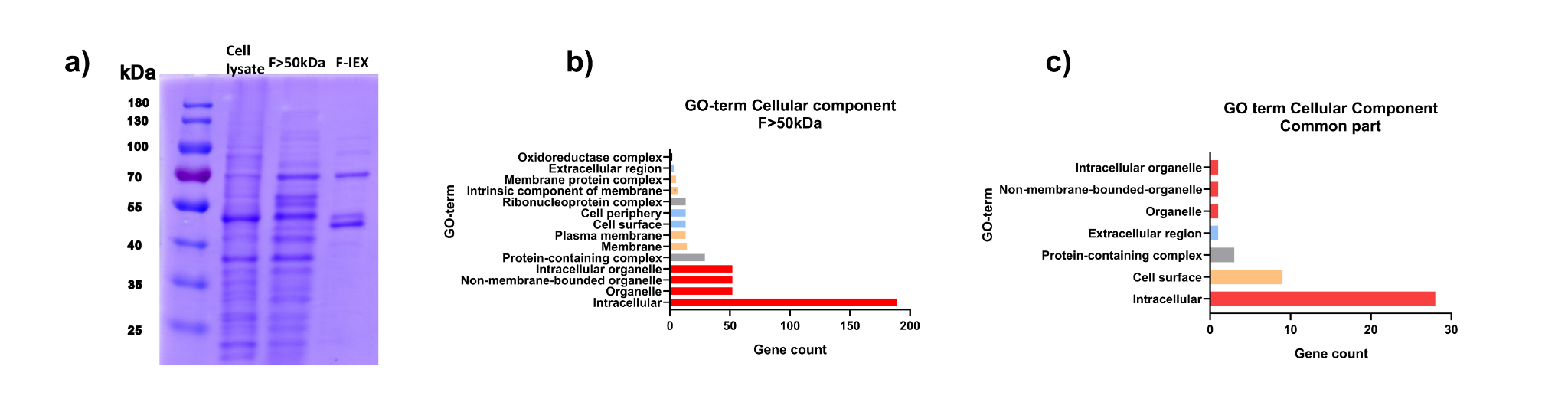


**Supplementary Figure 7. Most proteins present in F>50kDa and in the common part of F-GF75, F-GF200 and F-IEX active fractions have an intracellular origin.** a) SDS-PAGE gel-based comparison of the cell lysate, F>50kDa, F-IEX protein profiles after Coomassie R250 Blue staining. b) Number of proteins in F>50kDa fraction with GO-term annotation of their cellular localization. Red color indicates intracellular origin; light orange - associated with the membrane, light blue – extracellular, other GO-terms are colored gray. Analysis performed using ShinyGO. c) Number of proteins in the common part of F-GF75, F-GF200 and F-IEX active fractions with “cellular component” GO-term annotation. Color code and analysis performed identically as in b).


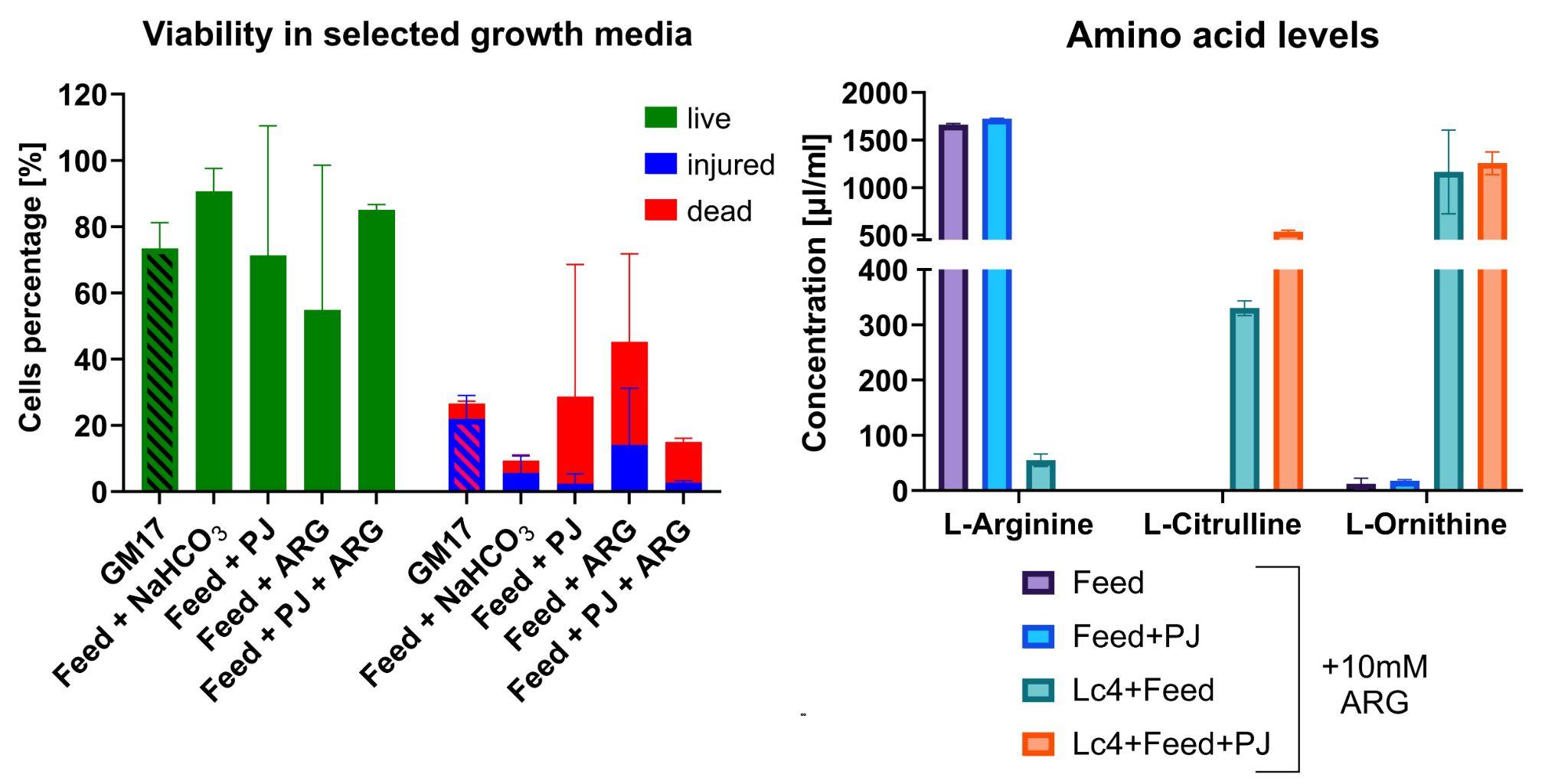


**Supplementary Figure 8. Viability and ADI enzymatic activity of Lc4 strain in SHIME Feed.** Left: viability in different combinations of SHIME feed, PJ, and ARG after 24 h of cultivation. Right: amino acid levels with and without Lc4 strain as a proxy of ADI-pathway enzymatic activity.


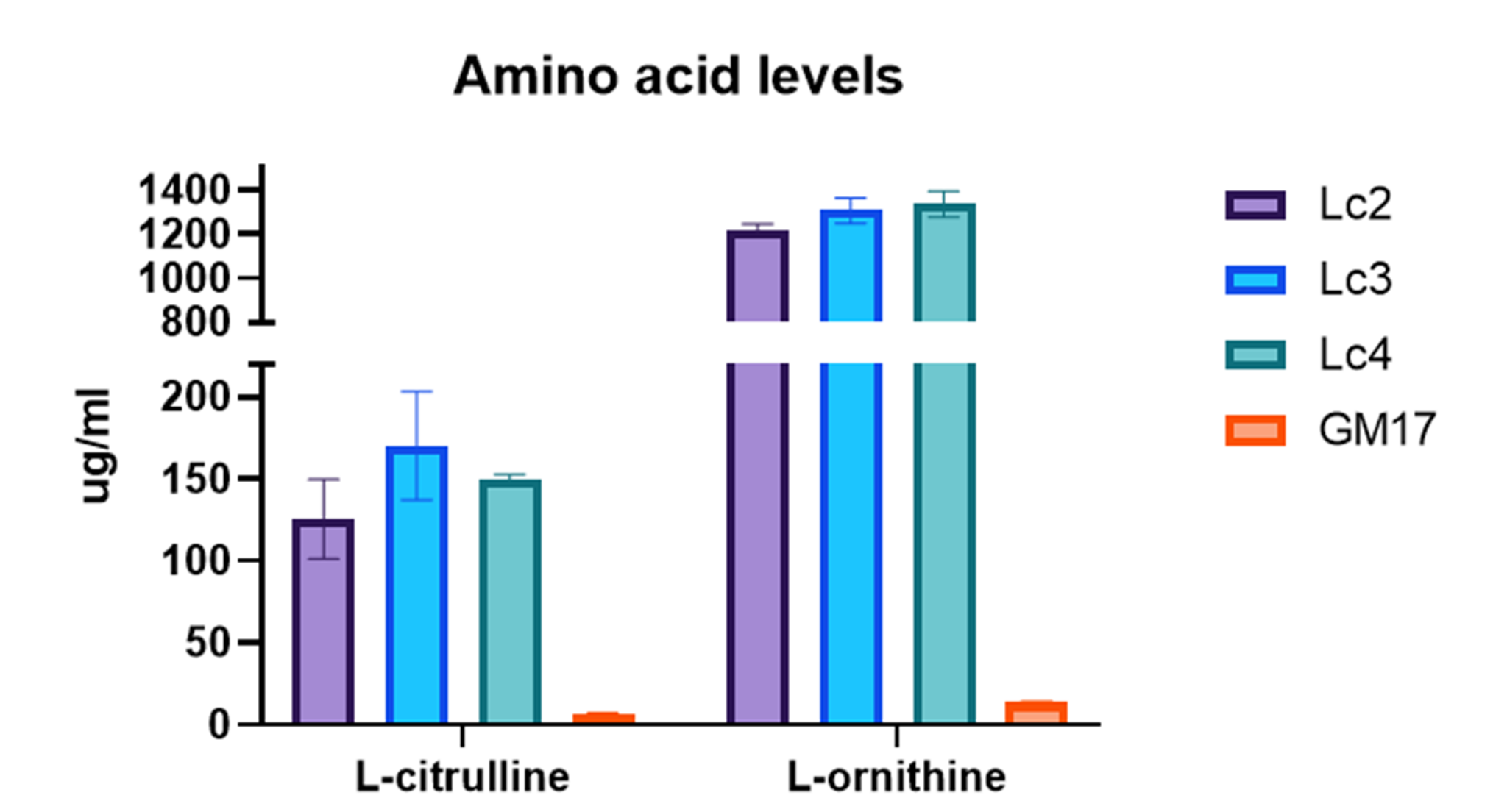


**Supplementary Figure 9. All tested L. lactis strains (Lc2, Lc3, Lc4) are able to produce L-citrulline and L-ornithine.** Strains were grown on GM17 medium with the addition of 15mM of L-arginine in conditions described in the Materials and Methods section. After 24h of cultivation, cultures were centrifuged, 0.22 µm filter-sterilized, and supernatants were subjected to LC-MS/MS analysis of amino acid content.


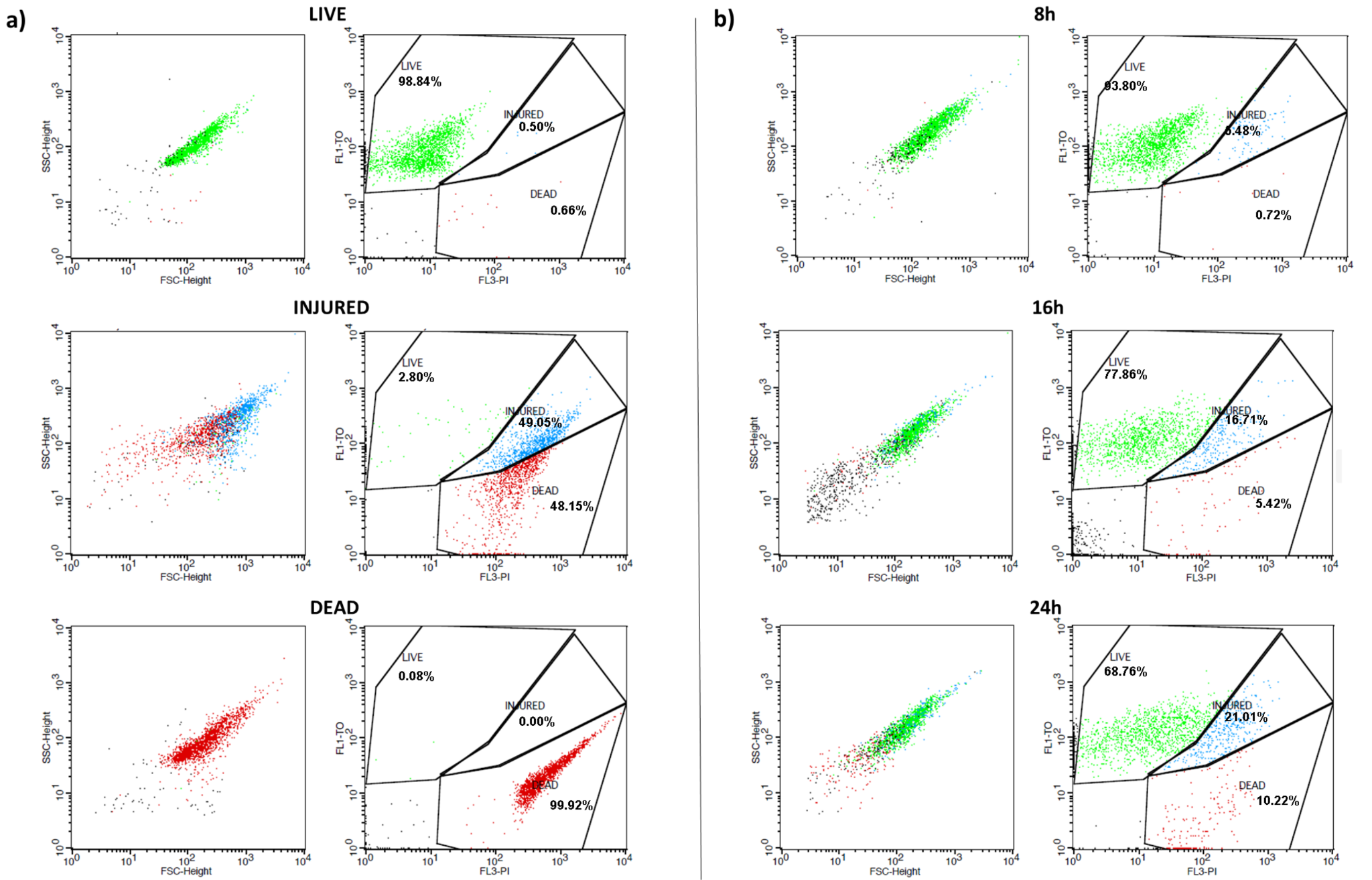


**Supplementary Figure 10. The presence of ADI protein in the supernatant is interconnected with changes in the viability of the L. lactis Lc4 strain during cultivation.** a) Representative diagrams of viability analysis of cells: live cells from the logarithmic growth phase (30℃, GM17 medium), injured by 5 min treatment with 0.05% Triton X-100 (Sigma Aldrich, cat. no. 93443), and dead cells, which were killed with 70% isopropanol. Based on the presented data, gates were adjusted for further experiments. b) Representative diagrams of viability analysis of Lc4 strain cells from time course experiments (see Figure 7a,b). Data are presented from top to bottom (8h, 16h, 24h).
